# Falling behind: life expectancy in US counties from 2000 to 2007 in an international context

**DOI:** 10.1186/1478-7954-9-16

**Published:** 2011-06-15

**Authors:** Sandeep C Kulkarni, Alison Levin-Rector, Majid Ezzati, Christopher JL Murray

**Affiliations:** 1 Institute for Health Metrics and Evaluation, University of Washington, Seattle, USA; 2 Imperial College, London, UK

## Abstract

**Background:**

The United States health care debate has focused on the nation's uniquely high rates of lack of insurance and poor health outcomes relative to other high-income countries. Large disparities in health outcomes are well-documented in the US, but the most recent assessment of county disparities in mortality is from 1999. It is critical to tracking progress of health reform legislation to have an up-to-date assessment of disparities in life expectancy across counties. US disparities can be seen more clearly in the context of how progress in each county compares to international trends.

**Methods:**

We use newly released mortality data by age, sex, and county for the US from 2000 to 2007 to compute life tables separately for each sex, for all races combined, for whites, and for blacks. We propose, validate, and apply novel methods to estimate recent life tables for small areas to generate up-to-date estimates. Life expectancy rates and changes in life expectancy for counties are compared to the life expectancies across nations in 2000 and 2007. We calculate the number of calendar years behind each county is in 2000 and 2007 compared to an international life expectancy time series.

**Results:**

Across US counties, life expectancy in 2007 ranged from 65.9 to 81.1 years for men and 73.5 to 86.0 years for women. When compared against a time series of life expectancy in the 10 nations with the lowest mortality, US counties range from being 15 calendar years ahead to over 50 calendar years behind for men and 16 calendar years ahead to over 50 calendar years behind for women. County life expectancy for black men ranges from 59.4 to 77.2 years, with counties ranging from seven to over 50 calendar years behind the international frontier; for black women, the range is 69.6 to 82.6 years, with counties ranging from eight to over 50 calendar years behind. Between 2000 and 2007, 80% (men) and 91% (women) of American counties fell in standing against this international life expectancy standard.

**Conclusions:**

The US has extremely large geographic and racial disparities, with some communities having life expectancies already well behind those of the best-performing nations. At the same time, relative performance for most communities continues to drop. Efforts to address these issues will need to tackle the leading preventable causes of death.

## Background

Over the past year, the United States has undergone a vigorous legislative and public debate on reform of the health care system leading to passage of new health care legislation. The debate has highlighted the high and growing costs of health care to the nation, 16.2% of GDP in 2008 [1], and the nation's uniquely high rates of lack of insurance compared with other high-income countries [2]. To a lesser extent, the debate has also focused on poor health outcomes for the US relative to other nations [3]. Despite legislation, consensus on how to move ahead remains elusive.

US racial/ethnic and geographic health disparities are vast [4,5]. In the US, life expectancy ranged from 70.0 for black male Americans and 80.8 for white females [6] to 85.7 years for Asian-American females in 2007 [7]. Larger disparities across counties and race/ethnicity groups within counties have been documented [4,8]. These disparities have been growing since 1983, but the most recent assessment is from 1999, now more than a decade old [8]. Tracking county disparities after 2002 until 2009 was impossible, because the US government did not release county-level mortality data during this time period. The challenges for the US health care system are intimately intertwined with health outcome disparities. The lack of insurance and associated health risks will play out differently across US counties both because of huge variations in the numbers of people lacking insurance and behavioral, environmental, and social risks to health [9].

The US picture, with its remarkable combination of poor health outcomes despite the highest levels of health spending per capita, is even more stark and disturbing when examined at the local level. Both as an input to the ongoing reform process and as a baseline for future changes in the public health and medical care systems, we believe it is essential to track county and race-county health outcomes in a timely fashion. In this study, we develop life tables for US counties in 2000 and 2007. We compare life expectancies of counties to those of the lowest-mortality nations to assess both absolute and relative progress for each county.

## Methods

We applied a statistical model to estimate annual life expectancy for counties in the US for the years 2000 through 2007, the latest year with available data.

### Modeling approach

There are three broad approaches for estimating health outcomes for small areas where directly observed data are unreliable or unstable due to small numbers of observations: pool multiple years of data, borrow strength across geospatial units, or use structured relationships with covariates (Srebotnjak T, Mokdad AH, Murray CJL: **A novel framework for validating and applying standardized small area measurement strategies**, submitted). Our method integrates these three approaches to measure mortality for each county and year. Specifically, we used a mixed-effects Poisson regression with time, geospatial, and covariate components. Poisson regression fits count outcome variables, e.g., death counts, and is preferable to a logistic model because the latter is biased when an outcome is rare (occurring in less than 1% of observations). The model is specified below:

where *y_rjt _*is the death count for race *r *within county *j *in year *t. Income_jt _is *county per-capita income for year *t. Education_j _*is the percent of adults within county *j *having completed high school in the 2000 census data. *Race *is a dummy variable for three race groups (white, black, and other). We've grouped Asians and Native Americans into a single category to reduce the sensitivity of the model to known racial miscoding in population and death counts.  is the geospatial component, calculated as the average of the posterior mode of the county random intercept for counties adjacent to county *j *to account for residual spatial patterns. The values for  are derived from running as a prior step the same model without the geospatial component to derive the posterior values of the county random effect. *μ_j _*is the posterior value of the county random intercept. *t *is the calendar year of mortality, and *γ_j _*is a random slope on time for each county. This specification allows mortality in each county to have a unique trend. The county population size affects the contribution of the random components on death counts, leading to more emphasis on recorded death counts when predicting mortality for larger counties.

The model was estimated separately by sex and five-year age groups because the magnitude of the county random effect varies by age. The outcome of the analysis is a predicted age-, sex-, and race-specific death count for each county in the year of analysis. We used these counts, together with corresponding population figures, to calculate sex-specific and sex-/race-specific life expectancy for each county [9]. We used the method proposed by Coale and Guo to estimate the years lived in the terminal age group of the life table [10]. To produce estimates for 2000, we use data from 1995-2000 inclusive, and for 2007, we use data for 2002-2007 inclusive.

Uncertainty in county life expectancy was calculated through simulation by drawing repeatedly from the posterior distributions of the race-, age-, and county-specific death counts.

### International comparisons

To show county mortality in an international context, we compared county life expectancy to an international life expectancy "frontier" time series, which we define as the average life expectancy of the 10 countries with the lowest mortality for each year from 1950 to 2010. We can use this series to calculate how many "years behind" each county is when compared to the frontier. For instance, if a county has a life expectancy in 2000 that is closest to the average life expectancy of the 10 leading countries in 1980, then we can say that this county is 20 years behind the international frontier. We calculate the number of calendar years behind each county is in 2000 as well as in 2007. This approach has been used in previous work on socioeconomic and health disparities [11-14].

### Units of analysis

The 3,147 US counties were arranged into 2,357 merged county clusters, each consisting of a single county or multiple counties. There were two reasons for this merging. First, merged county units are consistent over time even when there have been changes in county definitions and borders. Second, annual death counts in many counties remain too small for stable estimates. Merging counties overcomes this limitation. Counties with fewer than 7,000 males or 7,000 females were joined with contiguous counties in the same state of similar size, income, and percent of population that is black or Native American, until the cluster met this population cutoff.

### Data sources

Mortality data, including county of residence and cause of death certified and coded according to the International Classification of Diseases system, were obtained from the National Center for Health Statistics (NCHS). Standard public-use mortality files do not include geographic identifiers for deaths in counties with fewer than 100,000 people. We obtained county identifiers for all deaths for years 1959 to 2007 through a special request to the NCHS. County populations by age and race prior to 1990 were accessed through the US Census Bureau, and for 1990 and later, through the NCHS [15]. Estimates of county per-capita income were taken from the Bureau of Economic Analysis. Data on educational attainment came from the 2000 census. International estimates and projections for population and deaths were taken from the United Nations Population Division.

### Model validation

We validated the performance of the model by creating small counties whose "true" underlying death rates were known. We did this by treating counties with large populations (> 750,000) as those where death rates have little sampling uncertainty. We then repeatedly sampled residents and deaths from these counties (by year and sex) to construct simulated small-county populations. We used the above model to predict mortality for these small, sampled-down counties, which were then compared with the mortality of the original large county. Model validation shows that when sex-specific county population reaches 7,000, the correlation between model predictions and true life expectancy approaches 0.90 for both sexes and root mean squared error approaches 1.0 year (Table 1). At every sample size, the inclusion of the covariates and geospatial components improves the correlation and reduces the root mean squared error by as much as 50%. We provide in the Web appendix (Additional file 1) details on the performance of other models, including those based only on temporal pooling, on temporal pooling and a geospatial component, and on temporal pooling and covariates. These models do not perform as well as the model presented here.

**Table 1:** Statistics on model performance

	Male		Female	
**Sample**	**Root mean squared error**	**Correlation**	**Root mean squared error**	**Correlation**

**1000**	1.18	0.85	1.03	0.78

**2000**	1.14	0.86	0.94	0.83

**5000**	0.96	0.91	0.86	0.86

**7000**	0.95	0.91	0.79	0.89

**10000**	0.79	0.94	0.79	0.88

**50000**	0.49	0.98	0.55	0.95

Sample indicates the size of the small county drawn from large counties, where sampling error of death rates is negligible. Root mean squared error and correlation are all relative to the (true) life expectancy of the large county. See Methods for further details.

All analyses were done using Stata version 10.1.

## Results

**Figure 1:**
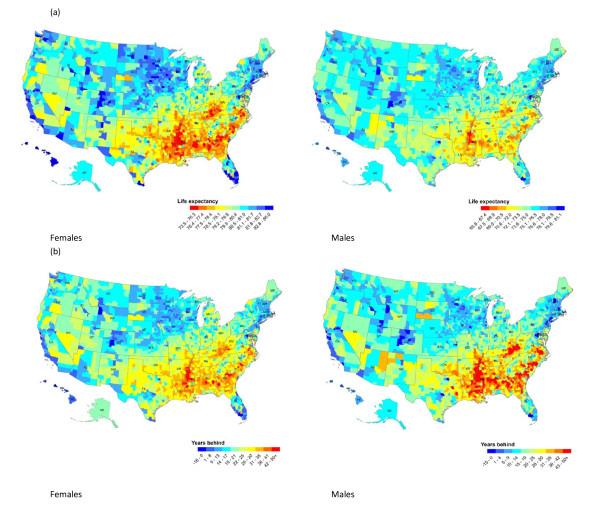
**(a) County life expectancy in 2007; (b) calendar years behind or ahead of the international frontier in 2007**.

In 2007, life expectancy at birth for American men and women was 75.6 and 80.8 years, ranking 37^th ^and 37^th^, respectively, in the world. Across US counties, life expectancy at birth ranged from 65.9 to 81.1 years for men and 73.5 to 86.0 years for women (Figure 1a). Geographically, the lowest life expectancies for both sexes were in counties in Appalachia and the Deep South, extending across northern Texas. Counties with the highest life expectancies tended to be in the northern Plains and along the Pacific coast and the Eastern Seaboard. In addition to these broad geographic patterns, there are more isolated counties with low life expectancies in a number of western counties with large Native American populations. Clusters of counties with high life expectancies for males and females are seen in Colorado, Minnesota, Utah, California, Washington, and Florida.

Another way of analyzing local patterns of life expectancy is to compare them to a life expectancy time series of the best-attained life expectancy in each year, measured by the average life expectancy in the top 10 countries. National life expectancy in the US in 2007 was lower than the international frontier by 3.2 years (13 years behind) for men and 3.2 years (16 years behind) for women. In 2000, county-level life expectancies range from nine years ahead of the international frontier to over 50 years behind for men and one year ahead of the international frontier to 45 years behind for women. In 2007, county-level life expectancies range from 15 years ahead of the international frontier to over 50 years behind for men and 16 years ahead to over 50 years behind for women (Figure 1b). Thirty-three counties for men and eight counties for women have higher life expectancies than the average of the 10 leading countries in 2007. Ninety-two counties for men and two counties for women have life expectancies that are comparable to that of the 10 leading countries before 1957.

**Figure 2:**
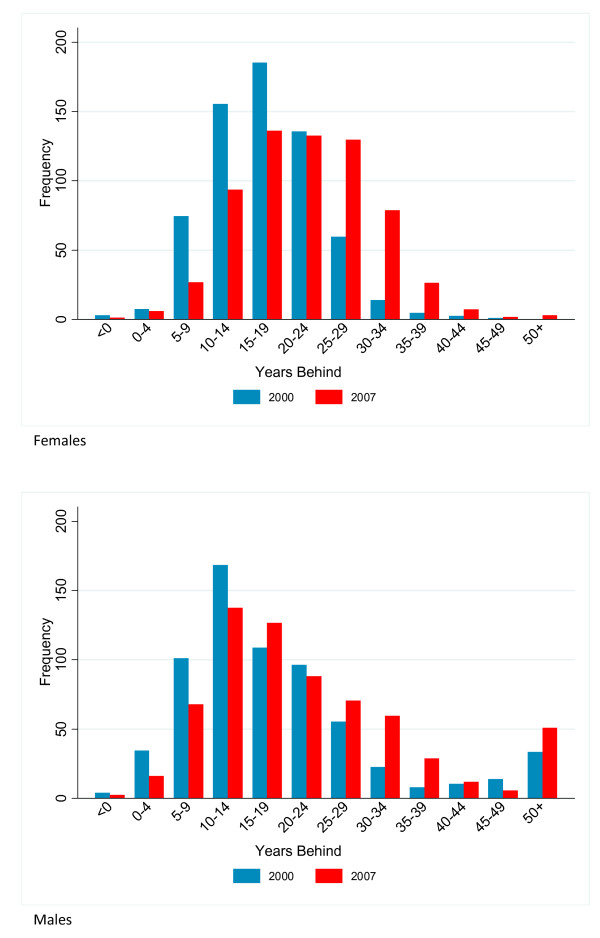
**Distribution of 2000 and 2007 calendar years behind or ahead of the international frontier for US counties**.

During the period 2000 to 2007, life expectancy in the US and most of its counties fell behind the progress seen in other nations. Over this period, 357 counties for men and 168 counties for women were fewer years behind the frontier metric in 2007 than they were in 2000. In contrast, 661 counties for men and 1,373 counties for women fell more than five more years behind the frontier between 2000 and 2007. Sixty-seven counties for males and 222 counties for females fell 10 or more years further behind the frontier. Figure 2 shows that for all counties, the distribution of years behind has shifted to the right from 2000 to 2007.

**Figure 3:**
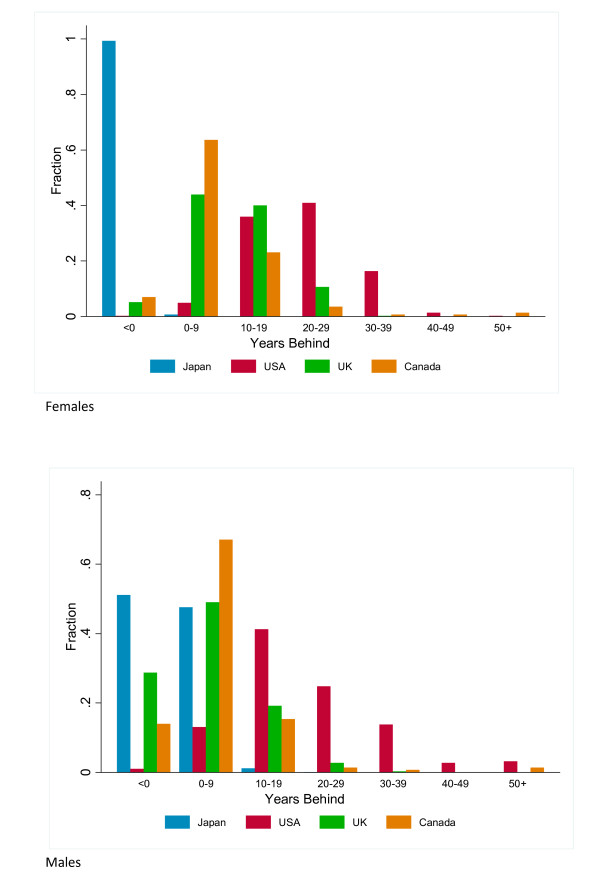
**Fraction of local areas in Japan, Canada, UK, and US falling into bins of calendar years behind or ahead of the international frontier**.

To further put US subnational variation in context, we have compared US counties with published data for three other high-income countries: Canada, the United Kingdom, and Japan. These published figures for life expectancy at the second administrative level have been computed by national statistical authorities using a variety of methods. While the methods are not completely comparable to ours, the methodological differences should not affect the broad patterns. For each of these datasets, we have compared local life expectancies to the international frontier (Figure 3). Women in 99% of the 1,964 municipalities in Japan in 2005 have a life expectancy that is higher than the international frontier, whereas for men, 25 municipalities are 10 years or more behind the frontier. Compared with Canada and the UK, the US has many more widespread disparities affecting a much larger fraction of counties. Only 0.2% of British local authorities and 2% of Canadian health areas for males have life expectancies that are more than 30 years behind the international frontier, compared to 17% of US counties for males. Of note, Canada has a small number of communities largely composed of Inuit populations that perform poorly. This mirrors the outcomes seen for selected US Native American populations, but in Canada the poorest outcomes are actually worse than in the US.

**Figure 4:**
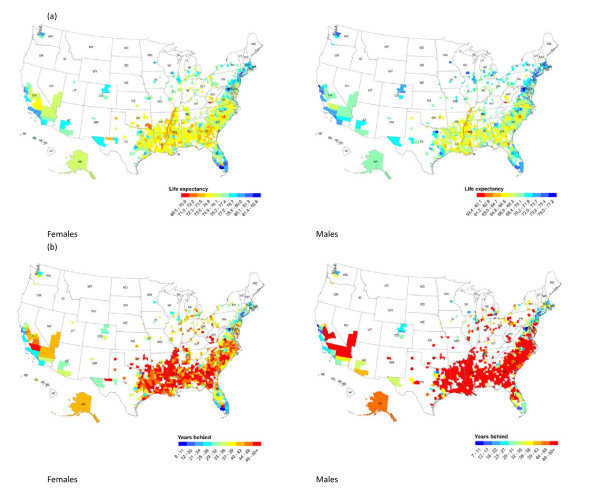
**(a) County life expectancy in 2007 for blacks; (b) calendar years behind or ahead of the international frontier in 2007 for blacks**.

We also computed life tables for 2007 for blacks at the county level where there are sufficient numbers of individuals in each race group to estimate a life table (Figure 4a). County life expectancies for black men, ranging from 59.4 to 77.2 years, are seven to over 50 years behind the international frontier, with 65% of counties having life expectancies that are over 50 years behind (Figure 4b). For black females, the range is from 69.6 to 82.6 years of life expectancy, which corresponds to eight to over 50 years behind the international frontier, although only 22% of counties are over 50 years behind. The pattern of life expectancy performance versus the international frontier for white Americans is similar to all races combined, reinforcing the point that poor relative performance of the US is not simply due to racial disparities.

**Figure 5:**
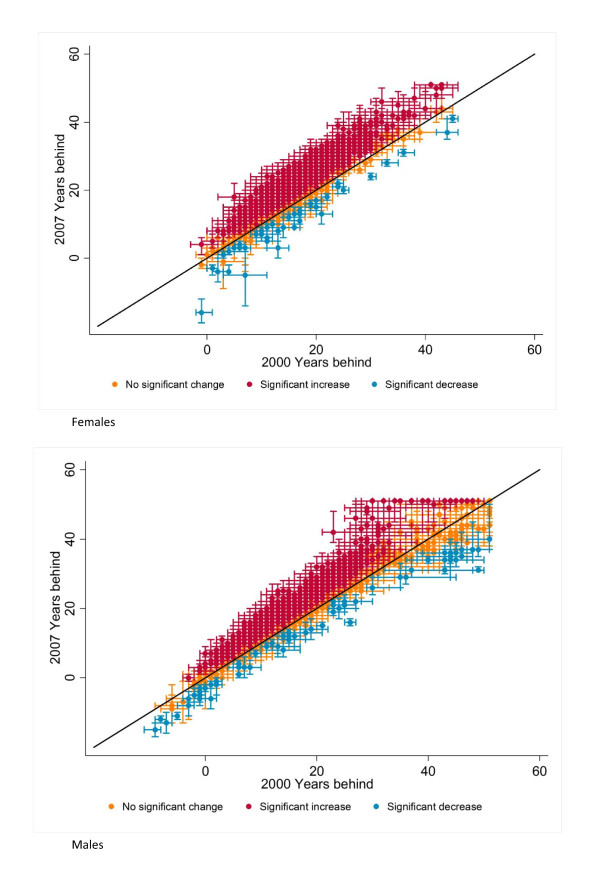
**2000 versus 2007 calendar years behind the international frontier with 90% confidence intervals for all US counties**.

Figure 5 shows the results of the uncertainty analysis by plotting the 90% confidence interval of years behind or ahead of the international frontier for years 2000 and 2007. The figure shows that the conclusions about falling further behind the international frontier for many US counties are robust to the size of the uncertainty intervals. For males, 1,406 counties fell further behind in 2007 (statistically significant at the 90% level), 78 counties experienced a significant improvement, and 1,663 counties were neither statistically ahead nor further behind the international frontier in 2007 as they were in 2000. The corresponding figures for females are 2,054 for falling further behind, 45 for improvement, and 1,048 counties with no change. A full list of life expectancies and years behind with 90% confidence intervals is available in the Web appendix (Additional file 1). The Web appendix also features a figure comparing life expectancy in 2000 with 2007 for males and females with uncertainty intervals for each county in the US (Additional file 1).

## Discussion

Large disparities in health outcomes have been documented in the US in relation to race, community of residence, and individual and community socioeconomic factors [5,8,16-29]. Our analysis shows that community-level disparities in 2007 cover a range of global health experiences - from counties with life expectancies better than the best-performing nations to those lagging behind these nations by 50 or more years. The extent of geographic inequality is substantially larger in the US than in the UK, Canada, or Japan. Equally concerning is that between 2000 and 2007, more than 85% of American counties have fallen further behind the international life expectancy frontier, of which 55% were statistically significant at the 90% confidence level. While the US and most of its communities fell further behind, the US maintained its position as the country that spent the most per capita on health care throughout this period.

### Limitations

Our findings are consistent with earlier analyses that had considered disparities across all counties, by region, and by community socioeconomic status [5,8,22,26,30-33]. Nevertheless, we found that applying the models to populations of fewer than 7,000 was associated with increased error; this, in turn, limits the granularity of the analysis that can be undertaken. There is an inherent trade-off between timeliness of estimates for small areas and the extent to which one borrows strength from structured covariates and geospatial relatedness. In settings where policy or adverse social trends may have a rapid impact on mortality, these approaches may underestimate the pace of change in mortality. Our estimates for 2007 depend on the validity of estimates of population by age, sex, and race by county, which are forecasted based on the decennial census and other data sources on births, deaths, and migration. The further away from the census year the more inaccurate these figures may become. The 2010 census round will provide a more robust assessment once it is released. Issues of age misreporting, especially at older ages, may confound the estimates of life expectancy, especially for black populations [34,35]. There are also issues of race/ethnicity classification on the death certificate compared to self-reports in the census that could affect our estimates of white and black life expectancies at the county level [36]. It is conceivable that the observed life expectancy disparities and disparity trends could be partially explained by migration. However, the best available data on county-to-county migration rule this out as a plausible explanation [8]. The NCHS has revised US life expectancy estimates downwards [37] based on examination of Medicare data, which provides one mechanism to deal with differential age misreporting on the census and death certificates. In this study, we have not been able to incorporate these types of modifications into local estimates of life expectancy and, as such, we may be overestimating local life expectancies in the US.

### Interpretation of findings

While documenting the pace of relative global decline and rising disparities is novel and may surprise some, the quantitative findings based on national data will be disputed by few. In contrast, interpreting how the US came to be in this position and what to do about it will continue to be vigorously debated. We believe it is worthwhile to examine the explanations that may help inform potential solutions, a debate that may take a long time to be resolved. While this debate continues, we believe there is sufficient evidence to identify some practical actions that can, in part, reverse this trend. The debate on the causes of poor performance will focus on three sets of factors: the social, cultural, and physical environment; modifiable behaviors, diet, and metabolic risk factors; and the performance of the health system. The roles of these factors are, of course, not mutually exclusive, as the same death can be related to social and material deprivation, risk factor exposure, and the failure of the health system.

Strong relationships have been documented between race/ethnicity, individual or community income, income inequality, and mortality in the US. While these factors convincingly affect mortality, they do not fully capture the performance variation in the US. Americans in counties with above median income ranged from being 16 years ahead of the frontier to 47 years behind. Moreover, between 2000 and 2007, 85% of these counties fell further behind the international life expectancy frontier. These findings confirm at the local level similar observations found for advantaged groups nationally [38]. Any analysis of causes of disparities will draw substantial attention to poverty, inequality, race, and ethnicity, but some of the poor performance and falling performance must be related to other factors [3].

How much of the poor performance of the US is due to differences or less favorable trends in critical risks to health such as tobacco smoking, hypertension, diabetes, physical inactivity, obesity, LDL cholesterol, diet, and alcohol? At the national level, these risk factors together lead to close to one million premature deaths [39]. If the leading four risk factors were addressed (smoking, high blood pressure, elevated blood glucose, and adiposity), life expectancy in 2005 would increase 4.9 and 4.1 years, respectively, for males and females. Disparities across eight race-county groupings would reduce by approximately 20% [39]. Given that risk factor exposures vary by county, and based on evidence from state-level analysis that risk factor exposures are larger in places with higher mortality rates [40-42], we would expect that addressing these risk factors would also tend to narrow disparities. An analysis that takes into account county exposures will be critical to fully understand the potential to reduce disparities through preventable causes of death. This, however, will require improving the measurement of exposure to leading risk factors at the local level.

How much better would the US and US counties perform if the US had had a high-performance health system? The answer rests on three dimensions that inherently underlie any analysis of health systems. First, over what duration do we assess a high-performance health system? If performance over a long duration is considered, then causes such as tobacco, road traffic injuries, and HIV might have been largely prevented or substantially mitigated. A shorter duration perspective, on the other hand, assigns a more limited scope and role to health system performance. Second, a high-performance health system could not have taken action until the scientific basis for action was established. Until tobacco was demonstrated to be a hazard, one cannot blame the health system for not taking action. Third, once the scientific basis of actions was established, to what extent should a high-performance health system have taken action? Once tobacco consumption was identified as a major risk in the 1950s and 1960s, should a high-performance health system have pursued all means to reduce consumption? Or should it have only provided information, taxed tobacco, or banned smoking in public places? Many of the debates on the extent to which the US health system is to blame for poor outcome performance turn on the scope and intensity of science-based action, which have an ideological dimension. Some in the US favor a narrow view of the duration and scope of action for a high-performance health system. These proponents emphasize treatment of disease or pharmacological management of risks. Others take a broader view [43]. Forging a consensus view on mortality attributable to a low-performance health system may be challenging.

What can be done to address the poor - and worsening - national and local performance of US communities? The US health care reform debate has focused on three strategies: extend insurance to all, improve quality of medical care for those who get sick, and focus on preventable causes of death [3]. Published studies estimate that 44,789 deaths out of 2,401,584 over age 18 in 2005 are attributable to a lack of health insurance [44,45]. These figures may be underestimated by not taking into account the fact that insurance coverage is lowest in communities with the highest mortality rates. Even taking into account such underestimation, the number of deaths attributable to lack of insurance is dramatically too small to explain much of the poor international performance and disparities in the US.

Quality of care for disease events varies substantially across the US [46,47]. An extensive literature highlights differences in quality as a function of race/ethnicity, income, and geography [5,46,47]. Improvements in quality would certainly have an impact on national life expectancy and on disparities, but there are few studies that have quantified these effects. Comparisons for specific outcomes, including breast and prostate cancer survival and acute myocardial infarction, suggest that the US, on average, has higher quality than many of the countries with better health outcomes [48,49]. Better outcomes for cancer may be influenced by the nonrepresentativeness of the Surveillance Epidemiology and End Results (SEER) cancer registration system and the concentration of some cancers in ages over 65 with near universal health insurance through Medicare [50,51]. For other conditions such as diabetes, however, the US has worse outcomes in some studies [52]. The Organisation for Economic Co-operation and Development (OECD) quality indicators project [53] is attempting to generate comparable measures for 541 indicators, but to date, the data have been plagued with definitional and measurement issues. To put quality-of-care issues in full light, we argue that a more comprehensive attempt to assess mortality attributable to low quality of care in the US and the impact of low quality of care on disparities should be undertaken.

Addressing leading preventable causes of death could dramatically improve the international performance of a large fraction of US counties for both males and females. What can the US health system do to realize these potential health improvements? Risks can be divided into those requiring concerted national action or community action or those that can be addressed through primary care. National, state, or even local policies [54] may be effective for banning trans-fat and regulating salt in packaged and prepared food, tobacco and alcohol taxes and control, increasing financial and physical access to healthier diets such as omega-3 fatty acids and fruits and vegetables, and authorizing the use of incentives by employers, insurers, and others for risk factor modification. Community intervention may be important for promoting physical activity and tailoring screening for hypertension, blood sugar, and cholesterol to local culture and context. Expanded and enhanced primary care can be the key locus for more aggressive management of hypertension, cholesterol, blood sugar, and personalized interventions for tobacco and alcohol. Major limitations to prioritize preventable causes of death include the need for more primary care physicians [55,56] and implementation of research efforts to improve adherence. A health system push on preventable causes of death would not be easy, but it is a target that is technically possible and could make a major impact on US health and life expectancy rates at the national and local levels.

What could motivate people, communities, and providers to have an increased focus on preventable causes of death? Some risks can be tackled through national legislation, such as banning trans-fat in manufactured foods or increasing federal taxation on tobacco. However, we believe the combination of measurement, incentive-based financing, and local innovation will also be essential. Local measurement of the baseline level of key risks and their trends can help set priorities and evaluate performance. Given the diversity of demography, epidemiology, physical infrastructure, and health system organization at the local level, a single national solution may not be the most effective for all risks. What will work to increase the effective coverage of hypertension management in Native Americans on the Pine Ridge and Rosebud reservations and in Hispanic communities in Miami may be very different. Local innovation for addressing some preventable causes of death can be harnessed by using national and state funding to pay communities for risk reduction. The experience of the GAVI Alliance is instructive. Results-based financing is feasible, but it is imperative that measurement is undertaken independent of those with a stake in the results [57]. Given the poor performance of the US on health outcomes, a performance that is worsening each year, it is time for new thinking targeted to where the biggest impact can be made on health outcomes.

## Competing interests

All authors declare that they have no competing interests and therefore have nothing to declare with the exception of stating our core grant funding from the state of Washington.

## Authors' contributions

SCK developed and applied the model to estimate mortality by age, sex, and race by county. ALR contributed to model implementation and conducted other data analysis. ME and CJLM designed the overall study and analytical strategy. SCK, ME, and CJLM wrote the first draft and revised the paper. All authors have read and approved the final manuscript.

## Supplementary Material

Additional file 1 **Web appendix**. A figure comparing life expectancy in 2000 with 2007 with uncertainty intervals for each county in the US, details on the performance of other models, cross-county standard deviations for observed and predicted life expectancies, and a full list of life expectancies and years behind with 90% confidence intervals for all counties. Click here for file
