# Supplementary material for: Falling behind: life expectancy in US counties from 2000 to 2007 in an international context
Source: Popul Health Metr. 2011 Jun 15;9:16. doi: 10.1186/1478-7954-9-16 (PMC3141397; doi:10.1186/1478-7954-9-16)
Supplement: Additional file 1 — Web appendix. A figure comparing life expectancy in 2000 with 2007 with uncertainty intervals for each county in the US, details on the performance of other models, cross-county standard deviations for observed and predicted life expectancies, and a full list of life expectancies and years behind with 90% confidence intervals for all counties. [file 1478-7954-9-16-S1.DOC]

**Web Appendix**

**Figure 1: 2000 versus 2007 US county life expectancies with 90% confidence intervals.**

Female Male

**Table 1: Statistics on model performance. Sample indicates the size of the small county drawn from large counties, where sampling error of death rates is negligible. Root mean squared error and correlation are all relative to the (true) life expectancy of the large county.**

|  |  | Male | | Female | |
| --- | --- | --- | --- | --- | --- |
| **Model** | **Sample** | **Root mean  squared error** | **Correlation** | **Root mean squared error** | **Correlation** |
| Covariate and temporal-pooling | 1000 | 1.23 | 0.83 | 1.08 | 0.76 |
|  | 2000 | 1.2 | 0.84 | 0.98 | 0.82 |
|  | 5000 | 1.0 | 0.9 | 0.9 | 0.85 |
|  | 7000 | 0.99 | 0.9 | 0.82 | 0.87 |
|  | 10000 | 0.83 | 0.93 | 0.82 | 0.87 |
|  | 50000 | 0.51 | 0.98 | 0.55 | 0.95 |
| Geospatial and temporal-pooling | 1000 | 1.59 | 0.75 | 1.16 | 0.78 |
|  | 2000 | 1.5 | 0.81 | 1.09 | 0.81 |
|  | 5000 | 1.26 | 0.88 | 0.98 | 0.86 |
|  | 7000 | 1.19 | 0.9 | 0.89 | 0.9 |
|  | 10000 | 0.99 | 0.94 | 0.85 | 0.9 |
|  | 50000 | 0.64 | 0.98 | 0.61 | 0.96 |
| Temporal-pooling | 1000 | 1.98 | 0.81 | 1.42 | 0.75 |
|  | 2000 | 1.88 | 0.85 | 1.32 | 0.82 |
|  | 5000 | 1.55 | 0.93 | 1.19 | 0.85 |
|  | 7000 | 1.46 | 0.93 | 1.1 | 0.88 |
|  | 10000 | 1.25 | 0.95 | 1.04 | 0.88 |
|  | 50000 | 0.76 | 0.98 | 0.7 | 0.95 |

**Table 2: Cross-county standard deviations for observed and predicted life expectancies.**

|  | **Male** | | **Female** | |
| --- | --- | --- | --- | --- |
| **Year** | Standard deviation in observed life expectancy | Standard deviation in predicted life expectancy | Standard deviation in observed life expectancy | Standard deviation in predicted life expectancy |
| **1985** | 2.43 | 1.72 | 2.11 | 1.17 |
| **1986** | 2.43 | 1.71 | 2.11 | 1.15 |
| **1987** | 2.51 | 1.72 | 2.10 | 1.18 |
| **1988** | 2.52 | 1.78 | 2.19 | 1.21 |
| **1989** | 2.62 | 1.85 | 2.20 | 1.23 |
| **1990** | 2.63 | 1.91 | 2.18 | 1.26 |
| **1991** | 2.66 | 1.97 | 2.11 | 1.27 |
| **1992** | 2.68 | 2.03 | 2.19 | 1.31 |
| **1993** | 2.64 | 2.06 | 2.15 | 1.33 |
| **1994** | 2.62 | 2.08 | 2.07 | 1.33 |
| **1995** | 2.69 | 2.09 | 2.15 | 1.35 |
| **1996** | 2.65 | 2.09 | 2.22 | 1.37 |
| **1997** | 2.63 | 2.09 | 2.14 | 1.39 |
| **1998** | 2.66 | 2.09 | 2.18 | 1.40 |
| **1999** | 2.65 | 2.09 | 2.21 | 1.41 |
| **2000** | 2.68 | 2.11 | 2.24 | 1.43 |
| **2001** | 2.72 | 2.09 | 2.30 | 1.47 |
| **2002** | 2.80 | 2.12 | 2.40 | 1.52 |
| **2003** | 2.82 | 2.17 | 2.44 | 1.57 |
| **2004** | 2.98 | 2.19 | 2.53 | 1.55 |
| **2005** | 2.96 | 2.30 | 2.45 | 1.63 |
| **2006** | 2.99 | 2.35 | 2.46 | 1.67 |
| **2007** | 3.01 | 2.35 | 2.53 | 1.68 |

**Table 3: 2000 and 2007 life expectancy and years behind with 90% confidence intervals for males and females in all US counties.**

|  | **Male** | | | | **Female** | | | |
| --- | --- | --- | --- | --- | --- | --- | --- | --- |
|  | **2000 Life expectancy** | **2000 Years behind** | **2007 Life expectancy** | **2007 Years behind** | **2000 Life expectancy** | **2000 Years behind** | **2007 Life expectancy** | **2007 Years behind** |
| **ALABAMA** |  |  |  |  |  |  |  |  |
| **Autauga** | 72.1 (71.5, 72.4) | 23 (21, 25) | 72.9 (72.4, 73.4) | 26 (23, 28) | 78.4 (77.9, 78.9) | 21 (20, 23) | 78 (77.5, 78.5) | 30 (28, 32) |
| **Baldwin** | 74.1 (73.7, 74.4) | 13 (12, 15) | 74.4 (74.1, 74.8) | 19 (17, 20) | 79.8 (79.4, 80.1) | 15 (13, 17) | 80.3 (79.9, 80.7) | 19 (17, 21) |
| **Barbour** | 69.8 (69.3, 70.2) | 36 (32, 44) | 71 (70.4, 71.4) | 34 (32, 38) | 76.8 (76.3, 77.2) | 27 (26, 30) | 76.9 (76.3, 77.3) | 34 (33, 37) |
| **Bibb** | 70 (69.3, 70.5) | 33 (30, 43) | 69.8 (69.1, 70.3) | 44 (38, 51) | 76.1 (75.6, 76.6) | 31 (29, 34) | 77.1 (76.5, 77.7) | 33 (31, 36) |
| **Blount** | 72.3 (71.7, 72.8) | 22 (19, 24) | 72.5 (71.9, 73) | 28 (25, 30) | 79 (78.4, 79.4) | 19 (17, 22) | 78.8 (78.2, 79.3) | 27 (25, 29) |
| **Bullock** | 67.3 (66.6, 67.7) | 51 (51, 51) | 68.4 (67.7, 68.9) | 51 (51, 51) | 75 (74.3, 75.5) | 37 (34, 42) | 75.3 (74.6, 75.8) | 43 (39, 47) |
| **Butler** | 68.9 (68.3, 69.2) | 47 (44, 51) | 69.6 (69, 70) | 47 (41, 51) | 76.8 (76.3, 77.2) | 27 (26, 30) | 77.2 (76.7, 77.6) | 33 (31, 35) |
| **Calhoun** | 70.2 (69.8, 70.5) | 32 (30, 36) | 70.5 (70.1, 70.8) | 37 (35, 39) | 77.1 (76.8, 77.4) | 26 (25, 28) | 77.3 (76.9, 77.7) | 33 (31, 34) |
| **Chambers** | 69.5 (69, 69.9) | 41 (35, 46) | 70.2 (69.7, 70.6) | 39 (36, 46) | 76.7 (76.3, 77.1) | 28 (26, 30) | 77.2 (76.6, 77.6) | 33 (32, 35) |
| **Cherokee** | 71.5 (70.8, 72) | 25 (23, 28) | 71.3 (70.5, 71.9) | 33 (30, 37) | 78.1 (77.5, 78.7) | 23 (21, 25) | 77.9 (77.2, 78.5) | 30 (28, 33) |
| **Chilton** | 70.7 (70.2, 71.1) | 28 (27, 32) | 70.4 (69.9, 70.8) | 37 (35, 43) | 77.7 (77.2, 78) | 24 (23, 26) | 77.3 (76.7, 77.7) | 33 (31, 35) |
| **Choctaw** | 69.2 (68.5, 69.6) | 45 (39, 49) | 70.4 (69.7, 70.9) | 37 (35, 45) | 77.1 (76.5, 77.5) | 26 (25, 29) | 77 (76.3, 77.4) | 34 (32, 37) |
| **Clarke** | 69.8 (69.2, 70.3) | 36 (31, 44) | 72.1 (71.5, 72.6) | 29 (27, 32) | 77.4 (76.9, 77.8) | 25 (24, 27) | 77.6 (77.1, 78.1) | 31 (30, 34) |
| **Clay** | 70.7 (70.1, 71.2) | 28 (26, 33) | 71.8 (71.2, 72.3) | 31 (29, 33) | 78.1 (77.5, 78.5) | 23 (21, 25) | 78 (77.3, 78.5) | 30 (28, 33) |
| **Cleburne** | 70.7 (70.1, 71.2) | 28 (26, 33) | 71.8 (71.2, 72.3) | 31 (29, 33) | 78.1 (77.5, 78.5) | 23 (21, 25) | 78 (77.3, 78.5) | 30 (28, 33) |
| **Coffee** | 72.4 (71.9, 72.8) | 21 (20, 23) | 73.4 (72.9, 73.8) | 23 (21, 26) | 78.6 (78.1, 79) | 21 (19, 23) | 79 (78.4, 79.4) | 26 (24, 29) |
| **Colbert** | 71.9 (71.4, 72.3) | 23 (22, 25) | 71.9 (71.3, 72.2) | 30 (29, 33) | 78.5 (78.1, 78.9) | 21 (20, 23) | 78.3 (77.8, 78.7) | 29 (27, 31) |
| **Conecuh** | 68.9 (68.3, 69.2) | 47 (44, 51) | 69.6 (69, 70) | 47 (41, 51) | 76.8 (76.3, 77.2) | 27 (26, 30) | 77.2 (76.7, 77.6) | 33 (31, 35) |
| **Coosa** | 70.7 (70.2, 71.1) | 28 (27, 32) | 70.4 (69.9, 70.8) | 37 (35, 43) | 77.7 (77.2, 78) | 24 (23, 26) | 77.3 (76.7, 77.7) | 33 (31, 35) |
| **Covington** | 70.8 (70.2, 71.2) | 28 (26, 32) | 71.4 (70.8, 71.8) | 32 (31, 35) | 78.5 (78, 78.9) | 21 (19, 23) | 77.9 (77.3, 78.3) | 30 (29, 33) |
| **Crenshaw** | 69 (68.4, 69.5) | 46 (42, 50) | 69.9 (69.2, 70.3) | 42 (38, 51) | 76 (75.4, 76.3) | 31 (30, 35) | 76.4 (75.8, 76.8) | 36 (35, 40) |
| **Cullman** | 72.2 (71.7, 72.6) | 22 (20, 24) | 71.9 (71.3, 72.2) | 30 (29, 33) | 78.6 (78.2, 79) | 21 (19, 22) | 78.4 (77.9, 78.8) | 29 (27, 30) |
| **Dale** | 72.3 (71.8, 72.6) | 22 (20, 24) | 72.9 (72.3, 73.2) | 26 (24, 29) | 78.6 (78.1, 79) | 21 (19, 23) | 79 (78.4, 79.4) | 26 (24, 29) |
| **Dallas** | 68.1 (67.5, 68.5) | 51 (49, 51) | 68.5 (67.9, 68.9) | 51 (51, 51) | 75.6 (75.1, 75.9) | 34 (32, 37) | 75.3 (74.7, 75.7) | 42 (40, 46) |
| **De Kalb** | 71.9 (71.3, 72.3) | 23 (22, 26) | 71.9 (71.3, 72.4) | 30 (28, 33) | 78.4 (77.8, 78.8) | 22 (20, 24) | 78.1 (77.5, 78.5) | 30 (28, 32) |
| **Elmore** | 72.5 (72, 72.8) | 21 (19, 23) | 73.1 (72.6, 73.5) | 25 (23, 27) | 78 (77.5, 78.4) | 23 (21, 25) | 78.8 (78.2, 79.2) | 27 (25, 29) |
| **Escambia** | 69.9 (69.4, 70.3) | 35 (31, 43) | 71.5 (70.9, 71.9) | 32 (30, 34) | 77.4 (76.9, 77.8) | 25 (24, 27) | 77.6 (77.1, 78.1) | 31 (30, 34) |
| **Etowah** | 70.6 (70.2, 70.9) | 29 (28, 32) | 70.5 (70, 70.8) | 37 (35, 41) | 77.5 (77.1, 77.8) | 25 (24, 26) | 77.2 (76.8, 77.6) | 33 (32, 34) |
| **Fayette** | 70.8 (70.1, 71.3) | 28 (26, 33) | 70.5 (69.8, 71) | 37 (34, 44) | 77.8 (77.2, 78.4) | 24 (22, 26) | 78 (77.3, 78.6) | 30 (28, 33) |
| **Franklin** | 70.4 (69.8, 70.9) | 30 (28, 37) | 70.7 (70, 71.3) | 36 (33, 41) | 78.2 (77.6, 78.7) | 22 (21, 24) | 77.1 (76.5, 77.7) | 33 (31, 36) |
| **Geneva** | 71.2 (70.6, 71.7) | 26 (24, 29) | 71.6 (70.8, 72.1) | 32 (29, 35) | 78.6 (78, 79.1) | 21 (19, 23) | 78.4 (77.8, 79) | 28 (26, 31) |
| **Greene** | 66.6 (65.8, 67.1) | 51 (51, 51) | 67.9 (67.2, 68.5) | 51 (51, 51) | 75.7 (75, 76.2) | 33 (30, 38) | 76.6 (75.9, 77.2) | 35 (33, 39) |
| **Hale** | 67.9 (67.3, 68.3) | 51 (51, 51) | 69.3 (68.6, 69.7) | 51 (45, 51) | 75.3 (74.8, 75.7) | 35 (33, 39) | 75.5 (74.9, 76) | 41 (38, 45) |
| **Henry** | 70.6 (69.9, 71) | 29 (27, 36) | 70.5 (69.8, 70.9) | 37 (34, 44) | 77.7 (77.1, 78.2) | 24 (22, 26) | 77.2 (76.5, 77.7) | 33 (31, 36) |
| **Houston** | 72.4 (72, 72.7) | 21 (20, 23) | 73.3 (72.8, 73.6) | 24 (22, 26) | 78.4 (78.1, 78.8) | 21 (20, 23) | 79.5 (79.1, 79.8) | 24 (22, 26) |
| **Jackson** | 71.7 (71.1, 72.2) | 24 (22, 27) | 71.2 (70.5, 71.7) | 33 (31, 37) | 78 (77.5, 78.4) | 23 (21, 25) | 77.6 (77, 78) | 32 (30, 34) |
| **Jefferson** | 70.5 (70.4, 70.7) | 30 (29, 31) | 71 (70.8, 71.1) | 34 (34, 35) | 77 (76.9, 77.2) | 27 (26, 27) | 77.8 (77.6, 77.9) | 31 (30, 31) |
| **Lamar** | 70.3 (69.6, 70.7) | 31 (28, 40) | 71.7 (71, 72.2) | 31 (29, 34) | 78.2 (77.6, 78.8) | 22 (20, 25) | 77.3 (76.6, 77.8) | 33 (31, 35) |
| **Lauderdale** | 72.9 (72.5, 73.2) | 19 (17, 21) | 73.3 (72.8, 73.6) | 24 (22, 26) | 79.4 (79, 79.8) | 17 (15, 19) | 79.7 (79.2, 80.1) | 23 (21, 25) |
| **Lawrence** | 70.8 (70.2, 71.1) | 28 (26, 32) | 71.5 (70.8, 71.9) | 32 (30, 35) | 78 (77.5, 78.4) | 23 (21, 25) | 77.7 (77.1, 78.1) | 31 (30, 33) |
| **Lee** | 72.3 (71.9, 72.7) | 21 (20, 23) | 73.9 (73.5, 74.2) | 21 (19, 23) | 78.6 (78.2, 79) | 21 (19, 22) | 78.6 (78.2, 78.9) | 28 (27, 29) |
| **Limestone** | 72.5 (72.1, 72.9) | 21 (19, 23) | 73 (72.6, 73.4) | 25 (23, 27) | 78.7 (78.3, 79.1) | 20 (19, 22) | 79 (78.5, 79.4) | 26 (24, 28) |
| **Lowndes** | 69 (68.4, 69.5) | 46 (42, 50) | 69.9 (69.2, 70.3) | 42 (38, 51) | 76 (75.4, 76.3) | 31 (30, 35) | 76.4 (75.8, 76.8) | 36 (35, 40) |
| **Macon** | 67.3 (66.6, 67.7) | 51 (51, 51) | 68.4 (67.7, 68.9) | 51 (51, 51) | 75 (74.3, 75.5) | 37 (34, 42) | 75.3 (74.6, 75.8) | 43 (39, 47) |
| **Madison** | 73.7 (73.4, 73.9) | 15 (14, 16) | 74.7 (74.5, 75) | 17 (16, 18) | 79.2 (78.9, 79.4) | 18 (17, 20) | 79.3 (79.1, 79.6) | 25 (23, 26) |
| **Marengo** | 68.9 (68.4, 69.3) | 46 (44, 51) | 69.9 (69.3, 70.3) | 42 (38, 51) | 76.1 (75.6, 76.4) | 31 (29, 34) | 76.3 (75.7, 76.6) | 37 (35, 40) |
| **Marion** | 70.4 (69.7, 70.9) | 30 (28, 38) | 71 (70.2, 71.5) | 34 (32, 39) | 78.4 (77.7, 78.9) | 22 (20, 24) | 77.7 (77, 78.3) | 31 (29, 34) |
| **Marshall** | 71.1 (70.6, 71.5) | 27 (25, 29) | 71.5 (71, 71.9) | 32 (30, 34) | 78.5 (78, 78.9) | 21 (20, 23) | 77.5 (77, 77.9) | 32 (30, 34) |
| **Mobile** | 70.2 (70, 70.4) | 32 (31, 34) | 70.7 (70.5, 70.9) | 36 (35, 37) | 76.9 (76.7, 77.1) | 27 (26, 28) | 77.8 (77.6, 78) | 31 (30, 32) |
| **Monroe** | 70.1 (69.4, 70.5) | 33 (30, 42) | 71.1 (70.5, 71.5) | 34 (32, 37) | 77.4 (76.9, 77.8) | 25 (24, 27) | 77.2 (76.6, 77.7) | 33 (31, 35) |
| **Montgomery** | 71.4 (71.1, 71.6) | 25 (24, 27) | 71.7 (71.4, 71.9) | 31 (30, 32) | 77.8 (77.5, 78) | 24 (23, 25) | 78 (77.7, 78.2) | 30 (29, 31) |
| **Morgan** | 72.9 (72.4, 73.2) | 19 (17, 21) | 72.9 (72.5, 73.3) | 26 (24, 28) | 78.8 (78.4, 79.1) | 20 (19, 22) | 78.9 (78.5, 79.3) | 26 (25, 28) |
| **Perry** | 67.9 (67.3, 68.3) | 51 (51, 51) | 69.3 (68.6, 69.7) | 51 (45, 51) | 75.3 (74.8, 75.7) | 35 (33, 39) | 75.5 (74.9, 76) | 41 (38, 45) |
| **Pickens** | 69.7 (69, 70.1) | 39 (33, 46) | 70.3 (69.7, 70.8) | 38 (35, 45) | 76.8 (76.3, 77.2) | 28 (26, 30) | 76.7 (76.1, 77.1) | 35 (33, 38) |
| **Pike** | 69.8 (69.2, 70.1) | 37 (32, 44) | 71.7 (71.1, 72.1) | 31 (29, 34) | 77.8 (77.3, 78.2) | 24 (22, 26) | 77.1 (76.5, 77.5) | 34 (32, 36) |
| **Randolph** | 70.2 (69.5, 70.6) | 32 (29, 42) | 70.8 (70, 71.3) | 35 (33, 40) | 77.4 (76.9, 77.9) | 25 (23, 27) | 77.4 (76.8, 77.9) | 32 (30, 35) |
| **Russell** | 68.5 (68.1, 68.8) | 49 (47, 51) | 69.3 (68.8, 69.6) | 51 (46, 51) | 75.7 (75.2, 76) | 33 (31, 36) | 76.3 (75.9, 76.7) | 36 (35, 39) |
| **Shelby** | 74.5 (74.1, 74.8) | 11 (10, 13) | 75.6 (75.2, 76) | 13 (11, 15) | 79.6 (79.2, 80) | 16 (14, 18) | 80 (79.6, 80.3) | 21 (19, 23) |
| **St. Clair** | 71.8 (71.3, 72.2) | 24 (22, 26) | 71.8 (71.3, 72.2) | 31 (29, 33) | 78.3 (77.8, 78.7) | 22 (20, 24) | 77.7 (77.2, 78.1) | 31 (30, 33) |
| **Sumter** | 66.6 (65.8, 67.1) | 51 (51, 51) | 67.9 (67.2, 68.5) | 51 (51, 51) | 75.7 (75, 76.2) | 33 (30, 38) | 76.6 (75.9, 77.2) | 35 (33, 39) |
| **Talladega** | 69.7 (69.3, 70) | 38 (34, 44) | 70.5 (70.1, 70.8) | 37 (35, 40) | 76.8 (76.4, 77.1) | 28 (26, 29) | 77.2 (76.8, 77.5) | 33 (32, 35) |
| **Tallapoosa** | 70.7 (70.2, 71.1) | 29 (27, 32) | 71.7 (71.2, 72.1) | 31 (29, 33) | 77.5 (77, 77.9) | 25 (24, 27) | 78.2 (77.6, 78.6) | 29 (28, 31) |
| **Tuscaloosa** | 72 (71.7, 72.2) | 23 (22, 24) | 71.9 (71.5, 72.1) | 30 (29, 32) | 78.5 (78.2, 78.8) | 21 (20, 22) | 77.8 (77.5, 78) | 31 (30, 32) |
| **Walker** | 69.2 (68.6, 69.6) | 45 (40, 48) | 68.3 (67.7, 68.7) | 51 (51, 51) | 77.1 (76.6, 77.4) | 27 (25, 28) | 75.9 (75.4, 76.3) | 39 (37, 42) |
| **Washington** | 70.8 (70.1, 71.2) | 28 (26, 33) | 72.9 (72.3, 73.4) | 26 (23, 29) | 77.8 (77.2, 78.3) | 24 (22, 26) | 78.5 (77.8, 79.1) | 28 (26, 31) |
| **Wilcox** | 68.9 (68.4, 69.3) | 46 (44, 51) | 69.9 (69.3, 70.3) | 42 (38, 51) | 76.1 (75.6, 76.4) | 31 (29, 34) | 76.3 (75.7, 76.6) | 37 (35, 40) |
| **Winston** | 70.5 (69.6, 71.1) | 30 (27, 39) | 70.9 (70.1, 71.5) | 35 (32, 40) | 78.2 (77.5, 78.7) | 22 (20, 25) | 77.6 (76.9, 78.2) | 31 (29, 34) |
| **ALASKA** |  |  |  |  |  |  |  |  |
| **All counties** | 74.5 (74.2, 74.7) | 11 (10, 13) | 75.9 (75.7, 76.2) | 12 (11, 13) | 79.6 (79.3, 79.8) | 16 (15, 18) | 80.5 (80.2, 80.8) | 18 (16, 20) |
| **ARIZONA** |  |  |  |  |  |  |  |  |
| **Apache** | 71.3 (70.7, 71.7) | 26 (24, 29) | 70.8 (70.3, 71.3) | 35 (33, 38) | 79.3 (78.7, 79.7) | 18 (16, 20) | 80 (79.4, 80.5) | 21 (18, 24) |
| **Cochise** | 74.6 (74.2, 75) | 11 (9, 13) | 75.8 (75.3, 76.2) | 12 (10, 14) | 79.4 (79, 79.8) | 17 (15, 19) | 81.1 (80.5, 81.5) | 15 (13, 18) |
| **Coconino** | 75.8 (75.3, 76.2) | 5 (4, 8) | 75.8 (75.3, 76.2) | 12 (10, 14) | 80.2 (79.8, 80.7) | 13 (10, 15) | 80.8 (80.3, 81.2) | 17 (14, 19) |
| **Gila** | 72.7 (72.1, 73.2) | 20 (17, 22) | 72.9 (72.3, 73.4) | 26 (23, 29) | 79.4 (78.8, 79.8) | 17 (15, 20) | 79.6 (79, 80.1) | 23 (20, 26) |
| **Graham** | 73.3 (72.8, 73.8) | 17 (15, 19) | 74.6 (74, 75.2) | 18 (15, 20) | 79.1 (78.5, 79.6) | 19 (16, 21) | 79.5 (78.7, 80.1) | 24 (21, 27) |
| **Greenlee** | 73.3 (72.8, 73.8) | 17 (15, 19) | 74.6 (74, 75.2) | 18 (15, 20) | 79.1 (78.5, 79.6) | 19 (16, 21) | 79.5 (78.7, 80.1) | 24 (21, 27) |
| **La Paz** | 73.1 (72.2, 73.6) | 18 (15, 22) | 73.9 (73, 74.6) | 21 (18, 25) | 80 (79.2, 80.6) | 14 (10, 18) | 81 (80, 81.7) | 15 (12, 21) |
| **Maricopa** | 75.1 (75, 75.2) | 8 (8, 9) | 76.8 (76.7, 76.9) | 8 (8, 9) | 80.5 (80.4, 80.6) | 11 (11, 12) | 81.6 (81.5, 81.7) | 12 (12, 13) |
| **Mohave** | 71.8 (71.4, 72.1) | 24 (23, 26) | 72 (71.6, 72.3) | 30 (29, 31) | 78.8 (78.4, 79.1) | 20 (19, 22) | 78.5 (78.1, 78.8) | 28 (27, 30) |
| **Navajo** | 72 (71.5, 72.3) | 23 (21, 25) | 71.4 (70.9, 71.7) | 32 (31, 34) | 78.5 (78.1, 78.8) | 21 (20, 23) | 79.5 (79, 79.9) | 24 (22, 26) |
| **Pima** | 74.3 (74.1, 74.5) | 12 (11, 13) | 75.8 (75.7, 76) | 12 (11, 13) | 80.4 (80.2, 80.6) | 12 (10, 13) | 81.7 (81.5, 81.9) | 12 (11, 13) |
| **Pinal** | 73.7 (73.3, 74) | 15 (13, 17) | 75.3 (74.9, 75.6) | 14 (13, 16) | 79.6 (79.2, 80) | 16 (14, 18) | 80.7 (80.3, 81.1) | 17 (15, 19) |
| **Santa Cruz** | 73.9 (73.2, 74.5) | 14 (11, 17) | 74.3 (73.5, 74.8) | 19 (17, 23) | 79.8 (79.1, 80.4) | 15 (12, 19) | 81 (80.2, 81.7) | 15 (12, 20) |
| **Yavapai** | 74.8 (74.4, 75.1) | 10 (8, 12) | 75.8 (75.4, 76.2) | 12 (11, 14) | 80.4 (80, 80.7) | 12 (10, 14) | 81.6 (81.2, 82) | 12 (10, 14) |
| **Yuma** | 76.2 (75.8, 76.7) | 3 (2, 5) | 78 (77.6, 78.5) | 4 (2, 6) | 82.1 (81.6, 82.6) | 3 (1, 5) | 83.8 (83.2, 84.3) | 2 (-2, 5) |
| **ARKANSAS** |  |  |  |  |  |  |  |  |
| **Arkansas** | 71.1 (70.4, 71.5) | 27 (25, 30) | 71.5 (70.8, 72) | 32 (30, 35) | 78.1 (77.6, 78.6) | 23 (21, 25) | 78.5 (77.8, 79) | 28 (26, 31) |
| **Ashley** | 70.6 (70, 71) | 29 (27, 34) | 72.4 (71.8, 72.9) | 28 (26, 31) | 77.4 (76.9, 77.8) | 25 (24, 27) | 77.4 (76.8, 77.9) | 32 (30, 35) |
| **Baxter** | 73.8 (73.1, 74.2) | 15 (13, 18) | 73.4 (72.7, 74) | 23 (21, 27) | 80 (79.5, 80.5) | 14 (11, 17) | 79.8 (79.2, 80.3) | 22 (19, 25) |
| **Benton** | 75.3 (74.8, 75.6) | 8 (6, 10) | 76.8 (76.4, 77.1) | 8 (7, 10) | 80.8 (80.4, 81.2) | 9 (7, 12) | 81.8 (81.4, 82.3) | 11 (9, 13) |
| **Boone** | 73.6 (73, 74.1) | 15 (13, 18) | 74.1 (73.5, 74.7) | 20 (17, 23) | 79.6 (79, 80.2) | 16 (13, 19) | 79.6 (78.9, 80.2) | 23 (20, 26) |
| **Bradley** | 70.6 (70, 71) | 29 (27, 34) | 72 (71.3, 72.4) | 30 (28, 33) | 77.4 (76.9, 77.8) | 25 (24, 27) | 78.3 (77.6, 78.7) | 29 (27, 32) |
| **Calhoun** | 69.9 (69.2, 70.4) | 35 (31, 45) | 70.8 (70.1, 71.3) | 35 (33, 40) | 76.6 (76, 77) | 28 (27, 31) | 77.4 (76.7, 77.9) | 32 (31, 35) |
| **Carroll** | 73.1 (72.3, 73.6) | 18 (15, 22) | 74.3 (73.5, 74.9) | 19 (17, 23) | 79.7 (79, 80.3) | 16 (12, 19) | 79.4 (78.6, 80) | 24 (21, 28) |
| **Chicot** | 68.8 (68.2, 69.2) | 47 (45, 51) | 69.4 (68.7, 69.8) | 50 (43, 51) | 76.4 (75.8, 76.8) | 29 (28, 32) | 75.8 (75.2, 76.2) | 39 (37, 43) |
| **Clark** | 72 (71.4, 72.4) | 23 (21, 25) | 72.9 (72.3, 73.3) | 26 (24, 29) | 78.2 (77.6, 78.6) | 22 (21, 24) | 78.5 (77.9, 79) | 28 (26, 31) |
| **Clay** | 70.8 (70, 71.4) | 28 (25, 34) | 70.3 (69.4, 71) | 38 (34, 50) | 78 (77.3, 78.5) | 23 (21, 26) | 78.1 (77.2, 78.8) | 30 (27, 33) |
| **Cleburne** | 73.5 (72.8, 74) | 16 (13, 19) | 74.4 (73.6, 74.9) | 19 (16, 22) | 79.9 (79.3, 80.5) | 15 (11, 18) | 79.5 (78.8, 80.1) | 24 (20, 27) |
| **Cleveland** | 70.6 (70, 71) | 29 (27, 34) | 72 (71.3, 72.4) | 30 (28, 33) | 77.4 (76.9, 77.8) | 25 (24, 27) | 78.3 (77.6, 78.7) | 29 (27, 32) |
| **Columbia** | 70.7 (70.1, 71.1) | 29 (27, 33) | 71.8 (71.1, 72.2) | 31 (29, 34) | 77.9 (77.3, 78.3) | 23 (22, 25) | 77.7 (77.1, 78.2) | 31 (29, 33) |
| **Conway** | 72.2 (71.5, 72.6) | 22 (20, 25) | 72.5 (71.8, 73) | 28 (25, 31) | 78.7 (78.1, 79.1) | 21 (19, 23) | 78.1 (77.5, 78.6) | 30 (28, 32) |
| **Craighead** | 72.4 (72, 72.8) | 21 (19, 23) | 72.3 (71.8, 72.7) | 29 (27, 31) | 78.7 (78.3, 79.1) | 20 (19, 22) | 78.5 (78, 78.9) | 28 (26, 30) |
| **Crawford** | 72 (71.5, 72.5) | 23 (21, 25) | 72.7 (72.2, 73.2) | 27 (24, 29) | 78.6 (78.1, 79.1) | 21 (19, 23) | 78.6 (78, 79.2) | 28 (25, 30) |
| **Crittenden** | 68.2 (67.7, 68.6) | 51 (48, 51) | 69.6 (69, 70) | 47 (41, 51) | 75.6 (75.1, 76) | 33 (31, 37) | 75.9 (75.3, 76.3) | 39 (37, 42) |
| **Cross** | 70.4 (69.7, 70.8) | 31 (28, 38) | 70.7 (70, 71.2) | 36 (33, 40) | 76.9 (76.4, 77.4) | 27 (25, 29) | 77.2 (76.6, 77.8) | 33 (31, 35) |
| **Dallas** | 69.9 (69.2, 70.4) | 35 (31, 45) | 70.8 (70.1, 71.3) | 35 (33, 40) | 76.6 (76, 77) | 28 (27, 31) | 77.4 (76.7, 77.9) | 32 (31, 35) |
| **Desha** | 68.8 (68.2, 69.2) | 47 (45, 51) | 69.4 (68.7, 69.8) | 50 (43, 51) | 76.4 (75.8, 76.8) | 29 (28, 32) | 75.8 (75.2, 76.2) | 39 (37, 43) |
| **Drew** | 71.2 (70.7, 71.6) | 26 (24, 28) | 71.8 (71.3, 72.2) | 31 (29, 33) | 77.7 (77.1, 78.1) | 24 (23, 26) | 77.4 (76.8, 77.8) | 32 (31, 35) |
| **Faulkner** | 74.3 (73.8, 74.7) | 12 (11, 15) | 73.5 (73.1, 73.9) | 23 (21, 25) | 79.6 (79.1, 80.1) | 16 (14, 18) | 79.8 (79.3, 80.2) | 22 (20, 25) |
| **Franklin** | 71.9 (71.2, 72.5) | 23 (21, 26) | 73.3 (72.5, 74) | 24 (21, 28) | 79.4 (78.6, 80) | 17 (14, 21) | 79.3 (78.5, 80) | 25 (21, 28) |
| **Fulton** | 72 (71.3, 72.6) | 23 (20, 26) | 72.5 (71.8, 73.2) | 28 (24, 31) | 79.1 (78.4, 79.6) | 19 (16, 22) | 78.7 (77.9, 79.3) | 27 (25, 31) |
| **Garland** | 72.9 (72.4, 73.2) | 19 (17, 21) | 72.5 (72, 72.8) | 28 (26, 30) | 79 (78.6, 79.4) | 19 (18, 21) | 79.6 (79.1, 79.9) | 23 (21, 26) |
| **Grant** | 73.3 (72.5, 73.8) | 17 (14, 21) | 73.7 (72.9, 74.2) | 22 (20, 26) | 78.8 (78.2, 79.4) | 20 (18, 22) | 79.6 (78.8, 80.2) | 23 (20, 27) |
| **Greene** | 71.4 (70.8, 71.9) | 25 (23, 28) | 72.7 (72, 73.2) | 27 (24, 30) | 78.7 (78.1, 79.2) | 21 (18, 23) | 78.5 (77.8, 79.1) | 28 (26, 31) |
| **Hempstead** | 70.7 (70.1, 71.1) | 28 (27, 33) | 71.5 (70.8, 71.9) | 32 (30, 35) | 77.8 (77.2, 78.3) | 24 (22, 26) | 77.2 (76.6, 77.7) | 33 (31, 35) |
| **Hot Spring** | 72.4 (71.8, 72.9) | 21 (19, 24) | 72.7 (72.1, 73.2) | 27 (24, 30) | 78.5 (77.9, 79) | 21 (19, 23) | 78.4 (77.7, 78.9) | 29 (27, 31) |
| **Howard** | 71.3 (70.7, 71.7) | 26 (24, 28) | 72 (71.4, 72.4) | 30 (28, 32) | 77.9 (77.3, 78.3) | 24 (22, 25) | 78 (77.4, 78.4) | 30 (28, 32) |
| **Independence** | 72.2 (71.5, 72.6) | 22 (20, 25) | 73.5 (72.9, 74.1) | 23 (20, 26) | 78.8 (78.3, 79.3) | 20 (18, 22) | 78.9 (78.3, 79.4) | 27 (24, 29) |
| **Izard** | 72 (71.3, 72.6) | 23 (20, 26) | 72.5 (71.8, 73.2) | 28 (24, 31) | 79.1 (78.4, 79.6) | 19 (16, 22) | 78.7 (77.9, 79.3) | 27 (25, 31) |
| **Jackson** | 70.7 (70.1, 71.3) | 28 (26, 33) | 70.6 (69.9, 71.1) | 36 (34, 43) | 77.4 (76.8, 77.9) | 25 (23, 28) | 76.9 (76.2, 77.4) | 34 (32, 37) |
| **Jefferson** | 69.7 (69.2, 70) | 39 (34, 44) | 70.4 (70, 70.8) | 37 (35, 41) | 76.8 (76.4, 77.1) | 28 (26, 29) | 77.2 (76.8, 77.6) | 33 (32, 35) |
| **Johnson** | 72.8 (72, 73.4) | 19 (16, 23) | 72.5 (71.8, 73.1) | 28 (25, 31) | 78.7 (78, 79.3) | 20 (18, 23) | 78.7 (77.9, 79.4) | 27 (24, 31) |
| **Lafayette** | 69.7 (69.1, 70.2) | 38 (32, 46) | 70.2 (69.5, 70.7) | 39 (36, 48) | 77.1 (76.5, 77.6) | 26 (25, 29) | 77.1 (76.5, 77.6) | 33 (32, 36) |
| **Lawrence** | 71 (70.2, 71.6) | 27 (24, 32) | 71.6 (70.6, 72.2) | 32 (29, 36) | 77.9 (77.3, 78.5) | 23 (21, 26) | 78.6 (77.8, 79.3) | 28 (25, 31) |
| **Lee** | 68.3 (67.6, 68.7) | 51 (48, 51) | 69.5 (68.8, 69.9) | 49 (42, 51) | 75.6 (75, 76) | 34 (31, 38) | 76.3 (75.6, 76.9) | 36 (34, 40) |
| **Lincoln** | 71.2 (70.7, 71.6) | 26 (24, 28) | 71.8 (71.3, 72.2) | 31 (29, 33) | 77.7 (77.1, 78.1) | 24 (23, 26) | 77.4 (76.8, 77.8) | 32 (31, 35) |
| **Little River** | 71.3 (70.7, 71.7) | 26 (24, 28) | 72 (71.4, 72.4) | 30 (28, 32) | 77.9 (77.3, 78.3) | 24 (22, 25) | 78 (77.4, 78.4) | 30 (28, 32) |
| **Logan** | 72.4 (71.6, 72.9) | 21 (19, 24) | 72.5 (71.6, 73.1) | 28 (25, 31) | 79.3 (78.5, 79.8) | 18 (15, 21) | 78.7 (77.9, 79.4) | 27 (24, 30) |
| **Lonoke** | 71.9 (71.4, 72.3) | 23 (22, 26) | 73.3 (72.8, 73.8) | 24 (21, 27) | 78.7 (78.2, 79.1) | 21 (19, 22) | 79 (78.4, 79.4) | 26 (24, 28) |
| **Madison** | 72 (71.1, 72.5) | 23 (21, 27) | 73 (72.2, 73.7) | 25 (22, 29) | 78.8 (78.1, 79.4) | 20 (17, 23) | 79.4 (78.4, 80.2) | 24 (20, 29) |
| **Marion** | 73.7 (72.8, 74.2) | 15 (13, 19) | 75.1 (74.1, 75.7) | 16 (13, 20) | 79.8 (79, 80.4) | 15 (12, 19) | 79.4 (78.6, 80.1) | 24 (21, 28) |
| **Miller** | 71.6 (71, 72) | 24 (23, 27) | 73.3 (72.7, 73.7) | 24 (22, 27) | 78.7 (78.1, 79.1) | 21 (19, 23) | 79 (78.4, 79.5) | 26 (24, 29) |
| **Mississippi** | 68.3 (67.8, 68.6) | 51 (48, 51) | 68.7 (68.2, 69.1) | 51 (51, 51) | 75.4 (75, 75.7) | 35 (33, 38) | 76.2 (75.6, 76.6) | 37 (36, 40) |
| **Monroe** | 68.3 (67.6, 68.7) | 51 (48, 51) | 69.5 (68.8, 69.9) | 49 (42, 51) | 75.6 (75, 76) | 34 (31, 38) | 76.3 (75.6, 76.9) | 36 (34, 40) |
| **Montgomery** | 71.9 (71.2, 72.5) | 23 (21, 26) | 73.2 (72.4, 73.7) | 25 (22, 28) | 79.2 (78.5, 79.8) | 18 (15, 21) | 78.7 (78, 79.3) | 27 (25, 30) |
| **Nevada** | 69.7 (69.1, 70.2) | 38 (32, 46) | 70.2 (69.5, 70.7) | 39 (36, 48) | 77.1 (76.5, 77.6) | 26 (25, 29) | 77.1 (76.5, 77.6) | 33 (32, 36) |
| **Newton** | 72 (71.2, 72.5) | 23 (21, 26) | 73.1 (72.2, 73.8) | 25 (21, 29) | 78.8 (78, 79.4) | 20 (17, 23) | 79.2 (78.3, 79.9) | 25 (22, 29) |
| **Ouachita** | 69.4 (68.9, 69.8) | 43 (37, 47) | 71.1 (70.5, 71.5) | 34 (32, 37) | 76.5 (76, 76.9) | 29 (27, 31) | 76.9 (76.4, 77.3) | 34 (32, 36) |
| **Perry** | 72.2 (71.5, 72.6) | 22 (20, 25) | 72.5 (71.8, 73) | 28 (25, 31) | 78.7 (78.1, 79.1) | 21 (19, 23) | 78.1 (77.5, 78.6) | 30 (28, 32) |
| **Phillips** | 65.9 (65.2, 66.4) | 51 (51, 51) | 66.8 (66, 67.3) | 51 (51, 51) | 74.1 (73.6, 74.4) | 43 (41, 45) | 75 (74.4, 75.4) | 44 (41, 48) |
| **Pike** | 72 (71.3, 72.5) | 23 (21, 26) | 73.1 (72.4, 73.6) | 25 (22, 28) | 78.8 (78.2, 79.3) | 20 (18, 22) | 79.3 (78.5, 79.9) | 25 (22, 28) |
| **Poinsett** | 69.6 (68.9, 70.1) | 39 (33, 46) | 69.3 (68.5, 69.8) | 51 (43, 51) | 77.4 (76.8, 77.9) | 25 (24, 28) | 76.6 (75.9, 77.2) | 35 (33, 39) |
| **Polk** | 72 (71.3, 72.5) | 23 (21, 26) | 73.1 (72.4, 73.6) | 25 (22, 28) | 78.8 (78.2, 79.3) | 20 (18, 22) | 79.3 (78.5, 79.9) | 25 (22, 28) |
| **Pope** | 73.3 (72.7, 73.7) | 17 (15, 20) | 75.1 (74.4, 75.6) | 16 (13, 19) | 79.9 (79.3, 80.4) | 14 (11, 18) | 79 (78.5, 79.5) | 26 (24, 28) |
| **Prairie** | 70.2 (69.5, 70.6) | 32 (29, 42) | 70.9 (70.2, 71.5) | 34 (32, 39) | 77.2 (76.6, 77.6) | 26 (25, 28) | 77.3 (76.6, 77.8) | 33 (31, 35) |
| **Pulaski** | 71.8 (71.6, 72) | 24 (23, 24) | 72.1 (71.9, 72.3) | 29 (29, 30) | 78.5 (78.2, 78.7) | 21 (20, 22) | 78.9 (78.7, 79.2) | 26 (25, 27) |
| **Randolph** | 71.9 (71.1, 72.5) | 23 (21, 27) | 72.6 (71.7, 73.2) | 27 (24, 31) | 79.2 (78.4, 79.8) | 18 (15, 21) | 79.1 (78.2, 79.7) | 26 (23, 29) |
| **Saline** | 74 (73.5, 74.4) | 14 (12, 16) | 74.7 (74.2, 75.2) | 17 (15, 20) | 79.3 (78.8, 79.7) | 18 (16, 20) | 79.8 (79.3, 80.2) | 22 (20, 25) |
| **Scott** | 71.9 (71.2, 72.5) | 23 (21, 26) | 73.2 (72.4, 73.7) | 25 (22, 28) | 79.2 (78.5, 79.8) | 18 (15, 21) | 78.7 (78, 79.3) | 27 (25, 30) |
| **Searcy** | 72 (71.2, 72.5) | 23 (21, 26) | 73.1 (72.2, 73.8) | 25 (21, 29) | 78.8 (78, 79.4) | 20 (17, 23) | 79.2 (78.3, 79.9) | 25 (22, 29) |
| **Sebastian** | 73 (72.6, 73.3) | 18 (17, 20) | 73.6 (73.1, 73.9) | 23 (21, 25) | 79.5 (79.1, 79.9) | 17 (15, 19) | 79.3 (78.9, 79.7) | 25 (23, 27) |
| **Sevier** | 71 (70.2, 71.6) | 27 (25, 32) | 71.8 (70.9, 72.4) | 31 (28, 35) | 77.8 (77.1, 78.4) | 24 (22, 26) | 77.9 (77.1, 78.6) | 30 (28, 33) |
| **Sharp** | 72.3 (71.5, 72.9) | 22 (19, 25) | 72.7 (71.8, 73.3) | 27 (24, 31) | 79.2 (78.5, 79.8) | 18 (15, 21) | 79.4 (78.5, 80.2) | 24 (20, 28) |
| **St. Francis** | 68.4 (67.8, 68.8) | 50 (47, 51) | 69.4 (68.7, 69.8) | 50 (44, 51) | 75.9 (75.3, 76.3) | 32 (30, 35) | 75.3 (74.7, 75.8) | 42 (40, 46) |
| **Stone** | 72.5 (71.8, 73.1) | 21 (18, 24) | 73.5 (72.7, 74.1) | 23 (20, 27) | 78.9 (78.3, 79.4) | 20 (18, 22) | 79.7 (79, 80.4) | 22 (19, 26) |
| **Union** | 70.6 (70.1, 71) | 29 (27, 32) | 71.2 (70.6, 71.6) | 33 (32, 36) | 77.8 (77.3, 78.1) | 24 (23, 25) | 77.6 (77.1, 78) | 31 (30, 33) |
| **Van Buren** | 72.5 (71.8, 73.1) | 21 (18, 24) | 73.5 (72.7, 74.1) | 23 (20, 27) | 78.9 (78.3, 79.4) | 20 (18, 22) | 79.7 (79, 80.4) | 22 (19, 26) |
| **Washington** | 73.7 (73.3, 74) | 15 (13, 17) | 74.9 (74.5, 75.3) | 16 (15, 18) | 79.4 (79, 79.7) | 17 (15, 19) | 80.1 (79.6, 80.5) | 20 (18, 23) |
| **White** | 71.9 (71.4, 72.4) | 23 (21, 25) | 73.2 (72.7, 73.7) | 24 (22, 27) | 79 (78.4, 79.4) | 19 (17, 21) | 78.8 (78.3, 79.3) | 27 (25, 29) |
| **Woodruff** | 70.2 (69.5, 70.6) | 32 (29, 42) | 70.9 (70.2, 71.5) | 34 (32, 39) | 77.2 (76.6, 77.6) | 26 (25, 28) | 77.3 (76.6, 77.8) | 33 (31, 35) |
| **Yell** | 71.4 (70.7, 72) | 25 (23, 29) | 71.6 (70.8, 72.2) | 31 (29, 35) | 79.1 (78.3, 79.6) | 19 (16, 22) | 78.3 (77.5, 78.9) | 29 (27, 32) |
| **CALIFORNIA** |  |  |  |  |  |  |  |  |
| **Alameda** | 75.9 (75.8, 76) | 5 (4, 5) | 77.7 (77.6, 77.8) | 5 (5, 6) | 80.5 (80.4, 80.7) | 11 (10, 12) | 82.3 (82.2, 82.4) | 9 (8, 10) |
| **Alpine** | 76.2 (75.7, 76.6) | 4 (2, 6) | 77 (76.4, 77.4) | 8 (6, 10) | 80.8 (80.2, 81.3) | 9 (7, 13) | 80.7 (80.1, 81.2) | 17 (14, 20) |
| **Amador** | 75.7 (75.1, 76.1) | 6 (4, 8) | 76.3 (75.7, 76.9) | 10 (8, 13) | 80.2 (79.6, 80.8) | 13 (9, 16) | 81.1 (80.4, 81.7) | 15 (12, 19) |
| **Butte** | 73.9 (73.6, 74.2) | 14 (13, 16) | 74 (73.7, 74.3) | 21 (19, 22) | 79.7 (79.3, 79.9) | 16 (14, 18) | 80 (79.6, 80.3) | 21 (19, 23) |
| **Calaveras** | 75.5 (74.9, 76) | 7 (5, 9) | 76.6 (76, 77.1) | 9 (7, 12) | 81 (80.4, 81.6) | 8 (5, 11) | 81.6 (80.9, 82.2) | 12 (9, 16) |
| **Colusa** | 73.2 (72.5, 73.8) | 17 (15, 21) | 74.2 (73.4, 74.8) | 20 (17, 23) | 79 (78.3, 79.6) | 19 (16, 22) | 80.3 (79.4, 81) | 19 (15, 24) |
| **Contra Costa** | 76.6 (76.5, 76.8) | 2 (1, 3) | 78.3 (78.1, 78.4) | 3 (2, 4) | 81 (80.8, 81.1) | 8 (8, 9) | 82.4 (82.2, 82.5) | 9 (8, 10) |
| **Del Norte** | 72.7 (72, 73.1) | 20 (18, 23) | 72.8 (72, 73.3) | 27 (24, 30) | 78.4 (77.8, 79) | 21 (19, 24) | 79.8 (79, 80.5) | 22 (18, 26) |
| **El Dorado** | 76.6 (76.2, 77) | 2 (1, 3) | 77.2 (76.8, 77.6) | 7 (6, 8) | 81.5 (81.1, 82) | 6 (3, 8) | 81.5 (81.1, 81.9) | 13 (11, 15) |
| **Fresno** | 74.6 (74.4, 74.8) | 11 (10, 12) | 75.2 (75, 75.3) | 15 (14, 16) | 79.8 (79.6, 80) | 15 (14, 16) | 80.2 (80, 80.4) | 20 (19, 21) |
| **Glenn** | 73 (72.4, 73.5) | 18 (16, 21) | 73.6 (72.9, 74.1) | 22 (20, 26) | 79.2 (78.5, 79.8) | 18 (15, 21) | 79.2 (78.5, 79.9) | 25 (22, 28) |
| **Humboldt** | 73.6 (73.2, 73.9) | 15 (14, 17) | 73.9 (73.5, 74.2) | 21 (20, 23) | 79.2 (78.8, 79.5) | 18 (17, 20) | 79.1 (78.7, 79.5) | 26 (24, 27) |
| **Imperial** | 73.2 (72.8, 73.6) | 17 (16, 19) | 75.1 (74.6, 75.4) | 16 (14, 18) | 79.8 (79.3, 80.2) | 15 (13, 18) | 81.7 (81.1, 82.2) | 12 (10, 15) |
| **Inyo** | 74.5 (73.8, 75) | 11 (9, 14) | 75.5 (74.7, 76) | 14 (11, 17) | 80.3 (79.6, 80.9) | 12 (9, 16) | 81.1 (80.3, 81.9) | 15 (11, 19) |
| **Kern** | 73.5 (73.3, 73.7) | 16 (15, 17) | 73.5 (73.3, 73.7) | 23 (22, 24) | 78.2 (78, 78.4) | 22 (22, 23) | 78.7 (78.5, 78.9) | 27 (26, 28) |
| **Kings** | 73.5 (73.1, 73.8) | 16 (15, 18) | 74.6 (74.2, 75) | 18 (16, 20) | 78.4 (77.9, 78.7) | 22 (20, 23) | 79.6 (79.1, 80.1) | 23 (20, 26) |
| **Lake** | 72.9 (72.3, 73.3) | 19 (17, 22) | 73.2 (72.7, 73.7) | 24 (22, 27) | 79 (78.6, 79.5) | 19 (17, 21) | 78.4 (77.8, 78.9) | 29 (27, 31) |
| **Lassen** | 74.5 (73.9, 74.9) | 11 (9, 14) | 75.5 (74.9, 76.1) | 13 (11, 16) | 79.1 (78.4, 79.6) | 19 (16, 21) | 79.8 (79, 80.4) | 22 (18, 26) |
| **Los Angeles** | 75.8 (75.8, 75.9) | 5 (5, 5) | 77.4 (77.4, 77.5) | 6 (6, 6) | 80.8 (80.7, 80.8) | 10 (9, 10) | 82.5 (82.4, 82.5) | 8 (8, 8) |
| **Madera** | 74 (73.6, 74.4) | 13 (12, 16) | 74.6 (74.1, 75) | 18 (16, 20) | 79.7 (79.2, 80.1) | 16 (14, 18) | 80.2 (79.7, 80.6) | 20 (17, 23) |
| **Marin** | 78.9 (78.6, 79.2) | -7 (-9, -6) | 80.8 (80.4, 81.1) | -13 (-16, -10) | 82.2 (81.9, 82.6) | 2 (1, 4) | 84.5 (84.1, 84.9) | -4 (-7, 0) |
| **Mariposa** | 75.3 (74.5, 75.8) | 7 (5, 11) | 76.3 (75.4, 76.9) | 10 (8, 14) | 80 (79.3, 80.6) | 14 (11, 18) | 81.1 (80.3, 81.9) | 15 (11, 19) |
| **Mendocino** | 74.7 (74.2, 75.1) | 10 (9, 13) | 75.6 (75, 76) | 13 (11, 16) | 79.7 (79.2, 80.1) | 16 (13, 18) | 80.8 (80.2, 81.3) | 16 (14, 20) |
| **Merced** | 73.6 (73.3, 73.9) | 15 (14, 17) | 75 (74.6, 75.3) | 16 (15, 18) | 79.2 (78.8, 79.5) | 18 (17, 20) | 79.9 (79.6, 80.3) | 21 (19, 23) |
| **Modoc** | 74.5 (73.9, 74.9) | 11 (9, 14) | 75.5 (74.9, 76.1) | 13 (11, 16) | 79.1 (78.4, 79.6) | 19 (16, 21) | 79.8 (79, 80.4) | 22 (18, 26) |
| **Mono** | 76.2 (75.7, 76.6) | 4 (2, 6) | 77 (76.4, 77.4) | 8 (6, 10) | 80.8 (80.2, 81.3) | 9 (7, 13) | 80.7 (80.1, 81.2) | 17 (14, 20) |
| **Monterey** | 76.9 (76.6, 77.1) | 1 (0, 2) | 78 (77.7, 78.2) | 4 (3, 5) | 80.8 (80.6, 81.1) | 9 (8, 11) | 82.6 (82.3, 82.9) | 8 (6, 9) |
| **Napa** | 75.6 (75.3, 75.9) | 6 (5, 8) | 77.2 (76.9, 77.6) | 7 (6, 8) | 81.1 (80.7, 81.5) | 8 (6, 10) | 81.9 (81.5, 82.3) | 11 (9, 13) |
| **Nevada** | 77.1 (76.6, 77.6) | 0 (-1, 2) | 78 (77.5, 78.4) | 4 (2, 6) | 81.5 (81, 81.9) | 6 (4, 8) | 81.8 (81.3, 82.1) | 11 (10, 14) |
| **Orange** | 77.6 (77.5, 77.7) | -1 (-2, -1) | 79.3 (79.2, 79.4) | -3 (-3, -2) | 81.7 (81.6, 81.8) | 5 (4, 5) | 83.6 (83.5, 83.8) | 3 (2, 3) |
| **Placer** | 77.1 (76.8, 77.4) | 0 (-1, 1) | 79 (78.7, 79.3) | -1 (-3, 1) | 81.2 (80.8, 81.5) | 7 (6, 9) | 82.8 (82.4, 83.1) | 7 (5, 8) |
| **Plumas** | 75.5 (74.9, 76) | 7 (4, 10) | 76.3 (75.6, 77) | 10 (8, 13) | 80.4 (79.8, 81.1) | 11 (8, 15) | 81.5 (80.7, 82.2) | 13 (9, 17) |
| **Riverside** | 74.9 (74.7, 75) | 9 (9, 10) | 75.8 (75.7, 75.9) | 12 (12, 13) | 80 (79.9, 80.2) | 14 (13, 15) | 81.3 (81.1, 81.4) | 14 (13, 15) |
| **Sacramento** | 75 (74.9, 75.1) | 9 (8, 10) | 75.6 (75.5, 75.7) | 13 (12, 14) | 79.8 (79.6, 79.9) | 15 (15, 16) | 81 (80.9, 81.2) | 15 (14, 16) |
| **San Benito** | 75.7 (75.2, 76.2) | 6 (4, 8) | 77.7 (77, 78.3) | 5 (3, 8) | 81 (80.3, 81.6) | 8 (5, 12) | 82 (81.2, 82.7) | 10 (7, 14) |
| **San Bernardino** | 73.8 (73.7, 73.9) | 15 (14, 15) | 74.3 (74.2, 74.4) | 19 (19, 20) | 78.6 (78.5, 78.7) | 21 (20, 21) | 79.5 (79.3, 79.6) | 24 (23, 25) |
| **San Diego** | 76.5 (76.4, 76.6) | 2 (2, 3) | 77.6 (77.5, 77.7) | 5 (5, 6) | 81 (80.9, 81.1) | 8 (8, 9) | 82.4 (82.3, 82.5) | 9 (8, 9) |
| **San Francisco** | 75.5 (75.4, 75.6) | 6 (6, 7) | 77.6 (77.4, 77.7) | 6 (5, 6) | 81.9 (81.7, 82) | 4 (3, 5) | 83.8 (83.6, 84) | 2 (1, 3) |
| **San Joaquin** | 74 (73.8, 74.1) | 14 (13, 15) | 74.9 (74.7, 75.1) | 16 (16, 17) | 79.2 (79, 79.4) | 18 (17, 19) | 80.1 (79.9, 80.3) | 20 (19, 22) |
| **San Luis Obispo** | 76.9 (76.6, 77.2) | 1 (0, 2) | 77.9 (77.6, 78.2) | 4 (3, 6) | 81.8 (81.4, 82.1) | 4 (3, 6) | 82.2 (81.9, 82.5) | 9 (8, 11) |
| **San Mateo** | 78.1 (77.9, 78.2) | -3 (-4, -3) | 79.8 (79.6, 80) | -6 (-7, -4) | 81.9 (81.8, 82.1) | 4 (3, 4) | 84.5 (84.3, 84.7) | -4 (-5, -2) |
| **Santa Barbara** | 77.2 (76.9, 77.4) | 0 (-1, 1) | 78.3 (78.1, 78.6) | 3 (1, 4) | 81.7 (81.5, 82) | 5 (3, 6) | 83.1 (82.8, 83.4) | 6 (4, 7) |
| **Santa Clara** | 78.6 (78.4, 78.7) | -5 (-6, -5) | 80.6 (80.4, 80.7) | -11 (-12, -10) | 82.2 (82, 82.3) | 3 (2, 3) | 83.9 (83.8, 84) | 1 (0, 2) |
| **Santa Cruz** | 76.9 (76.6, 77.2) | 1 (0, 2) | 78.1 (77.8, 78.4) | 4 (2, 5) | 81.3 (81, 81.7) | 6 (5, 8) | 82.6 (82.2, 82.9) | 8 (6, 9) |
| **Shasta** | 73.6 (73.2, 73.9) | 15 (14, 17) | 73.9 (73.5, 74.2) | 21 (20, 23) | 79.1 (78.7, 79.4) | 19 (17, 20) | 79.1 (78.7, 79.3) | 26 (25, 28) |
| **Sierra** | 75.5 (74.9, 76) | 7 (4, 10) | 76.3 (75.6, 77) | 10 (8, 13) | 80.4 (79.8, 81.1) | 11 (8, 15) | 81.5 (80.7, 82.2) | 13 (9, 17) |
| **Siskiyou** | 74.3 (73.8, 74.7) | 12 (10, 14) | 74.6 (74, 75.1) | 18 (16, 20) | 79.9 (79.4, 80.3) | 15 (12, 18) | 80.7 (80.1, 81.2) | 17 (14, 20) |
| **Solano** | 75.2 (75, 75.4) | 8 (7, 9) | 76.1 (75.9, 76.3) | 11 (10, 12) | 79.9 (79.7, 80.2) | 14 (13, 16) | 81 (80.7, 81.2) | 15 (14, 17) |
| **Sonoma** | 76.6 (76.4, 76.8) | 2 (1, 3) | 77.8 (77.5, 78) | 5 (4, 6) | 80.7 (80.5, 81) | 10 (9, 11) | 82.1 (81.9, 82.4) | 10 (9, 11) |
| **Stanislaus** | 73.8 (73.5, 74) | 14 (13, 16) | 74.8 (74.6, 75) | 17 (16, 18) | 78.7 (78.5, 79) | 20 (19, 21) | 79.4 (79.1, 79.7) | 24 (23, 25) |
| **Sutter** | 74.5 (74, 74.9) | 11 (10, 14) | 75 (74.5, 75.4) | 16 (14, 18) | 79.2 (78.7, 79.6) | 18 (16, 20) | 80.4 (79.9, 80.8) | 19 (16, 22) |
| **Tehama** | 73.3 (72.7, 73.7) | 17 (15, 20) | 74.1 (73.5, 74.6) | 20 (18, 23) | 79.3 (78.8, 79.8) | 18 (15, 20) | 79.1 (78.5, 79.5) | 26 (24, 28) |
| **Trinity** | 74.3 (73.8, 74.7) | 12 (10, 14) | 74.6 (74, 75.1) | 18 (16, 20) | 79.9 (79.4, 80.3) | 15 (12, 18) | 80.7 (80.1, 81.2) | 17 (14, 20) |
| **Tulare** | 73.3 (73, 73.5) | 17 (16, 18) | 74.1 (73.8, 74.3) | 20 (19, 22) | 78.8 (78.5, 79) | 20 (19, 21) | 79.4 (79.1, 79.7) | 24 (23, 26) |
| **Tuolumne** | 76.2 (75.7, 76.6) | 4 (2, 6) | 77 (76.4, 77.4) | 8 (6, 10) | 80.8 (80.2, 81.3) | 9 (7, 13) | 80.7 (80.1, 81.2) | 17 (14, 20) |
| **Ventura** | 77.2 (77, 77.4) | 0 (-1, 1) | 78.1 (77.9, 78.3) | 3 (3, 4) | 81.1 (80.9, 81.3) | 8 (7, 9) | 82.6 (82.4, 82.8) | 8 (7, 9) |
| **Yolo** | 75.5 (75.2, 75.9) | 6 (5, 8) | 76.9 (76.5, 77.2) | 8 (7, 9) | 79.7 (79.4, 80.1) | 16 (14, 17) | 81.5 (81.1, 81.9) | 13 (11, 15) |
| **Yuba** | 71.8 (71.3, 72.2) | 23 (22, 26) | 72.1 (71.6, 72.5) | 29 (28, 32) | 78.1 (77.6, 78.6) | 23 (21, 24) | 78.9 (78.3, 79.4) | 26 (24, 29) |
| **COLORADO** |  |  |  |  |  |  |  |  |
| **Adams** | 76.6 (76.4, 76.8) | 2 (2, 3) | 77.6 (77.4, 77.8) | 6 (5, 6) | 80.6 (80.4, 80.8) | 10 (9, 11) | 81.4 (81.2, 81.5) | 13 (13, 14) |
| **Alamosa** | 73.1 (72.3, 73.6) | 18 (15, 21) | 74.5 (73.7, 75.1) | 18 (15, 22) | 80.4 (79.7, 81) | 12 (8, 16) | 80.1 (79.3, 80.8) | 20 (16, 25) |
| **Arapahoe** | 76.9 (76.7, 77.2) | 1 (0, 2) | 78.4 (78.2, 78.7) | 2 (1, 3) | 80.8 (80.6, 81.1) | 9 (8, 11) | 82.3 (82, 82.6) | 9 (8, 10) |
| **Archuleta** | 74.1 (73.3, 74.7) | 13 (11, 17) | 76.8 (75.9, 77.4) | 9 (6, 12) | 80 (79.2, 80.6) | 14 (10, 18) | 81.4 (80.5, 82.2) | 13 (9, 18) |
| **Baca** | 72.9 (72.2, 73.5) | 19 (16, 22) | 74.2 (73.5, 74.8) | 19 (17, 23) | 80.1 (79.4, 80.7) | 14 (10, 18) | 79.3 (78.6, 80) | 25 (21, 28) |
| **Bent** | 72.9 (72.2, 73.5) | 19 (16, 22) | 74.2 (73.5, 74.8) | 19 (17, 23) | 80.1 (79.4, 80.7) | 14 (10, 18) | 79.3 (78.6, 80) | 25 (21, 28) |
| **Boulder** | 76.6 (76.4, 76.8) | 2 (2, 3) | 77.6 (77.4, 77.8) | 6 (5, 6) | 80.6 (80.4, 80.8) | 10 (9, 11) | 81.4 (81.2, 81.5) | 13 (13, 14) |
| **Broomfield** | 76.6 (76.4, 76.8) | 2 (2, 3) | 77.6 (77.4, 77.8) | 6 (5, 6) | 80.6 (80.4, 80.8) | 10 (9, 11) | 81.4 (81.2, 81.5) | 13 (13, 14) |
| **Chaffee** | 75.5 (74.8, 76.1) | 6 (4, 10) | 77.1 (76.3, 77.8) | 7 (5, 10) | 80.6 (79.9, 81.2) | 11 (7, 15) | 81.1 (80.3, 81.8) | 15 (11, 20) |
| **Cheyenne** | 74.2 (73.4, 74.7) | 13 (11, 16) | 74.4 (73.6, 74.9) | 19 (16, 22) | 79.8 (79.1, 80.3) | 15 (12, 19) | 80.6 (79.8, 81.3) | 17 (14, 22) |
| **Clear Creek** | 76.6 (75.9, 77.2) | 2 (0, 5) | 78.2 (77.3, 79) | 3 (-1, 7) | 80.9 (80.2, 81.7) | 9 (5, 13) | 82 (81.1, 82.9) | 11 (6, 15) |
| **Conejos** | 74.1 (73.3, 74.7) | 13 (11, 17) | 76.8 (75.9, 77.4) | 9 (6, 12) | 80 (79.2, 80.6) | 14 (10, 18) | 81.4 (80.5, 82.2) | 13 (9, 18) |
| **Costilla** | 73.1 (72.3, 73.6) | 18 (15, 21) | 74.5 (73.7, 75.1) | 18 (15, 22) | 80.4 (79.7, 81) | 12 (8, 16) | 80.1 (79.3, 80.8) | 20 (16, 25) |
| **Crowley** | 74 (73.6, 74.2) | 14 (12, 15) | 74.1 (73.7, 74.4) | 20 (19, 22) | 79.8 (79.3, 80.1) | 15 (14, 18) | 80 (79.5, 80.3) | 21 (19, 24) |
| **Custer** | 74 (73.5, 74.5) | 13 (11, 16) | 74.9 (74.3, 75.3) | 17 (14, 19) | 79.1 (78.5, 79.6) | 19 (16, 21) | 79.9 (79.3, 80.5) | 21 (18, 25) |
| **Delta** | 74.5 (73.8, 75) | 11 (9, 14) | 75.9 (75.1, 76.4) | 12 (10, 15) | 80.2 (79.5, 80.8) | 13 (10, 17) | 81.5 (80.7, 82.2) | 13 (10, 17) |
| **Denver** | 73.1 (72.9, 73.3) | 18 (17, 19) | 74.4 (74.2, 74.6) | 19 (18, 20) | 79.1 (78.9, 79.3) | 19 (18, 20) | 80.9 (80.7, 81.2) | 16 (14, 17) |
| **Dolores** | 74.7 (74, 75.2) | 11 (8, 14) | 75.6 (74.9, 76.2) | 13 (10, 16) | 79.7 (79.1, 80.4) | 16 (12, 19) | 80.9 (80.1, 81.7) | 16 (12, 20) |
| **Douglas** | 78.6 (78.1, 79) | -6 (-8, -3) | 80.3 (79.8, 80.8) | -9 (-13, -5) | 81.7 (81.1, 82.2) | 5 (2, 8) | 83.5 (82.9, 84.1) | 3 (0, 6) |
| **Eagle** | 77.6 (76.9, 78.2) | -1 (-4, 1) | 79.1 (78.3, 79.9) | -1 (-7, 3) | 81.2 (80.4, 82) | 7 (3, 11) | 83.1 (82.1, 84) | 5 (0, 10) |
| **El Paso** | 76.2 (75.9, 76.4) | 4 (3, 5) | 76.6 (76.4, 76.9) | 9 (8, 10) | 80.4 (80.1, 80.6) | 12 (10, 14) | 81.1 (80.8, 81.4) | 15 (13, 16) |
| **Elbert** | 76.5 (75.6, 77) | 3 (1, 6) | 77.9 (77.1, 78.6) | 4 (1, 7) | 81 (80.1, 81.7) | 8 (5, 13) | 81.9 (80.9, 82.7) | 11 (7, 16) |
| **Fremont** | 74 (73.5, 74.5) | 13 (11, 16) | 74.9 (74.3, 75.3) | 17 (14, 19) | 79.1 (78.5, 79.6) | 19 (16, 21) | 79.9 (79.3, 80.5) | 21 (18, 25) |
| **Garfield** | 75.8 (75.1, 76.3) | 5 (3, 8) | 76.7 (76, 77.2) | 9 (7, 11) | 80.5 (79.8, 81.1) | 11 (8, 15) | 81.5 (80.8, 82.1) | 13 (10, 16) |
| **Gilpin** | 76.8 (75.9, 77.5) | 1 (-1, 5) | 77.1 (76.1, 77.9) | 7 (4, 11) | 81.2 (80.3, 82.1) | 7 (3, 12) | 81.8 (80.7, 82.9) | 11 (6, 17) |
| **Grand** | 76.6 (75.9, 77.2) | 2 (0, 5) | 78.2 (77.3, 79) | 3 (-1, 7) | 80.9 (80.2, 81.7) | 9 (5, 13) | 82 (81.1, 82.9) | 11 (6, 15) |
| **Gunnison** | 78.2 (77.5, 78.8) | -4 (-7, -1) | 80 (79, 80.8) | -7 (-13, -1) | 82 (81.2, 82.8) | 3 (0, 7) | 84.2 (83.1, 85.2) | -1 (-9, 6) |
| **Hinsdale** | 74.8 (73.9, 75.4) | 10 (7, 14) | 75.7 (74.9, 76.4) | 12 (10, 16) | 80.7 (80, 81.5) | 10 (6, 14) | 81.2 (80.4, 82) | 14 (10, 19) |
| **Huerfano** | 73.1 (72.3, 73.6) | 18 (15, 21) | 74.5 (73.7, 75.1) | 18 (15, 22) | 80.4 (79.7, 81) | 12 (8, 16) | 80.1 (79.3, 80.8) | 20 (16, 25) |
| **Jackson** | 76.6 (75.8, 77.2) | 2 (0, 5) | 78.6 (77.7, 79.4) | 1 (-3, 5) | 81.2 (80.4, 81.9) | 7 (4, 12) | 83.3 (82.2, 84.3) | 5 (-2, 9) |
| **Jefferson** | 76.6 (76.4, 76.8) | 2 (2, 3) | 77.6 (77.4, 77.8) | 6 (5, 6) | 80.6 (80.4, 80.8) | 10 (9, 11) | 81.4 (81.2, 81.5) | 13 (13, 14) |
| **Kiowa** | 74.2 (73.4, 74.7) | 13 (11, 16) | 74.4 (73.6, 74.9) | 19 (16, 22) | 79.8 (79.1, 80.3) | 15 (12, 19) | 80.6 (79.8, 81.3) | 17 (14, 22) |
| **Kit Carson** | 74 (73.2, 74.5) | 14 (11, 17) | 75.2 (74.4, 75.8) | 15 (12, 19) | 80.6 (79.8, 81.2) | 11 (7, 15) | 79.9 (79.1, 80.6) | 21 (18, 26) |
| **La Plata** | 76.4 (75.8, 77) | 3 (1, 5) | 77.9 (77.2, 78.4) | 5 (2, 7) | 81.1 (80.4, 81.7) | 8 (5, 11) | 82.2 (81.4, 82.9) | 9 (6, 13) |
| **Lake** | 75.7 (74.9, 76.3) | 6 (3, 9) | 77.1 (76.3, 77.8) | 7 (5, 10) | 80.5 (79.8, 81.3) | 11 (7, 15) | 81.6 (80.7, 82.5) | 12 (8, 17) |
| **Larimer** | 77.8 (77.4, 78.1) | -2 (-3, -1) | 79.2 (78.8, 79.5) | -2 (-4, 0) | 81.5 (81.1, 81.8) | 6 (4, 8) | 82.7 (82.3, 83.1) | 7 (6, 9) |
| **Las Animas** | 73.3 (72.5, 73.8) | 17 (14, 21) | 75 (74.2, 75.6) | 16 (13, 20) | 80.3 (79.5, 80.9) | 12 (9, 17) | 80.5 (79.6, 81.2) | 18 (14, 23) |
| **Lincoln** | 74.2 (73.4, 74.7) | 13 (11, 16) | 74.4 (73.6, 74.9) | 19 (16, 22) | 79.8 (79.1, 80.3) | 15 (12, 19) | 80.6 (79.8, 81.3) | 17 (14, 22) |
| **Logan** | 74.8 (74.1, 75.3) | 10 (7, 13) | 75.8 (75.1, 76.3) | 12 (10, 16) | 80.3 (79.6, 80.9) | 12 (9, 16) | 80.2 (79.4, 80.9) | 20 (16, 24) |
| **Mesa** | 75 (74.5, 75.4) | 9 (7, 11) | 75.9 (75.4, 76.3) | 12 (10, 14) | 80.6 (80.2, 81.1) | 10 (8, 13) | 81.2 (80.8, 81.6) | 14 (12, 17) |
| **Mineral** | 74.8 (73.9, 75.4) | 10 (7, 14) | 75.7 (74.9, 76.4) | 12 (10, 16) | 80.7 (80, 81.5) | 10 (6, 14) | 81.2 (80.4, 82) | 14 (10, 19) |
| **Moffat** | 74.6 (73.7, 75.1) | 11 (8, 15) | 74.8 (74, 75.4) | 17 (14, 21) | 79.8 (79, 80.4) | 15 (12, 19) | 80.3 (79.4, 81) | 19 (15, 24) |
| **Montezuma** | 74.7 (74, 75.2) | 11 (8, 14) | 75.6 (74.9, 76.2) | 13 (10, 16) | 79.7 (79.1, 80.4) | 16 (12, 19) | 80.9 (80.1, 81.7) | 16 (12, 20) |
| **Montrose** | 74.7 (74, 75.2) | 10 (8, 13) | 75.3 (74.6, 75.8) | 14 (12, 18) | 80.2 (79.6, 80.7) | 13 (10, 16) | 81 (80.4, 81.6) | 15 (12, 19) |
| **Morgan** | 74 (73.3, 74.6) | 13 (11, 17) | 75 (74.2, 75.6) | 16 (13, 19) | 79.8 (79.1, 80.4) | 15 (12, 19) | 80.7 (79.9, 81.4) | 17 (13, 22) |
| **Otero** | 74.2 (73.4, 74.7) | 13 (11, 16) | 74.4 (73.6, 74.9) | 19 (16, 22) | 79.8 (79.1, 80.3) | 15 (12, 19) | 80.6 (79.8, 81.3) | 17 (14, 22) |
| **Ouray** | 74.8 (73.9, 75.4) | 10 (7, 14) | 75.7 (74.9, 76.4) | 12 (10, 16) | 80.7 (80, 81.5) | 10 (6, 14) | 81.2 (80.4, 82) | 14 (10, 19) |
| **Park** | 75.7 (74.9, 76.3) | 6 (3, 9) | 77.1 (76.3, 77.8) | 7 (5, 10) | 80.5 (79.8, 81.3) | 11 (7, 15) | 81.6 (80.7, 82.5) | 12 (8, 17) |
| **Phillips** | 74.8 (74.1, 75.3) | 10 (7, 13) | 75.8 (75.1, 76.3) | 12 (10, 16) | 80.3 (79.6, 80.9) | 12 (9, 16) | 80.2 (79.4, 80.9) | 20 (16, 24) |
| **Pitkin** | 78.2 (77.5, 78.8) | -4 (-7, -1) | 80 (79, 80.8) | -7 (-13, -1) | 82 (81.2, 82.8) | 3 (0, 7) | 84.2 (83.1, 85.2) | -1 (-9, 6) |
| **Prowers** | 72.9 (72.2, 73.5) | 19 (16, 22) | 74.2 (73.5, 74.8) | 19 (17, 23) | 80.1 (79.4, 80.7) | 14 (10, 18) | 79.3 (78.6, 80) | 25 (21, 28) |
| **Pueblo** | 74 (73.6, 74.2) | 14 (12, 15) | 74.1 (73.7, 74.4) | 20 (19, 22) | 79.8 (79.3, 80.1) | 15 (14, 18) | 80 (79.5, 80.3) | 21 (19, 24) |
| **Rio Blanco** | 74.6 (73.7, 75.1) | 11 (8, 15) | 74.8 (74, 75.4) | 17 (14, 21) | 79.8 (79, 80.4) | 15 (12, 19) | 80.3 (79.4, 81) | 19 (15, 24) |
| **Rio Grande** | 74.8 (73.9, 75.4) | 10 (7, 14) | 75.7 (74.9, 76.4) | 12 (10, 16) | 80.7 (80, 81.5) | 10 (6, 14) | 81.2 (80.4, 82) | 14 (10, 19) |
| **Routt** | 76.6 (75.8, 77.2) | 2 (0, 5) | 78.6 (77.7, 79.4) | 1 (-3, 5) | 81.2 (80.4, 81.9) | 7 (4, 12) | 83.3 (82.2, 84.3) | 5 (-2, 9) |
| **Saguache** | 74 (73.5, 74.5) | 13 (11, 16) | 74.9 (74.3, 75.3) | 17 (14, 19) | 79.1 (78.5, 79.6) | 19 (16, 21) | 79.9 (79.3, 80.5) | 21 (18, 25) |
| **San Juan** | 74.7 (74, 75.2) | 11 (8, 14) | 75.6 (74.9, 76.2) | 13 (10, 16) | 79.7 (79.1, 80.4) | 16 (12, 19) | 80.9 (80.1, 81.7) | 16 (12, 20) |
| **San Miguel** | 74.7 (74, 75.2) | 11 (8, 14) | 75.6 (74.9, 76.2) | 13 (10, 16) | 79.7 (79.1, 80.4) | 16 (12, 19) | 80.9 (80.1, 81.7) | 16 (12, 20) |
| **Sedgwick** | 74.8 (74.1, 75.3) | 10 (7, 13) | 75.8 (75.1, 76.3) | 12 (10, 16) | 80.3 (79.6, 80.9) | 12 (9, 16) | 80.2 (79.4, 80.9) | 20 (16, 24) |
| **Summit** | 77.3 (76.5, 78) | 0 (-3, 3) | 79.3 (78.3, 80.2) | -2 (-9, 3) | 81.6 (80.7, 82.4) | 5 (1, 10) | 83 (81.9, 84.2) | 6 (-1, 11) |
| **Teller** | 76.6 (75.8, 77.2) | 2 (0, 5) | 77.9 (77.1, 78.6) | 4 (1, 7) | 81.4 (80.6, 82.2) | 6 (2, 11) | 81.8 (80.9, 82.7) | 11 (7, 16) |
| **Washington** | 74 (73.3, 74.6) | 13 (11, 17) | 75 (74.2, 75.6) | 16 (13, 19) | 79.8 (79.1, 80.4) | 15 (12, 19) | 80.7 (79.9, 81.4) | 17 (13, 22) |
| **Weld** | 76.6 (76.4, 76.8) | 2 (2, 3) | 77.6 (77.4, 77.8) | 6 (5, 6) | 80.6 (80.4, 80.8) | 10 (9, 11) | 81.4 (81.2, 81.5) | 13 (13, 14) |
| **Yuma** | 74 (73.2, 74.5) | 14 (11, 17) | 75.2 (74.4, 75.8) | 15 (12, 19) | 80.6 (79.8, 81.2) | 11 (7, 15) | 79.9 (79.1, 80.6) | 21 (18, 26) |
| **CONNECTICUT** |  |  |  |  |  |  |  |  |
| **Fairfield** | 76.8 (76.7, 77) | 1 (1, 2) | 78.9 (78.7, 79) | 0 (-1, 1) | 81.6 (81.4, 81.8) | 5 (4, 6) | 83.3 (83.1, 83.4) | 5 (4, 6) |
| **Hartford** | 75.3 (75.1, 75.4) | 8 (7, 8) | 76.6 (76.4, 76.7) | 9 (9, 10) | 80.2 (80.1, 80.4) | 13 (12, 14) | 81.7 (81.5, 81.9) | 12 (11, 13) |
| **Litchfield** | 76.1 (75.8, 76.4) | 4 (3, 5) | 77.3 (76.9, 77.7) | 6 (5, 8) | 81.4 (81, 81.7) | 6 (5, 8) | 82.5 (82.1, 82.9) | 8 (6, 10) |
| **Middlesex** | 76.1 (75.8, 76.4) | 4 (3, 5) | 77.8 (77.4, 78.1) | 5 (3, 6) | 80.7 (80.4, 81) | 10 (8, 12) | 82.5 (82.1, 82.9) | 8 (6, 10) |
| **New Haven** | 75.2 (75.1, 75.4) | 8 (7, 8) | 76.3 (76.1, 76.4) | 10 (10, 11) | 80.4 (80.2, 80.5) | 12 (11, 13) | 81.3 (81.1, 81.4) | 14 (13, 15) |
| **New London** | 76.1 (75.7, 76.3) | 4 (3, 5) | 76.4 (76.1, 76.7) | 10 (9, 11) | 81.2 (80.9, 81.6) | 7 (5, 9) | 81.5 (81.1, 81.8) | 13 (11, 15) |
| **Tolland** | 76.4 (76, 76.8) | 3 (1, 4) | 78.3 (77.9, 78.7) | 3 (1, 5) | 80.7 (80.2, 81.1) | 10 (8, 13) | 82.5 (82, 83) | 8 (6, 10) |
| **Windham** | 74.3 (73.8, 74.6) | 12 (11, 14) | 75.4 (74.9, 75.7) | 14 (13, 16) | 80.2 (79.7, 80.5) | 13 (11, 16) | 80.7 (80.2, 81.1) | 17 (15, 20) |
| **DELAWARE** |  |  |  |  |  |  |  |  |
| **Kent** | 73.2 (72.8, 73.5) | 17 (16, 19) | 73.5 (73.2, 73.8) | 23 (21, 24) | 78.6 (78.2, 78.9) | 21 (20, 22) | 79.5 (79.1, 79.8) | 24 (22, 26) |
| **New Castle** | 74 (73.8, 74.2) | 14 (13, 14) | 75.2 (75, 75.4) | 15 (14, 16) | 79.2 (79, 79.4) | 18 (17, 19) | 80.1 (79.9, 80.3) | 20 (19, 22) |
| **Sussex** | 73.6 (73.3, 73.9) | 15 (14, 17) | 74.8 (74.5, 75.1) | 17 (15, 18) | 79.2 (78.9, 79.5) | 18 (17, 20) | 80.8 (80.5, 81.2) | 16 (14, 18) |
| **D. Of COLUMBIA** |  |  |  |  |  |  |  |  |
| **D. Of Columbia** | 68.5 (68.4, 68.7) | 49 (48, 50) | 71.6 (71.4, 71.8) | 31 (31, 32) | 76.4 (76.2, 76.6) | 29 (28, 30) | 78.5 (78.3, 78.7) | 28 (27, 29) |
| **FLORIDA** |  |  |  |  |  |  |  |  |
| **Alachua** | 73.9 (73.6, 74.1) | 14 (13, 16) | 75.1 (74.8, 75.4) | 15 (14, 17) | 79.6 (79.2, 79.9) | 16 (15, 18) | 79.8 (79.4, 80) | 22 (21, 24) |
| **Baker** | 68 (67.4, 68.4) | 51 (50, 51) | 68.1 (67.5, 68.5) | 51 (51, 51) | 77.1 (76.5, 77.6) | 26 (25, 29) | 77.7 (77, 78.3) | 31 (29, 34) |
| **Bay** | 73.1 (72.8, 73.4) | 18 (16, 19) | 73.7 (73.4, 74) | 22 (20, 23) | 78.6 (78.2, 78.9) | 21 (20, 22) | 79.2 (78.9, 79.6) | 25 (23, 27) |
| **Bradford** | 71.2 (70.6, 71.7) | 26 (24, 29) | 71.8 (71.2, 72.3) | 31 (29, 33) | 77.7 (77.1, 78.2) | 24 (22, 27) | 78.9 (78.2, 79.5) | 27 (24, 29) |
| **Brevard** | 75.2 (75, 75.5) | 8 (7, 9) | 75.9 (75.7, 76.1) | 12 (11, 13) | 80.8 (80.6, 81) | 9 (8, 10) | 81.2 (81, 81.4) | 14 (13, 15) |
| **Broward** | 75.4 (75.3, 75.5) | 7 (6, 8) | 76.5 (76.3, 76.6) | 10 (9, 10) | 80.9 (80.8, 81.1) | 9 (8, 9) | 82 (81.8, 82.1) | 10 (10, 11) |
| **Calhoun** | 71.1 (70.5, 71.5) | 27 (25, 30) | 72 (71.4, 72.5) | 30 (28, 32) | 77.7 (77.1, 78.1) | 24 (23, 26) | 77.7 (77.1, 78.2) | 31 (29, 33) |
| **Charlotte** | 76.2 (75.8, 76.5) | 3 (2, 5) | 76.2 (75.8, 76.5) | 11 (9, 12) | 82 (81.6, 82.3) | 3 (2, 5) | 83.1 (82.7, 83.5) | 5 (4, 7) |
| **Citrus** | 74.1 (73.7, 74.5) | 13 (11, 15) | 73.1 (72.7, 73.5) | 25 (23, 27) | 80.2 (79.8, 80.5) | 13 (11, 15) | 80.8 (80.4, 81.2) | 16 (14, 19) |
| **Clay** | 73.7 (73.3, 74) | 15 (13, 17) | 74.8 (74.4, 75.1) | 17 (15, 19) | 78.8 (78.4, 79.2) | 20 (18, 21) | 79.1 (78.7, 79.4) | 26 (24, 27) |
| **Collier** | 78.1 (77.8, 78.4) | -3 (-5, -2) | 80.2 (79.8, 80.5) | -8 (-11, -6) | 82.9 (82.5, 83.2) | -1 (-2, 1) | 86 (85.6, 86.3) | -16 (-19, -12) |
| **Columbia** | 71.6 (71.1, 72) | 24 (23, 26) | 71.8 (71.3, 72.2) | 31 (29, 33) | 77.7 (77.2, 78.1) | 24 (23, 26) | 77.8 (77.2, 78.2) | 31 (29, 33) |
| **De Soto** | 73 (72.3, 73.3) | 19 (17, 21) | 74.2 (73.5, 74.6) | 20 (18, 23) | 79.2 (78.6, 79.6) | 18 (16, 21) | 80.1 (79.4, 80.6) | 21 (17, 25) |
| **Dixie** | 71.9 (71.2, 72.4) | 23 (21, 26) | 71.3 (70.7, 71.9) | 33 (30, 36) | 78.3 (77.6, 78.9) | 22 (20, 25) | 78.8 (78, 79.5) | 27 (24, 30) |
| **Duval** | 71.8 (71.7, 72) | 24 (23, 24) | 72.5 (72.4, 72.7) | 28 (27, 28) | 77.5 (77.3, 77.6) | 25 (25, 26) | 78 (77.9, 78.2) | 30 (29, 31) |
| **Escambia** | 72.6 (72.3, 72.8) | 20 (19, 21) | 73.6 (73.3, 73.8) | 22 (21, 24) | 78.5 (78.2, 78.7) | 21 (20, 22) | 79 (78.7, 79.2) | 26 (25, 27) |
| **Flagler** | 75.3 (74.7, 75.7) | 7 (6, 10) | 76.7 (76.2, 77.1) | 9 (7, 11) | 81 (80.5, 81.4) | 8 (6, 11) | 83.1 (82.5, 83.5) | 6 (3, 8) |
| **Franklin** | 72.2 (71.5, 72.7) | 22 (20, 25) | 72.4 (71.7, 73) | 28 (25, 31) | 77.4 (76.8, 77.9) | 25 (24, 28) | 78.5 (77.7, 79.2) | 28 (25, 31) |
| **Gadsden** | 69.2 (68.7, 69.6) | 45 (40, 48) | 70.1 (69.6, 70.5) | 40 (37, 48) | 76.6 (76.1, 77.1) | 28 (26, 31) | 77.9 (77.3, 78.3) | 31 (29, 33) |
| **Gilchrist** | 71.9 (71.2, 72.4) | 23 (21, 26) | 71.3 (70.7, 71.9) | 33 (30, 36) | 78.3 (77.6, 78.9) | 22 (20, 25) | 78.8 (78, 79.5) | 27 (24, 30) |
| **Glades** | 73 (72.3, 73.3) | 19 (17, 21) | 74.2 (73.5, 74.6) | 20 (18, 23) | 79.2 (78.6, 79.6) | 18 (16, 21) | 80.1 (79.4, 80.6) | 21 (17, 25) |
| **Gulf** | 71.1 (70.5, 71.5) | 27 (25, 30) | 72 (71.4, 72.5) | 30 (28, 32) | 77.7 (77.1, 78.1) | 24 (23, 26) | 77.7 (77.1, 78.2) | 31 (29, 33) |
| **Hamilton** | 70.3 (69.7, 70.6) | 31 (29, 37) | 70.2 (69.7, 70.6) | 39 (36, 45) | 76.3 (75.8, 76.7) | 30 (28, 33) | 76.5 (75.9, 76.9) | 36 (34, 39) |
| **Hardee** | 71.7 (71, 72.2) | 24 (22, 27) | 73.2 (72.4, 73.8) | 24 (22, 28) | 78.5 (77.8, 79.1) | 21 (19, 24) | 78.9 (78.1, 79.5) | 27 (24, 30) |
| **Hendry** | 71.3 (70.7, 71.7) | 26 (24, 29) | 71.8 (71.1, 72.3) | 31 (29, 33) | 77.1 (76.5, 77.6) | 26 (25, 29) | 77.8 (77.1, 78.4) | 31 (29, 33) |
| **Hernando** | 74.5 (74, 74.8) | 11 (10, 13) | 74.3 (73.9, 74.5) | 19 (18, 21) | 80.2 (79.9, 80.5) | 13 (11, 15) | 81.1 (80.7, 81.4) | 15 (13, 17) |
| **Highlands** | 74.1 (73.6, 74.5) | 13 (11, 15) | 75.5 (75, 75.9) | 14 (12, 16) | 80.9 (80.4, 81.2) | 9 (7, 12) | 81.6 (81, 82) | 12 (10, 15) |
| **Hillsborough** | 73.2 (73.1, 73.4) | 17 (16, 18) | 74.7 (74.5, 74.8) | 17 (17, 18) | 78.9 (78.8, 79.1) | 19 (19, 20) | 80.4 (80.2, 80.5) | 19 (18, 20) |
| **Holmes** | 71.4 (70.7, 71.9) | 25 (23, 29) | 71.8 (71, 72.4) | 31 (28, 34) | 77.9 (77.3, 78.5) | 23 (21, 26) | 78 (77.3, 78.7) | 30 (28, 33) |
| **Indian River** | 76.3 (75.8, 76.7) | 3 (2, 5) | 77 (76.6, 77.4) | 7 (6, 9) | 82.3 (81.8, 82.7) | 2 (0, 4) | 82.6 (82.1, 83) | 8 (6, 10) |
| **Jackson** | 71.6 (71.1, 71.9) | 25 (23, 27) | 72.5 (72, 72.9) | 28 (26, 30) | 77.7 (77.3, 78.1) | 24 (23, 26) | 77.9 (77.4, 78.3) | 30 (29, 32) |
| **Jefferson** | 71.6 (71, 72) | 25 (23, 27) | 72.5 (71.9, 73) | 28 (26, 30) | 77.2 (76.7, 77.6) | 26 (25, 28) | 78.3 (77.6, 78.8) | 29 (27, 31) |
| **Lafayette** | 71.8 (71.2, 72.2) | 24 (22, 26) | 72.4 (71.8, 72.8) | 28 (26, 31) | 78.2 (77.6, 78.6) | 22 (21, 24) | 78.2 (77.6, 78.7) | 29 (27, 31) |
| **Lake** | 75 (74.7, 75.3) | 9 (8, 10) | 76.8 (76.5, 77.1) | 8 (7, 9) | 80.6 (80.3, 80.8) | 11 (9, 12) | 82.9 (82.6, 83.2) | 6 (5, 8) |
| **Lee** | 75.8 (75.5, 76) | 5 (4, 6) | 76.4 (76.2, 76.7) | 10 (9, 11) | 82 (81.7, 82.2) | 4 (2, 5) | 83.1 (82.8, 83.3) | 6 (4, 7) |
| **Leon** | 74.3 (74, 74.6) | 12 (11, 13) | 76.1 (75.8, 76.4) | 11 (10, 12) | 79.2 (78.9, 79.5) | 18 (17, 20) | 80.4 (80.1, 80.7) | 19 (17, 20) |
| **Levy** | 71.7 (71, 72.2) | 24 (22, 27) | 71.5 (70.9, 72) | 32 (30, 35) | 78.2 (77.6, 78.6) | 22 (21, 24) | 78.7 (78, 79.2) | 28 (25, 30) |
| **Liberty** | 72.2 (71.5, 72.7) | 22 (20, 25) | 72.4 (71.7, 73) | 28 (25, 31) | 77.4 (76.8, 77.9) | 25 (24, 28) | 78.5 (77.7, 79.2) | 28 (25, 31) |
| **Madison** | 70.3 (69.7, 70.6) | 31 (29, 37) | 70.2 (69.7, 70.6) | 39 (36, 45) | 76.3 (75.8, 76.7) | 30 (28, 33) | 76.5 (75.9, 76.9) | 36 (34, 39) |
| **Manatee** | 75.1 (74.8, 75.4) | 8 (7, 10) | 75.9 (75.6, 76.2) | 12 (10, 13) | 81.4 (81.1, 81.7) | 6 (5, 8) | 82.8 (82.5, 83.2) | 7 (5, 8) |
| **Marion** | 73.4 (73.1, 73.6) | 16 (15, 18) | 73.9 (73.7, 74.2) | 21 (20, 22) | 80.3 (80, 80.5) | 12 (11, 14) | 80.9 (80.6, 81.2) | 16 (14, 17) |
| **Martin** | 77 (76.6, 77.3) | 1 (-1, 2) | 77.7 (77.2, 78) | 5 (4, 7) | 82 (81.6, 82.3) | 3 (2, 5) | 83.4 (82.9, 83.7) | 4 (2, 6) |
| **Miami-Dade** | 74.8 (74.7, 74.9) | 10 (9, 10) | 76.6 (76.5, 76.7) | 9 (9, 10) | 80.7 (80.6, 80.8) | 10 (9, 10) | 82.8 (82.7, 82.9) | 7 (6, 7) |
| **Monroe** | 75.2 (74.7, 75.6) | 8 (6, 10) | 76 (75.5, 76.5) | 11 (9, 14) | 80.8 (80.4, 81.3) | 9 (7, 12) | 81.8 (81.2, 82.3) | 11 (9, 14) |
| **Nassau** | 73.4 (72.9, 73.8) | 16 (14, 19) | 74.6 (73.9, 75) | 18 (16, 21) | 79.4 (78.9, 79.9) | 17 (15, 20) | 79.4 (78.9, 79.9) | 24 (22, 27) |
| **Okaloosa** | 74.9 (74.5, 75.2) | 9 (8, 11) | 75.6 (75.2, 75.9) | 13 (12, 15) | 79 (78.7, 79.3) | 19 (18, 21) | 80.2 (79.8, 80.5) | 20 (18, 22) |
| **Okeechobee** | 71.3 (70.7, 71.8) | 26 (24, 28) | 72.2 (71.6, 72.7) | 29 (27, 32) | 77.6 (76.9, 78.1) | 25 (23, 27) | 78.7 (77.9, 79.3) | 28 (25, 30) |
| **Orange** | 74.1 (74, 74.3) | 13 (12, 14) | 75.5 (75.3, 75.6) | 14 (13, 14) | 80 (79.8, 80.1) | 14 (13, 15) | 80.9 (80.8, 81.1) | 16 (15, 17) |
| **Osceola** | 73.7 (73.4, 74) | 15 (13, 16) | 76 (75.7, 76.4) | 11 (10, 13) | 79.7 (79.3, 80.1) | 16 (14, 18) | 80.8 (80.4, 81.1) | 17 (15, 19) |
| **Palm Beach** | 76.5 (76.3, 76.6) | 2 (2, 3) | 77.1 (76.9, 77.2) | 7 (7, 8) | 81.9 (81.7, 82) | 4 (3, 5) | 83.5 (83.3, 83.6) | 4 (3, 4) |
| **Pasco** | 73.8 (73.5, 74) | 15 (13, 16) | 73.7 (73.4, 73.9) | 22 (21, 23) | 79.9 (79.6, 80.1) | 15 (13, 16) | 80.6 (80.4, 80.9) | 17 (16, 19) |
| **Pinellas** | 74.3 (74.1, 74.4) | 12 (12, 13) | 74.7 (74.6, 74.9) | 17 (16, 18) | 80.5 (80.3, 80.6) | 11 (10, 12) | 81.4 (81.2, 81.5) | 13 (12, 14) |
| **Polk** | 73.2 (73, 73.4) | 17 (16, 18) | 74.3 (74.1, 74.5) | 19 (18, 20) | 79.7 (79.5, 79.9) | 16 (14, 17) | 80 (79.8, 80.2) | 21 (20, 22) |
| **Putnam** | 71.2 (70.8, 71.6) | 26 (25, 28) | 70.9 (70.4, 71.2) | 35 (33, 38) | 77.5 (77.1, 77.9) | 25 (24, 26) | 78 (77.5, 78.4) | 30 (29, 32) |
| **Santa Rosa** | 74.1 (73.6, 74.4) | 13 (12, 15) | 75.6 (75.1, 76) | 13 (11, 15) | 79.1 (78.6, 79.4) | 19 (17, 21) | 79.8 (79.3, 80.2) | 22 (20, 25) |
| **Sarasota** | 76.5 (76.3, 76.8) | 2 (1, 3) | 77.8 (77.5, 78.1) | 5 (4, 6) | 82.6 (82.3, 82.9) | 1 (0, 2) | 83.8 (83.4, 84) | 2 (0, 4) |
| **Seminole** | 75.5 (75.3, 75.8) | 6 (5, 8) | 77.1 (76.9, 77.3) | 7 (6, 8) | 80.5 (80.3, 80.8) | 11 (9, 12) | 81.5 (81.2, 81.7) | 13 (12, 14) |
| **St. Johns** | 75.6 (75.1, 75.9) | 6 (5, 8) | 77.6 (77.2, 77.9) | 6 (4, 7) | 80.6 (80.2, 81) | 10 (8, 13) | 82.8 (82.3, 83.2) | 7 (5, 9) |
| **St. Lucie** | 73.5 (73.1, 73.8) | 16 (15, 18) | 75.1 (74.7, 75.3) | 16 (14, 17) | 79.9 (79.5, 80.2) | 15 (13, 17) | 81.5 (81.1, 81.8) | 13 (11, 15) |
| **Sumter** | 74.7 (74.1, 75.1) | 11 (8, 13) | 74.4 (73.9, 74.8) | 19 (17, 21) | 80.4 (79.8, 80.9) | 12 (9, 15) | 80.2 (79.6, 80.7) | 20 (17, 23) |
| **Suwannee** | 71.8 (71.2, 72.2) | 24 (22, 26) | 72.4 (71.8, 72.8) | 28 (26, 31) | 78.2 (77.6, 78.6) | 22 (21, 24) | 78.2 (77.6, 78.7) | 29 (27, 31) |
| **Taylor** | 71.6 (71, 72) | 25 (23, 27) | 72.5 (71.9, 73) | 28 (26, 30) | 77.2 (76.7, 77.6) | 26 (25, 28) | 78.3 (77.6, 78.8) | 29 (27, 31) |
| **Union** | 68 (67.4, 68.4) | 51 (50, 51) | 68.1 (67.5, 68.5) | 51 (51, 51) | 77.1 (76.5, 77.6) | 26 (25, 29) | 77.7 (77, 78.3) | 31 (29, 34) |
| **Volusia** | 74 (73.8, 74.2) | 13 (12, 15) | 74.3 (74.1, 74.5) | 19 (18, 20) | 80.1 (79.9, 80.4) | 13 (12, 14) | 80.6 (80.4, 80.8) | 17 (16, 19) |
| **Wakulla** | 72.8 (72.1, 73.3) | 19 (17, 23) | 74.5 (73.7, 75) | 18 (16, 22) | 78.3 (77.6, 78.8) | 22 (20, 25) | 79.8 (78.9, 80.4) | 22 (19, 26) |
| **Walton** | 73.1 (72.5, 73.5) | 18 (16, 21) | 73.7 (73.2, 74.2) | 22 (19, 24) | 78.8 (78.3, 79.4) | 20 (17, 22) | 79.7 (79.2, 80.3) | 22 (19, 25) |
| **Washington** | 71.4 (70.7, 71.9) | 25 (23, 29) | 71.3 (70.6, 71.8) | 33 (31, 36) | 78.9 (78.3, 79.5) | 20 (17, 22) | 77.6 (76.9, 78.1) | 32 (30, 34) |
| **GEORGIA** |  |  |  |  |  |  |  |  |
| **Appling** | 70.6 (69.9, 71.1) | 29 (27, 35) | 70.8 (70.1, 71.3) | 35 (33, 40) | 77.9 (77.3, 78.4) | 23 (22, 26) | 76.9 (76.2, 77.4) | 34 (32, 37) |
| **Atkinson** | 69.5 (68.8, 69.9) | 42 (35, 47) | 69.4 (68.6, 69.9) | 50 (42, 51) | 76.2 (75.5, 76.7) | 30 (28, 34) | 76.5 (75.7, 77.1) | 36 (33, 40) |
| **Bacon** | 69.3 (68.6, 69.7) | 44 (38, 48) | 68.8 (68.1, 69.3) | 51 (51, 51) | 77 (76.5, 77.5) | 27 (25, 29) | 76.4 (75.7, 76.9) | 36 (34, 40) |
| **Baker** | 70.6 (69.9, 71) | 29 (27, 35) | 70.1 (69.5, 70.6) | 39 (36, 49) | 78.3 (77.7, 78.8) | 22 (20, 24) | 77.2 (76.5, 77.6) | 33 (31, 36) |
| **Baldwin** | 72.1 (71.6, 72.4) | 23 (21, 25) | 72.1 (71.7, 72.5) | 29 (28, 31) | 77.5 (76.9, 77.9) | 25 (23, 27) | 77.7 (77.1, 78.1) | 31 (30, 33) |
| **Banks** | 72.4 (71.6, 72.9) | 21 (19, 25) | 72.9 (72, 73.6) | 26 (22, 30) | 79.6 (78.7, 80.3) | 16 (12, 20) | 79 (78, 79.7) | 26 (23, 30) |
| **Barrow** | 72.6 (71.9, 73) | 21 (18, 23) | 72.5 (71.9, 72.9) | 28 (26, 30) | 78.9 (78.3, 79.3) | 20 (18, 22) | 78.6 (77.9, 79.1) | 28 (26, 30) |
| **Bartow** | 71.9 (71.4, 72.3) | 23 (22, 25) | 71.9 (71.4, 72.3) | 30 (29, 32) | 77.7 (77.2, 78.1) | 24 (23, 26) | 78.2 (77.7, 78.6) | 29 (28, 31) |
| **Ben Hill** | 69.3 (68.7, 69.7) | 44 (39, 48) | 70.1 (69.5, 70.5) | 40 (37, 48) | 76.4 (75.9, 76.7) | 29 (28, 32) | 76 (75.4, 76.4) | 38 (36, 42) |
| **Berrien** | 71 (70.2, 71.5) | 27 (25, 32) | 71.1 (70.3, 71.6) | 34 (31, 38) | 77.8 (77, 78.4) | 24 (22, 27) | 77.6 (76.7, 78.2) | 32 (29, 35) |
| **Bibb** | 69.5 (69.2, 69.8) | 42 (37, 45) | 70 (69.7, 70.3) | 41 (38, 45) | 76.5 (76.2, 76.7) | 29 (28, 30) | 76.9 (76.6, 77.2) | 34 (33, 35) |
| **Bleckley** | 69.5 (68.9, 70) | 41 (34, 47) | 70.5 (69.9, 71) | 37 (34, 42) | 77.1 (76.6, 77.6) | 26 (24, 28) | 77.1 (76.5, 77.6) | 33 (32, 36) |
| **Brantley** | 71 (70.4, 71.5) | 27 (25, 31) | 70.6 (69.9, 71.1) | 36 (33, 42) | 77.5 (76.9, 78) | 25 (23, 27) | 77.2 (76.5, 77.8) | 33 (31, 36) |
| **Brooks** | 69.4 (68.7, 69.8) | 43 (36, 48) | 70.1 (69.4, 70.6) | 40 (36, 49) | 76.7 (76.1, 77.1) | 28 (26, 31) | 76.7 (76.1, 77.2) | 35 (33, 38) |
| **Bryan** | 72.9 (72.3, 73.3) | 19 (17, 22) | 73.7 (73.1, 74.2) | 22 (20, 25) | 78.8 (78.1, 79.3) | 20 (18, 23) | 78.9 (78.2, 79.4) | 27 (24, 29) |
| **Bulloch** | 72.6 (72.2, 73) | 20 (18, 22) | 73.2 (72.7, 73.6) | 24 (22, 27) | 78.5 (78, 78.9) | 21 (19, 23) | 78.9 (78.4, 79.4) | 27 (24, 29) |
| **Burke** | 68.1 (67.4, 68.6) | 51 (48, 51) | 68.3 (67.6, 68.8) | 51 (51, 51) | 75.4 (74.8, 75.8) | 35 (32, 39) | 75 (74.3, 75.4) | 45 (42, 49) |
| **Butts** | 71.1 (70.5, 71.6) | 27 (25, 30) | 71.9 (71.3, 72.3) | 30 (29, 33) | 77.7 (77.1, 78.1) | 24 (23, 27) | 76.9 (76.3, 77.5) | 34 (32, 37) |
| **Calhoun** | 68.1 (67.5, 68.5) | 51 (49, 51) | 67.6 (67, 68) | 51 (51, 51) | 75.8 (75.2, 76.2) | 32 (30, 36) | 74.7 (74.1, 75.1) | 46 (44, 50) |
| **Camden** | 73.4 (72.8, 73.8) | 16 (15, 19) | 74.2 (73.6, 74.7) | 20 (17, 22) | 78.9 (78.3, 79.4) | 20 (17, 22) | 79.5 (78.8, 80) | 24 (21, 27) |
| **Candler** | 69.1 (68.4, 69.5) | 46 (42, 50) | 70.1 (69.4, 70.5) | 40 (37, 50) | 76.6 (76, 77) | 28 (27, 31) | 77.7 (77, 78.2) | 31 (29, 34) |
| **Carroll** | 71.6 (71.2, 72) | 24 (23, 26) | 72.6 (72.2, 73) | 27 (25, 29) | 77.7 (77.3, 78.1) | 24 (23, 26) | 77.8 (77.4, 78.2) | 31 (29, 32) |
| **Catoosa** | 73.8 (73.1, 74.2) | 15 (12, 18) | 73.3 (72.7, 73.8) | 24 (21, 27) | 79.3 (78.7, 79.7) | 18 (15, 20) | 79.2 (78.6, 79.6) | 25 (23, 28) |
| **Charlton** | 71 (70.4, 71.5) | 27 (25, 31) | 70.6 (69.9, 71.1) | 36 (33, 42) | 77.5 (76.9, 78) | 25 (23, 27) | 77.2 (76.5, 77.8) | 33 (31, 36) |
| **Chatham** | 72.2 (72, 72.4) | 22 (21, 23) | 73 (72.7, 73.2) | 25 (24, 27) | 78.1 (77.9, 78.3) | 23 (22, 24) | 78.4 (78.1, 78.6) | 29 (28, 30) |
| **Chattahoochee** | 71.3 (70.7, 71.6) | 26 (25, 29) | 71.9 (71.3, 72.3) | 30 (29, 33) | 77.4 (76.8, 77.8) | 25 (24, 28) | 78.2 (77.5, 78.7) | 29 (28, 32) |
| **Chattooga** | 70.8 (70.2, 71.2) | 28 (26, 32) | 70.1 (69.5, 70.6) | 40 (36, 49) | 77.5 (76.9, 78) | 25 (23, 27) | 76.9 (76.3, 77.4) | 34 (32, 37) |
| **Cherokee** | 74.4 (74, 74.8) | 12 (10, 13) | 76 (75.5, 76.3) | 12 (10, 13) | 79.3 (78.8, 79.7) | 18 (16, 20) | 79.7 (79.3, 80.1) | 23 (21, 25) |
| **Clarke** | 73.2 (72.8, 73.5) | 17 (16, 19) | 74.3 (73.8, 74.6) | 19 (18, 21) | 78.7 (78.3, 79) | 20 (19, 22) | 79.6 (79.1, 79.9) | 23 (21, 26) |
| **Clay** | 69.1 (68.4, 69.5) | 46 (42, 50) | 69.6 (69, 70.1) | 46 (40, 51) | 76.6 (76.1, 77) | 28 (27, 31) | 75.8 (75.1, 76.2) | 40 (37, 44) |
| **Clayton** | 72 (71.6, 72.3) | 23 (22, 24) | 73.5 (73.2, 73.8) | 23 (22, 24) | 77.3 (77, 77.6) | 26 (25, 27) | 78.8 (78.4, 79.1) | 27 (26, 29) |
| **Clinch** | 71.5 (71.1, 71.8) | 25 (24, 27) | 72.2 (71.8, 72.5) | 29 (28, 31) | 77.3 (76.9, 77.6) | 26 (25, 27) | 77.9 (77.5, 78.2) | 30 (29, 32) |
| **Cobb** | 75.4 (75.2, 75.6) | 7 (6, 8) | 77.6 (77.4, 77.8) | 6 (5, 6) | 79.7 (79.4, 79.9) | 16 (15, 17) | 81.3 (81.1, 81.6) | 13 (12, 15) |
| **Coffee** | 70.3 (69.7, 70.8) | 31 (28, 38) | 70.2 (69.5, 70.6) | 39 (36, 48) | 77.1 (76.5, 77.6) | 26 (25, 29) | 78 (77.3, 78.5) | 30 (28, 33) |
| **Colquitt** | 70.5 (70, 70.9) | 30 (27, 34) | 70.9 (70.4, 71.4) | 34 (32, 37) | 77.2 (76.7, 77.6) | 26 (25, 28) | 77.2 (76.7, 77.7) | 33 (31, 35) |
| **Columbia** | 75.2 (74.7, 75.6) | 8 (6, 10) | 75.4 (75, 75.7) | 14 (12, 16) | 79.6 (79.1, 80) | 16 (14, 19) | 80.2 (79.7, 80.6) | 20 (17, 22) |
| **Cook** | 69.6 (68.9, 70) | 41 (34, 47) | 70 (69.3, 70.4) | 41 (37, 51) | 76.6 (76, 77.1) | 29 (27, 31) | 76.4 (75.7, 77) | 36 (34, 40) |
| **Coweta** | 73.1 (72.7, 73.4) | 18 (16, 20) | 74.1 (73.7, 74.4) | 20 (19, 22) | 78.6 (78.2, 79) | 21 (19, 22) | 79.1 (78.7, 79.4) | 26 (24, 27) |
| **Crawford** | 70.1 (69.5, 70.5) | 33 (30, 42) | 70.6 (70, 71) | 36 (34, 41) | 77.4 (76.8, 77.8) | 25 (24, 28) | 76.6 (76, 77.1) | 35 (33, 38) |
| **Crisp** | 68.7 (68, 69.1) | 48 (45, 51) | 69.8 (69.1, 70.2) | 44 (39, 51) | 76.4 (75.9, 76.8) | 29 (28, 32) | 76.6 (76, 77.1) | 35 (33, 38) |
| **Dade** | 72.4 (71.6, 73) | 21 (18, 25) | 71.7 (70.9, 72.4) | 31 (28, 35) | 79 (78.2, 79.7) | 19 (16, 22) | 78.3 (77.5, 79) | 29 (26, 32) |
| **Dawson** | 75 (74.1, 75.6) | 9 (6, 13) | 74.6 (73.8, 75.2) | 18 (15, 21) | 79.5 (78.7, 80.2) | 17 (13, 20) | 80.2 (79.3, 81) | 20 (15, 25) |
| **De Kalb** | 73.5 (73.3, 73.6) | 16 (15, 17) | 75.9 (75.7, 76.1) | 12 (11, 13) | 79.4 (79.2, 79.6) | 17 (16, 18) | 81.4 (81.1, 81.6) | 13 (12, 15) |
| **Decatur** | 70.1 (69.5, 70.5) | 33 (30, 42) | 70.7 (70.1, 71.1) | 36 (34, 40) | 77.1 (76.5, 77.5) | 26 (25, 29) | 77.8 (77.2, 78.2) | 31 (29, 33) |
| **Dodge** | 69.5 (68.9, 69.9) | 41 (36, 47) | 70.7 (70, 71.1) | 36 (34, 41) | 76.6 (76.1, 77) | 28 (27, 31) | 76.4 (75.8, 76.9) | 36 (34, 40) |
| **Dooly** | 68.7 (68.1, 69.1) | 48 (45, 51) | 69.2 (68.6, 69.6) | 51 (47, 51) | 75.6 (75.1, 76) | 33 (31, 37) | 76.2 (75.5, 76.6) | 37 (35, 41) |
| **Dougherty** | 69.8 (69.3, 70.1) | 37 (33, 43) | 70.8 (70.3, 71.1) | 35 (34, 38) | 77.2 (76.8, 77.5) | 26 (25, 28) | 77.6 (77.2, 77.9) | 32 (30, 33) |
| **Douglas** | 73.2 (72.8, 73.6) | 17 (15, 20) | 73.4 (72.9, 73.7) | 23 (22, 26) | 78.4 (78, 78.9) | 21 (20, 23) | 77.5 (77.1, 77.9) | 32 (30, 33) |
| **Early** | 69.1 (68.4, 69.5) | 46 (42, 50) | 69.6 (69, 70.1) | 46 (40, 51) | 76.6 (76.1, 77) | 28 (27, 31) | 75.8 (75.1, 76.2) | 40 (37, 44) |
| **Echols** | 71.5 (71.1, 71.8) | 25 (24, 27) | 72.2 (71.8, 72.5) | 29 (28, 31) | 77.3 (76.9, 77.6) | 26 (25, 27) | 77.9 (77.5, 78.2) | 30 (29, 32) |
| **Effingham** | 73.3 (72.8, 73.8) | 17 (15, 20) | 74.4 (73.8, 74.9) | 19 (16, 22) | 79 (78.3, 79.5) | 19 (17, 22) | 78.8 (78.2, 79.4) | 27 (24, 29) |
| **Elbert** | 70.7 (70.2, 71.1) | 28 (27, 32) | 71.7 (71.1, 72.2) | 31 (29, 33) | 77.8 (77.3, 78.2) | 24 (22, 26) | 78.1 (77.6, 78.6) | 30 (28, 32) |
| **Emanuel** | 68.7 (68, 69.1) | 48 (45, 51) | 69.3 (68.7, 69.8) | 50 (44, 51) | 76.7 (76.1, 77.1) | 28 (26, 30) | 75.6 (75, 76) | 41 (38, 45) |
| **Evans** | 69.1 (68.4, 69.5) | 46 (42, 50) | 70.1 (69.4, 70.5) | 40 (37, 50) | 76.6 (76, 77) | 28 (27, 31) | 77.7 (77, 78.2) | 31 (29, 34) |
| **Fannin** | 73.1 (72.3, 73.7) | 18 (15, 22) | 72.9 (72.1, 73.5) | 26 (23, 29) | 79.5 (78.8, 80.1) | 17 (14, 20) | 79.3 (78.5, 80) | 25 (21, 28) |
| **Fayette** | 76.2 (75.8, 76.6) | 3 (2, 5) | 77.8 (77.4, 78.2) | 5 (3, 6) | 80.9 (80.4, 81.4) | 9 (6, 12) | 82 (81.5, 82.5) | 10 (8, 13) |
| **Floyd** | 71.8 (71.3, 72.1) | 24 (23, 26) | 72.1 (71.6, 72.4) | 30 (28, 31) | 77.5 (77.1, 77.8) | 25 (24, 26) | 78.2 (77.7, 78.6) | 29 (28, 31) |
| **Forsyth** | 75.6 (75.1, 76) | 6 (4, 9) | 77.2 (76.7, 77.6) | 7 (5, 9) | 80.5 (80, 81) | 11 (8, 14) | 81.8 (81.2, 82.3) | 11 (9, 14) |
| **Franklin** | 71.6 (70.9, 72.2) | 25 (22, 28) | 72 (71.3, 72.6) | 30 (27, 33) | 78.5 (77.9, 79) | 21 (19, 24) | 78.9 (78.1, 79.6) | 26 (23, 30) |
| **Fulton** | 71.3 (71.1, 71.4) | 26 (25, 27) | 75 (74.8, 75.1) | 16 (15, 17) | 77.4 (77.2, 77.6) | 25 (25, 26) | 80.2 (80, 80.3) | 20 (19, 21) |
| **Gilmer** | 72.8 (72, 73.4) | 19 (16, 23) | 72.6 (71.8, 73.2) | 27 (24, 31) | 78.7 (78, 79.4) | 20 (17, 23) | 78.5 (77.7, 79.2) | 28 (25, 31) |
| **Glascock** | 68.1 (67.4, 68.6) | 51 (48, 51) | 68.4 (67.7, 68.8) | 51 (51, 51) | 75.1 (74.5, 75.6) | 37 (34, 41) | 75.4 (74.7, 75.9) | 42 (39, 46) |
| **Glynn** | 73.1 (72.7, 73.4) | 18 (16, 20) | 73 (72.5, 73.3) | 26 (24, 28) | 78.7 (78.3, 79) | 20 (19, 22) | 79 (78.6, 79.3) | 26 (25, 28) |
| **Gordon** | 71.7 (71.1, 72.2) | 24 (22, 27) | 72 (71.4, 72.5) | 30 (28, 32) | 77.9 (77.3, 78.4) | 23 (21, 25) | 78.5 (77.8, 79.1) | 28 (26, 31) |
| **Grady** | 70 (69.3, 70.4) | 34 (30, 44) | 70.8 (70.1, 71.2) | 35 (33, 40) | 77.4 (76.8, 77.9) | 25 (24, 28) | 78 (77.3, 78.6) | 30 (28, 32) |
| **Greene** | 71.6 (71, 72) | 24 (23, 27) | 73.2 (72.6, 73.7) | 24 (22, 27) | 77.8 (77.3, 78.3) | 24 (22, 26) | 78.5 (77.9, 78.9) | 28 (26, 30) |
| **Gwinnett** | 75.8 (75.5, 76) | 5 (4, 6) | 77.2 (76.9, 77.4) | 7 (6, 8) | 80.5 (80.2, 80.8) | 11 (9, 13) | 80.9 (80.6, 81.1) | 16 (15, 17) |
| **Habersham** | 73.5 (72.9, 74) | 16 (13, 19) | 74.6 (74, 75.2) | 18 (15, 21) | 79.4 (78.8, 79.9) | 17 (15, 20) | 80 (79.3, 80.7) | 21 (17, 25) |
| **Hall** | 73.5 (73.1, 73.9) | 16 (14, 18) | 74.7 (74.4, 75.1) | 17 (15, 19) | 78.7 (78.3, 79) | 20 (19, 22) | 79.7 (79.3, 80.1) | 22 (20, 25) |
| **Hancock** | 68.1 (67.4, 68.6) | 51 (48, 51) | 68.4 (67.7, 68.8) | 51 (51, 51) | 75.1 (74.5, 75.6) | 37 (34, 41) | 75.4 (74.7, 75.9) | 42 (39, 46) |
| **Haralson** | 71.2 (70.5, 71.7) | 26 (24, 30) | 71.4 (70.7, 72) | 32 (30, 36) | 78.3 (77.6, 78.8) | 22 (20, 24) | 77.7 (76.9, 78.3) | 31 (29, 34) |
| **Harris** | 73.2 (72.6, 73.6) | 17 (15, 20) | 74.9 (74.3, 75.3) | 16 (14, 19) | 79 (78.5, 79.5) | 19 (17, 21) | 80.1 (79.4, 80.7) | 20 (17, 24) |
| **Hart** | 72 (71.4, 72.5) | 23 (21, 25) | 72 (71.4, 72.5) | 30 (28, 32) | 78.4 (77.8, 78.8) | 22 (20, 24) | 78.4 (77.8, 79) | 28 (26, 31) |
| **Heard** | 70.2 (69.7, 70.5) | 32 (30, 37) | 71.4 (70.9, 71.7) | 33 (31, 35) | 76.7 (76.3, 77) | 28 (27, 30) | 77.1 (76.6, 77.5) | 33 (32, 35) |
| **Henry** | 73.7 (73.3, 74.1) | 15 (13, 17) | 74 (73.6, 74.3) | 21 (19, 22) | 78.7 (78.3, 79.1) | 20 (19, 22) | 78.7 (78.3, 79) | 28 (26, 29) |
| **Houston** | 73.6 (73.2, 73.9) | 15 (14, 17) | 74.2 (73.8, 74.5) | 20 (18, 21) | 78.2 (77.8, 78.5) | 22 (21, 24) | 79.6 (79.1, 79.9) | 23 (21, 26) |
| **Irwin** | 69.6 (68.9, 70) | 40 (33, 46) | 70.8 (70.1, 71.2) | 35 (33, 40) | 76.7 (76.2, 77.2) | 28 (26, 30) | 76.6 (76, 77.1) | 35 (33, 38) |
| **Jackson** | 71.5 (70.9, 71.9) | 25 (23, 28) | 72.2 (71.5, 72.6) | 29 (27, 32) | 78 (77.4, 78.5) | 23 (21, 25) | 78.6 (77.9, 79.1) | 28 (26, 30) |
| **Jasper** | 72.1 (71.6, 72.5) | 22 (21, 25) | 73 (72.5, 73.4) | 25 (23, 28) | 78.4 (77.8, 78.8) | 22 (20, 24) | 78.7 (78.1, 79.1) | 27 (26, 30) |
| **Jeff Davis** | 69.3 (68.6, 69.7) | 44 (38, 48) | 68.8 (68.1, 69.3) | 51 (51, 51) | 77 (76.5, 77.5) | 27 (25, 29) | 76.4 (75.7, 76.9) | 36 (34, 40) |
| **Jefferson** | 67.4 (66.6, 67.9) | 51 (51, 51) | 67.9 (67.1, 68.3) | 51 (51, 51) | 75.3 (74.6, 75.7) | 36 (33, 40) | 75.2 (74.5, 75.7) | 43 (40, 48) |
| **Jenkins** | 69.2 (68.5, 69.6) | 45 (41, 49) | 70.1 (69.4, 70.5) | 40 (37, 50) | 76.6 (76.1, 77) | 28 (27, 31) | 76.4 (75.8, 76.8) | 36 (34, 40) |
| **Johnson** | 69.9 (69.2, 70.3) | 35 (31, 44) | 70.6 (70, 71) | 36 (34, 41) | 76.9 (76.3, 77.3) | 27 (26, 30) | 76.1 (75.5, 76.6) | 38 (35, 41) |
| **Jones** | 72.2 (71.6, 72.6) | 22 (20, 24) | 73.1 (72.5, 73.5) | 25 (23, 28) | 78.6 (78.1, 79.1) | 21 (19, 23) | 79.3 (78.7, 79.8) | 25 (22, 27) |
| **Lamar** | 71.4 (70.8, 71.8) | 25 (24, 28) | 72.1 (71.5, 72.5) | 29 (28, 32) | 78.2 (77.6, 78.6) | 22 (21, 24) | 77.9 (77.2, 78.3) | 31 (29, 33) |
| **Lanier** | 69.5 (68.8, 69.9) | 42 (35, 47) | 69.4 (68.6, 69.9) | 50 (42, 51) | 76.2 (75.5, 76.7) | 30 (28, 34) | 76.5 (75.7, 77.1) | 36 (33, 40) |
| **Laurens** | 70.8 (70.3, 71.1) | 28 (27, 31) | 71.4 (71, 71.8) | 32 (31, 34) | 77.7 (77.2, 78) | 24 (23, 26) | 77.2 (76.7, 77.6) | 33 (32, 35) |
| **Lee** | 73.8 (73.1, 74.2) | 15 (13, 18) | 74.9 (74.2, 75.4) | 17 (14, 20) | 80 (79.3, 80.6) | 14 (11, 18) | 80 (79.2, 80.6) | 21 (17, 25) |
| **Liberty** | 71.8 (71.3, 72.2) | 24 (22, 26) | 73 (72.5, 73.4) | 25 (23, 28) | 77.2 (76.7, 77.5) | 26 (25, 28) | 78.1 (77.5, 78.6) | 30 (28, 32) |
| **Lincoln** | 69.6 (69, 70) | 40 (34, 46) | 70.8 (70.2, 71.3) | 35 (33, 39) | 76.7 (76.2, 77.1) | 28 (26, 30) | 76.8 (76.2, 77.2) | 35 (33, 37) |
| **Long** | 70.3 (69.6, 70.7) | 31 (29, 39) | 70.6 (69.9, 71.1) | 36 (34, 42) | 77.2 (76.6, 77.7) | 26 (24, 28) | 77.8 (77.1, 78.3) | 31 (29, 33) |
| **Lowndes** | 71.5 (71.1, 71.8) | 25 (24, 27) | 72.2 (71.8, 72.5) | 29 (28, 31) | 77.3 (76.9, 77.6) | 26 (25, 27) | 77.9 (77.5, 78.2) | 30 (29, 32) |
| **Lumpkin** | 72.9 (72.1, 73.4) | 19 (16, 22) | 73.6 (72.8, 74.3) | 22 (19, 26) | 78.8 (78.1, 79.4) | 20 (17, 23) | 79 (78.2, 79.7) | 26 (23, 29) |
| **Macon** | 68.7 (68.1, 69.1) | 48 (45, 51) | 69.2 (68.6, 69.6) | 51 (47, 51) | 75.6 (75.1, 76) | 33 (31, 37) | 76.2 (75.5, 76.6) | 37 (35, 41) |
| **Madison** | 72.7 (72, 73.2) | 20 (17, 23) | 72.5 (71.8, 73) | 28 (25, 31) | 78.4 (77.8, 78.9) | 21 (19, 24) | 78.7 (78, 79.3) | 27 (25, 30) |
| **Marion** | 71.3 (70.7, 71.6) | 26 (25, 29) | 71.9 (71.3, 72.3) | 30 (29, 33) | 77.4 (76.8, 77.8) | 25 (24, 28) | 78.2 (77.5, 78.7) | 29 (28, 32) |
| **McDuffie** | 69.3 (68.6, 69.7) | 44 (38, 48) | 70.1 (69.5, 70.6) | 39 (36, 49) | 76.6 (76, 77) | 29 (27, 31) | 76.3 (75.7, 76.7) | 37 (35, 40) |
| **McIntosh** | 70.3 (69.6, 70.7) | 31 (29, 39) | 70.6 (69.9, 71.1) | 36 (34, 42) | 77.2 (76.6, 77.7) | 26 (24, 28) | 77.8 (77.1, 78.3) | 31 (29, 33) |
| **Meriwether** | 68.4 (67.8, 68.8) | 50 (47, 51) | 69.4 (68.8, 69.8) | 50 (44, 51) | 76.6 (76.1, 77) | 28 (27, 31) | 76 (75.5, 76.4) | 38 (36, 41) |
| **Miller** | 70.6 (69.9, 71) | 29 (27, 35) | 70.1 (69.5, 70.6) | 39 (36, 49) | 78.3 (77.7, 78.8) | 22 (20, 24) | 77.2 (76.5, 77.6) | 33 (31, 36) |
| **Mitchell** | 68.9 (68.3, 69.3) | 46 (44, 51) | 69.8 (69.2, 70.2) | 44 (39, 51) | 76.5 (75.9, 76.9) | 29 (27, 32) | 76.7 (76.1, 77.1) | 35 (33, 38) |
| **Monroe** | 71.6 (71.1, 72) | 24 (23, 27) | 73.1 (72.5, 73.5) | 25 (23, 28) | 77.7 (77.1, 78.1) | 24 (23, 26) | 78.1 (77.5, 78.5) | 30 (28, 32) |
| **Montgomery** | 70.1 (69.5, 70.5) | 33 (30, 41) | 71.3 (70.7, 71.7) | 33 (31, 36) | 77.4 (76.8, 77.8) | 25 (24, 28) | 76.5 (75.8, 76.9) | 36 (34, 39) |
| **Morgan** | 71.6 (71, 72) | 24 (23, 27) | 73.2 (72.6, 73.7) | 24 (22, 27) | 77.8 (77.3, 78.3) | 24 (22, 26) | 78.5 (77.9, 78.9) | 28 (26, 30) |
| **Murray** | 71.3 (70.6, 71.8) | 26 (24, 29) | 70.8 (70.2, 71.4) | 35 (32, 39) | 77.8 (77.2, 78.4) | 24 (21, 26) | 77.8 (77, 78.4) | 31 (28, 34) |
| **Muscogee** | 70.3 (70, 70.5) | 31 (30, 34) | 70.9 (70.6, 71.1) | 35 (34, 36) | 76.2 (76, 76.5) | 30 (29, 31) | 77.5 (77.1, 77.7) | 32 (31, 33) |
| **Newton** | 72.4 (71.9, 72.8) | 21 (20, 23) | 72.6 (72.1, 72.9) | 27 (26, 29) | 77.7 (77.2, 78) | 24 (23, 26) | 78.1 (77.6, 78.5) | 30 (28, 32) |
| **Oconee** | 75.7 (75, 76.2) | 6 (4, 9) | 76.8 (76.1, 77.4) | 8 (6, 11) | 80.3 (79.6, 80.8) | 12 (9, 16) | 81.3 (80.5, 81.9) | 14 (11, 18) |
| **Oglethorpe** | 70.7 (70.2, 71.1) | 28 (27, 32) | 71.7 (71.1, 72.2) | 31 (29, 33) | 77.8 (77.3, 78.2) | 24 (22, 26) | 78.1 (77.6, 78.6) | 30 (28, 32) |
| **Paulding** | 73.3 (72.8, 73.8) | 17 (15, 19) | 74.1 (73.7, 74.6) | 20 (18, 22) | 79.2 (78.7, 79.8) | 18 (15, 21) | 78.4 (77.9, 78.9) | 29 (27, 30) |
| **Peach** | 70.1 (69.5, 70.5) | 33 (30, 42) | 70.6 (70, 71.1) | 36 (34, 41) | 76.6 (76.1, 77.1) | 28 (27, 31) | 76.3 (75.8, 76.8) | 37 (35, 40) |
| **Pickens** | 73.3 (72.5, 73.8) | 17 (14, 21) | 74.5 (73.8, 75.1) | 18 (15, 22) | 79.3 (78.6, 79.9) | 18 (15, 21) | 79.7 (79, 80.4) | 22 (19, 26) |
| **Pierce** | 70.8 (70, 71.3) | 28 (26, 34) | 70.3 (69.5, 70.9) | 38 (35, 48) | 78.7 (78, 79.2) | 21 (18, 23) | 77.5 (76.8, 78.1) | 32 (30, 35) |
| **Pike** | 71.4 (70.8, 71.8) | 25 (24, 28) | 72.1 (71.5, 72.5) | 29 (28, 32) | 78.2 (77.6, 78.6) | 22 (21, 24) | 77.9 (77.2, 78.3) | 31 (29, 33) |
| **Polk** | 70.3 (69.8, 70.8) | 31 (28, 37) | 70.4 (69.8, 70.9) | 37 (35, 43) | 77.2 (76.7, 77.7) | 26 (24, 28) | 76.9 (76.3, 77.4) | 34 (32, 37) |
| **Pulaski** | 70.8 (70.2, 71.2) | 28 (26, 32) | 70.9 (70.3, 71.3) | 35 (33, 38) | 77 (76.4, 77.4) | 27 (25, 29) | 77.1 (76.4, 77.6) | 33 (32, 36) |
| **Putnam** | 72.1 (71.6, 72.5) | 22 (21, 25) | 73 (72.5, 73.4) | 25 (23, 28) | 78.4 (77.8, 78.8) | 22 (20, 24) | 78.7 (78.1, 79.1) | 27 (26, 30) |
| **Quitman** | 69.1 (68.4, 69.5) | 46 (42, 50) | 69.6 (69, 70.1) | 46 (40, 51) | 76.6 (76.1, 77) | 28 (27, 31) | 75.8 (75.1, 76.2) | 40 (37, 44) |
| **Rabun** | 73.9 (73.2, 74.5) | 14 (11, 17) | 75.1 (74.3, 75.8) | 15 (12, 19) | 79.9 (79.2, 80.4) | 15 (12, 18) | 81 (80.2, 81.7) | 15 (12, 20) |
| **Randolph** | 68.1 (67.5, 68.5) | 51 (49, 51) | 67.6 (67, 68) | 51 (51, 51) | 75.8 (75.2, 76.2) | 32 (30, 36) | 74.7 (74.1, 75.1) | 46 (44, 50) |
| **Richmond** | 69.4 (69.1, 69.6) | 43 (40, 45) | 69.7 (69.4, 69.9) | 45 (42, 50) | 76.2 (75.9, 76.5) | 30 (29, 32) | 77.2 (76.9, 77.4) | 33 (32, 34) |
| **Rockdale** | 73.9 (73.4, 74.3) | 14 (12, 16) | 74.4 (73.9, 74.8) | 19 (17, 21) | 79.6 (79.1, 80.1) | 16 (14, 19) | 78.9 (78.4, 79.3) | 27 (25, 29) |
| **Schley** | 70 (69.5, 70.5) | 33 (30, 41) | 70.9 (70.3, 71.3) | 35 (33, 38) | 76.7 (76.2, 77.1) | 28 (27, 30) | 77 (76.5, 77.5) | 34 (32, 36) |
| **Screven** | 69.2 (68.5, 69.6) | 45 (41, 49) | 70.1 (69.4, 70.5) | 40 (37, 50) | 76.6 (76.1, 77) | 28 (27, 31) | 76.4 (75.8, 76.8) | 36 (34, 40) |
| **Seminole** | 70.6 (69.9, 71) | 29 (27, 35) | 70.1 (69.5, 70.6) | 39 (36, 49) | 78.3 (77.7, 78.8) | 22 (20, 24) | 77.2 (76.5, 77.6) | 33 (31, 36) |
| **Spalding** | 71.2 (70.6, 71.5) | 26 (25, 29) | 71.2 (70.7, 71.6) | 33 (32, 36) | 76.9 (76.4, 77.3) | 27 (26, 29) | 77 (76.5, 77.4) | 34 (32, 36) |
| **Stephens** | 71.9 (71.3, 72.3) | 23 (22, 26) | 71.7 (71, 72.2) | 31 (29, 34) | 78.6 (78.1, 79.1) | 21 (19, 23) | 78.1 (77.5, 78.6) | 30 (28, 32) |
| **Stewart** | 69.1 (68.4, 69.5) | 46 (42, 50) | 69.6 (69, 70.1) | 46 (40, 51) | 76.6 (76.1, 77) | 28 (27, 31) | 75.8 (75.1, 76.2) | 40 (37, 44) |
| **Sumter** | 70 (69.5, 70.5) | 33 (30, 41) | 70.9 (70.3, 71.3) | 35 (33, 38) | 76.7 (76.2, 77.1) | 28 (27, 30) | 77 (76.5, 77.5) | 34 (32, 36) |
| **Talbot** | 68.4 (67.8, 68.8) | 50 (47, 51) | 69.4 (68.8, 69.8) | 50 (44, 51) | 76.6 (76.1, 77) | 28 (27, 31) | 76 (75.5, 76.4) | 38 (36, 41) |
| **Taliaferro** | 69.6 (69, 70) | 40 (34, 46) | 70.8 (70.2, 71.3) | 35 (33, 39) | 76.7 (76.2, 77.1) | 28 (26, 30) | 76.8 (76.2, 77.2) | 35 (33, 37) |
| **Tattnall** | 70.3 (69.7, 70.7) | 31 (29, 38) | 70.6 (69.9, 71) | 36 (34, 42) | 77.3 (76.7, 77.8) | 26 (24, 28) | 77.1 (76.4, 77.7) | 33 (31, 36) |
| **Taylor** | 70.1 (69.5, 70.5) | 33 (30, 42) | 70.6 (70, 71) | 36 (34, 41) | 77.4 (76.8, 77.8) | 25 (24, 28) | 76.6 (76, 77.1) | 35 (33, 38) |
| **Telfair** | 69.3 (68.7, 69.7) | 44 (39, 48) | 70.1 (69.5, 70.5) | 40 (37, 48) | 76.4 (75.9, 76.7) | 29 (28, 32) | 76 (75.4, 76.4) | 38 (36, 42) |
| **Terrell** | 68.1 (67.5, 68.5) | 51 (49, 51) | 67.6 (67, 68) | 51 (51, 51) | 75.8 (75.2, 76.2) | 32 (30, 36) | 74.7 (74.1, 75.1) | 46 (44, 50) |
| **Thomas** | 70.3 (69.8, 70.6) | 31 (29, 37) | 72 (71.4, 72.3) | 30 (29, 32) | 77.1 (76.6, 77.4) | 26 (25, 28) | 78.8 (78.3, 79.2) | 27 (25, 29) |
| **Tift** | 70.4 (69.9, 70.8) | 30 (28, 36) | 72.1 (71.5, 72.5) | 29 (28, 32) | 77.3 (76.8, 77.7) | 26 (24, 28) | 78.2 (77.6, 78.7) | 29 (28, 32) |
| **Toombs** | 69.4 (68.8, 69.8) | 43 (36, 47) | 70.6 (70, 71.1) | 36 (34, 41) | 77.4 (76.8, 77.8) | 25 (24, 27) | 77.5 (76.8, 78) | 32 (30, 34) |
| **Towns** | 73.9 (73.2, 74.5) | 14 (11, 17) | 75.1 (74.3, 75.8) | 15 (12, 19) | 79.9 (79.2, 80.4) | 15 (12, 18) | 81 (80.2, 81.7) | 15 (12, 20) |
| **Treutlen** | 70.1 (69.5, 70.5) | 33 (30, 41) | 71.3 (70.7, 71.7) | 33 (31, 36) | 77.4 (76.8, 77.8) | 25 (24, 28) | 76.5 (75.8, 76.9) | 36 (34, 39) |
| **Troup** | 70.2 (69.7, 70.5) | 32 (30, 37) | 71.4 (70.9, 71.7) | 33 (31, 35) | 76.7 (76.3, 77) | 28 (27, 30) | 77.1 (76.6, 77.5) | 33 (32, 35) |
| **Turner** | 69.6 (68.9, 70) | 40 (33, 46) | 70.8 (70.1, 71.2) | 35 (33, 40) | 76.7 (76.2, 77.2) | 28 (26, 30) | 76.6 (76, 77.1) | 35 (33, 38) |
| **Twiggs** | 69.5 (68.9, 70) | 41 (34, 47) | 70.5 (69.9, 71) | 37 (34, 42) | 77.1 (76.6, 77.6) | 26 (24, 28) | 77.1 (76.5, 77.6) | 33 (32, 36) |
| **Union** | 73.8 (73.1, 74.4) | 14 (12, 18) | 74.9 (74.1, 75.5) | 16 (13, 20) | 79.7 (79, 80.4) | 15 (12, 19) | 80.1 (79.3, 80.8) | 20 (16, 25) |
| **Upson** | 70.4 (69.8, 70.8) | 30 (28, 36) | 70.5 (69.9, 70.9) | 37 (35, 42) | 76.8 (76.3, 77.1) | 28 (26, 30) | 76.3 (75.8, 76.8) | 36 (35, 39) |
| **Walker** | 71 (70.5, 71.4) | 27 (25, 30) | 71.3 (70.8, 71.7) | 33 (31, 35) | 77.8 (77.3, 78.2) | 24 (22, 25) | 78 (77.4, 78.4) | 30 (29, 32) |
| **Walton** | 72.6 (72.1, 73) | 20 (18, 22) | 74.4 (73.9, 74.8) | 19 (17, 21) | 79 (78.5, 79.4) | 19 (17, 21) | 79.6 (79.1, 80) | 23 (21, 26) |
| **Ware** | 70.5 (69.9, 70.9) | 30 (28, 35) | 69.7 (69.1, 70.1) | 46 (40, 51) | 77.7 (77.2, 78.2) | 24 (22, 26) | 76.6 (76.1, 77.1) | 35 (34, 38) |
| **Warren** | 68.1 (67.4, 68.6) | 51 (48, 51) | 68.4 (67.7, 68.8) | 51 (51, 51) | 75.1 (74.5, 75.6) | 37 (34, 41) | 75.4 (74.7, 75.9) | 42 (39, 46) |
| **Washington** | 68.9 (68.3, 69.3) | 47 (44, 51) | 70.1 (69.6, 70.6) | 39 (36, 47) | 76.8 (76.2, 77.2) | 28 (26, 30) | 77 (76.3, 77.5) | 34 (32, 37) |
| **Wayne** | 71.2 (70.6, 71.6) | 26 (25, 29) | 70.9 (70.3, 71.4) | 34 (32, 38) | 77.7 (77.2, 78.2) | 24 (22, 26) | 77.3 (76.6, 77.8) | 33 (31, 35) |
| **Webster** | 69.1 (68.4, 69.5) | 46 (42, 50) | 69.6 (69, 70.1) | 46 (40, 51) | 76.6 (76.1, 77) | 28 (27, 31) | 75.8 (75.1, 76.2) | 40 (37, 44) |
| **Wheeler** | 70.1 (69.5, 70.5) | 33 (30, 41) | 71.3 (70.7, 71.7) | 33 (31, 36) | 77.4 (76.8, 77.8) | 25 (24, 28) | 76.5 (75.8, 76.9) | 36 (34, 39) |
| **White** | 73.6 (72.9, 74.1) | 15 (13, 19) | 74.6 (73.8, 75.2) | 18 (15, 22) | 79.8 (79.1, 80.5) | 15 (11, 19) | 79.7 (78.9, 80.3) | 23 (19, 26) |
| **Whitfield** | 72.1 (71.6, 72.5) | 22 (21, 24) | 72.5 (71.9, 72.8) | 28 (26, 30) | 78.5 (78, 78.9) | 21 (19, 23) | 78.6 (78, 79) | 28 (26, 30) |
| **Wilcox** | 70.8 (70.2, 71.2) | 28 (26, 32) | 70.9 (70.3, 71.3) | 35 (33, 38) | 77 (76.4, 77.4) | 27 (25, 29) | 77.1 (76.4, 77.6) | 33 (32, 36) |
| **Wilkes** | 69.6 (69, 70) | 40 (34, 46) | 70.8 (70.2, 71.3) | 35 (33, 39) | 76.7 (76.2, 77.1) | 28 (26, 30) | 76.8 (76.2, 77.2) | 35 (33, 37) |
| **Wilkinson** | 69.9 (69.2, 70.3) | 35 (31, 44) | 70.6 (70, 71) | 36 (34, 41) | 76.9 (76.3, 77.3) | 27 (26, 30) | 76.1 (75.5, 76.6) | 38 (35, 41) |
| **Worth** | 70.7 (70.1, 71.1) | 29 (27, 33) | 71.8 (71.1, 72.2) | 31 (29, 33) | 78.2 (77.6, 78.7) | 22 (21, 24) | 78.3 (77.7, 78.9) | 29 (27, 31) |
| **HAWAII** |  |  |  |  |  |  |  |  |
| **Hawaii** | 75.2 (74.9, 75.5) | 8 (7, 9) | 76.6 (76.3, 76.9) | 9 (8, 10) | 81.2 (80.9, 81.5) | 7 (6, 9) | 82.9 (82.5, 83.2) | 7 (5, 8) |
| **Honolulu** | 77.5 (77.3, 77.6) | -1 (-2, -1) | 78.4 (78.3, 78.6) | 2 (2, 3) | 82.9 (82.7, 83.1) | -1 (-1, 0) | 84.3 (84.1, 84.5) | -2 (-3, 0) |
| **Kalawao** | 75.2 (74.9, 75.5) | 8 (7, 9) | 76.6 (76.3, 76.9) | 9 (8, 10) | 81.2 (80.9, 81.5) | 7 (6, 9) | 82.9 (82.5, 83.2) | 7 (5, 8) |
| **Kauai** | 76.4 (76, 76.8) | 3 (1, 5) | 76.6 (76.1, 77) | 9 (8, 11) | 82.9 (82.3, 83.4) | -1 (-3, 2) | 83.5 (82.9, 83.9) | 4 (1, 6) |
| **Maui** | 77.1 (76.8, 77.4) | 0 (-1, 1) | 77.1 (76.7, 77.4) | 7 (6, 9) | 82.5 (82.1, 82.9) | 1 (-1, 3) | 83.1 (82.7, 83.5) | 5 (3, 7) |
| **IDAHO** |  |  |  |  |  |  |  |  |
| **Ada** | 76.5 (76.2, 76.8) | 2 (1, 4) | 77.8 (77.4, 78.1) | 5 (4, 6) | 81.6 (81.2, 81.9) | 5 (4, 7) | 81.9 (81.6, 82.2) | 11 (9, 12) |
| **Adams** | 74.9 (74.2, 75.4) | 9 (7, 13) | 76.7 (75.8, 77.4) | 9 (6, 12) | 80.3 (79.6, 80.9) | 12 (9, 16) | 81 (80.2, 81.8) | 15 (11, 20) |
| **Bannock** | 74.4 (73.9, 74.8) | 12 (10, 14) | 75.9 (75.3, 76.3) | 12 (10, 14) | 80 (79.4, 80.6) | 14 (11, 17) | 80.3 (79.6, 80.8) | 19 (16, 23) |
| **Bear Lake** | 74.6 (73.9, 75.1) | 11 (8, 14) | 76.2 (75.5, 76.8) | 10 (8, 13) | 80.4 (79.8, 81.1) | 11 (8, 15) | 80.9 (80.2, 81.6) | 16 (12, 20) |
| **Benewah** | 72.9 (72.1, 73.4) | 19 (17, 22) | 74.6 (73.9, 75.2) | 18 (15, 21) | 78.8 (78.2, 79.4) | 20 (17, 22) | 80.2 (79.4, 80.9) | 20 (16, 24) |
| **Bingham** | 74.1 (73.5, 74.6) | 13 (11, 16) | 75.4 (74.7, 75.9) | 14 (12, 17) | 80.1 (79.4, 80.6) | 14 (10, 17) | 80.4 (79.7, 81.1) | 18 (15, 23) |
| **Blaine** | 77.4 (76.6, 78) | -1 (-3, 2) | 79.3 (78.4, 80.1) | -2 (-8, 2) | 81.3 (80.5, 82) | 7 (3, 11) | 83.6 (82.6, 84.6) | 3 (-4, 8) |
| **Boise** | 75 (74.2, 75.5) | 9 (6, 13) | 75.8 (75, 76.4) | 12 (10, 16) | 80.1 (79.4, 80.8) | 13 (10, 17) | 80.4 (79.6, 81.1) | 18 (15, 23) |
| **Bonner** | 74.9 (74.2, 75.4) | 9 (7, 13) | 76.1 (75.4, 76.7) | 11 (9, 14) | 80.1 (79.5, 80.7) | 13 (10, 17) | 80.7 (80.1, 81.3) | 17 (14, 21) |
| **Bonneville** | 75.4 (74.9, 75.8) | 7 (5, 9) | 76.4 (75.9, 76.8) | 10 (8, 12) | 80.6 (80, 81.1) | 11 (8, 14) | 80 (79.5, 80.4) | 21 (19, 24) |
| **Boundary** | 74.9 (74.2, 75.4) | 9 (7, 13) | 76.1 (75.4, 76.7) | 11 (9, 14) | 80.1 (79.5, 80.7) | 13 (10, 17) | 80.7 (80.1, 81.3) | 17 (14, 21) |
| **Butte** | 74.3 (73.7, 74.8) | 12 (10, 15) | 76.2 (75.4, 76.8) | 11 (8, 14) | 79.6 (79, 80.2) | 16 (13, 19) | 80.4 (79.6, 81.1) | 18 (15, 23) |
| **Camas** | 74.3 (73.5, 74.8) | 12 (10, 16) | 74.3 (73.4, 75) | 19 (16, 23) | 79.9 (79.2, 80.5) | 15 (11, 18) | 80 (79.1, 80.7) | 21 (17, 26) |
| **Canyon** | 74.4 (74, 74.7) | 12 (10, 14) | 75.1 (74.7, 75.5) | 15 (14, 17) | 80 (79.5, 80.4) | 14 (12, 17) | 80.3 (79.9, 80.7) | 19 (17, 22) |
| **Caribou** | 74.6 (73.9, 75.1) | 11 (8, 14) | 76.2 (75.5, 76.8) | 10 (8, 13) | 80.4 (79.8, 81.1) | 11 (8, 15) | 80.9 (80.2, 81.6) | 16 (12, 20) |
| **Cassia** | 74.4 (73.7, 74.9) | 12 (9, 15) | 75.6 (74.9, 76.2) | 13 (11, 17) | 80.2 (79.5, 80.8) | 13 (9, 17) | 80.5 (79.7, 81.2) | 18 (14, 23) |
| **Clark** | 74.3 (73.7, 74.8) | 12 (10, 15) | 76.2 (75.4, 76.8) | 11 (8, 14) | 79.6 (79, 80.2) | 16 (13, 19) | 80.4 (79.6, 81.1) | 18 (15, 23) |
| **Clearwater** | 74.8 (74, 75.3) | 10 (7, 13) | 75.8 (75, 76.4) | 12 (10, 16) | 80 (79.3, 80.6) | 14 (10, 18) | 80.6 (79.8, 81.3) | 18 (14, 22) |
| **Custer** | 75 (74.2, 75.5) | 9 (6, 13) | 75.8 (75, 76.4) | 12 (10, 16) | 80.1 (79.4, 80.8) | 13 (10, 17) | 80.4 (79.6, 81.1) | 18 (15, 23) |
| **Elmore** | 74.5 (73.8, 75) | 11 (9, 15) | 75.5 (74.8, 76) | 14 (11, 17) | 79.8 (79, 80.4) | 15 (12, 19) | 79.9 (79.2, 80.5) | 22 (18, 25) |
| **Franklin** | 74.6 (73.9, 75.1) | 11 (8, 14) | 76.2 (75.5, 76.8) | 10 (8, 13) | 80.4 (79.8, 81.1) | 11 (8, 15) | 80.9 (80.2, 81.6) | 16 (12, 20) |
| **Fremont** | 74.3 (73.7, 74.8) | 12 (10, 15) | 76.2 (75.4, 76.8) | 11 (8, 14) | 79.6 (79, 80.2) | 16 (13, 19) | 80.4 (79.6, 81.1) | 18 (15, 23) |
| **Gem** | 75 (74.2, 75.5) | 9 (6, 13) | 75.8 (75, 76.4) | 12 (10, 16) | 80.1 (79.4, 80.8) | 13 (10, 17) | 80.4 (79.6, 81.1) | 18 (15, 23) |
| **Gooding** | 74.3 (73.5, 74.8) | 12 (10, 16) | 74.3 (73.4, 75) | 19 (16, 23) | 79.9 (79.2, 80.5) | 15 (11, 18) | 80 (79.1, 80.7) | 21 (17, 26) |
| **Idaho** | 74.8 (74, 75.3) | 10 (7, 13) | 75.8 (75, 76.4) | 12 (10, 16) | 80 (79.3, 80.6) | 14 (10, 18) | 80.6 (79.8, 81.3) | 18 (14, 22) |
| **Jefferson** | 74.1 (73.3, 74.6) | 13 (11, 17) | 75.4 (74.6, 76) | 14 (11, 18) | 80.6 (79.7, 81.3) | 11 (7, 16) | 80.4 (79.5, 81.2) | 18 (14, 24) |
| **Jerome** | 73.8 (73, 74.4) | 15 (12, 18) | 74.7 (73.9, 75.4) | 17 (14, 21) | 79.8 (79.1, 80.5) | 15 (11, 19) | 80.3 (79.4, 81.1) | 19 (15, 24) |
| **Kootenai** | 75.8 (75.3, 76.2) | 5 (4, 7) | 77.5 (77, 77.9) | 6 (4, 8) | 80.5 (80, 81) | 11 (8, 14) | 81.8 (81.3, 82.2) | 11 (9, 14) |
| **Latah** | 76.4 (75.8, 76.9) | 3 (1, 5) | 77.6 (76.9, 78.2) | 6 (3, 8) | 81 (80.3, 81.6) | 8 (5, 12) | 81.6 (80.8, 82.3) | 12 (9, 16) |
| **Lemhi** | 74.3 (73.7, 74.8) | 12 (10, 15) | 76.2 (75.4, 76.8) | 11 (8, 14) | 79.6 (79, 80.2) | 16 (13, 19) | 80.4 (79.6, 81.1) | 18 (15, 23) |
| **Lewis** | 74.8 (74, 75.3) | 10 (7, 13) | 75.8 (75, 76.4) | 12 (10, 16) | 80 (79.3, 80.6) | 14 (10, 18) | 80.6 (79.8, 81.3) | 18 (14, 22) |
| **Lincoln** | 74.3 (73.5, 74.8) | 12 (10, 16) | 74.3 (73.4, 75) | 19 (16, 23) | 79.9 (79.2, 80.5) | 15 (11, 18) | 80 (79.1, 80.7) | 21 (17, 26) |
| **Madison** | 74.9 (74.2, 75.5) | 9 (7, 13) | 76.7 (75.9, 77.2) | 9 (7, 12) | 80.1 (79.4, 80.8) | 13 (9, 17) | 80.4 (79.5, 81.1) | 19 (15, 24) |
| **Minidoka** | 73.2 (72.3, 73.7) | 18 (15, 21) | 74.2 (73.3, 74.8) | 20 (17, 24) | 79.5 (78.8, 80.1) | 17 (13, 20) | 79.6 (78.7, 80.2) | 23 (20, 27) |
| **Nez Perce** | 75.4 (74.8, 75.9) | 7 (5, 10) | 76.1 (75.5, 76.6) | 11 (9, 13) | 80.8 (80.2, 81.3) | 9 (6, 13) | 80.7 (80, 81.2) | 17 (14, 21) |
| **Oneida** | 74.4 (73.7, 74.9) | 12 (9, 15) | 75.6 (74.9, 76.2) | 13 (11, 17) | 80.2 (79.5, 80.8) | 13 (9, 17) | 80.5 (79.7, 81.2) | 18 (14, 23) |
| **Owyhee** | 74.5 (73.8, 75) | 11 (9, 15) | 75.5 (74.8, 76) | 14 (11, 17) | 79.8 (79, 80.4) | 15 (12, 19) | 79.9 (79.2, 80.5) | 22 (18, 25) |
| **Payette** | 73.5 (72.8, 74.1) | 16 (13, 19) | 75 (74.2, 75.6) | 16 (13, 20) | 79.2 (78.5, 79.8) | 18 (15, 21) | 79.6 (78.8, 80.4) | 23 (19, 27) |
| **Power** | 74.4 (73.7, 74.9) | 12 (9, 15) | 75.6 (74.9, 76.2) | 13 (11, 17) | 80.2 (79.5, 80.8) | 13 (9, 17) | 80.5 (79.7, 81.2) | 18 (14, 23) |
| **Shoshone** | 72.9 (72.1, 73.4) | 19 (17, 22) | 74.6 (73.9, 75.2) | 18 (15, 21) | 78.8 (78.2, 79.4) | 20 (17, 22) | 80.2 (79.4, 80.9) | 20 (16, 24) |
| **Teton** | 74.9 (74.2, 75.5) | 9 (7, 13) | 76.7 (75.9, 77.2) | 9 (7, 12) | 80.1 (79.4, 80.8) | 13 (9, 17) | 80.4 (79.5, 81.1) | 19 (15, 24) |
| **Twin Falls** | 74.7 (74.2, 75.1) | 10 (8, 13) | 75.4 (74.8, 75.8) | 14 (12, 17) | 80.1 (79.6, 80.6) | 13 (11, 16) | 79.9 (79.4, 80.4) | 22 (19, 24) |
| **Valley** | 74.9 (74.2, 75.4) | 9 (7, 13) | 76.7 (75.8, 77.4) | 9 (6, 12) | 80.3 (79.6, 80.9) | 12 (9, 16) | 81 (80.2, 81.8) | 15 (11, 20) |
| **Washington** | 74.9 (74.2, 75.4) | 9 (7, 13) | 76.7 (75.8, 77.4) | 9 (6, 12) | 80.3 (79.6, 80.9) | 12 (9, 16) | 81 (80.2, 81.8) | 15 (11, 20) |
| **ILLINOIS** |  |  |  |  |  |  |  |  |
| **Adams** | 74.2 (73.7, 74.5) | 13 (11, 15) | 74.4 (73.9, 74.8) | 19 (17, 21) | 80.9 (80.4, 81.4) | 9 (6, 12) | 80.7 (80.1, 81.2) | 17 (14, 20) |
| **Alexander** | 69.6 (68.9, 70) | 41 (35, 47) | 69.9 (69.2, 70.4) | 42 (38, 51) | 77 (76.4, 77.5) | 27 (25, 29) | 77 (76.4, 77.5) | 34 (32, 36) |
| **Bond** | 73.9 (73.1, 74.3) | 14 (12, 18) | 74.1 (73.3, 74.6) | 20 (18, 24) | 79.6 (78.9, 80.2) | 16 (13, 20) | 79.5 (78.7, 80.2) | 24 (20, 27) |
| **Boone** | 75 (74.4, 75.4) | 9 (7, 12) | 76.5 (75.9, 77) | 10 (8, 12) | 80.6 (79.9, 81.1) | 11 (8, 15) | 80.6 (79.9, 81.2) | 18 (14, 22) |
| **Brown** | 74 (73.4, 74.4) | 14 (12, 16) | 74.4 (73.7, 75) | 19 (16, 22) | 79.9 (79.3, 80.5) | 15 (11, 18) | 79.5 (78.8, 80.1) | 24 (21, 27) |
| **Bureau** | 75.1 (74.5, 75.5) | 8 (6, 11) | 75.9 (75.2, 76.4) | 12 (10, 15) | 81 (80.4, 81.6) | 8 (5, 11) | 80.7 (80, 81.3) | 17 (14, 21) |
| **Calhoun** | 73.4 (72.7, 74) | 16 (14, 20) | 74.5 (73.8, 75.1) | 18 (15, 21) | 79.5 (78.9, 80.1) | 17 (13, 20) | 79.5 (78.7, 80.1) | 24 (20, 27) |
| **Carroll** | 75 (74.2, 75.5) | 9 (7, 13) | 75.6 (74.8, 76.2) | 13 (11, 17) | 79.7 (79, 80.2) | 16 (13, 19) | 81.1 (80.2, 81.7) | 15 (12, 20) |
| **Cass** | 74 (73.3, 74.5) | 14 (11, 17) | 74.8 (74, 75.4) | 17 (14, 20) | 80.3 (79.6, 80.8) | 13 (9, 16) | 80 (79.3, 80.6) | 21 (17, 25) |
| **Champaign** | 76.2 (75.8, 76.5) | 4 (2, 5) | 76.6 (76.2, 77) | 9 (8, 10) | 80.3 (79.9, 80.7) | 12 (10, 14) | 80.8 (80.4, 81.1) | 17 (15, 19) |
| **Christian** | 73.9 (73.2, 74.3) | 14 (12, 17) | 73.6 (72.9, 74.1) | 23 (20, 26) | 79.7 (79.1, 80.2) | 16 (13, 19) | 80.2 (79.6, 80.8) | 20 (16, 23) |
| **Clark** | 73.9 (73.2, 74.5) | 14 (11, 17) | 74.5 (73.6, 75.2) | 18 (15, 22) | 79.8 (79.1, 80.4) | 15 (12, 19) | 80.5 (79.7, 81.3) | 18 (14, 23) |
| **Clay** | 73.7 (73.1, 74.2) | 15 (12, 18) | 74.4 (73.7, 74.9) | 19 (16, 22) | 79.7 (79, 80.2) | 16 (13, 19) | 80 (79.3, 80.6) | 21 (17, 25) |
| **Clinton** | 73.7 (73.1, 74.2) | 15 (13, 18) | 74.9 (74.3, 75.4) | 16 (14, 19) | 80.7 (80.1, 81.3) | 10 (7, 14) | 81.2 (80.5, 81.9) | 14 (11, 18) |
| **Coles** | 74.3 (73.8, 74.8) | 12 (10, 15) | 74.7 (74.1, 75.3) | 17 (15, 20) | 79.7 (79.1, 80.2) | 16 (13, 19) | 80.1 (79.5, 80.7) | 21 (17, 24) |
| **Cook** | 72.5 (72.5, 72.6) | 21 (20, 21) | 75.1 (75, 75.2) | 15 (15, 16) | 78.7 (78.6, 78.8) | 20 (20, 21) | 80.7 (80.7, 80.8) | 17 (16, 17) |
| **Crawford** | 73.8 (73, 74.3) | 15 (12, 18) | 74.9 (74.1, 75.4) | 17 (14, 20) | 79.6 (78.9, 80.1) | 16 (13, 19) | 79.6 (78.8, 80.2) | 23 (20, 27) |
| **Cumberland** | 74.5 (73.8, 75) | 11 (9, 14) | 75.3 (74.5, 76) | 14 (12, 18) | 80.4 (79.7, 81) | 12 (8, 16) | 80.2 (79.5, 80.9) | 20 (16, 24) |
| **De Kalb** | 75.7 (75.2, 76.1) | 6 (4, 8) | 77 (76.5, 77.5) | 8 (6, 10) | 80.2 (79.6, 80.6) | 13 (11, 16) | 81 (80.4, 81.5) | 15 (13, 19) |
| **De Witt** | 74.3 (73.6, 74.9) | 12 (9, 15) | 73.9 (73.1, 74.6) | 21 (18, 25) | 80.3 (79.6, 80.9) | 12 (9, 16) | 79.6 (78.8, 80.3) | 23 (19, 27) |
| **Douglas** | 73.9 (73.1, 74.4) | 14 (12, 18) | 74.6 (73.8, 75.2) | 18 (15, 21) | 80.4 (79.7, 81.1) | 12 (8, 16) | 80.3 (79.5, 81) | 19 (15, 24) |
| **Du Page** | 77.3 (77.1, 77.5) | 0 (-1, 0) | 79 (78.8, 79.2) | -1 (-2, 0) | 81.3 (81.1, 81.5) | 7 (6, 8) | 82.4 (82.2, 82.6) | 9 (8, 9) |
| **Edgar** | 73.6 (72.9, 74.2) | 15 (13, 19) | 73.8 (73, 74.4) | 21 (19, 25) | 79.9 (79.2, 80.5) | 15 (11, 18) | 79.7 (78.9, 80.4) | 23 (19, 26) |
| **Edwards** | 73.8 (73.1, 74.3) | 14 (12, 18) | 75.2 (74.4, 75.8) | 15 (12, 19) | 80.3 (79.7, 80.9) | 12 (9, 16) | 80.1 (79.3, 80.7) | 20 (17, 25) |
| **Effingham** | 74.5 (73.9, 75) | 11 (9, 14) | 75.3 (74.5, 75.8) | 15 (12, 18) | 80.3 (79.7, 80.8) | 12 (9, 16) | 80.7 (79.9, 81.3) | 17 (14, 21) |
| **Fayette** | 73.1 (72.4, 73.6) | 18 (15, 21) | 73.6 (72.9, 74.2) | 22 (20, 26) | 79.8 (79.1, 80.4) | 15 (12, 19) | 79.2 (78.4, 79.8) | 25 (22, 28) |
| **Ford** | 74.4 (73.8, 74.9) | 12 (10, 14) | 74.6 (73.9, 75) | 18 (16, 21) | 80.1 (79.5, 80.6) | 14 (11, 17) | 79.5 (78.9, 79.9) | 24 (21, 27) |
| **Franklin** | 72.1 (71.5, 72.6) | 22 (20, 25) | 72.2 (71.4, 72.7) | 29 (27, 32) | 78.8 (78.2, 79.3) | 20 (18, 22) | 78.6 (77.9, 79.2) | 28 (25, 30) |
| **Fulton** | 74.3 (73.7, 74.8) | 12 (10, 15) | 73.9 (73.3, 74.4) | 21 (19, 24) | 79.6 (79.1, 80.2) | 16 (13, 19) | 79.8 (79.1, 80.4) | 22 (19, 26) |
| **Gallatin** | 72.4 (71.7, 72.8) | 21 (19, 24) | 73 (72.3, 73.6) | 25 (22, 29) | 79.2 (78.6, 79.8) | 18 (15, 21) | 79.5 (78.7, 80.2) | 24 (20, 27) |
| **Greene** | 73.4 (72.7, 74) | 16 (14, 20) | 74.5 (73.8, 75.1) | 18 (15, 21) | 79.5 (78.9, 80.1) | 17 (13, 20) | 79.5 (78.7, 80.1) | 24 (20, 27) |
| **Grundy** | 75 (74.4, 75.5) | 9 (6, 12) | 75.8 (75.1, 76.3) | 12 (10, 15) | 80.3 (79.6, 80.8) | 13 (9, 16) | 80.5 (79.9, 81.1) | 18 (15, 22) |
| **Hamilton** | 72.4 (71.7, 72.8) | 21 (19, 24) | 73 (72.3, 73.6) | 25 (22, 29) | 79.2 (78.6, 79.8) | 18 (15, 21) | 79.5 (78.7, 80.2) | 24 (20, 27) |
| **Hancock** | 74.7 (73.9, 75.3) | 10 (8, 14) | 76 (75.1, 76.6) | 11 (9, 15) | 80.7 (79.9, 81.3) | 10 (7, 14) | 81.2 (80.3, 82) | 14 (10, 19) |
| **Hardin** | 72.4 (71.7, 72.8) | 21 (19, 24) | 73 (72.3, 73.6) | 25 (22, 29) | 79.2 (78.6, 79.8) | 18 (15, 21) | 79.5 (78.7, 80.2) | 24 (20, 27) |
| **Henderson** | 74.3 (73.6, 74.8) | 12 (10, 15) | 75.9 (75.1, 76.5) | 12 (9, 16) | 80.4 (79.7, 80.9) | 12 (9, 16) | 80.9 (80.1, 81.5) | 16 (13, 20) |
| **Henry** | 75 (74.5, 75.5) | 9 (6, 11) | 75.9 (75.4, 76.4) | 12 (10, 14) | 80.7 (80.1, 81.2) | 10 (7, 13) | 81 (80.4, 81.6) | 15 (12, 19) |
| **Iroquois** | 74.4 (73.8, 74.9) | 12 (10, 14) | 74.6 (73.9, 75) | 18 (16, 21) | 80.1 (79.5, 80.6) | 14 (11, 17) | 79.5 (78.9, 79.9) | 24 (21, 27) |
| **Jackson** | 73.8 (73.3, 74.2) | 15 (13, 17) | 75.3 (74.7, 75.8) | 14 (12, 17) | 79.4 (78.9, 79.8) | 17 (15, 20) | 80.1 (79.5, 80.6) | 21 (17, 24) |
| **Jasper** | 74.5 (73.8, 75) | 11 (9, 14) | 75.3 (74.5, 76) | 14 (12, 18) | 80.4 (79.7, 81) | 12 (8, 16) | 80.2 (79.5, 80.9) | 20 (16, 24) |
| **Jefferson** | 73.8 (73.2, 74.2) | 15 (12, 17) | 74.3 (73.7, 74.8) | 19 (17, 22) | 79.9 (79.3, 80.4) | 15 (12, 18) | 79.7 (79.1, 80.2) | 23 (20, 26) |
| **Jersey** | 74.4 (73.7, 75) | 12 (9, 15) | 75.3 (74.6, 75.9) | 14 (12, 18) | 79.6 (78.9, 80.2) | 16 (13, 20) | 79.8 (79.1, 80.5) | 22 (18, 26) |
| **Jo Daviess** | 75.3 (74.6, 75.8) | 8 (5, 11) | 76.5 (75.7, 77.2) | 9 (7, 13) | 80.9 (80.2, 81.5) | 9 (6, 13) | 81.6 (80.8, 82.3) | 12 (9, 16) |
| **Johnson** | 72.3 (71.6, 72.7) | 22 (20, 24) | 72.8 (72.1, 73.3) | 26 (24, 29) | 78.5 (77.9, 79) | 21 (19, 23) | 78.8 (78.1, 79.3) | 27 (25, 30) |
| **Kane** | 75.3 (75, 75.5) | 8 (6, 9) | 77.4 (77.2, 77.7) | 6 (5, 7) | 79.7 (79.4, 80) | 16 (14, 17) | 81 (80.7, 81.3) | 15 (14, 17) |
| **Kankakee** | 71.6 (71.2, 71.9) | 25 (23, 26) | 73.3 (72.9, 73.6) | 24 (22, 26) | 78.5 (78.1, 78.8) | 21 (20, 23) | 79 (78.6, 79.4) | 26 (24, 28) |
| **Kendall** | 76 (75.4, 76.5) | 4 (2, 7) | 77.4 (76.8, 77.9) | 6 (4, 8) | 80.2 (79.6, 80.8) | 13 (10, 16) | 81.9 (81.2, 82.5) | 11 (8, 14) |
| **Knox** | 74.5 (74, 74.9) | 11 (9, 13) | 74.2 (73.7, 74.6) | 20 (18, 22) | 80.1 (79.6, 80.5) | 14 (11, 16) | 79.3 (78.8, 79.8) | 25 (22, 27) |
| **La Salle** | 74.6 (74.1, 74.9) | 11 (9, 13) | 74.4 (74, 74.7) | 19 (17, 21) | 80.3 (79.9, 80.7) | 12 (10, 14) | 80.1 (79.6, 80.4) | 21 (18, 23) |
| **Lake** | 76.4 (76.2, 76.6) | 3 (2, 4) | 78.5 (78.3, 78.7) | 2 (1, 3) | 80.3 (80.1, 80.5) | 12 (11, 13) | 81.7 (81.5, 82) | 12 (11, 13) |
| **Lawrence** | 73 (72.2, 73.6) | 18 (15, 22) | 73.7 (72.9, 74.4) | 22 (19, 26) | 79.4 (78.7, 80) | 17 (14, 20) | 79.3 (78.5, 80) | 25 (21, 28) |
| **Lee** | 73.2 (72.6, 73.6) | 17 (15, 20) | 74.8 (74.2, 75.3) | 17 (14, 20) | 79.8 (79.2, 80.3) | 15 (12, 18) | 79.9 (79.1, 80.5) | 22 (18, 26) |
| **Livingston** | 73.9 (73.3, 74.4) | 14 (12, 17) | 74 (73.4, 74.5) | 20 (18, 23) | 80.1 (79.5, 80.6) | 13 (10, 17) | 79.3 (78.6, 79.8) | 25 (22, 28) |
| **Logan** | 74 (73.3, 74.4) | 14 (12, 17) | 74 (73.4, 74.6) | 20 (18, 23) | 79.8 (79.2, 80.4) | 15 (12, 18) | 79.8 (79.2, 80.4) | 22 (19, 25) |
| **Macon** | 73.5 (73.1, 73.9) | 16 (14, 18) | 74 (73.6, 74.3) | 21 (19, 23) | 79.6 (79.3, 80) | 16 (14, 18) | 80.1 (79.7, 80.5) | 20 (18, 23) |
| **Macoupin** | 74.1 (73.5, 74.5) | 13 (11, 16) | 74.6 (74, 75) | 18 (16, 21) | 79.4 (78.9, 79.9) | 17 (15, 19) | 80.2 (79.6, 80.7) | 20 (17, 23) |
| **Madison** | 73.2 (73, 73.5) | 17 (16, 19) | 74.5 (74.2, 74.7) | 18 (17, 20) | 78.5 (78.3, 78.8) | 21 (20, 22) | 79.5 (79.2, 79.8) | 24 (22, 25) |
| **Marion** | 72.9 (72.3, 73.3) | 19 (17, 22) | 73.6 (72.9, 74.1) | 22 (20, 26) | 79.6 (79.1, 80.1) | 16 (13, 19) | 79.5 (78.9, 80.1) | 24 (21, 27) |
| **Marshall** | 75 (74.3, 75.5) | 9 (6, 12) | 74.9 (74.1, 75.4) | 17 (14, 20) | 80.1 (79.5, 80.7) | 14 (10, 17) | 80.3 (79.6, 81) | 19 (15, 23) |
| **Mason** | 74 (73.4, 74.4) | 14 (12, 16) | 74.4 (73.7, 75) | 19 (16, 22) | 79.9 (79.3, 80.5) | 15 (11, 18) | 79.5 (78.8, 80.1) | 24 (21, 27) |
| **Massac** | 72.3 (71.6, 72.7) | 22 (20, 24) | 72.8 (72.1, 73.3) | 26 (24, 29) | 78.5 (77.9, 79) | 21 (19, 23) | 78.8 (78.1, 79.3) | 27 (25, 30) |
| **McDonough** | 75.3 (74.7, 75.8) | 7 (5, 10) | 76.1 (75.4, 76.7) | 11 (9, 14) | 80.4 (79.8, 80.9) | 12 (9, 15) | 80.5 (79.8, 81.1) | 18 (15, 22) |
| **McHenry** | 75.6 (75.3, 75.9) | 6 (5, 8) | 77.7 (77.3, 78) | 5 (4, 7) | 80.3 (79.9, 80.6) | 12 (11, 15) | 81.6 (81.2, 81.9) | 12 (11, 14) |
| **McLean** | 76.3 (75.9, 76.6) | 3 (2, 5) | 76.6 (76.1, 76.9) | 9 (8, 11) | 80.5 (80.1, 80.9) | 11 (9, 14) | 80.9 (80.5, 81.3) | 16 (14, 18) |
| **Menard** | 74 (73.3, 74.5) | 14 (11, 17) | 74.8 (74, 75.4) | 17 (14, 20) | 80.3 (79.6, 80.8) | 13 (9, 16) | 80 (79.3, 80.6) | 21 (17, 25) |
| **Mercer** | 74.3 (73.6, 74.8) | 12 (10, 15) | 75.9 (75.1, 76.5) | 12 (9, 16) | 80.4 (79.7, 80.9) | 12 (9, 16) | 80.9 (80.1, 81.5) | 16 (13, 20) |
| **Monroe** | 75.8 (75.1, 76.4) | 5 (3, 8) | 76.8 (76.1, 77.4) | 8 (6, 11) | 80.9 (80.2, 81.5) | 9 (6, 13) | 80.7 (80, 81.4) | 17 (13, 21) |
| **Montgomery** | 73.1 (72.5, 73.5) | 18 (16, 21) | 74.1 (73.4, 74.6) | 20 (18, 23) | 79.5 (78.9, 79.9) | 17 (14, 20) | 79.8 (79.1, 80.3) | 22 (19, 26) |
| **Morgan** | 74 (73.4, 74.5) | 13 (11, 16) | 74.6 (73.9, 75.1) | 18 (16, 21) | 79.7 (79.1, 80.2) | 16 (13, 19) | 79.5 (78.8, 80) | 24 (21, 27) |
| **Moultrie** | 74.6 (73.9, 75.1) | 11 (9, 14) | 75.1 (74.4, 75.6) | 15 (13, 19) | 80.2 (79.7, 80.8) | 13 (9, 16) | 81 (80.3, 81.7) | 15 (12, 19) |
| **Ogle** | 75.4 (74.9, 75.9) | 7 (5, 9) | 75.8 (75.2, 76.3) | 12 (10, 15) | 80.4 (79.9, 81) | 11 (8, 15) | 80.5 (79.9, 81) | 18 (15, 22) |
| **Peoria** | 74.1 (73.8, 74.3) | 13 (12, 15) | 74.5 (74.2, 74.8) | 18 (17, 20) | 79.7 (79.4, 80) | 16 (14, 17) | 79.9 (79.6, 80.2) | 21 (20, 23) |
| **Perry** | 72.6 (71.9, 73.1) | 21 (18, 23) | 73.4 (72.8, 73.9) | 23 (21, 27) | 79 (78.4, 79.6) | 19 (16, 21) | 79.1 (78.4, 79.8) | 26 (22, 29) |
| **Piatt** | 74.6 (73.9, 75.1) | 11 (9, 14) | 75.1 (74.4, 75.6) | 15 (13, 19) | 80.2 (79.7, 80.8) | 13 (9, 16) | 81 (80.3, 81.7) | 15 (12, 19) |
| **Pike** | 74 (73.3, 74.5) | 14 (11, 17) | 75 (74.3, 75.6) | 16 (13, 19) | 79.5 (78.9, 80) | 17 (14, 20) | 80.5 (79.6, 81.1) | 18 (15, 23) |
| **Pope** | 71.9 (71.3, 72.3) | 23 (21, 26) | 72.4 (71.7, 72.9) | 28 (26, 31) | 78.8 (78.2, 79.2) | 20 (18, 22) | 78.7 (78.1, 79.2) | 27 (25, 30) |
| **Pulaski** | 69.6 (68.9, 70) | 41 (35, 47) | 69.9 (69.2, 70.4) | 42 (38, 51) | 77 (76.4, 77.5) | 27 (25, 29) | 77 (76.4, 77.5) | 34 (32, 36) |
| **Putnam** | 75.1 (74.5, 75.5) | 8 (6, 11) | 75.9 (75.2, 76.4) | 12 (10, 15) | 81 (80.4, 81.6) | 8 (5, 11) | 80.7 (80, 81.3) | 17 (14, 21) |
| **Randolph** | 72.7 (72.1, 73.1) | 20 (18, 22) | 73.8 (73.1, 74.3) | 22 (19, 25) | 79.3 (78.6, 79.8) | 18 (15, 21) | 79.2 (78.5, 79.8) | 25 (22, 28) |
| **Richland** | 73.7 (73.1, 74.2) | 15 (12, 18) | 74.4 (73.7, 74.9) | 19 (16, 22) | 79.7 (79, 80.2) | 16 (13, 19) | 80 (79.3, 80.6) | 21 (17, 25) |
| **Rock Island** | 74.1 (73.7, 74.4) | 13 (12, 15) | 75.7 (75.3, 76) | 13 (11, 14) | 79.7 (79.3, 80) | 16 (14, 18) | 81 (80.6, 81.3) | 16 (14, 18) |
| **Saline** | 71.9 (71.3, 72.3) | 23 (21, 26) | 72.4 (71.7, 72.9) | 28 (26, 31) | 78.8 (78.2, 79.2) | 20 (18, 22) | 78.7 (78.1, 79.2) | 27 (25, 30) |
| **Sangamon** | 74 (73.7, 74.3) | 13 (12, 15) | 74.8 (74.4, 75.1) | 17 (16, 19) | 79.9 (79.6, 80.2) | 15 (13, 16) | 80 (79.7, 80.4) | 21 (19, 23) |
| **Schuyler** | 74 (73.4, 74.4) | 14 (12, 16) | 74.4 (73.7, 75) | 19 (16, 22) | 79.9 (79.3, 80.5) | 15 (11, 18) | 79.5 (78.8, 80.1) | 24 (21, 27) |
| **Scott** | 74 (73.3, 74.5) | 14 (11, 17) | 75 (74.3, 75.6) | 16 (13, 19) | 79.5 (78.9, 80) | 17 (14, 20) | 80.5 (79.6, 81.1) | 18 (15, 23) |
| **Shelby** | 74.7 (74, 75.2) | 11 (8, 14) | 75.9 (75.1, 76.5) | 12 (9, 15) | 80.7 (79.9, 81.3) | 10 (7, 14) | 80.5 (79.7, 81.2) | 18 (14, 22) |
| **St. Clair** | 71.8 (71.5, 72) | 24 (23, 25) | 72.2 (71.9, 72.4) | 29 (28, 30) | 77.7 (77.4, 77.9) | 24 (24, 25) | 78.5 (78.3, 78.8) | 28 (27, 29) |
| **Stark** | 75 (74.3, 75.5) | 9 (6, 12) | 74.9 (74.1, 75.4) | 17 (14, 20) | 80.1 (79.5, 80.7) | 14 (10, 17) | 80.3 (79.6, 81) | 19 (15, 23) |
| **Stephenson** | 74.8 (74.2, 75.1) | 10 (8, 13) | 75.5 (74.9, 75.9) | 14 (12, 16) | 80.5 (80, 81) | 11 (8, 14) | 80.8 (80.2, 81.3) | 16 (14, 20) |
| **Tazewell** | 75.3 (74.8, 75.6) | 8 (6, 10) | 75.4 (75, 75.7) | 14 (12, 16) | 80.2 (79.8, 80.5) | 13 (11, 15) | 80.3 (79.9, 80.7) | 19 (17, 22) |
| **Union** | 72.6 (71.9, 73.2) | 20 (17, 23) | 72.9 (72.1, 73.5) | 26 (23, 29) | 79.1 (78.4, 79.6) | 19 (16, 21) | 79.7 (78.9, 80.4) | 22 (18, 26) |
| **Vermilion** | 72.7 (72.3, 73) | 20 (18, 22) | 73.1 (72.6, 73.4) | 25 (23, 27) | 78.5 (78.1, 78.9) | 21 (20, 23) | 78.7 (78.2, 79.1) | 27 (26, 29) |
| **Wabash** | 73.8 (73.1, 74.3) | 14 (12, 18) | 75.2 (74.4, 75.8) | 15 (12, 19) | 80.3 (79.7, 80.9) | 12 (9, 16) | 80.1 (79.3, 80.7) | 20 (17, 25) |
| **Warren** | 74.3 (73.7, 74.9) | 12 (9, 15) | 75.1 (74.4, 75.8) | 15 (12, 19) | 80.1 (79.4, 80.7) | 14 (10, 17) | 79.6 (78.9, 80.3) | 23 (19, 27) |
| **Washington** | 74.2 (73.4, 74.8) | 13 (10, 16) | 75.3 (74.5, 75.9) | 14 (12, 18) | 80.2 (79.5, 80.8) | 13 (9, 17) | 80.6 (79.7, 81.2) | 18 (14, 23) |
| **Wayne** | 73.8 (73.1, 74.4) | 14 (12, 18) | 74.6 (73.8, 75.2) | 18 (15, 22) | 80.2 (79.5, 80.9) | 13 (9, 17) | 80.3 (79.5, 81) | 19 (15, 24) |
| **White** | 73.5 (72.7, 74) | 16 (13, 20) | 73.5 (72.7, 74.1) | 23 (20, 27) | 79 (78.4, 79.5) | 19 (17, 22) | 79.5 (78.7, 80.1) | 24 (20, 27) |
| **Whiteside** | 74.7 (74.2, 75.1) | 10 (8, 13) | 75.2 (74.6, 75.6) | 15 (13, 18) | 80.2 (79.7, 80.6) | 13 (10, 16) | 80.2 (79.6, 80.7) | 20 (17, 23) |
| **Will** | 75.2 (75, 75.4) | 8 (7, 9) | 77 (76.8, 77.2) | 8 (7, 8) | 80.1 (79.8, 80.3) | 14 (12, 15) | 80.9 (80.7, 81.2) | 16 (14, 17) |
| **Williamson** | 73.3 (72.7, 73.7) | 17 (15, 20) | 73.2 (72.7, 73.6) | 24 (22, 27) | 79.5 (78.9, 79.9) | 17 (15, 19) | 79.2 (78.7, 79.7) | 25 (23, 27) |
| **Winnebago** | 74 (73.7, 74.2) | 14 (13, 15) | 75.1 (74.8, 75.4) | 15 (14, 17) | 79.5 (79.2, 79.7) | 17 (16, 18) | 80.8 (80.5, 81.1) | 16 (15, 18) |
| **Woodford** | 75.7 (75.1, 76.2) | 6 (4, 8) | 76.6 (75.9, 77.1) | 9 (7, 12) | 80.7 (80.1, 81.3) | 10 (7, 13) | 81.4 (80.8, 82) | 13 (10, 17) |
| **INDIANA** |  |  |  |  |  |  |  |  |
| **Adams** | 74.7 (74.1, 75.2) | 10 (8, 13) | 75.9 (75.1, 76.5) | 12 (10, 15) | 80.3 (79.7, 80.8) | 12 (9, 16) | 80.8 (80, 81.4) | 16 (13, 21) |
| **Allen** | 74.6 (74.3, 74.8) | 11 (10, 12) | 75.4 (75.2, 75.6) | 14 (13, 15) | 79.7 (79.5, 80) | 15 (14, 17) | 80.6 (80.3, 80.9) | 17 (16, 19) |
| **Bartholomew** | 74.6 (74.1, 75) | 11 (9, 13) | 75.3 (74.7, 75.7) | 15 (13, 17) | 79.4 (78.9, 79.8) | 17 (15, 20) | 80.4 (79.8, 80.9) | 19 (16, 22) |
| **Benton** | 74.1 (73.4, 74.6) | 13 (11, 16) | 74.8 (74.1, 75.4) | 17 (14, 20) | 80.4 (79.7, 81) | 12 (8, 16) | 80.6 (79.8, 81.4) | 17 (13, 22) |
| **Blackford** | 73.2 (72.5, 73.6) | 17 (15, 21) | 74.2 (73.4, 74.7) | 20 (17, 23) | 78.9 (78.3, 79.4) | 20 (17, 22) | 79.5 (78.8, 80.1) | 24 (20, 27) |
| **Boone** | 75.3 (74.7, 75.8) | 7 (5, 10) | 76.6 (76, 77.1) | 9 (7, 11) | 80.1 (79.5, 80.6) | 14 (11, 17) | 80.9 (80.3, 81.4) | 16 (13, 19) |
| **Brown** | 75.3 (74.5, 75.9) | 8 (5, 11) | 75.8 (74.9, 76.5) | 12 (9, 16) | 79.6 (78.9, 80.3) | 16 (12, 19) | 81 (80.1, 81.9) | 15 (11, 21) |
| **Carroll** | 74.8 (74.1, 75.3) | 10 (7, 13) | 75.3 (74.5, 75.9) | 15 (12, 18) | 80.4 (79.7, 80.9) | 12 (9, 16) | 80.8 (79.9, 81.6) | 16 (12, 21) |
| **Cass** | 74.1 (73.5, 74.6) | 13 (11, 16) | 74.5 (73.8, 75) | 18 (16, 21) | 79.6 (79, 80.1) | 16 (14, 19) | 79.4 (78.8, 79.9) | 24 (21, 27) |
| **Clark** | 72.7 (72.3, 73.1) | 20 (18, 22) | 73.3 (72.9, 73.6) | 24 (22, 26) | 78.2 (77.8, 78.6) | 22 (21, 24) | 78.8 (78.3, 79.1) | 27 (26, 29) |
| **Clay** | 73.3 (72.5, 73.8) | 17 (15, 21) | 73.7 (73, 74.3) | 22 (19, 25) | 79.4 (78.8, 80) | 17 (14, 20) | 78.7 (77.9, 79.2) | 28 (25, 30) |
| **Clinton** | 73.5 (72.8, 74) | 16 (14, 19) | 74.6 (73.8, 75.2) | 18 (15, 21) | 79.2 (78.6, 79.7) | 18 (16, 21) | 79.9 (79.2, 80.5) | 22 (18, 25) |
| **Crawford** | 72.4 (71.7, 72.9) | 21 (19, 24) | 73.5 (72.8, 74) | 23 (20, 27) | 78.9 (78.3, 79.4) | 19 (17, 22) | 78.9 (78.2, 79.5) | 27 (24, 29) |
| **Daviess** | 73.6 (73, 74.1) | 15 (13, 18) | 74.2 (73.5, 74.7) | 19 (17, 23) | 79.3 (78.6, 79.8) | 18 (15, 21) | 79.8 (79.1, 80.4) | 22 (19, 26) |
| **De Kalb** | 74.1 (73.5, 74.6) | 13 (11, 16) | 75.4 (74.8, 76) | 14 (11, 17) | 79.4 (78.8, 80) | 17 (14, 20) | 80.4 (79.7, 81) | 19 (15, 23) |
| **Dearborn** | 74.2 (73.5, 74.6) | 13 (11, 16) | 75.3 (74.7, 75.8) | 14 (12, 18) | 79.6 (79, 80.1) | 16 (14, 19) | 80.6 (79.9, 81.1) | 18 (15, 22) |
| **Decatur** | 74.3 (73.6, 74.9) | 12 (9, 15) | 74.3 (73.5, 74.9) | 19 (16, 23) | 79.4 (78.7, 80) | 17 (14, 21) | 80.2 (79.3, 81) | 20 (15, 25) |
| **Delaware** | 73.4 (73, 73.7) | 16 (15, 18) | 73.6 (73.2, 74) | 22 (21, 24) | 78.5 (78.1, 78.8) | 21 (20, 23) | 78.9 (78.5, 79.3) | 27 (25, 28) |
| **Dubois** | 74.2 (73.6, 74.7) | 13 (10, 15) | 75.5 (74.8, 76) | 14 (11, 17) | 80.4 (79.8, 81) | 12 (8, 15) | 80.9 (80.2, 81.6) | 16 (12, 20) |
| **Elkhart** | 74.3 (73.9, 74.6) | 12 (11, 14) | 75.6 (75.2, 76) | 13 (11, 15) | 79.7 (79.3, 80) | 16 (14, 18) | 80.3 (79.9, 80.7) | 19 (17, 22) |
| **Fayette** | 73.2 (72.5, 73.7) | 17 (15, 21) | 72.9 (72.2, 73.5) | 26 (23, 29) | 78.6 (77.9, 79.1) | 21 (19, 23) | 79.2 (78.5, 79.9) | 25 (22, 28) |
| **Floyd** | 74.6 (74, 74.9) | 11 (9, 13) | 74.9 (74.4, 75.3) | 16 (14, 19) | 78.9 (78.4, 79.3) | 19 (18, 21) | 79.3 (78.8, 79.6) | 25 (23, 27) |
| **Fountain** | 73 (72.3, 73.5) | 18 (16, 22) | 74.3 (73.5, 74.9) | 19 (16, 23) | 79.2 (78.6, 79.8) | 18 (15, 21) | 79.5 (78.7, 80.2) | 24 (20, 27) |
| **Franklin** | 74.3 (73.6, 74.8) | 12 (10, 16) | 74.7 (74, 75.3) | 17 (14, 21) | 79.8 (79.1, 80.4) | 15 (12, 19) | 80.7 (79.9, 81.4) | 17 (13, 22) |
| **Fulton** | 74.2 (73.5, 74.7) | 13 (10, 16) | 73.3 (72.7, 73.8) | 24 (21, 27) | 79.5 (78.9, 80) | 17 (14, 20) | 79.3 (78.7, 79.9) | 25 (22, 27) |
| **Gibson** | 73.9 (73.4, 74.4) | 14 (12, 16) | 74.8 (74.2, 75.4) | 17 (14, 20) | 79.5 (79, 80) | 17 (14, 19) | 80 (79.4, 80.6) | 21 (17, 24) |
| **Grant** | 72.5 (72.1, 72.9) | 21 (19, 22) | 73 (72.5, 73.3) | 26 (24, 28) | 78.7 (78.3, 79.1) | 20 (19, 22) | 79.2 (78.7, 79.6) | 25 (23, 27) |
| **Greene** | 73.8 (73.1, 74.3) | 14 (12, 18) | 74.2 (73.5, 74.8) | 19 (17, 23) | 79.9 (79.2, 80.4) | 15 (12, 18) | 79.4 (78.7, 80) | 24 (21, 28) |
| **Hamilton** | 77.1 (76.7, 77.5) | 0 (-1, 2) | 78.6 (78.2, 79) | 1 (-1, 3) | 80.9 (80.5, 81.3) | 9 (7, 11) | 82.7 (82.3, 83.2) | 7 (5, 9) |
| **Hancock** | 75.3 (74.7, 75.7) | 8 (5, 10) | 76.2 (75.6, 76.7) | 10 (9, 13) | 80.1 (79.5, 80.6) | 14 (11, 17) | 80.4 (79.9, 80.9) | 19 (16, 22) |
| **Harrison** | 74.3 (73.7, 74.8) | 12 (10, 15) | 75.1 (74.4, 75.7) | 15 (13, 19) | 80 (79.4, 80.6) | 14 (10, 17) | 80.2 (79.5, 80.9) | 20 (16, 24) |
| **Hendricks** | 75.9 (75.4, 76.3) | 5 (3, 7) | 76.9 (76.5, 77.3) | 8 (6, 9) | 80.1 (79.7, 80.6) | 13 (11, 16) | 80.8 (80.3, 81.2) | 17 (14, 19) |
| **Henry** | 73.7 (73.1, 74.1) | 15 (13, 18) | 73.2 (72.6, 73.7) | 24 (22, 27) | 79.4 (78.9, 79.9) | 17 (15, 20) | 79.3 (78.7, 79.8) | 25 (22, 27) |
| **Howard** | 73.7 (73.2, 74) | 15 (13, 17) | 73.8 (73.3, 74.1) | 22 (20, 24) | 79.4 (78.9, 79.8) | 17 (15, 20) | 79.3 (78.9, 79.7) | 25 (22, 27) |
| **Huntington** | 74.7 (74.1, 75.2) | 10 (8, 13) | 75.7 (75, 76.3) | 13 (10, 16) | 80 (79.4, 80.5) | 14 (11, 17) | 81 (80.4, 81.7) | 15 (12, 19) |
| **Jackson** | 73.3 (72.6, 73.7) | 17 (15, 20) | 74.7 (74, 75.2) | 17 (15, 20) | 79.4 (78.9, 79.9) | 17 (14, 20) | 79.4 (78.8, 79.9) | 24 (21, 27) |
| **Jasper** | 74.3 (73.7, 74.9) | 12 (9, 15) | 74.9 (74.2, 75.5) | 16 (13, 20) | 79.8 (79.2, 80.4) | 15 (12, 18) | 80.6 (79.8, 81.3) | 17 (14, 22) |
| **Jay** | 73.2 (72.5, 73.6) | 17 (15, 21) | 74.2 (73.4, 74.7) | 20 (17, 23) | 78.9 (78.3, 79.4) | 20 (17, 22) | 79.5 (78.8, 80.1) | 24 (20, 27) |
| **Jefferson** | 73 (72.4, 73.5) | 18 (16, 21) | 74.4 (73.7, 74.9) | 19 (16, 22) | 78.7 (78.1, 79.2) | 20 (18, 23) | 79.9 (79.2, 80.5) | 22 (18, 25) |
| **Jennings** | 73.3 (72.6, 73.8) | 17 (14, 20) | 73.2 (72.4, 73.7) | 24 (22, 28) | 78.2 (77.6, 78.7) | 22 (20, 25) | 79.2 (78.4, 79.8) | 25 (22, 28) |
| **Johnson** | 74.9 (74.5, 75.3) | 9 (8, 11) | 76 (75.6, 76.4) | 11 (10, 13) | 79.5 (79.1, 79.9) | 17 (15, 19) | 80.3 (79.9, 80.7) | 19 (17, 22) |
| **Knox** | 72.8 (72.2, 73.2) | 20 (17, 22) | 73.5 (72.8, 74) | 23 (21, 26) | 79.1 (78.6, 79.6) | 19 (16, 21) | 79.9 (79.2, 80.4) | 22 (18, 25) |
| **Kosciusko** | 74.5 (74, 74.9) | 11 (9, 14) | 75.2 (74.7, 75.7) | 15 (13, 17) | 80.2 (79.6, 80.7) | 13 (10, 16) | 80.8 (80.2, 81.4) | 16 (13, 20) |
| **La Porte** | 73.5 (73.1, 73.9) | 16 (14, 18) | 73.7 (73.3, 74) | 22 (20, 24) | 78.7 (78.3, 79.1) | 20 (19, 22) | 80.1 (79.6, 80.5) | 21 (18, 23) |
| **Lagrange** | 73.2 (72.5, 73.7) | 17 (15, 21) | 74.1 (73.4, 74.7) | 20 (17, 23) | 79.3 (78.7, 79.9) | 18 (14, 21) | 79.7 (78.9, 80.3) | 23 (19, 27) |
| **Lake** | 71.1 (70.9, 71.3) | 27 (26, 27) | 72.6 (72.4, 72.8) | 27 (27, 28) | 77.6 (77.4, 77.8) | 25 (24, 25) | 78.6 (78.4, 78.8) | 28 (27, 28) |
| **Lawrence** | 73.3 (72.7, 73.7) | 17 (15, 20) | 74 (73.4, 74.6) | 20 (18, 24) | 79.2 (78.6, 79.7) | 18 (16, 21) | 79.5 (78.9, 80.1) | 24 (20, 27) |
| **Madison** | 73.3 (72.9, 73.6) | 17 (15, 19) | 74.1 (73.7, 74.4) | 20 (19, 22) | 78.8 (78.4, 79.1) | 20 (19, 22) | 79.1 (78.7, 79.4) | 26 (24, 28) |
| **Marion** | 71.4 (71.2, 71.5) | 25 (25, 26) | 72.6 (72.5, 72.7) | 27 (27, 28) | 77.7 (77.5, 77.8) | 24 (24, 25) | 78.7 (78.5, 78.8) | 28 (27, 28) |
| **Marshall** | 74.2 (73.6, 74.7) | 13 (11, 16) | 75 (74.4, 75.6) | 16 (13, 19) | 79.6 (79, 80.1) | 16 (13, 19) | 81.1 (80.4, 81.7) | 15 (12, 19) |
| **Martin** | 73.6 (73, 74.1) | 15 (13, 18) | 74.2 (73.5, 74.7) | 19 (17, 23) | 79.3 (78.6, 79.8) | 18 (15, 21) | 79.8 (79.1, 80.4) | 22 (19, 26) |
| **Miami** | 73.7 (73.1, 74.1) | 15 (13, 18) | 74.2 (73.6, 74.8) | 19 (17, 22) | 79 (78.5, 79.5) | 19 (17, 21) | 79.9 (79.2, 80.5) | 22 (18, 25) |
| **Monroe** | 75.8 (75.3, 76.2) | 5 (3, 7) | 76.6 (76.1, 77) | 9 (8, 11) | 80.3 (79.8, 80.8) | 12 (10, 15) | 81.6 (81.1, 82.1) | 12 (10, 15) |
| **Montgomery** | 74.2 (73.5, 74.7) | 13 (10, 16) | 74.9 (74.2, 75.5) | 16 (13, 19) | 79.4 (78.8, 79.9) | 17 (15, 20) | 80.3 (79.6, 80.9) | 19 (16, 23) |
| **Morgan** | 74 (73.5, 74.5) | 13 (11, 16) | 74.6 (74, 75) | 18 (16, 20) | 79.4 (78.8, 79.8) | 17 (15, 20) | 79.2 (78.7, 79.7) | 25 (23, 27) |
| **Newton** | 73.7 (72.9, 74.3) | 15 (12, 19) | 74 (73.2, 74.7) | 20 (18, 24) | 79.7 (78.9, 80.4) | 15 (11, 19) | 79.3 (78.4, 80) | 25 (21, 29) |
| **Noble** | 73.8 (73.2, 74.4) | 14 (12, 17) | 74.4 (73.8, 75) | 19 (16, 22) | 79.3 (78.7, 79.8) | 18 (15, 21) | 79.9 (79.2, 80.6) | 21 (17, 25) |
| **Ohio** | 73.2 (72.4, 73.8) | 17 (15, 21) | 74.7 (73.7, 75.5) | 17 (14, 22) | 79 (78.2, 79.6) | 19 (16, 22) | 79.5 (78.7, 80.3) | 24 (19, 28) |
| **Orange** | 72.4 (71.7, 72.9) | 21 (19, 24) | 73.5 (72.8, 74) | 23 (20, 27) | 78.9 (78.3, 79.4) | 19 (17, 22) | 78.9 (78.2, 79.5) | 27 (24, 29) |
| **Owen** | 73.8 (73.1, 74.4) | 14 (12, 18) | 73.6 (72.8, 74.2) | 23 (20, 26) | 79.2 (78.6, 79.9) | 18 (15, 21) | 79 (78.2, 79.7) | 26 (23, 29) |
| **Parke** | 73.8 (72.9, 74.3) | 15 (12, 19) | 74.5 (73.7, 75.2) | 18 (15, 22) | 79.4 (78.7, 80.1) | 17 (14, 21) | 80.1 (79.2, 80.9) | 20 (16, 25) |
| **Perry** | 73.5 (72.8, 74) | 16 (14, 19) | 74 (73.3, 74.6) | 21 (18, 24) | 79.3 (78.6, 79.8) | 18 (15, 21) | 79.5 (78.8, 80.2) | 24 (20, 27) |
| **Pike** | 73.9 (73.4, 74.4) | 14 (12, 16) | 74.8 (74.2, 75.4) | 17 (14, 20) | 79.5 (79, 80) | 17 (14, 19) | 80 (79.4, 80.6) | 21 (17, 24) |
| **Porter** | 74.9 (74.5, 75.3) | 9 (8, 11) | 76.2 (75.7, 76.5) | 11 (9, 13) | 79.9 (79.4, 80.3) | 15 (13, 17) | 80.5 (80, 80.8) | 18 (16, 21) |
| **Posey** | 75 (74.4, 75.5) | 9 (6, 12) | 75.1 (74.4, 75.6) | 15 (13, 19) | 80.1 (79.5, 80.6) | 13 (10, 17) | 80.9 (80.2, 81.6) | 16 (12, 20) |
| **Pulaski** | 74.2 (73.5, 74.7) | 13 (10, 16) | 73.3 (72.7, 73.8) | 24 (21, 27) | 79.5 (78.9, 80) | 17 (14, 20) | 79.3 (78.7, 79.9) | 25 (22, 27) |
| **Putnam** | 74.5 (73.9, 75) | 11 (9, 14) | 75.8 (75.1, 76.4) | 12 (10, 15) | 80 (79.4, 80.6) | 14 (11, 18) | 79.7 (79, 80.3) | 23 (19, 26) |
| **Randolph** | 73.8 (73.1, 74.3) | 14 (12, 18) | 74.2 (73.5, 74.7) | 20 (17, 23) | 79.1 (78.5, 79.6) | 19 (16, 21) | 79.5 (78.8, 80.1) | 24 (21, 27) |
| **Ripley** | 73.8 (73.1, 74.4) | 14 (12, 18) | 74.9 (74.1, 75.4) | 17 (14, 20) | 80 (79.3, 80.6) | 14 (10, 18) | 79.8 (79, 80.5) | 22 (18, 26) |
| **Rush** | 73.6 (72.8, 74.2) | 15 (13, 19) | 74.5 (73.7, 75.1) | 18 (15, 22) | 79.3 (78.6, 79.9) | 18 (14, 21) | 80.1 (79.2, 80.8) | 21 (16, 25) |
| **Scott** | 71.8 (71.1, 72.4) | 24 (21, 27) | 72 (71.2, 72.6) | 30 (27, 33) | 78 (77.4, 78.6) | 23 (21, 25) | 77.9 (77.2, 78.6) | 30 (28, 33) |
| **Shelby** | 74.2 (73.6, 74.6) | 13 (11, 15) | 74.4 (73.8, 74.8) | 19 (17, 22) | 79.7 (79.1, 80.2) | 16 (13, 19) | 79.6 (79, 80.2) | 23 (20, 26) |
| **Spencer** | 74.5 (73.8, 75.1) | 11 (9, 15) | 75.3 (74.5, 75.9) | 14 (12, 18) | 80.2 (79.5, 80.9) | 13 (9, 17) | 80.3 (79.5, 81) | 19 (15, 24) |
| **St. Joseph** | 74 (73.7, 74.2) | 14 (13, 15) | 74.4 (74.1, 74.7) | 19 (18, 20) | 79.4 (79.1, 79.6) | 17 (16, 19) | 80.8 (80.5, 81.1) | 16 (15, 18) |
| **Starke** | 71.7 (70.9, 72.2) | 24 (22, 27) | 72.1 (71.3, 72.8) | 29 (26, 33) | 78 (77.3, 78.5) | 23 (21, 25) | 77.8 (77, 78.4) | 31 (28, 34) |
| **Steuben** | 74.4 (73.8, 74.9) | 12 (10, 15) | 75.2 (74.6, 75.8) | 15 (12, 18) | 79.8 (79.2, 80.3) | 15 (12, 18) | 80.5 (79.8, 81.1) | 18 (15, 22) |
| **Sullivan** | 72.7 (72, 73.2) | 20 (18, 23) | 73.1 (72.3, 73.6) | 25 (22, 29) | 78.6 (77.9, 79.2) | 21 (18, 23) | 79 (78.2, 79.6) | 26 (23, 29) |
| **Switzerland** | 73.2 (72.4, 73.8) | 17 (15, 21) | 74.7 (73.7, 75.5) | 17 (14, 22) | 79 (78.2, 79.6) | 19 (16, 22) | 79.5 (78.7, 80.3) | 24 (19, 28) |
| **Tippecanoe** | 74.5 (74.2, 74.9) | 11 (10, 13) | 75.6 (75.2, 76) | 13 (11, 15) | 79.5 (79.1, 79.9) | 17 (15, 19) | 80.7 (80.2, 81.1) | 17 (15, 20) |
| **Tipton** | 75.1 (74.4, 75.6) | 8 (6, 12) | 75.7 (74.8, 76.3) | 13 (10, 17) | 80.2 (79.5, 80.9) | 13 (9, 17) | 80.7 (79.8, 81.4) | 17 (13, 22) |
| **Union** | 74.3 (73.6, 74.8) | 12 (10, 16) | 74.7 (74, 75.3) | 17 (14, 21) | 79.8 (79.1, 80.4) | 15 (12, 19) | 80.7 (79.9, 81.4) | 17 (13, 22) |
| **Vanderburgh** | 73.1 (72.8, 73.4) | 18 (16, 19) | 73.8 (73.4, 74.1) | 22 (20, 23) | 79.3 (79, 79.6) | 18 (16, 19) | 79.4 (79, 79.7) | 24 (23, 26) |
| **Vermillion** | 73.5 (72.7, 74) | 16 (14, 20) | 73.8 (72.9, 74.4) | 22 (19, 26) | 78.8 (78.1, 79.4) | 20 (17, 23) | 79.2 (78.4, 79.8) | 25 (22, 29) |
| **Vigo** | 72.6 (72.2, 72.9) | 20 (19, 22) | 73.6 (73.2, 74) | 22 (21, 24) | 78.3 (77.9, 78.6) | 22 (21, 23) | 79.2 (78.8, 79.6) | 25 (23, 27) |
| **Wabash** | 74.3 (73.7, 74.8) | 12 (10, 15) | 74.9 (74.3, 75.5) | 16 (13, 19) | 80.1 (79.5, 80.7) | 13 (10, 17) | 80.5 (79.8, 81.1) | 18 (15, 22) |
| **Warren** | 74.1 (73.4, 74.6) | 13 (11, 16) | 74.8 (74.1, 75.4) | 17 (14, 20) | 80.4 (79.7, 81) | 12 (8, 16) | 80.6 (79.8, 81.4) | 17 (13, 22) |
| **Warrick** | 74.9 (74.4, 75.3) | 9 (7, 12) | 76.2 (75.6, 76.7) | 10 (9, 13) | 79.8 (79.3, 80.3) | 15 (12, 18) | 79.7 (79.2, 80.2) | 23 (20, 25) |
| **Washington** | 73.2 (72.6, 73.8) | 17 (15, 21) | 73.7 (73, 74.3) | 22 (19, 25) | 79 (78.3, 79.6) | 19 (16, 22) | 79 (78.3, 79.7) | 26 (23, 29) |
| **Wayne** | 72.9 (72.5, 73.3) | 19 (17, 21) | 73.4 (72.9, 73.7) | 23 (22, 26) | 79 (78.6, 79.4) | 19 (17, 21) | 79.6 (79.1, 80) | 23 (21, 26) |
| **Wells** | 74.5 (73.9, 75.1) | 11 (9, 14) | 75.7 (75, 76.3) | 12 (10, 16) | 80.4 (79.7, 80.9) | 12 (9, 16) | 81.4 (80.6, 82.1) | 13 (10, 17) |
| **White** | 74 (73.4, 74.5) | 13 (11, 16) | 74.5 (73.8, 75) | 18 (16, 22) | 79.9 (79.3, 80.5) | 15 (11, 18) | 80.7 (79.9, 81.3) | 17 (14, 22) |
| **Whitley** | 74.9 (74.2, 75.5) | 9 (6, 12) | 75.2 (74.5, 75.7) | 15 (12, 18) | 80.5 (79.8, 81.1) | 11 (8, 15) | 80.6 (79.8, 81.3) | 17 (14, 22) |
| **IOWA** |  |  |  |  |  |  |  |  |
| **Adair** | 75.8 (75, 76.3) | 5 (3, 9) | 76.1 (75.3, 76.7) | 11 (9, 15) | 80.9 (80.2, 81.5) | 9 (6, 13) | 82.6 (81.7, 83.5) | 8 (4, 12) |
| **Adams** | 74.6 (73.8, 75.1) | 11 (9, 14) | 76.4 (75.6, 77.1) | 10 (7, 13) | 80.4 (79.7, 81) | 12 (8, 16) | 80.9 (80, 81.6) | 16 (12, 21) |
| **Allamakee** | 74.8 (74.1, 75.3) | 10 (7, 13) | 76 (75.2, 76.6) | 11 (9, 15) | 80.1 (79.5, 80.7) | 13 (10, 17) | 80.2 (79.4, 80.9) | 20 (16, 24) |
| **Appanoose** | 74.4 (73.6, 74.9) | 12 (9, 15) | 75 (74.2, 75.6) | 16 (13, 20) | 80.3 (79.7, 80.9) | 12 (9, 16) | 80.2 (79.4, 80.8) | 20 (16, 24) |
| **Audubon** | 75.8 (75, 76.3) | 5 (3, 9) | 76.1 (75.3, 76.7) | 11 (9, 15) | 80.9 (80.2, 81.5) | 9 (6, 13) | 82.6 (81.7, 83.5) | 8 (4, 12) |
| **Benton** | 76.1 (75.3, 76.6) | 4 (2, 7) | 77.7 (76.9, 78.4) | 5 (2, 8) | 80.8 (80.1, 81.4) | 9 (6, 13) | 82 (81.2, 82.7) | 10 (7, 14) |
| **Black Hawk** | 75.1 (74.7, 75.3) | 9 (7, 10) | 76.8 (76.4, 77.1) | 8 (7, 10) | 80.4 (80, 80.7) | 12 (10, 14) | 80.8 (80.4, 81.2) | 16 (14, 19) |
| **Boone** | 75.1 (74.5, 75.7) | 8 (6, 11) | 76.4 (75.7, 77) | 10 (8, 13) | 80.7 (80.1, 81.3) | 10 (7, 14) | 81.5 (80.8, 82.2) | 13 (9, 17) |
| **Bremer** | 76.1 (75.4, 76.7) | 4 (2, 7) | 77.6 (76.9, 78.2) | 5 (3, 8) | 81.2 (80.5, 81.8) | 7 (4, 11) | 82.2 (81.4, 82.9) | 10 (6, 13) |
| **Buchanan** | 74.7 (73.9, 75.3) | 10 (7, 14) | 76.2 (75.4, 76.8) | 11 (8, 14) | 80.3 (79.5, 80.9) | 12 (9, 17) | 81.3 (80.4, 82) | 14 (10, 19) |
| **Buena Vista** | 75.1 (74.4, 75.7) | 8 (6, 12) | 76.7 (75.9, 77.3) | 9 (7, 12) | 80.9 (80.2, 81.6) | 9 (5, 13) | 81.2 (80.3, 81.9) | 14 (11, 19) |
| **Butler** | 75.2 (74.6, 75.8) | 8 (5, 11) | 76.3 (75.6, 76.8) | 10 (8, 13) | 81 (80.4, 81.5) | 8 (6, 12) | 81 (80.3, 81.6) | 15 (12, 19) |
| **Calhoun** | 75.1 (74.4, 75.6) | 9 (6, 12) | 76.3 (75.6, 76.9) | 10 (8, 13) | 80 (79.4, 80.5) | 14 (11, 17) | 81.2 (80.4, 81.9) | 14 (11, 18) |
| **Carroll** | 74.9 (74.2, 75.5) | 9 (7, 13) | 76.7 (75.9, 77.2) | 9 (7, 12) | 81.2 (80.5, 81.8) | 7 (4, 11) | 82 (81.2, 82.7) | 10 (7, 14) |
| **Cass** | 75.3 (74.7, 75.8) | 7 (5, 10) | 77 (76.3, 77.5) | 8 (6, 10) | 80.4 (79.8, 80.9) | 12 (9, 15) | 81.6 (80.9, 82.3) | 12 (9, 16) |
| **Cedar** | 75.9 (75.1, 76.4) | 5 (3, 8) | 76.9 (76.2, 77.5) | 8 (6, 11) | 81.4 (80.6, 82) | 6 (3, 10) | 82.2 (81.3, 83) | 9 (6, 14) |
| **Cerro Gordo** | 74.9 (74.4, 75.3) | 9 (7, 12) | 75.7 (75, 76.1) | 13 (11, 16) | 80.4 (79.9, 80.9) | 11 (9, 15) | 81.5 (80.9, 82) | 13 (10, 16) |
| **Cherokee** | 75.1 (74.4, 75.6) | 8 (6, 12) | 75.9 (75.2, 76.4) | 12 (10, 15) | 80.9 (80.2, 81.4) | 9 (6, 13) | 81.8 (81.1, 82.5) | 11 (8, 15) |
| **Chickasaw** | 75.2 (74.6, 75.8) | 8 (5, 11) | 76.3 (75.6, 76.8) | 10 (8, 13) | 81 (80.4, 81.5) | 8 (6, 12) | 81 (80.3, 81.6) | 15 (12, 19) |
| **Clarke** | 74.4 (73.7, 75) | 12 (9, 15) | 75.5 (74.7, 76.2) | 13 (11, 17) | 80.5 (79.8, 81.2) | 11 (7, 15) | 80.8 (79.9, 81.6) | 16 (12, 21) |
| **Clay** | 75.3 (74.6, 75.9) | 7 (5, 11) | 77 (76.2, 77.7) | 8 (5, 10) | 80.9 (80.3, 81.6) | 9 (5, 12) | 81.6 (80.8, 82.3) | 12 (9, 16) |
| **Clayton** | 74.7 (73.9, 75.3) | 10 (8, 14) | 76.1 (75.3, 76.8) | 11 (8, 14) | 80 (79.3, 80.6) | 14 (11, 18) | 81.5 (80.6, 82.3) | 13 (9, 17) |
| **Clinton** | 74.6 (74.1, 75) | 11 (9, 13) | 75.5 (75, 76) | 13 (11, 16) | 79.8 (79.3, 80.3) | 15 (13, 18) | 80.7 (80.1, 81.3) | 17 (14, 20) |
| **Crawford** | 74.7 (73.9, 75.2) | 10 (8, 14) | 75.4 (74.6, 76) | 14 (11, 18) | 81 (80.3, 81.7) | 8 (5, 13) | 81.7 (80.7, 82.4) | 12 (9, 17) |
| **Dallas** | 75.9 (75.3, 76.4) | 5 (3, 7) | 77 (76.4, 77.5) | 8 (6, 10) | 80.8 (80.2, 81.4) | 9 (6, 13) | 81.9 (81.1, 82.5) | 11 (8, 15) |
| **Davis** | 74 (73.3, 74.5) | 14 (11, 17) | 74.7 (74, 75.3) | 17 (15, 21) | 80.7 (80, 81.3) | 10 (7, 14) | 80.9 (80.1, 81.6) | 16 (12, 21) |
| **Decatur** | 74.4 (73.7, 75) | 12 (9, 15) | 75.5 (74.7, 76.2) | 13 (11, 17) | 80.5 (79.8, 81.2) | 11 (7, 15) | 80.8 (79.9, 81.6) | 16 (12, 21) |
| **Delaware** | 75.1 (74.4, 75.7) | 8 (5, 12) | 77.4 (76.5, 78.1) | 6 (4, 9) | 81.3 (80.5, 82) | 7 (3, 11) | 81.7 (80.8, 82.5) | 12 (8, 16) |
| **Des Moines** | 74.7 (74.1, 75.1) | 10 (8, 13) | 75.9 (75.2, 76.3) | 12 (10, 15) | 80.6 (80, 81.1) | 11 (8, 14) | 81.6 (80.9, 82.2) | 12 (10, 16) |
| **Dickinson** | 76 (75.3, 76.5) | 4 (2, 8) | 78 (77.1, 78.6) | 4 (1, 7) | 81.2 (80.5, 81.9) | 7 (4, 11) | 82.1 (81.2, 82.8) | 10 (7, 14) |
| **Dubuque** | 75.4 (75, 75.8) | 7 (5, 9) | 76.9 (76.4, 77.3) | 8 (7, 10) | 80.2 (79.8, 80.6) | 13 (11, 15) | 81.5 (81, 81.9) | 13 (11, 15) |
| **Emmet** | 74.5 (73.9, 75) | 11 (9, 14) | 75.7 (74.9, 76.2) | 13 (10, 16) | 80.8 (80.1, 81.4) | 9 (6, 13) | 81.2 (80.5, 81.9) | 14 (11, 18) |
| **Fayette** | 74.7 (74, 75.2) | 10 (8, 13) | 76.1 (75.4, 76.7) | 11 (9, 14) | 80.9 (80.3, 81.5) | 9 (6, 12) | 80.8 (80, 81.4) | 17 (13, 21) |
| **Floyd** | 75.1 (74.4, 75.7) | 8 (6, 12) | 76.5 (75.7, 77.1) | 9 (7, 13) | 80.8 (80.1, 81.4) | 9 (6, 13) | 81.1 (80.3, 81.8) | 15 (11, 19) |
| **Franklin** | 75.3 (74.6, 75.8) | 8 (5, 11) | 76.6 (75.8, 77.2) | 9 (7, 12) | 80.9 (80.2, 81.6) | 9 (5, 13) | 81.4 (80.6, 82.1) | 13 (10, 17) |
| **Fremont** | 74.3 (73.6, 74.8) | 12 (10, 16) | 76.2 (75.4, 76.8) | 10 (8, 14) | 79.7 (79.1, 80.3) | 16 (12, 19) | 81.1 (80.3, 81.8) | 15 (11, 19) |
| **Greene** | 75.1 (74.4, 75.7) | 8 (6, 12) | 76.2 (75.5, 76.8) | 10 (8, 13) | 80.9 (80.2, 81.5) | 9 (6, 13) | 82 (81.2, 82.7) | 11 (7, 14) |
| **Grundy** | 74.9 (74.2, 75.4) | 9 (7, 13) | 77.1 (76.3, 77.6) | 7 (5, 10) | 81.5 (80.8, 82) | 6 (3, 9) | 81.4 (80.7, 82) | 13 (10, 17) |
| **Guthrie** | 75.1 (74.4, 75.7) | 8 (6, 12) | 76.2 (75.5, 76.8) | 10 (8, 13) | 80.9 (80.2, 81.5) | 9 (6, 13) | 82 (81.2, 82.7) | 11 (7, 14) |
| **Hamilton** | 75.5 (74.7, 76) | 7 (4, 10) | 76.7 (75.9, 77.3) | 9 (7, 12) | 81 (80.2, 81.6) | 8 (5, 13) | 82.4 (81.5, 83.2) | 9 (5, 13) |
| **Hancock** | 75.3 (74.6, 75.8) | 8 (5, 11) | 76.6 (75.8, 77.2) | 9 (7, 12) | 80.9 (80.2, 81.6) | 9 (5, 13) | 81.4 (80.6, 82.1) | 13 (10, 17) |
| **Hardin** | 74.8 (74.1, 75.4) | 10 (7, 13) | 76 (75.2, 76.7) | 11 (9, 15) | 80.9 (80.3, 81.5) | 9 (6, 13) | 81.1 (80.4, 81.8) | 15 (11, 19) |
| **Harrison** | 74.6 (73.8, 75.1) | 11 (8, 14) | 75 (74.3, 75.6) | 16 (13, 19) | 80.2 (79.5, 80.8) | 13 (9, 17) | 80.4 (79.6, 81) | 19 (15, 23) |
| **Henry** | 75.4 (74.7, 75.9) | 7 (5, 10) | 76 (75.3, 76.5) | 11 (9, 14) | 80.8 (80.1, 81.5) | 9 (6, 13) | 81.4 (80.5, 82.2) | 13 (10, 18) |
| **Howard** | 75.2 (74.5, 75.7) | 8 (6, 11) | 76.6 (75.9, 77.2) | 9 (7, 12) | 81.1 (80.4, 81.7) | 8 (5, 12) | 81.4 (80.6, 82.1) | 13 (10, 17) |
| **Humboldt** | 75.6 (74.8, 76.1) | 6 (4, 10) | 75.9 (75.2, 76.5) | 12 (9, 15) | 80.9 (80.3, 81.6) | 9 (5, 12) | 81.1 (80.3, 81.9) | 15 (11, 19) |
| **Ida** | 74.6 (73.9, 75.1) | 11 (8, 14) | 75.1 (74.3, 75.7) | 15 (13, 19) | 80.3 (79.7, 80.9) | 12 (9, 16) | 80.4 (79.6, 81) | 19 (16, 23) |
| **Iowa** | 75.2 (74.6, 75.7) | 8 (5, 11) | 76.6 (75.9, 77.1) | 9 (7, 12) | 80.6 (80, 81.2) | 10 (7, 14) | 81.8 (81, 82.5) | 11 (8, 15) |
| **Jackson** | 74 (73.2, 74.5) | 14 (11, 17) | 76 (75.2, 76.6) | 11 (9, 15) | 80.8 (80, 81.4) | 9 (6, 14) | 80.8 (80, 81.6) | 16 (12, 21) |
| **Jasper** | 75.8 (75.1, 76.3) | 5 (3, 8) | 76.9 (76.2, 77.4) | 8 (6, 11) | 80.9 (80.3, 81.5) | 9 (6, 12) | 80.9 (80.2, 81.5) | 16 (13, 20) |
| **Jefferson** | 75.1 (74.4, 75.6) | 9 (6, 12) | 76.9 (76, 77.6) | 8 (6, 11) | 80.4 (79.7, 81.1) | 11 (8, 16) | 80.9 (80.1, 81.6) | 16 (12, 21) |
| **Johnson** | 77.6 (77.1, 78.1) | -2 (-3, 0) | 79.3 (78.7, 79.8) | -3 (-6, 1) | 81.4 (80.9, 81.9) | 6 (4, 9) | 82.5 (81.9, 83.1) | 8 (6, 11) |
| **Jones** | 75.2 (74.5, 75.7) | 8 (6, 11) | 76.1 (75.4, 76.7) | 11 (9, 14) | 81.1 (80.3, 81.7) | 8 (5, 12) | 81.4 (80.5, 82.1) | 13 (10, 18) |
| **Keokuk** | 75.2 (74.6, 75.7) | 8 (5, 11) | 76.6 (75.9, 77.1) | 9 (7, 12) | 80.6 (80, 81.2) | 10 (7, 14) | 81.8 (81, 82.5) | 11 (8, 15) |
| **Kossuth** | 75.2 (74.5, 75.8) | 8 (5, 11) | 77.1 (76.3, 77.8) | 7 (5, 10) | 80.8 (80.1, 81.5) | 9 (6, 13) | 82.7 (81.8, 83.5) | 7 (3, 11) |
| **Lee** | 73.9 (73.3, 74.3) | 14 (12, 17) | 74.5 (73.9, 75) | 18 (16, 21) | 79.7 (79.1, 80.2) | 16 (13, 19) | 80.1 (79.5, 80.6) | 21 (17, 24) |
| **Linn** | 76.6 (76.3, 76.9) | 2 (1, 3) | 77.6 (77.2, 78) | 5 (4, 7) | 81.3 (80.9, 81.7) | 7 (5, 9) | 81.5 (81.1, 81.9) | 13 (11, 15) |
| **Louisa** | 75.8 (75.1, 76.3) | 5 (3, 8) | 76.4 (75.7, 76.9) | 10 (8, 13) | 81.4 (80.7, 82) | 6 (3, 10) | 82 (81.2, 82.7) | 10 (7, 14) |
| **Lucas** | 74.4 (73.6, 74.9) | 12 (9, 15) | 75 (74.2, 75.6) | 16 (13, 20) | 80.3 (79.7, 80.9) | 12 (9, 16) | 80.2 (79.4, 80.8) | 20 (16, 24) |
| **Lyon** | 74.9 (74.2, 75.5) | 9 (7, 13) | 75.8 (75, 76.4) | 12 (10, 16) | 81.4 (80.6, 82.1) | 6 (3, 10) | 81.2 (80.3, 82) | 14 (11, 19) |
| **Madison** | 74.5 (73.9, 75) | 11 (9, 14) | 76.5 (75.8, 77.1) | 9 (7, 12) | 80.2 (79.6, 80.8) | 13 (9, 16) | 81.5 (80.6, 82.1) | 13 (10, 17) |
| **Mahaska** | 75.7 (75, 76.3) | 5 (3, 9) | 76.3 (75.5, 76.9) | 10 (8, 14) | 80.5 (79.8, 81.1) | 11 (8, 15) | 81.6 (80.7, 82.4) | 12 (9, 17) |
| **Marion** | 74.5 (73.9, 74.9) | 11 (9, 14) | 75.7 (75, 76.2) | 13 (11, 16) | 81.2 (80.5, 81.7) | 7 (5, 11) | 81.1 (80.3, 81.6) | 15 (12, 19) |
| **Marshall** | 73 (72.4, 73.3) | 19 (17, 21) | 74.4 (73.9, 74.8) | 19 (17, 21) | 80.5 (79.9, 81) | 11 (8, 14) | 81.7 (81, 82.3) | 12 (9, 16) |
| **Mills** | 74.4 (73.6, 75) | 12 (9, 16) | 75.9 (74.9, 76.5) | 12 (9, 16) | 80.5 (79.6, 81.2) | 11 (7, 16) | 80.4 (79.5, 81.2) | 18 (14, 24) |
| **Mitchell** | 75.2 (74.5, 75.7) | 8 (6, 11) | 76.6 (75.9, 77.2) | 9 (7, 12) | 81.1 (80.4, 81.7) | 8 (5, 12) | 81.4 (80.6, 82.1) | 13 (10, 17) |
| **Monona** | 74.6 (73.9, 75.1) | 11 (8, 14) | 75.1 (74.3, 75.7) | 15 (13, 19) | 80.3 (79.7, 80.9) | 12 (9, 16) | 80.4 (79.6, 81) | 19 (16, 23) |
| **Monroe** | 74 (73.3, 74.5) | 14 (11, 17) | 74.7 (74, 75.3) | 17 (15, 21) | 80.7 (80, 81.3) | 10 (7, 14) | 80.9 (80.1, 81.6) | 16 (12, 21) |
| **Montgomery** | 74.3 (73.6, 74.8) | 12 (10, 16) | 76.2 (75.4, 76.8) | 10 (8, 14) | 79.7 (79.1, 80.3) | 16 (12, 19) | 81.1 (80.3, 81.8) | 15 (11, 19) |
| **Muscatine** | 74.8 (74.2, 75.3) | 10 (8, 13) | 75.7 (75.1, 76.2) | 13 (11, 16) | 80.7 (80, 81.2) | 10 (7, 14) | 80.7 (80, 81.3) | 17 (14, 21) |
| **O'Brien** | 75.1 (74.4, 75.6) | 8 (6, 12) | 75.9 (75.2, 76.4) | 12 (10, 15) | 80.9 (80.2, 81.4) | 9 (6, 13) | 81.8 (81.1, 82.5) | 11 (8, 15) |
| **Osceola** | 74.9 (74.2, 75.5) | 9 (7, 13) | 75.8 (75, 76.4) | 12 (10, 16) | 81.4 (80.6, 82.1) | 6 (3, 10) | 81.2 (80.3, 82) | 14 (11, 19) |
| **Page** | 74.8 (74.1, 75.3) | 10 (8, 13) | 76.3 (75.6, 76.8) | 10 (8, 13) | 79.9 (79.3, 80.5) | 15 (11, 18) | 80.7 (79.9, 81.4) | 17 (13, 22) |
| **Palo Alto** | 74.5 (73.9, 75) | 11 (9, 14) | 75.7 (74.9, 76.2) | 13 (10, 16) | 80.8 (80.1, 81.4) | 9 (6, 13) | 81.2 (80.5, 81.9) | 14 (11, 18) |
| **Plymouth** | 75.4 (74.7, 76) | 7 (4, 10) | 76.4 (75.7, 77) | 10 (8, 13) | 81 (80.3, 81.6) | 8 (5, 12) | 81.8 (81, 82.6) | 11 (8, 15) |
| **Pocahontas** | 75.6 (74.8, 76.1) | 6 (4, 10) | 75.9 (75.2, 76.5) | 12 (9, 15) | 80.9 (80.3, 81.6) | 9 (5, 12) | 81.1 (80.3, 81.9) | 15 (11, 19) |
| **Polk** | 75 (74.8, 75.3) | 9 (8, 10) | 76.4 (76.2, 76.7) | 10 (9, 11) | 80.3 (80, 80.6) | 12 (11, 14) | 81.2 (80.9, 81.4) | 14 (13, 16) |
| **Pottawattamie** | 73.7 (73.3, 74.1) | 15 (13, 17) | 74.9 (74.4, 75.3) | 16 (14, 19) | 79.7 (79.2, 80.1) | 16 (14, 18) | 80 (79.5, 80.4) | 21 (19, 24) |
| **Poweshiek** | 75.4 (74.8, 76) | 7 (4, 10) | 77.4 (76.6, 78.1) | 6 (4, 9) | 80.8 (80.1, 81.5) | 9 (6, 13) | 80.8 (80, 81.5) | 16 (13, 21) |
| **Ringgold** | 74.6 (73.8, 75.1) | 11 (9, 14) | 76.4 (75.6, 77.1) | 10 (7, 13) | 80.4 (79.7, 81) | 12 (8, 16) | 80.9 (80, 81.6) | 16 (12, 21) |
| **Sac** | 75.1 (74.4, 75.6) | 9 (6, 12) | 76.3 (75.6, 76.9) | 10 (8, 13) | 80 (79.4, 80.5) | 14 (11, 17) | 81.2 (80.4, 81.9) | 14 (11, 18) |
| **Scott** | 75.1 (74.7, 75.4) | 8 (7, 10) | 76 (75.7, 76.4) | 11 (10, 13) | 79.8 (79.5, 80.2) | 15 (13, 17) | 80.9 (80.5, 81.2) | 16 (14, 18) |
| **Shelby** | 75.3 (74.7, 75.8) | 7 (5, 10) | 77 (76.3, 77.5) | 8 (6, 10) | 80.4 (79.8, 80.9) | 12 (9, 15) | 81.6 (80.9, 82.3) | 12 (9, 16) |
| **Sioux** | 76.4 (75.7, 76.9) | 3 (1, 6) | 77.5 (76.7, 78.1) | 6 (4, 9) | 81.2 (80.5, 81.8) | 7 (4, 11) | 82.8 (81.9, 83.6) | 7 (3, 11) |
| **Story** | 77.2 (76.7, 77.7) | 0 (-2, 2) | 78.5 (77.9, 79) | 2 (-1, 4) | 82.3 (81.7, 82.9) | 2 (0, 5) | 83.1 (82.4, 83.7) | 6 (2, 9) |
| **Tama** | 74.9 (74.2, 75.4) | 9 (7, 13) | 77.1 (76.3, 77.6) | 7 (5, 10) | 81.5 (80.8, 82) | 6 (3, 9) | 81.4 (80.7, 82) | 13 (10, 17) |
| **Taylor** | 74.6 (73.8, 75.1) | 11 (9, 14) | 76.4 (75.6, 77.1) | 10 (7, 13) | 80.4 (79.7, 81) | 12 (8, 16) | 80.9 (80, 81.6) | 16 (12, 21) |
| **Union** | 74.5 (73.9, 75) | 11 (9, 14) | 76.5 (75.8, 77.1) | 9 (7, 12) | 80.2 (79.6, 80.8) | 13 (9, 16) | 81.5 (80.6, 82.1) | 13 (10, 17) |
| **Van Buren** | 75.1 (74.4, 75.6) | 9 (6, 12) | 76.9 (76, 77.6) | 8 (6, 11) | 80.4 (79.7, 81.1) | 11 (8, 16) | 80.9 (80.1, 81.6) | 16 (12, 21) |
| **Wapello** | 73.8 (73.2, 74.3) | 14 (12, 17) | 75.1 (74.4, 75.6) | 16 (13, 19) | 79.6 (79, 80) | 16 (14, 19) | 80.8 (80, 81.3) | 16 (13, 21) |
| **Warren** | 75.8 (75.1, 76.3) | 5 (3, 8) | 77.1 (76.5, 77.6) | 7 (6, 10) | 80.5 (79.9, 81) | 11 (8, 15) | 81.8 (81.1, 82.6) | 11 (8, 15) |
| **Washington** | 75.8 (75.1, 76.3) | 5 (3, 8) | 76.4 (75.7, 76.9) | 10 (8, 13) | 81.4 (80.7, 82) | 6 (3, 10) | 82 (81.2, 82.7) | 10 (7, 14) |
| **Wayne** | 74 (73.3, 74.5) | 14 (11, 17) | 74.7 (74, 75.3) | 17 (15, 21) | 80.7 (80, 81.3) | 10 (7, 14) | 80.9 (80.1, 81.6) | 16 (12, 21) |
| **Webster** | 74.4 (73.8, 74.8) | 12 (10, 14) | 75.1 (74.5, 75.5) | 16 (13, 18) | 80 (79.5, 80.5) | 14 (11, 17) | 80.2 (79.5, 80.7) | 20 (17, 24) |
| **Winnebago** | 75.2 (74.5, 75.8) | 8 (5, 11) | 76.5 (75.6, 77.1) | 9 (7, 13) | 81.2 (80.4, 81.8) | 7 (4, 11) | 81.8 (80.9, 82.5) | 11 (8, 16) |
| **Winneshiek** | 76.1 (75.3, 76.6) | 4 (2, 7) | 77.6 (76.7, 78.2) | 6 (3, 9) | 81.4 (80.7, 82) | 6 (3, 10) | 82.9 (81.9, 83.6) | 6 (3, 11) |
| **Woodbury** | 74.3 (73.9, 74.6) | 12 (11, 14) | 75.4 (75, 75.8) | 14 (12, 16) | 80.1 (79.7, 80.5) | 13 (11, 16) | 80.5 (80, 80.9) | 18 (16, 21) |
| **Worth** | 75.2 (74.5, 75.8) | 8 (5, 11) | 76.5 (75.6, 77.1) | 9 (7, 13) | 81.2 (80.4, 81.8) | 7 (4, 11) | 81.8 (80.9, 82.5) | 11 (8, 16) |
| **Wright** | 75.6 (74.8, 76.2) | 6 (4, 10) | 76 (75.2, 76.6) | 11 (9, 15) | 81 (80.2, 81.6) | 8 (5, 13) | 81.9 (80.9, 82.6) | 11 (8, 16) |
| **KANSAS** |  |  |  |  |  |  |  |  |
| **Allen** | 73.7 (73, 74.2) | 15 (13, 18) | 74.7 (74, 75.3) | 17 (14, 21) | 79.5 (78.8, 80.1) | 17 (14, 20) | 79.4 (78.7, 80.1) | 24 (21, 27) |
| **Anderson** | 74.8 (74, 75.3) | 10 (7, 13) | 75.2 (74.4, 75.8) | 15 (12, 19) | 80.6 (80, 81.3) | 10 (7, 14) | 80.6 (79.8, 81.3) | 18 (14, 22) |
| **Atchison** | 74.2 (73.5, 74.7) | 13 (11, 16) | 75.2 (74.5, 75.7) | 15 (12, 18) | 79.6 (79, 80.1) | 16 (13, 19) | 79.9 (79.2, 80.5) | 22 (18, 25) |
| **Barber** | 73.7 (72.9, 74.3) | 15 (12, 19) | 75.3 (74.5, 75.9) | 15 (12, 18) | 80.2 (79.6, 80.8) | 13 (9, 16) | 80.1 (79.4, 80.8) | 20 (16, 24) |
| **Barton** | 74.6 (73.9, 75.1) | 11 (8, 14) | 75.6 (74.8, 76.2) | 13 (11, 17) | 80.4 (79.7, 80.9) | 12 (9, 16) | 81.9 (81, 82.5) | 11 (8, 15) |
| **Bourbon** | 73.5 (72.8, 74.1) | 16 (13, 19) | 74.6 (73.8, 75.3) | 18 (14, 22) | 79.9 (79.2, 80.5) | 15 (11, 18) | 79.4 (78.6, 80.1) | 24 (21, 28) |
| **Brown** | 74.7 (74, 75.2) | 11 (8, 14) | 75.1 (74.5, 75.7) | 15 (12, 18) | 80.9 (80.2, 81.5) | 9 (6, 13) | 80.9 (80.2, 81.6) | 16 (12, 20) |
| **Butler** | 74.8 (74.3, 75.3) | 10 (8, 12) | 75.8 (75.2, 76.3) | 12 (10, 15) | 80.2 (79.7, 80.7) | 13 (10, 16) | 80.3 (79.7, 80.8) | 19 (16, 23) |
| **Chase** | 74.3 (73.5, 74.8) | 12 (10, 16) | 74.7 (73.9, 75.3) | 17 (14, 21) | 80.3 (79.5, 80.9) | 12 (9, 17) | 80.4 (79.6, 81.1) | 18 (15, 23) |
| **Chautauqua** | 73.4 (72.8, 73.8) | 17 (14, 20) | 73 (72.4, 73.5) | 25 (23, 28) | 79.5 (79, 80) | 17 (14, 19) | 79.9 (79.3, 80.5) | 21 (18, 25) |
| **Cherokee** | 72.5 (71.8, 73.1) | 21 (18, 23) | 73.2 (72.5, 73.8) | 24 (21, 28) | 79.2 (78.5, 79.7) | 18 (16, 21) | 78.9 (78.1, 79.5) | 27 (24, 30) |
| **Cheyenne** | 74.9 (74.2, 75.4) | 9 (7, 13) | 76 (75.2, 76.5) | 11 (9, 15) | 81.7 (81, 82.3) | 5 (2, 8) | 80.6 (79.8, 81.2) | 18 (14, 22) |
| **Clark** | 73.6 (73.1, 74.1) | 15 (13, 18) | 75.4 (74.8, 75.9) | 14 (12, 17) | 79.8 (79.2, 80.3) | 15 (12, 18) | 80 (79.4, 80.5) | 21 (18, 25) |
| **Clay** | 75 (74.3, 75.6) | 9 (6, 12) | 75.8 (75, 76.5) | 12 (10, 16) | 80.7 (80, 81.3) | 10 (7, 14) | 81.2 (80.3, 81.9) | 14 (11, 19) |
| **Cloud** | 75 (74.3, 75.6) | 9 (6, 12) | 75.8 (75, 76.5) | 12 (10, 16) | 80.7 (80, 81.3) | 10 (7, 14) | 81.2 (80.3, 81.9) | 14 (11, 19) |
| **Coffey** | 74.8 (74, 75.3) | 10 (7, 13) | 75.2 (74.4, 75.8) | 15 (12, 19) | 80.6 (80, 81.3) | 10 (7, 14) | 80.6 (79.8, 81.3) | 18 (14, 22) |
| **Comanche** | 73.6 (73.1, 74.1) | 15 (13, 18) | 75.4 (74.8, 75.9) | 14 (12, 17) | 79.8 (79.2, 80.3) | 15 (12, 18) | 80 (79.4, 80.5) | 21 (18, 25) |
| **Cowley** | 73.9 (73.2, 74.3) | 14 (12, 17) | 73.1 (72.5, 73.6) | 25 (23, 28) | 79.8 (79.2, 80.4) | 15 (12, 18) | 79.9 (79.2, 80.5) | 22 (18, 25) |
| **Crawford** | 73.3 (72.7, 73.7) | 17 (15, 20) | 73.9 (73.3, 74.4) | 21 (19, 24) | 79.8 (79.2, 80.3) | 15 (13, 18) | 79.6 (78.9, 80.2) | 23 (20, 27) |
| **Decatur** | 74.9 (74.2, 75.4) | 9 (7, 13) | 76 (75.2, 76.5) | 11 (9, 15) | 81.7 (81, 82.3) | 5 (2, 8) | 80.6 (79.8, 81.2) | 18 (14, 22) |
| **Dickinson** | 74.7 (74, 75.2) | 10 (8, 13) | 75.1 (74.4, 75.6) | 15 (13, 19) | 80.8 (80.2, 81.4) | 9 (6, 13) | 81.2 (80.4, 81.9) | 14 (11, 19) |
| **Doniphan** | 74.2 (73.5, 74.7) | 13 (11, 16) | 75.2 (74.5, 75.7) | 15 (12, 18) | 79.6 (79, 80.1) | 16 (13, 19) | 79.9 (79.2, 80.5) | 22 (18, 25) |
| **Douglas** | 76.7 (76.2, 77.1) | 2 (0, 3) | 78.1 (77.5, 78.5) | 4 (2, 6) | 81.1 (80.5, 81.5) | 8 (6, 11) | 81.9 (81.3, 82.4) | 11 (9, 14) |
| **Edwards** | 74.4 (73.7, 74.9) | 12 (9, 15) | 74.9 (74.1, 75.6) | 16 (13, 20) | 80.3 (79.5, 80.8) | 13 (9, 17) | 80.1 (79.3, 80.8) | 20 (16, 25) |
| **Elk** | 73.4 (72.8, 73.8) | 17 (14, 20) | 73 (72.4, 73.5) | 25 (23, 28) | 79.5 (79, 80) | 17 (14, 19) | 79.9 (79.3, 80.5) | 21 (18, 25) |
| **Ellis** | 75.4 (74.7, 76) | 7 (4, 10) | 76.6 (75.9, 77.2) | 9 (7, 12) | 80.8 (80.1, 81.4) | 9 (6, 13) | 81.6 (80.8, 82.3) | 12 (9, 16) |
| **Ellsworth** | 74.4 (73.6, 74.9) | 12 (9, 15) | 75.2 (74.4, 75.8) | 15 (12, 19) | 80.9 (80.2, 81.5) | 9 (6, 13) | 80.7 (79.9, 81.4) | 17 (13, 22) |
| **Finney** | 73.9 (73.3, 74.4) | 14 (12, 17) | 75.3 (74.7, 76) | 14 (11, 18) | 79.6 (79, 80.2) | 16 (13, 19) | 80.2 (79.5, 80.9) | 20 (16, 24) |
| **Ford** | 73.6 (73.1, 74.1) | 15 (13, 18) | 75.4 (74.8, 75.9) | 14 (12, 17) | 79.8 (79.2, 80.3) | 15 (12, 18) | 80 (79.4, 80.5) | 21 (18, 25) |
| **Franklin** | 74.2 (73.6, 74.8) | 12 (10, 16) | 75.7 (75, 76.3) | 13 (10, 16) | 79.9 (79.2, 80.5) | 15 (11, 18) | 79.6 (78.9, 80.2) | 23 (20, 26) |
| **Geary** | 72.9 (72.3, 73.3) | 19 (17, 22) | 74.4 (73.7, 74.8) | 19 (17, 22) | 79.5 (78.9, 80.1) | 17 (14, 20) | 80.2 (79.4, 80.8) | 20 (17, 24) |
| **Gove** | 75.1 (74.4, 75.6) | 8 (6, 12) | 76.3 (75.6, 76.9) | 10 (8, 13) | 81 (80.3, 81.5) | 8 (6, 12) | 81.5 (80.7, 82.1) | 13 (10, 17) |
| **Graham** | 75.1 (74.4, 75.6) | 8 (6, 12) | 76.3 (75.6, 76.9) | 10 (8, 13) | 81 (80.3, 81.5) | 8 (6, 12) | 81.5 (80.7, 82.1) | 13 (10, 17) |
| **Grant** | 73.7 (72.9, 74.3) | 15 (12, 19) | 74.6 (73.8, 75.2) | 18 (15, 22) | 79.7 (79, 80.3) | 16 (12, 19) | 79.4 (78.6, 80.1) | 24 (21, 28) |
| **Gray** | 73.9 (73.3, 74.4) | 14 (12, 17) | 75.3 (74.7, 76) | 14 (11, 18) | 79.6 (79, 80.2) | 16 (13, 19) | 80.2 (79.5, 80.9) | 20 (16, 24) |
| **Greeley** | 74.7 (74, 75.3) | 10 (7, 14) | 75.9 (75.1, 76.5) | 12 (9, 16) | 80.7 (80, 81.4) | 10 (6, 14) | 81.2 (80.3, 82) | 14 (10, 19) |
| **Greenwood** | 74.3 (73.5, 74.8) | 12 (10, 16) | 74.7 (73.9, 75.3) | 17 (14, 21) | 80.3 (79.5, 80.9) | 12 (9, 17) | 80.4 (79.6, 81.1) | 18 (15, 23) |
| **Hamilton** | 74.7 (74, 75.3) | 10 (7, 14) | 75.9 (75.1, 76.5) | 12 (9, 16) | 80.7 (80, 81.4) | 10 (6, 14) | 81.2 (80.3, 82) | 14 (10, 19) |
| **Harper** | 73.7 (72.9, 74.3) | 15 (12, 19) | 75.3 (74.5, 75.9) | 15 (12, 18) | 80.2 (79.6, 80.8) | 13 (9, 16) | 80.1 (79.4, 80.8) | 20 (16, 24) |
| **Harvey** | 75.5 (74.9, 76) | 6 (4, 9) | 76 (75.3, 76.6) | 11 (9, 14) | 80.6 (79.9, 81.2) | 11 (7, 14) | 80.9 (80.2, 81.6) | 16 (12, 20) |
| **Haskell** | 73.9 (73.3, 74.4) | 14 (12, 17) | 75.3 (74.7, 76) | 14 (11, 18) | 79.6 (79, 80.2) | 16 (13, 19) | 80.2 (79.5, 80.9) | 20 (16, 24) |
| **Hodgeman** | 74.8 (74.1, 75.3) | 10 (7, 13) | 75.2 (74.4, 75.7) | 15 (12, 19) | 80.6 (79.9, 81.2) | 10 (7, 15) | 80.6 (79.8, 81.3) | 17 (14, 22) |
| **Jackson** | 74.9 (74.2, 75.4) | 10 (7, 13) | 75.8 (75.2, 76.4) | 12 (10, 15) | 80.5 (79.9, 81.1) | 11 (8, 15) | 80.8 (80.1, 81.5) | 16 (13, 20) |
| **Jefferson** | 74.7 (73.9, 75.2) | 10 (8, 14) | 75.4 (74.5, 75.9) | 14 (12, 18) | 80.7 (79.8, 81.3) | 10 (7, 15) | 80 (79.2, 80.6) | 21 (17, 25) |
| **Jewell** | 74.7 (74, 75.3) | 10 (7, 14) | 75.7 (74.9, 76.3) | 13 (10, 16) | 80.5 (79.8, 81) | 11 (8, 15) | 82 (81.1, 82.7) | 10 (7, 15) |
| **Johnson** | 78.1 (77.8, 78.3) | -3 (-4, -2) | 78.9 (78.6, 79.1) | 0 (-1, 1) | 82.1 (81.8, 82.3) | 3 (2, 4) | 82.6 (82.3, 82.8) | 8 (7, 9) |
| **Kearny** | 73.7 (72.9, 74.3) | 15 (12, 19) | 74.6 (73.8, 75.2) | 18 (15, 22) | 79.7 (79, 80.3) | 16 (12, 19) | 79.4 (78.6, 80.1) | 24 (21, 28) |
| **Kingman** | 73.7 (72.9, 74.3) | 15 (12, 19) | 75.3 (74.5, 75.9) | 15 (12, 18) | 80.2 (79.6, 80.8) | 13 (9, 16) | 80.1 (79.4, 80.8) | 20 (16, 24) |
| **Kiowa** | 73.6 (73.1, 74.1) | 15 (13, 18) | 75.4 (74.8, 75.9) | 14 (12, 17) | 79.8 (79.2, 80.3) | 15 (12, 18) | 80 (79.4, 80.5) | 21 (18, 25) |
| **Labette** | 73.2 (72.5, 73.7) | 17 (15, 21) | 73.8 (73, 74.4) | 22 (19, 25) | 79.8 (79.2, 80.4) | 15 (12, 18) | 79 (78.3, 79.6) | 26 (23, 29) |
| **Lane** | 74.7 (74, 75.3) | 10 (7, 14) | 75.9 (75.1, 76.5) | 12 (9, 16) | 80.7 (80, 81.4) | 10 (6, 14) | 81.2 (80.3, 82) | 14 (10, 19) |
| **Leavenworth** | 74.7 (74.2, 75) | 11 (9, 13) | 75.2 (74.7, 75.5) | 15 (13, 17) | 79.9 (79.4, 80.3) | 15 (12, 17) | 80 (79.5, 80.4) | 21 (18, 24) |
| **Lincoln** | 74.4 (73.6, 74.9) | 12 (9, 15) | 75.2 (74.4, 75.8) | 15 (12, 19) | 80.9 (80.2, 81.5) | 9 (6, 13) | 80.7 (79.9, 81.4) | 17 (13, 22) |
| **Linn** | 73.7 (73, 74.2) | 15 (13, 18) | 74.7 (74, 75.3) | 17 (14, 21) | 79.5 (78.8, 80.1) | 17 (14, 20) | 79.4 (78.7, 80.1) | 24 (21, 27) |
| **Logan** | 75.1 (74.4, 75.6) | 8 (6, 12) | 76.3 (75.6, 76.9) | 10 (8, 13) | 81 (80.3, 81.5) | 8 (6, 12) | 81.5 (80.7, 82.1) | 13 (10, 17) |
| **Lyon** | 74.2 (73.6, 74.7) | 12 (10, 15) | 75.5 (74.8, 76.1) | 13 (11, 17) | 80.5 (79.8, 81.1) | 11 (8, 15) | 79.5 (78.8, 80.1) | 24 (20, 27) |
| **Marion** | 74.7 (74, 75.2) | 10 (8, 13) | 75.1 (74.4, 75.6) | 15 (13, 19) | 80.8 (80.2, 81.4) | 9 (6, 13) | 81.2 (80.4, 81.9) | 14 (11, 19) |
| **Marshall** | 75.2 (74.5, 75.7) | 8 (6, 11) | 76.3 (75.5, 76.9) | 10 (8, 13) | 80.9 (80.2, 81.4) | 9 (6, 13) | 81.7 (80.9, 82.3) | 12 (9, 16) |
| **McPherson** | 76 (75.4, 76.5) | 4 (2, 7) | 76.7 (76, 77.3) | 9 (7, 11) | 80.7 (80.1, 81.3) | 10 (7, 14) | 81.3 (80.5, 81.9) | 14 (11, 18) |
| **Meade** | 73.6 (73.1, 74.1) | 15 (13, 18) | 75.4 (74.8, 75.9) | 14 (12, 17) | 79.8 (79.2, 80.3) | 15 (12, 18) | 80 (79.4, 80.5) | 21 (18, 25) |
| **Miami** | 75 (74.3, 75.5) | 9 (7, 12) | 75.9 (75.2, 76.4) | 12 (10, 15) | 80.1 (79.4, 80.7) | 14 (10, 17) | 80.2 (79.5, 80.8) | 20 (16, 24) |
| **Mitchell** | 75 (74.5, 75.4) | 9 (7, 11) | 75.5 (75, 76) | 13 (11, 16) | 80.4 (79.9, 80.8) | 12 (9, 15) | 80.1 (79.5, 80.5) | 21 (18, 24) |
| **Montgomery** | 73.4 (72.8, 73.8) | 17 (14, 20) | 73 (72.4, 73.5) | 25 (23, 28) | 79.5 (79, 80) | 17 (14, 19) | 79.9 (79.3, 80.5) | 21 (18, 25) |
| **Morris** | 74.3 (73.5, 74.8) | 12 (10, 16) | 74.7 (73.9, 75.3) | 17 (14, 21) | 80.3 (79.5, 80.9) | 12 (9, 17) | 80.4 (79.6, 81.1) | 18 (15, 23) |
| **Morton** | 73.7 (72.9, 74.3) | 15 (12, 19) | 74.6 (73.8, 75.2) | 18 (15, 22) | 79.7 (79, 80.3) | 16 (12, 19) | 79.4 (78.6, 80.1) | 24 (21, 28) |
| **Nemaha** | 74.7 (74, 75.2) | 11 (8, 14) | 75.1 (74.5, 75.7) | 15 (12, 18) | 80.9 (80.2, 81.5) | 9 (6, 13) | 80.9 (80.2, 81.6) | 16 (12, 20) |
| **Neosho** | 73.5 (72.8, 74.1) | 16 (13, 19) | 74 (73.3, 74.6) | 21 (18, 24) | 80 (79.3, 80.5) | 14 (11, 18) | 79.6 (78.8, 80.2) | 23 (20, 27) |
| **Ness** | 74.8 (74.1, 75.3) | 10 (7, 13) | 75.2 (74.4, 75.7) | 15 (12, 19) | 80.6 (79.9, 81.2) | 10 (7, 15) | 80.6 (79.8, 81.3) | 17 (14, 22) |
| **Norton** | 75.1 (74.4, 75.6) | 8 (6, 12) | 76.3 (75.6, 76.9) | 10 (8, 13) | 81 (80.3, 81.5) | 8 (6, 12) | 81.5 (80.7, 82.1) | 13 (10, 17) |
| **Osage** | 74.7 (74, 75.3) | 10 (7, 13) | 75.3 (74.6, 76) | 14 (11, 18) | 80.4 (79.7, 81.1) | 12 (8, 16) | 80.4 (79.6, 81.2) | 18 (14, 23) |
| **Osborne** | 74.7 (74, 75.3) | 10 (7, 14) | 75.7 (74.9, 76.3) | 13 (10, 16) | 80.5 (79.8, 81) | 11 (8, 15) | 82 (81.1, 82.7) | 10 (7, 15) |
| **Ottawa** | 75 (74.5, 75.4) | 9 (7, 11) | 75.5 (75, 76) | 13 (11, 16) | 80.4 (79.9, 80.8) | 12 (9, 15) | 80.1 (79.5, 80.5) | 21 (18, 24) |
| **Pawnee** | 74.8 (74.1, 75.3) | 10 (7, 13) | 75.2 (74.4, 75.7) | 15 (12, 19) | 80.6 (79.9, 81.2) | 10 (7, 15) | 80.6 (79.8, 81.3) | 17 (14, 22) |
| **Phillips** | 75.1 (74.4, 75.6) | 8 (6, 12) | 76.3 (75.6, 76.9) | 10 (8, 13) | 81 (80.3, 81.5) | 8 (6, 12) | 81.5 (80.7, 82.1) | 13 (10, 17) |
| **Pottawatomie** | 74.9 (74.2, 75.4) | 10 (7, 13) | 75.8 (75.2, 76.4) | 12 (10, 15) | 80.5 (79.9, 81.1) | 11 (8, 15) | 80.8 (80.1, 81.5) | 16 (13, 20) |
| **Pratt** | 74.9 (74.4, 75.2) | 9 (8, 12) | 75.4 (74.9, 75.8) | 14 (12, 16) | 80.5 (80, 80.9) | 11 (9, 14) | 80.6 (80, 81) | 17 (15, 21) |
| **Rawlins** | 74.9 (74.2, 75.4) | 9 (7, 13) | 76 (75.2, 76.5) | 11 (9, 15) | 81.7 (81, 82.3) | 5 (2, 8) | 80.6 (79.8, 81.2) | 18 (14, 22) |
| **Reno** | 74.9 (74.4, 75.2) | 9 (8, 12) | 75.4 (74.9, 75.8) | 14 (12, 16) | 80.5 (80, 80.9) | 11 (9, 14) | 80.6 (80, 81) | 17 (15, 21) |
| **Republic** | 75.2 (74.5, 75.7) | 8 (6, 11) | 76.3 (75.5, 76.9) | 10 (8, 13) | 80.9 (80.2, 81.4) | 9 (6, 13) | 81.7 (80.9, 82.3) | 12 (9, 16) |
| **Rice** | 74.4 (73.7, 74.9) | 12 (9, 15) | 74.9 (74.1, 75.6) | 16 (13, 20) | 80.3 (79.5, 80.8) | 13 (9, 17) | 80.1 (79.3, 80.8) | 20 (16, 25) |
| **Riley** | 76.8 (76.2, 77.2) | 1 (0, 4) | 78.1 (77.5, 78.6) | 4 (1, 6) | 81 (80.4, 81.5) | 9 (6, 12) | 81.9 (81.2, 82.6) | 11 (8, 14) |
| **Rooks** | 74.7 (74, 75.3) | 10 (7, 14) | 75.7 (74.9, 76.3) | 13 (10, 16) | 80.5 (79.8, 81) | 11 (8, 15) | 82 (81.1, 82.7) | 10 (7, 15) |
| **Rush** | 74.8 (74.1, 75.3) | 10 (7, 13) | 75.2 (74.4, 75.7) | 15 (12, 19) | 80.6 (79.9, 81.2) | 10 (7, 15) | 80.6 (79.8, 81.3) | 17 (14, 22) |
| **Russell** | 74.4 (73.6, 74.9) | 12 (9, 15) | 75.2 (74.4, 75.8) | 15 (12, 19) | 80.9 (80.2, 81.5) | 9 (6, 13) | 80.7 (79.9, 81.4) | 17 (13, 22) |
| **Saline** | 75 (74.5, 75.4) | 9 (7, 11) | 75.5 (75, 76) | 13 (11, 16) | 80.4 (79.9, 80.8) | 12 (9, 15) | 80.1 (79.5, 80.5) | 21 (18, 24) |
| **Scott** | 74.7 (74, 75.3) | 10 (7, 14) | 75.9 (75.1, 76.5) | 12 (9, 16) | 80.7 (80, 81.4) | 10 (6, 14) | 81.2 (80.3, 82) | 14 (10, 19) |
| **Sedgwick** | 74 (73.8, 74.2) | 14 (13, 14) | 74.3 (74.1, 74.5) | 19 (18, 20) | 79.7 (79.4, 79.9) | 16 (15, 17) | 79.8 (79.6, 80) | 22 (21, 23) |
| **Seward** | 72.9 (72.2, 73.5) | 19 (16, 22) | 73.3 (72.6, 73.9) | 24 (21, 27) | 78.6 (77.9, 79.2) | 21 (18, 23) | 79.2 (78.3, 79.9) | 25 (21, 29) |
| **Shawnee** | 73.4 (73.1, 73.7) | 16 (15, 18) | 74.6 (74.2, 74.9) | 18 (16, 19) | 79.8 (79.4, 80.1) | 15 (14, 17) | 80.4 (80, 80.7) | 19 (17, 21) |
| **Sheridan** | 75.1 (74.4, 75.6) | 8 (6, 12) | 76.3 (75.6, 76.9) | 10 (8, 13) | 81 (80.3, 81.5) | 8 (6, 12) | 81.5 (80.7, 82.1) | 13 (10, 17) |
| **Sherman** | 74.9 (74.2, 75.4) | 9 (7, 13) | 76 (75.2, 76.5) | 11 (9, 15) | 81.7 (81, 82.3) | 5 (2, 8) | 80.6 (79.8, 81.2) | 18 (14, 22) |
| **Smith** | 74.7 (74, 75.3) | 10 (7, 14) | 75.7 (74.9, 76.3) | 13 (10, 16) | 80.5 (79.8, 81) | 11 (8, 15) | 82 (81.1, 82.7) | 10 (7, 15) |
| **Stafford** | 74.4 (73.7, 74.9) | 12 (9, 15) | 74.9 (74.1, 75.6) | 16 (13, 20) | 80.3 (79.5, 80.8) | 13 (9, 17) | 80.1 (79.3, 80.8) | 20 (16, 25) |
| **Stanton** | 74.7 (74, 75.3) | 10 (7, 14) | 75.9 (75.1, 76.5) | 12 (9, 16) | 80.7 (80, 81.4) | 10 (6, 14) | 81.2 (80.3, 82) | 14 (10, 19) |
| **Stevens** | 73.7 (72.9, 74.3) | 15 (12, 19) | 74.6 (73.8, 75.2) | 18 (15, 22) | 79.7 (79, 80.3) | 16 (12, 19) | 79.4 (78.6, 80.1) | 24 (21, 28) |
| **Sumner** | 73.9 (73.2, 74.4) | 14 (12, 17) | 75 (74.3, 75.6) | 16 (13, 19) | 80.2 (79.5, 80.8) | 13 (9, 17) | 79.7 (79, 80.3) | 23 (19, 26) |
| **Thomas** | 74.9 (74.2, 75.4) | 9 (7, 13) | 76 (75.2, 76.5) | 11 (9, 15) | 81.7 (81, 82.3) | 5 (2, 8) | 80.6 (79.8, 81.2) | 18 (14, 22) |
| **Trego** | 75.1 (74.4, 75.6) | 8 (6, 12) | 76.3 (75.6, 76.9) | 10 (8, 13) | 81 (80.3, 81.5) | 8 (6, 12) | 81.5 (80.7, 82.1) | 13 (10, 17) |
| **Wabaunsee** | 74.7 (74, 75.3) | 10 (7, 13) | 75.3 (74.6, 76) | 14 (11, 18) | 80.4 (79.7, 81.1) | 12 (8, 16) | 80.4 (79.6, 81.2) | 18 (14, 23) |
| **Wallace** | 74.7 (74, 75.3) | 10 (7, 14) | 75.9 (75.1, 76.5) | 12 (9, 16) | 80.7 (80, 81.4) | 10 (6, 14) | 81.2 (80.3, 82) | 14 (10, 19) |
| **Washington** | 75.2 (74.5, 75.7) | 8 (6, 11) | 76.3 (75.5, 76.9) | 10 (8, 13) | 80.9 (80.2, 81.4) | 9 (6, 13) | 81.7 (80.9, 82.3) | 12 (9, 16) |
| **Wichita** | 74.7 (74, 75.3) | 10 (7, 14) | 75.9 (75.1, 76.5) | 12 (9, 16) | 80.7 (80, 81.4) | 10 (6, 14) | 81.2 (80.3, 82) | 14 (10, 19) |
| **Wilson** | 73.5 (72.8, 74.1) | 16 (13, 19) | 74 (73.3, 74.6) | 21 (18, 24) | 80 (79.3, 80.5) | 14 (11, 18) | 79.6 (78.8, 80.2) | 23 (20, 27) |
| **Woodson** | 73.5 (72.8, 74.1) | 16 (13, 19) | 74 (73.3, 74.6) | 21 (18, 24) | 80 (79.3, 80.5) | 14 (11, 18) | 79.6 (78.8, 80.2) | 23 (20, 27) |
| **Wyandotte** | 69.9 (69.6, 70.2) | 35 (32, 39) | 71.1 (70.7, 71.3) | 34 (33, 35) | 76.8 (76.5, 77.1) | 27 (26, 29) | 77.5 (77.2, 77.8) | 32 (31, 33) |
| **KENTUCKY** |  |  |  |  |  |  |  |  |
| **Adair** | 71.6 (70.8, 72.1) | 25 (22, 28) | 72.6 (71.8, 73.1) | 28 (25, 31) | 78.5 (77.9, 79.2) | 21 (18, 24) | 78.1 (77.4, 78.8) | 30 (27, 32) |
| **Allen** | 71.3 (70.6, 71.8) | 26 (24, 29) | 71.5 (70.8, 72.1) | 32 (30, 35) | 78 (77.4, 78.5) | 23 (21, 25) | 77.9 (77.2, 78.5) | 30 (28, 33) |
| **Anderson** | 73.6 (72.9, 74.1) | 15 (13, 19) | 75 (74.3, 75.6) | 16 (13, 19) | 79.2 (78.5, 79.8) | 18 (15, 21) | 79 (78.3, 79.7) | 26 (23, 29) |
| **Ballard** | 72.8 (72.4, 73.1) | 19 (18, 21) | 73.6 (73.1, 73.9) | 23 (21, 25) | 78.8 (78.4, 79.1) | 20 (19, 22) | 79.1 (78.7, 79.5) | 26 (24, 27) |
| **Barren** | 72.8 (72.1, 73.3) | 19 (17, 22) | 73.4 (72.7, 73.9) | 23 (21, 27) | 79.1 (78.5, 79.6) | 19 (16, 21) | 78.6 (78, 79.2) | 28 (25, 30) |
| **Bath** | 71.5 (70.8, 72) | 25 (23, 28) | 71 (70.3, 71.6) | 34 (32, 38) | 77.8 (77.2, 78.3) | 24 (22, 26) | 77.5 (76.8, 78) | 32 (30, 35) |
| **Bell** | 69.9 (69.2, 70.4) | 35 (30, 44) | 69.3 (68.5, 69.8) | 51 (43, 51) | 76.7 (76.1, 77.2) | 28 (26, 31) | 75.9 (75.2, 76.4) | 39 (36, 43) |
| **Boone** | 75 (74.5, 75.5) | 9 (7, 11) | 75.4 (74.9, 75.8) | 14 (12, 17) | 79.6 (79.1, 80.1) | 16 (14, 19) | 79.6 (79.1, 80) | 23 (21, 26) |
| **Bourbon** | 73 (72.3, 73.4) | 19 (16, 22) | 74 (73.2, 74.5) | 21 (18, 24) | 78.9 (78.3, 79.5) | 19 (17, 22) | 79.2 (78.4, 79.7) | 25 (22, 28) |
| **Boyd** | 72.4 (72, 72.9) | 21 (19, 23) | 72.5 (72, 73) | 28 (25, 30) | 78.3 (77.8, 78.7) | 22 (20, 24) | 78.2 (77.7, 78.7) | 29 (27, 31) |
| **Boyle** | 73 (72.3, 73.4) | 18 (16, 21) | 73.9 (73.2, 74.4) | 21 (19, 24) | 79.1 (78.5, 79.5) | 19 (17, 21) | 79 (78.2, 79.5) | 26 (24, 29) |
| **Bracken** | 72.3 (71.6, 72.8) | 22 (19, 25) | 72.3 (71.6, 72.9) | 29 (26, 32) | 78.6 (78, 79.2) | 21 (18, 23) | 78.5 (77.8, 79.2) | 28 (25, 31) |
| **Breathitt** | 69.1 (68.4, 69.7) | 45 (38, 50) | 68.2 (67.3, 68.8) | 51 (51, 51) | 77.4 (76.7, 78) | 25 (23, 28) | 76.1 (75.3, 76.7) | 38 (35, 43) |
| **Breckinridge** | 72.6 (71.9, 73.1) | 20 (18, 23) | 72.8 (72.1, 73.3) | 26 (24, 29) | 79 (78.3, 79.5) | 19 (17, 22) | 78.9 (78.1, 79.5) | 27 (24, 30) |
| **Bullitt** | 73.8 (73.3, 74.3) | 14 (12, 17) | 75.4 (74.8, 75.9) | 14 (12, 17) | 79 (78.4, 79.5) | 19 (17, 21) | 80.2 (79.5, 80.8) | 20 (16, 24) |
| **Butler** | 71.7 (70.9, 72.2) | 24 (22, 28) | 72.8 (72, 73.4) | 26 (23, 30) | 78.7 (78, 79.3) | 20 (18, 23) | 77.8 (77.1, 78.4) | 31 (28, 34) |
| **Caldwell** | 72.7 (72, 73.1) | 20 (18, 23) | 73 (72.3, 73.5) | 25 (23, 29) | 79 (78.3, 79.5) | 19 (17, 22) | 79.3 (78.6, 79.8) | 25 (22, 28) |
| **Calloway** | 74 (73.5, 74.5) | 13 (11, 16) | 74.7 (74.1, 75.3) | 17 (15, 20) | 79.6 (79, 80.1) | 16 (14, 19) | 79.7 (79.1, 80.2) | 23 (20, 26) |
| **Campbell** | 73.2 (72.8, 73.6) | 17 (15, 19) | 73.7 (73.2, 74.1) | 22 (20, 24) | 79 (78.6, 79.4) | 19 (17, 21) | 79.6 (79.1, 80) | 23 (21, 26) |
| **Carlisle** | 72.8 (72.4, 73.1) | 19 (18, 21) | 73.6 (73.1, 73.9) | 23 (21, 25) | 78.8 (78.4, 79.1) | 20 (19, 22) | 79.1 (78.7, 79.5) | 26 (24, 27) |
| **Carroll** | 72.1 (71.3, 72.6) | 23 (20, 26) | 72.8 (72, 73.4) | 26 (23, 30) | 78.2 (77.5, 78.8) | 22 (20, 25) | 78.6 (77.8, 79.2) | 28 (25, 31) |
| **Carter** | 71.3 (70.5, 71.8) | 26 (24, 30) | 70.8 (69.9, 71.4) | 35 (32, 42) | 78.6 (77.8, 79.3) | 21 (18, 24) | 77.5 (76.7, 78.2) | 32 (29, 35) |
| **Casey** | 70.4 (69.6, 71) | 31 (27, 41) | 71.1 (70.3, 71.8) | 34 (31, 38) | 78.3 (77.6, 78.9) | 22 (20, 24) | 78.2 (77.3, 78.9) | 29 (27, 33) |
| **Christian** | 72.1 (71.7, 72.4) | 22 (21, 24) | 72.7 (72.3, 73.1) | 27 (25, 29) | 78.6 (78.1, 78.9) | 21 (20, 23) | 78.6 (78, 79) | 28 (26, 30) |
| **Clark** | 73.3 (72.7, 73.8) | 17 (14, 20) | 74.1 (73.3, 74.7) | 20 (17, 24) | 79.1 (78.5, 79.6) | 19 (16, 21) | 78.8 (78.1, 79.3) | 27 (25, 30) |
| **Clay** | 69.6 (69, 70.1) | 40 (33, 46) | 69.2 (68.6, 69.7) | 51 (45, 51) | 76.9 (76.2, 77.4) | 27 (25, 30) | 76.1 (75.4, 76.6) | 38 (35, 42) |
| **Clinton** | 70.4 (69.6, 70.9) | 31 (27, 40) | 70.2 (69.3, 70.8) | 39 (35, 51) | 77.8 (77.1, 78.4) | 24 (22, 26) | 77.3 (76.5, 77.9) | 33 (30, 36) |
| **Crittenden** | 72.3 (71.6, 72.7) | 22 (20, 25) | 72.3 (71.6, 72.9) | 29 (26, 32) | 78.7 (78, 79.2) | 21 (18, 23) | 77.9 (77.2, 78.4) | 31 (28, 33) |
| **Cumberland** | 70.4 (69.6, 70.9) | 31 (27, 40) | 70.2 (69.3, 70.8) | 39 (35, 51) | 77.8 (77.1, 78.4) | 24 (22, 26) | 77.3 (76.5, 77.9) | 33 (30, 36) |
| **Daviess** | 73.2 (72.7, 73.5) | 17 (16, 20) | 74.3 (73.8, 74.6) | 19 (18, 22) | 79.4 (78.9, 79.8) | 17 (15, 19) | 79.7 (79.2, 80.1) | 23 (21, 25) |
| **Edmonson** | 71.7 (70.9, 72.2) | 24 (22, 28) | 72.8 (72, 73.4) | 26 (23, 30) | 78.7 (78, 79.3) | 20 (18, 23) | 77.8 (77.1, 78.4) | 31 (28, 34) |
| **Elliott** | 70.2 (69.4, 70.7) | 32 (28, 43) | 70.2 (69.3, 70.8) | 39 (35, 51) | 77.4 (76.7, 78) | 25 (23, 28) | 77.3 (76.4, 77.9) | 33 (30, 36) |
| **Estill** | 70.2 (69.4, 70.7) | 32 (28, 43) | 70.3 (69.5, 70.9) | 38 (35, 48) | 78 (77.3, 78.6) | 23 (21, 25) | 76.9 (76.2, 77.6) | 34 (31, 37) |
| **Fayette** | 73.9 (73.6, 74.2) | 14 (13, 15) | 75.4 (75.1, 75.7) | 14 (13, 16) | 79.1 (78.8, 79.4) | 19 (17, 20) | 80.3 (80, 80.6) | 19 (18, 21) |
| **Fleming** | 71.5 (70.8, 72) | 25 (23, 28) | 71 (70.3, 71.6) | 34 (32, 38) | 77.8 (77.2, 78.3) | 24 (22, 26) | 77.5 (76.8, 78) | 32 (30, 35) |
| **Floyd** | 69.8 (69.1, 70.3) | 37 (31, 45) | 69.7 (69.1, 70.3) | 44 (38, 51) | 76.7 (76.2, 77.2) | 28 (26, 30) | 76.2 (75.6, 76.8) | 37 (35, 40) |
| **Franklin** | 72.9 (72.4, 73.3) | 19 (17, 21) | 74.3 (73.7, 74.7) | 19 (17, 22) | 78.8 (78.3, 79.2) | 20 (18, 22) | 78.7 (78.2, 79.1) | 28 (26, 29) |
| **Fulton** | 72.4 (71.9, 72.8) | 21 (19, 23) | 72.6 (72, 73) | 27 (25, 30) | 79 (78.5, 79.4) | 19 (17, 21) | 78.2 (77.6, 78.6) | 29 (28, 31) |
| **Gallatin** | 71.8 (71.1, 72.3) | 24 (22, 27) | 72.6 (71.9, 73.1) | 27 (25, 30) | 78.6 (77.9, 79.2) | 21 (18, 23) | 78.7 (77.9, 79.4) | 27 (24, 30) |
| **Garrard** | 72.9 (72.1, 73.5) | 19 (16, 23) | 74.5 (73.6, 75.1) | 18 (15, 22) | 78.9 (78.2, 79.6) | 20 (16, 22) | 78.8 (77.9, 79.5) | 27 (24, 30) |
| **Grant** | 71.8 (71.1, 72.3) | 24 (22, 27) | 72.6 (71.9, 73.1) | 27 (25, 30) | 78.6 (77.9, 79.2) | 21 (18, 23) | 78.7 (77.9, 79.4) | 27 (24, 30) |
| **Graves** | 72.4 (71.9, 72.8) | 21 (19, 23) | 72.6 (72, 73) | 27 (25, 30) | 79 (78.5, 79.4) | 19 (17, 21) | 78.2 (77.6, 78.6) | 29 (28, 31) |
| **Grayson** | 71.5 (70.8, 72) | 25 (23, 28) | 71.9 (71.2, 72.5) | 30 (28, 33) | 78.1 (77.4, 78.7) | 23 (20, 25) | 77.8 (77, 78.4) | 31 (29, 34) |
| **Green** | 71.6 (70.8, 72.1) | 25 (23, 28) | 71.9 (71.1, 72.4) | 30 (28, 34) | 79.2 (78.5, 79.8) | 18 (15, 21) | 77.9 (77.1, 78.5) | 30 (28, 33) |
| **Greenup** | 72.9 (72.3, 73.5) | 19 (16, 22) | 73.3 (72.5, 73.9) | 24 (21, 28) | 78.2 (77.7, 78.8) | 22 (20, 24) | 78.5 (77.9, 79) | 28 (26, 31) |
| **Hancock** | 72.6 (71.9, 73.1) | 20 (18, 23) | 72.8 (72.1, 73.3) | 26 (24, 29) | 79 (78.3, 79.5) | 19 (17, 22) | 78.9 (78.1, 79.5) | 27 (24, 30) |
| **Hardin** | 73.6 (73.2, 74) | 15 (14, 17) | 75.2 (74.7, 75.6) | 15 (13, 17) | 79.4 (78.9, 79.8) | 17 (15, 20) | 80.2 (79.6, 80.6) | 20 (17, 23) |
| **Harlan** | 69.2 (68.5, 69.8) | 44 (37, 49) | 68.4 (67.6, 69) | 51 (51, 51) | 77.2 (76.6, 77.7) | 26 (24, 28) | 76.2 (75.5, 76.8) | 37 (35, 41) |
| **Harrison** | 72.6 (71.9, 73) | 21 (18, 23) | 72.5 (71.8, 73) | 28 (25, 31) | 79.1 (78.4, 79.6) | 19 (16, 22) | 78 (77.3, 78.5) | 30 (28, 33) |
| **Hart** | 71.8 (71.1, 72.3) | 24 (22, 27) | 72.4 (71.7, 72.9) | 28 (26, 31) | 78.5 (77.9, 78.9) | 21 (19, 24) | 78.2 (77.5, 78.8) | 29 (27, 32) |
| **Henderson** | 73.4 (72.8, 73.8) | 16 (14, 19) | 73.6 (72.9, 74.1) | 22 (20, 26) | 78.8 (78.2, 79.3) | 20 (18, 22) | 78.7 (78.1, 79.2) | 27 (25, 30) |
| **Henry** | 72.2 (71.4, 72.7) | 22 (20, 25) | 73 (72.2, 73.6) | 25 (23, 29) | 78.6 (78, 79.2) | 21 (18, 23) | 78.9 (78.2, 79.6) | 26 (23, 29) |
| **Hickman** | 72.4 (71.9, 72.8) | 21 (19, 23) | 72.6 (72, 73) | 27 (25, 30) | 79 (78.5, 79.4) | 19 (17, 21) | 78.2 (77.6, 78.6) | 29 (28, 31) |
| **Hopkins** | 72.4 (71.9, 72.8) | 21 (19, 23) | 72.3 (71.7, 72.7) | 29 (27, 31) | 78.6 (78.1, 79) | 21 (19, 23) | 78.1 (77.5, 78.5) | 30 (28, 32) |
| **Jackson** | 70.2 (69.4, 70.7) | 32 (28, 43) | 70.3 (69.5, 70.9) | 38 (35, 48) | 78 (77.3, 78.6) | 23 (21, 25) | 76.9 (76.2, 77.6) | 34 (31, 37) |
| **Jefferson** | 72.4 (72.3, 72.6) | 21 (20, 22) | 73.4 (73.3, 73.6) | 23 (22, 24) | 78.3 (78.2, 78.5) | 22 (21, 22) | 79.2 (79.1, 79.4) | 25 (24, 26) |
| **Jessamine** | 73.2 (72.6, 73.6) | 17 (15, 20) | 74.3 (73.7, 74.8) | 19 (17, 22) | 79.7 (79.1, 80.3) | 16 (12, 19) | 79.5 (78.8, 80.1) | 24 (21, 27) |
| **Johnson** | 70.5 (69.9, 70.9) | 30 (27, 36) | 69.7 (68.9, 70.2) | 46 (39, 51) | 77.4 (76.8, 77.9) | 25 (23, 28) | 77.4 (76.7, 78.1) | 32 (30, 35) |
| **Kenton** | 73.4 (73, 73.7) | 16 (15, 18) | 73.5 (73.1, 73.9) | 23 (21, 25) | 79.6 (79.2, 79.9) | 16 (14, 18) | 78.6 (78.2, 78.9) | 28 (27, 29) |
| **Knott** | 70.6 (69.8, 71.2) | 29 (26, 37) | 69.7 (68.8, 70.4) | 44 (37, 51) | 78.1 (77.3, 78.7) | 23 (20, 26) | 77.6 (76.7, 78.3) | 31 (29, 35) |
| **Knox** | 70.3 (69.7, 70.9) | 31 (27, 39) | 69.9 (69.1, 70.5) | 43 (37, 51) | 77.8 (77.2, 78.3) | 24 (22, 26) | 76.4 (75.7, 77) | 36 (34, 40) |
| **Larue** | 71.8 (71.1, 72.3) | 24 (22, 27) | 72.4 (71.7, 72.9) | 28 (26, 31) | 78.5 (77.9, 78.9) | 21 (19, 24) | 78.2 (77.5, 78.8) | 29 (27, 32) |
| **Laurel** | 72.2 (71.6, 72.7) | 22 (20, 24) | 72 (71.4, 72.5) | 30 (28, 32) | 78.9 (78.3, 79.4) | 20 (17, 22) | 77.7 (77.1, 78.2) | 31 (29, 33) |
| **Lawrence** | 70.2 (69.4, 70.7) | 32 (28, 43) | 70.2 (69.3, 70.8) | 39 (35, 51) | 77.4 (76.7, 78) | 25 (23, 28) | 77.3 (76.4, 77.9) | 33 (30, 36) |
| **Lee** | 69.1 (68.3, 69.7) | 45 (38, 51) | 68.2 (67.2, 68.8) | 51 (51, 51) | 77.2 (76.4, 77.8) | 26 (24, 29) | 76.2 (75.3, 76.9) | 37 (34, 43) |
| **Leslie** | 69.6 (69, 70.1) | 40 (33, 46) | 69.2 (68.6, 69.7) | 51 (45, 51) | 76.9 (76.2, 77.4) | 27 (25, 30) | 76.1 (75.4, 76.6) | 38 (35, 42) |
| **Letcher** | 70.1 (69.3, 70.6) | 33 (29, 44) | 69.4 (68.5, 70.1) | 49 (40, 51) | 76.7 (76, 77.2) | 28 (26, 31) | 76.8 (76, 77.4) | 35 (32, 38) |
| **Lewis** | 70 (69.2, 70.7) | 33 (29, 45) | 69.8 (68.8, 70.5) | 44 (37, 51) | 77.4 (76.6, 78) | 25 (23, 28) | 77.2 (76.3, 78) | 33 (30, 37) |
| **Lincoln** | 71.6 (70.9, 72.1) | 25 (22, 28) | 71.7 (70.9, 72.2) | 31 (29, 34) | 78.3 (77.7, 78.9) | 22 (20, 24) | 78.2 (77.5, 78.9) | 29 (27, 32) |
| **Livingston** | 74.1 (73.5, 74.6) | 13 (11, 16) | 73.8 (73.1, 74.3) | 22 (19, 25) | 79.7 (79.1, 80.2) | 16 (13, 19) | 79.6 (79, 80.2) | 23 (20, 26) |
| **Logan** | 72.1 (71.5, 72.5) | 23 (21, 25) | 72.5 (71.8, 72.9) | 28 (26, 31) | 78.4 (77.8, 78.8) | 22 (20, 24) | 78.1 (77.4, 78.6) | 30 (28, 32) |
| **Lyon** | 72.3 (71.6, 72.7) | 22 (20, 25) | 72.3 (71.6, 72.9) | 29 (26, 32) | 78.7 (78, 79.2) | 21 (18, 23) | 77.9 (77.2, 78.4) | 31 (28, 33) |
| **Madison** | 73.3 (72.8, 73.7) | 17 (15, 19) | 73.8 (73.3, 74.2) | 22 (20, 24) | 79 (78.5, 79.4) | 19 (17, 21) | 79.5 (78.9, 79.9) | 24 (21, 27) |
| **Magoffin** | 70 (69.3, 70.5) | 34 (30, 44) | 70.8 (70.1, 71.4) | 35 (32, 40) | 77.3 (76.7, 77.9) | 26 (23, 28) | 76.9 (76.1, 77.6) | 34 (32, 38) |
| **Marion** | 72.3 (71.7, 72.7) | 22 (20, 24) | 73.3 (72.7, 73.9) | 24 (21, 27) | 78.8 (78.2, 79.4) | 20 (18, 22) | 79.6 (78.8, 80.2) | 23 (20, 27) |
| **Marshall** | 74.1 (73.5, 74.6) | 13 (11, 16) | 73.8 (73.1, 74.3) | 22 (19, 25) | 79.7 (79.1, 80.2) | 16 (13, 19) | 79.6 (79, 80.2) | 23 (20, 26) |
| **Martin** | 70.5 (69.9, 70.9) | 30 (27, 36) | 69.7 (68.9, 70.2) | 46 (39, 51) | 77.4 (76.8, 77.9) | 25 (23, 28) | 77.4 (76.7, 78.1) | 32 (30, 35) |
| **Mason** | 71.9 (71.1, 72.4) | 23 (21, 27) | 72.9 (72.1, 73.5) | 26 (23, 30) | 78.1 (77.4, 78.6) | 23 (21, 25) | 79.1 (78.3, 79.7) | 26 (23, 29) |
| **McCracken** | 72.8 (72.4, 73.1) | 19 (18, 21) | 73.6 (73.1, 73.9) | 23 (21, 25) | 78.8 (78.4, 79.1) | 20 (19, 22) | 79.1 (78.7, 79.5) | 26 (24, 27) |
| **McCreary** | 69.4 (68.5, 70) | 43 (34, 49) | 69 (68.1, 69.7) | 51 (46, 51) | 76.1 (75.4, 76.7) | 31 (28, 35) | 76.2 (75.4, 76.9) | 37 (34, 42) |
| **McLean** | 72.7 (72.1, 73.2) | 20 (17, 23) | 72.4 (71.6, 73) | 28 (26, 32) | 79.1 (78.4, 79.6) | 19 (16, 21) | 78.9 (78.1, 79.5) | 27 (24, 30) |
| **Meade** | 73.3 (72.6, 73.8) | 17 (14, 20) | 74.4 (73.7, 75) | 19 (16, 22) | 79.3 (78.6, 79.9) | 18 (14, 21) | 79.6 (78.8, 80.3) | 23 (19, 27) |
| **Menifee** | 70.4 (69.6, 71) | 30 (27, 40) | 70.8 (69.9, 71.5) | 35 (32, 42) | 77.4 (76.7, 78.1) | 25 (23, 28) | 77.6 (76.7, 78.4) | 31 (28, 35) |
| **Mercer** | 72.7 (72, 73.2) | 20 (17, 23) | 73.2 (72.4, 73.8) | 24 (21, 28) | 79.6 (79, 80.2) | 16 (13, 19) | 79.1 (78.4, 79.7) | 26 (23, 28) |
| **Metcalfe** | 71.6 (70.8, 72.1) | 25 (23, 28) | 71.9 (71.1, 72.4) | 30 (28, 34) | 79.2 (78.5, 79.8) | 18 (15, 21) | 77.9 (77.1, 78.5) | 30 (28, 33) |
| **Monroe** | 71.3 (70.6, 71.8) | 26 (24, 29) | 71.5 (70.8, 72.1) | 32 (30, 35) | 78 (77.4, 78.5) | 23 (21, 25) | 77.9 (77.2, 78.5) | 30 (28, 33) |
| **Montgomery** | 72.9 (72.1, 73.4) | 19 (16, 22) | 72.3 (71.6, 73) | 28 (26, 32) | 78.4 (77.8, 79) | 22 (19, 24) | 79.1 (78.3, 79.8) | 26 (22, 29) |
| **Morgan** | 70 (69.3, 70.5) | 34 (30, 44) | 70.8 (70.1, 71.4) | 35 (32, 40) | 77.3 (76.7, 77.9) | 26 (23, 28) | 76.9 (76.1, 77.6) | 34 (32, 38) |
| **Muhlenberg** | 71.4 (70.8, 71.9) | 25 (23, 28) | 71.6 (71, 72.1) | 32 (30, 34) | 78.4 (77.8, 78.9) | 22 (20, 24) | 77.8 (77.1, 78.3) | 31 (29, 33) |
| **Nelson** | 73.2 (72.6, 73.7) | 17 (15, 20) | 73.9 (73.2, 74.4) | 21 (19, 24) | 79.9 (79.2, 80.4) | 15 (12, 18) | 80.4 (79.7, 81) | 19 (15, 23) |
| **Nicholas** | 72.6 (71.9, 73) | 21 (18, 23) | 72.5 (71.8, 73) | 28 (25, 31) | 79.1 (78.4, 79.6) | 19 (16, 22) | 78 (77.3, 78.5) | 30 (28, 33) |
| **Ohio** | 71.8 (71, 72.3) | 24 (22, 27) | 72.8 (72, 73.3) | 26 (24, 30) | 78.1 (77.4, 78.7) | 23 (21, 25) | 78.3 (77.5, 78.9) | 29 (26, 32) |
| **Oldham** | 75.2 (74.6, 75.7) | 8 (6, 11) | 76.4 (75.8, 76.9) | 10 (8, 12) | 80.4 (79.7, 81) | 12 (8, 16) | 80.7 (80.1, 81.3) | 17 (14, 21) |
| **Owen** | 72.1 (71.3, 72.6) | 23 (20, 26) | 72.8 (72, 73.4) | 26 (23, 30) | 78.2 (77.5, 78.8) | 22 (20, 25) | 78.6 (77.8, 79.2) | 28 (25, 31) |
| **Owsley** | 69.1 (68.4, 69.7) | 45 (38, 50) | 68.2 (67.3, 68.8) | 51 (51, 51) | 77.4 (76.7, 78) | 25 (23, 28) | 76.1 (75.3, 76.7) | 38 (35, 43) |
| **Pendleton** | 72.3 (71.6, 72.8) | 22 (19, 25) | 72.3 (71.6, 72.9) | 29 (26, 32) | 78.6 (78, 79.2) | 21 (18, 23) | 78.5 (77.8, 79.2) | 28 (25, 31) |
| **Perry** | 70.5 (69.8, 71) | 30 (27, 37) | 68.4 (67.6, 68.9) | 51 (51, 51) | 77.3 (76.7, 77.8) | 26 (24, 28) | 76.6 (75.9, 77.2) | 35 (33, 39) |
| **Pike** | 69.7 (69.2, 70.2) | 37 (32, 45) | 68.7 (68.1, 69.1) | 51 (51, 51) | 76.7 (76.2, 77.1) | 28 (26, 30) | 76.3 (75.8, 76.7) | 37 (35, 40) |
| **Powell** | 70.4 (69.6, 71) | 30 (27, 40) | 70.8 (69.9, 71.5) | 35 (32, 42) | 77.4 (76.7, 78.1) | 25 (23, 28) | 77.6 (76.7, 78.4) | 31 (28, 35) |
| **Pulaski** | 72.2 (71.7, 72.7) | 22 (20, 24) | 72.5 (71.9, 73) | 28 (25, 30) | 78.5 (78, 78.9) | 21 (19, 23) | 78.9 (78.4, 79.5) | 27 (24, 29) |
| **Robertson** | 72.3 (71.6, 72.8) | 22 (19, 25) | 72.3 (71.6, 72.9) | 29 (26, 32) | 78.6 (78, 79.2) | 21 (18, 23) | 78.5 (77.8, 79.2) | 28 (25, 31) |
| **Rockcastle** | 71.1 (70.2, 71.7) | 27 (24, 32) | 71.1 (70.2, 71.9) | 34 (30, 39) | 77.9 (77.1, 78.5) | 24 (21, 26) | 77.2 (76.3, 77.9) | 33 (31, 37) |
| **Rowan** | 73 (72.2, 73.6) | 18 (15, 22) | 72.4 (71.6, 73.1) | 28 (25, 31) | 78.8 (78.1, 79.3) | 20 (18, 23) | 79 (78.2, 79.8) | 26 (22, 29) |
| **Russell** | 71.6 (70.8, 72.2) | 25 (22, 28) | 72.5 (71.6, 73.2) | 28 (24, 31) | 78 (77.4, 78.6) | 23 (21, 25) | 78.5 (77.7, 79.3) | 28 (25, 31) |
| **Scott** | 73.9 (73.2, 74.4) | 14 (12, 17) | 74.9 (74.2, 75.4) | 17 (14, 20) | 79.2 (78.6, 79.8) | 18 (15, 21) | 80.4 (79.7, 81.1) | 18 (15, 23) |
| **Shelby** | 73.9 (73.3, 74.4) | 14 (12, 17) | 75.4 (74.7, 76) | 14 (11, 17) | 79.4 (78.8, 79.9) | 17 (14, 20) | 80.2 (79.5, 80.9) | 20 (16, 24) |
| **Simpson** | 72.7 (72, 73.2) | 20 (17, 23) | 73.1 (72.4, 73.6) | 25 (22, 28) | 78.4 (77.8, 79) | 21 (19, 24) | 79.3 (78.5, 79.9) | 25 (22, 28) |
| **Spencer** | 73.6 (72.9, 74.1) | 15 (13, 19) | 75 (74.3, 75.6) | 16 (13, 19) | 79.2 (78.5, 79.8) | 18 (15, 21) | 79 (78.3, 79.7) | 26 (23, 29) |
| **Taylor** | 72 (71.3, 72.4) | 23 (21, 26) | 73.2 (72.4, 73.7) | 24 (22, 28) | 78.5 (77.9, 79) | 21 (19, 24) | 78.7 (78, 79.2) | 27 (25, 30) |
| **Todd** | 72.1 (71.5, 72.5) | 23 (21, 25) | 72.5 (71.8, 72.9) | 28 (26, 31) | 78.4 (77.8, 78.8) | 22 (20, 24) | 78.1 (77.4, 78.6) | 30 (28, 32) |
| **Trigg** | 72.7 (72, 73.1) | 20 (18, 23) | 73 (72.3, 73.5) | 25 (23, 29) | 79 (78.3, 79.5) | 19 (17, 22) | 79.3 (78.6, 79.8) | 25 (22, 28) |
| **Trimble** | 72.2 (71.4, 72.7) | 22 (20, 25) | 73 (72.2, 73.6) | 25 (23, 29) | 78.6 (78, 79.2) | 21 (18, 23) | 78.9 (78.2, 79.6) | 26 (23, 29) |
| **Union** | 72.5 (71.8, 73) | 21 (18, 24) | 72.8 (72.1, 73.4) | 26 (23, 30) | 78.9 (78.2, 79.5) | 20 (17, 22) | 78.8 (78, 79.5) | 27 (24, 30) |
| **Warren** | 73.1 (72.7, 73.4) | 18 (16, 20) | 73.9 (73.4, 74.2) | 21 (20, 23) | 79.6 (79.1, 80) | 16 (14, 19) | 80 (79.4, 80.5) | 21 (18, 24) |
| **Washington** | 72.3 (71.7, 72.7) | 22 (20, 24) | 73.3 (72.7, 73.9) | 24 (21, 27) | 78.8 (78.2, 79.4) | 20 (18, 22) | 79.6 (78.8, 80.2) | 23 (20, 27) |
| **Wayne** | 71.2 (70.5, 71.7) | 26 (24, 30) | 71.6 (70.8, 72.2) | 32 (29, 35) | 77.9 (77.1, 78.4) | 24 (21, 26) | 77.8 (76.9, 78.4) | 31 (29, 34) |
| **Webster** | 72.7 (72.1, 73.2) | 20 (17, 23) | 72.4 (71.6, 73) | 28 (26, 32) | 79.1 (78.4, 79.6) | 19 (16, 21) | 78.9 (78.1, 79.5) | 27 (24, 30) |
| **Whitley** | 70.8 (70.1, 71.3) | 28 (26, 33) | 70.7 (69.9, 71.3) | 36 (33, 42) | 77.8 (77.2, 78.3) | 24 (22, 26) | 77 (76.3, 77.6) | 34 (32, 36) |
| **Wolfe** | 69.1 (68.3, 69.7) | 45 (38, 51) | 68.2 (67.2, 68.8) | 51 (51, 51) | 77.2 (76.4, 77.8) | 26 (24, 29) | 76.2 (75.3, 76.9) | 37 (34, 43) |
| **Woodford** | 74.8 (74.1, 75.2) | 10 (8, 13) | 75.5 (74.8, 76.1) | 13 (11, 17) | 79.8 (79.2, 80.4) | 15 (12, 18) | 80.1 (79.4, 80.7) | 20 (17, 24) |
| **LOUISIANA** |  |  |  |  |  |  |  |  |
| **Acadia** | 70.3 (69.8, 70.6) | 31 (29, 37) | 70 (69.5, 70.4) | 41 (37, 48) | 77.1 (76.7, 77.5) | 26 (25, 28) | 76.7 (76.2, 77.1) | 35 (33, 37) |
| **Allen** | 70.7 (70.2, 71.2) | 28 (26, 32) | 73.1 (72.4, 73.5) | 25 (23, 28) | 77.2 (76.6, 77.6) | 26 (24, 28) | 78.7 (78, 79.3) | 27 (25, 30) |
| **Ascension** | 72.3 (71.9, 72.7) | 22 (20, 23) | 73.3 (72.8, 73.6) | 24 (22, 26) | 78.5 (78, 78.9) | 21 (20, 23) | 78.9 (78.4, 79.3) | 27 (25, 28) |
| **Assumption** | 70.3 (69.6, 70.7) | 31 (29, 40) | 71.1 (70.5, 71.6) | 33 (31, 37) | 76.9 (76.3, 77.4) | 27 (25, 30) | 77.8 (77.1, 78.3) | 31 (29, 33) |
| **Avoyelles** | 69.4 (68.9, 69.8) | 43 (37, 47) | 69.4 (68.9, 69.8) | 49 (43, 51) | 77 (76.5, 77.4) | 27 (25, 29) | 76.3 (75.8, 76.8) | 36 (35, 39) |
| **Beauregard** | 71.8 (71.3, 72.3) | 23 (22, 26) | 72.2 (71.6, 72.7) | 29 (27, 32) | 78.1 (77.5, 78.6) | 23 (21, 25) | 77.7 (77.1, 78.2) | 31 (29, 33) |
| **Bienville** | 69.3 (68.7, 69.7) | 44 (38, 48) | 70 (69.4, 70.5) | 41 (37, 50) | 76.9 (76.3, 77.3) | 27 (26, 30) | 76.1 (75.5, 76.5) | 38 (36, 41) |
| **Bossier** | 73 (72.6, 73.3) | 18 (17, 20) | 73.4 (73, 73.7) | 23 (22, 25) | 78.7 (78.3, 79) | 20 (19, 22) | 79.1 (78.6, 79.4) | 26 (24, 28) |
| **Caddo** | 70.8 (70.6, 71) | 28 (27, 29) | 70.8 (70.5, 71) | 35 (34, 37) | 77.5 (77.2, 77.7) | 25 (24, 26) | 77.6 (77.3, 77.8) | 32 (31, 32) |
| **Calcasieu** | 71.5 (71.2, 71.8) | 25 (24, 26) | 71.2 (70.9, 71.5) | 33 (32, 35) | 77.6 (77.3, 77.8) | 25 (24, 26) | 78.1 (77.8, 78.4) | 30 (28, 31) |
| **Caldwell** | 69.7 (69, 70.2) | 38 (32, 46) | 70.1 (69.5, 70.6) | 40 (36, 49) | 77 (76.5, 77.5) | 27 (25, 29) | 76.8 (76.2, 77.2) | 35 (33, 37) |
| **Cameron** | 70.8 (70.2, 71.2) | 28 (26, 32) | 72.6 (71.9, 73) | 28 (25, 30) | 77.8 (77.3, 78.2) | 24 (22, 26) | 78 (77.4, 78.5) | 30 (28, 32) |
| **Catahoula** | 69.7 (69, 70.2) | 38 (32, 46) | 70.1 (69.5, 70.6) | 40 (36, 49) | 77 (76.5, 77.5) | 27 (25, 29) | 76.8 (76.2, 77.2) | 35 (33, 37) |
| **Claiborne** | 69.3 (68.6, 69.8) | 43 (37, 48) | 71.1 (70.4, 71.6) | 34 (32, 38) | 77.4 (76.8, 77.9) | 25 (23, 27) | 76.7 (76, 77.2) | 35 (33, 38) |
| **Concordia** | 69.3 (68.7, 69.7) | 44 (39, 48) | 69.9 (69.3, 70.3) | 42 (38, 51) | 75.8 (75.3, 76.2) | 32 (30, 35) | 76.7 (76.1, 77.2) | 35 (33, 38) |
| **De Soto** | 69.6 (69, 70) | 40 (34, 46) | 70.5 (69.9, 70.9) | 37 (35, 42) | 76.6 (76.1, 77) | 28 (27, 31) | 77.3 (76.7, 77.7) | 33 (31, 35) |
| **E. Baton Rouge** | 72.2 (72, 72.4) | 22 (21, 23) | 72.3 (72, 72.4) | 29 (28, 30) | 77.9 (77.7, 78.1) | 23 (23, 24) | 78 (77.8, 78.2) | 30 (29, 31) |
| **East Carroll** | 68.3 (67.6, 68.8) | 51 (47, 51) | 70.1 (69.4, 70.5) | 40 (37, 50) | 76.1 (75.5, 76.5) | 31 (29, 34) | 76.2 (75.5, 76.7) | 37 (35, 41) |
| **East Feliciana** | 69.6 (69.1, 70) | 40 (34, 46) | 69.9 (69.3, 70.3) | 43 (38, 51) | 76.3 (75.8, 76.7) | 30 (28, 32) | 76.7 (76.2, 77.2) | 35 (33, 37) |
| **Evangeline** | 69.5 (69, 69.9) | 41 (35, 46) | 69.8 (69.2, 70.2) | 44 (39, 51) | 76 (75.5, 76.4) | 31 (29, 34) | 75.5 (74.9, 75.9) | 41 (39, 45) |
| **Franklin** | 69.1 (68.4, 69.5) | 45 (42, 49) | 70 (69.3, 70.4) | 41 (37, 51) | 76.1 (75.6, 76.5) | 31 (29, 34) | 76.6 (75.9, 77) | 36 (34, 39) |
| **Grant** | 71.6 (70.9, 72.1) | 24 (23, 28) | 71.3 (70.5, 71.8) | 33 (31, 37) | 77.9 (77.3, 78.4) | 23 (21, 26) | 78.1 (77.4, 78.7) | 30 (28, 32) |
| **Iberia** | 71.9 (71.4, 72.2) | 23 (22, 25) | 72 (71.5, 72.3) | 30 (29, 32) | 77.1 (76.7, 77.4) | 26 (25, 28) | 78 (77.6, 78.4) | 30 (28, 31) |
| **Iberville** | 69 (68.4, 69.4) | 46 (43, 49) | 69.4 (68.8, 69.8) | 49 (43, 51) | 76.3 (75.8, 76.6) | 30 (28, 32) | 76.3 (75.8, 76.7) | 37 (35, 40) |
| **Jackson** | 71.6 (70.9, 72) | 25 (23, 28) | 71.5 (70.8, 72) | 32 (30, 35) | 78.1 (77.5, 78.6) | 23 (21, 25) | 77.1 (76.5, 77.6) | 33 (31, 36) |
| **Jefferson** | 72.2 (72, 72.3) | 22 (22, 23) | 72.4 (72.2, 72.6) | 28 (27, 29) | 78.2 (78, 78.4) | 22 (22, 23) | 79.5 (79.3, 79.7) | 24 (23, 25) |
| **Jefferson Davis** | 70.8 (70.2, 71.2) | 28 (26, 32) | 72.6 (71.9, 73) | 28 (25, 30) | 77.8 (77.3, 78.2) | 24 (22, 26) | 78 (77.4, 78.5) | 30 (28, 32) |
| **La Salle** | 70.9 (70.2, 71.4) | 28 (25, 32) | 71.8 (71.1, 72.4) | 31 (28, 34) | 78 (77.4, 78.6) | 23 (21, 25) | 77.4 (76.7, 78) | 32 (30, 35) |
| **Lafayette** | 72.9 (72.5, 73.1) | 19 (18, 21) | 73.3 (72.9, 73.5) | 24 (23, 26) | 78.8 (78.5, 79.1) | 20 (19, 21) | 78.7 (78.4, 79) | 27 (26, 29) |
| **Lafourche** | 72.6 (72.1, 73) | 20 (19, 22) | 73.1 (72.6, 73.4) | 25 (23, 27) | 78.7 (78.2, 79.1) | 21 (19, 22) | 79.3 (78.8, 79.8) | 25 (22, 27) |
| **Lincoln** | 72.4 (71.9, 72.8) | 21 (19, 23) | 74 (73.4, 74.5) | 20 (18, 23) | 78.2 (77.8, 78.6) | 22 (21, 24) | 78.9 (78.3, 79.3) | 27 (25, 29) |
| **Livingston** | 72.3 (71.8, 72.7) | 22 (20, 24) | 72.3 (71.9, 72.8) | 28 (27, 30) | 78.3 (77.8, 78.8) | 22 (20, 24) | 78.6 (78.1, 79.1) | 28 (26, 30) |
| **Madison** | 68.1 (67.6, 68.5) | 51 (49, 51) | 69.3 (68.6, 69.7) | 51 (46, 51) | 76.1 (75.6, 76.5) | 31 (29, 34) | 75.3 (74.7, 75.7) | 43 (40, 47) |
| **Morehouse** | 68.7 (68.1, 69.1) | 48 (45, 51) | 69.5 (68.9, 69.9) | 49 (42, 51) | 76.2 (75.7, 76.6) | 30 (29, 33) | 75.6 (75.1, 76.1) | 40 (38, 44) |
| **Natchitoches** | 70.3 (69.7, 70.6) | 31 (29, 37) | 70.9 (70.4, 71.3) | 35 (33, 38) | 77 (76.6, 77.4) | 27 (25, 29) | 77.2 (76.8, 77.6) | 33 (31, 35) |
| **Orleans** | 68.1 (67.9, 68.2) | 51 (51, 51) | 68.4 (68.1, 68.6) | 51 (51, 51) | 76.2 (76, 76.3) | 30 (30, 31) | 79.5 (79.3, 79.7) | 24 (23, 25) |
| **Ouachita** | 71.1 (70.8, 71.4) | 27 (25, 28) | 72.1 (71.8, 72.4) | 30 (28, 31) | 78 (77.7, 78.3) | 23 (22, 24) | 77.8 (77.5, 78.1) | 31 (30, 32) |
| **Plaquemines** | 71.1 (70.5, 71.5) | 27 (25, 30) | 72.8 (72.1, 73.2) | 26 (24, 30) | 77.5 (77, 78) | 25 (23, 27) | 79.1 (78.5, 79.6) | 26 (23, 28) |
| **Pointe Coupee** | 70.2 (69.5, 70.6) | 32 (29, 42) | 71.7 (71.1, 72.2) | 31 (29, 34) | 77.7 (77.1, 78.1) | 24 (23, 26) | 78.1 (77.5, 78.7) | 30 (27, 32) |
| **Rapides** | 71.2 (70.9, 71.5) | 26 (25, 28) | 72 (71.7, 72.3) | 30 (29, 31) | 77.1 (76.8, 77.4) | 26 (25, 28) | 77.5 (77.2, 77.8) | 32 (31, 33) |
| **Red River** | 69.3 (68.7, 69.7) | 44 (38, 48) | 70 (69.4, 70.5) | 41 (37, 50) | 76.9 (76.3, 77.3) | 27 (26, 30) | 76.1 (75.5, 76.5) | 38 (36, 41) |
| **Richland** | 68.1 (67.6, 68.5) | 51 (49, 51) | 69.3 (68.6, 69.7) | 51 (46, 51) | 76.1 (75.6, 76.5) | 31 (29, 34) | 75.3 (74.7, 75.7) | 43 (40, 47) |
| **Sabine** | 71.6 (70.9, 72) | 25 (23, 28) | 72.8 (72.1, 73.2) | 26 (24, 29) | 78.1 (77.5, 78.6) | 23 (21, 25) | 78.9 (78.2, 79.4) | 27 (24, 29) |
| **St. Bernard** | 71.7 (71.2, 72) | 24 (23, 26) | 70.6 (70.1, 71.1) | 36 (34, 40) | 77.5 (77.1, 77.9) | 25 (23, 26) | 76.1 (75.6, 76.5) | 38 (36, 40) |
| **St. Charles** | 72.7 (72.2, 73.1) | 20 (18, 22) | 73.6 (73.1, 74.1) | 22 (20, 25) | 78.1 (77.6, 78.6) | 23 (21, 25) | 79.3 (78.7, 79.8) | 25 (22, 28) |
| **St. Helena** | 69.6 (69.1, 70) | 40 (34, 46) | 69.9 (69.3, 70.3) | 43 (38, 51) | 76.3 (75.8, 76.7) | 30 (28, 32) | 76.7 (76.2, 77.2) | 35 (33, 37) |
| **St. James** | 70.4 (69.7, 70.8) | 31 (28, 38) | 71.3 (70.5, 71.7) | 33 (31, 37) | 76.3 (75.7, 76.7) | 30 (28, 33) | 78.3 (77.5, 78.8) | 29 (27, 32) |
| **St. John Baptist** | 71.2 (70.6, 71.5) | 26 (25, 29) | 71.3 (70.8, 71.7) | 33 (31, 35) | 77.1 (76.6, 77.5) | 26 (25, 29) | 76.8 (76.3, 77.2) | 35 (33, 36) |
| **St. Landry** | 70 (69.5, 70.3) | 34 (31, 41) | 70.7 (70.2, 71) | 36 (34, 39) | 76.7 (76.3, 77) | 28 (27, 30) | 77.2 (76.8, 77.5) | 33 (32, 35) |
| **St. Martin** | 70.6 (70.1, 71) | 29 (27, 33) | 71.6 (71.1, 72) | 31 (30, 34) | 77 (76.5, 77.4) | 27 (25, 29) | 77.5 (76.9, 77.9) | 32 (30, 34) |
| **St. Mary** | 70.5 (70.1, 70.9) | 30 (28, 33) | 70.3 (69.8, 70.7) | 38 (36, 44) | 77.2 (76.7, 77.5) | 26 (25, 28) | 78 (77.4, 78.4) | 30 (29, 32) |
| **St. Tammany** | 73.8 (73.4, 74.1) | 15 (13, 16) | 74.1 (73.7, 74.3) | 20 (19, 22) | 79.3 (78.9, 79.6) | 18 (16, 20) | 79.6 (79.3, 79.9) | 23 (22, 25) |
| **Tangipahoa** | 70.1 (69.7, 70.4) | 33 (30, 37) | 70.2 (69.8, 70.6) | 39 (36, 43) | 76.7 (76.3, 77) | 28 (27, 29) | 76 (75.7, 76.3) | 38 (37, 40) |
| **Tensas** | 69.3 (68.7, 69.7) | 44 (39, 48) | 69.9 (69.3, 70.3) | 42 (38, 51) | 75.8 (75.3, 76.2) | 32 (30, 35) | 76.7 (76.1, 77.2) | 35 (33, 38) |
| **Terrebonne** | 71.3 (70.9, 71.6) | 26 (24, 28) | 71.8 (71.3, 72.2) | 31 (29, 33) | 77.4 (77, 77.8) | 25 (24, 27) | 78.6 (78.1, 79.1) | 28 (26, 30) |
| **Union** | 70.4 (69.8, 70.8) | 30 (28, 36) | 71.7 (71, 72.1) | 31 (29, 34) | 77.8 (77.3, 78.3) | 24 (22, 26) | 77.6 (77, 78.1) | 32 (30, 34) |
| **Vermilion** | 71.4 (70.9, 71.9) | 25 (23, 28) | 71.9 (71.3, 72.3) | 30 (29, 33) | 79 (78.5, 79.5) | 19 (17, 21) | 78.5 (77.9, 78.9) | 28 (26, 30) |
| **Vernon** | 72.4 (71.9, 72.8) | 21 (19, 23) | 73.8 (73.2, 74.2) | 22 (20, 24) | 77.9 (77.3, 78.3) | 24 (22, 26) | 79.3 (78.7, 79.9) | 25 (22, 27) |
| **Washington** | 68.8 (68.2, 69.1) | 47 (45, 51) | 68.8 (68.3, 69.1) | 51 (51, 51) | 75.8 (75.4, 76.2) | 32 (30, 35) | 75.7 (75.2, 76) | 40 (38, 43) |
| **Webster** | 70.6 (70.1, 70.9) | 29 (27, 33) | 70.7 (70.2, 71.1) | 36 (34, 39) | 77.1 (76.7, 77.4) | 26 (25, 28) | 77.1 (76.6, 77.4) | 34 (32, 36) |
| **W. Baton Roug.** | 70.7 (70.2, 71.1) | 29 (27, 32) | 71.7 (71.1, 72.1) | 31 (30, 34) | 77 (76.4, 77.3) | 27 (25, 29) | 77.2 (76.6, 77.6) | 33 (31, 35) |
| **West Carroll** | 68.3 (67.6, 68.8) | 51 (47, 51) | 70.1 (69.4, 70.5) | 40 (37, 50) | 76.1 (75.5, 76.5) | 31 (29, 34) | 76.2 (75.5, 76.7) | 37 (35, 41) |
| **West Feliciana** | 70.7 (70.2, 71.1) | 29 (27, 32) | 71.7 (71.1, 72.1) | 31 (30, 34) | 77 (76.4, 77.3) | 27 (25, 29) | 77.2 (76.6, 77.6) | 33 (31, 35) |
| **Winn** | 69.8 (69.1, 70.2) | 37 (32, 45) | 69.7 (69, 70.1) | 45 (40, 51) | 77 (76.4, 77.5) | 27 (25, 29) | 76.8 (76.1, 77.3) | 35 (32, 38) |
| **MAINE** |  |  |  |  |  |  |  |  |
| **Androscoggin** | 74.4 (74, 74.8) | 12 (10, 14) | 75.2 (74.7, 75.5) | 15 (13, 17) | 79.8 (79.4, 80.2) | 15 (13, 17) | 79.9 (79.4, 80.3) | 22 (19, 24) |
| **Aroostook** | 74.1 (73.6, 74.5) | 13 (11, 15) | 74.6 (74.1, 75) | 18 (16, 20) | 79.5 (79.1, 79.9) | 17 (15, 19) | 80.1 (79.6, 80.6) | 20 (17, 23) |
| **Cumberland** | 75.7 (75.4, 76) | 6 (4, 7) | 77.1 (76.7, 77.3) | 7 (7, 9) | 80.8 (80.5, 81.1) | 9 (8, 11) | 81.7 (81.3, 82) | 12 (10, 14) |
| **Franklin** | 75 (74.3, 75.5) | 9 (6, 12) | 76.1 (75.5, 76.7) | 11 (9, 14) | 80.3 (79.7, 80.9) | 12 (9, 16) | 80.3 (79.5, 80.9) | 20 (16, 24) |
| **Hancock** | 75.7 (75.1, 76.1) | 6 (4, 8) | 76 (75.5, 76.5) | 11 (10, 14) | 80.5 (80, 81) | 11 (8, 14) | 81.3 (80.7, 81.9) | 14 (11, 17) |
| **Kennebec** | 74.5 (74, 74.8) | 11 (10, 13) | 75.7 (75.2, 76.1) | 13 (11, 15) | 80.2 (79.7, 80.6) | 13 (11, 15) | 80.4 (79.9, 80.7) | 19 (17, 22) |
| **Knox** | 75.6 (75, 76.1) | 6 (4, 9) | 76.7 (76.1, 77.2) | 9 (7, 11) | 81 (80.4, 81.6) | 8 (5, 11) | 81.4 (80.8, 82) | 13 (10, 16) |
| **Lincoln** | 75.8 (75.2, 76.3) | 5 (3, 8) | 77.3 (76.6, 77.9) | 7 (5, 9) | 80.9 (80.2, 81.4) | 9 (6, 13) | 81 (80.3, 81.5) | 15 (13, 19) |
| **Oxford** | 74.9 (74.3, 75.3) | 10 (7, 12) | 74.5 (73.9, 74.9) | 18 (16, 21) | 79.5 (79, 80) | 17 (14, 19) | 80 (79.4, 80.5) | 21 (18, 24) |
| **Penobscot** | 74 (73.7, 74.3) | 13 (12, 15) | 75 (74.6, 75.3) | 16 (14, 18) | 79.5 (79.1, 79.8) | 17 (15, 19) | 80.1 (79.7, 80.4) | 21 (19, 23) |
| **Piscataquis** | 73.9 (73.2, 74.4) | 14 (12, 17) | 74.3 (73.5, 75) | 19 (16, 23) | 79.6 (79, 80.3) | 16 (13, 19) | 80.5 (79.6, 81.2) | 18 (14, 23) |
| **Sagadahoc** | 75.9 (75.2, 76.4) | 5 (3, 8) | 76.5 (75.9, 76.9) | 9 (8, 12) | 80.5 (79.9, 81.1) | 11 (8, 15) | 80.5 (79.9, 81.1) | 18 (15, 22) |
| **Somerset** | 74 (73.4, 74.5) | 13 (11, 16) | 74.8 (74.2, 75.3) | 17 (14, 20) | 79.6 (79.1, 80.1) | 16 (13, 19) | 79.8 (79.2, 80.3) | 22 (19, 25) |
| **Waldo** | 74.7 (74, 75.2) | 10 (8, 14) | 75.4 (74.7, 75.9) | 14 (12, 17) | 80 (79.4, 80.6) | 14 (11, 17) | 80.8 (80.1, 81.4) | 16 (13, 21) |
| **Washington** | 73.7 (73.1, 74.1) | 15 (13, 18) | 73.1 (72.5, 73.6) | 25 (22, 28) | 79.2 (78.6, 79.7) | 18 (16, 21) | 80.1 (79.4, 80.6) | 21 (17, 24) |
| **York** | 75.8 (75.4, 76.1) | 5 (4, 7) | 77 (76.6, 77.3) | 8 (6, 9) | 80.6 (80.3, 81) | 10 (8, 13) | 81.5 (81.1, 81.9) | 13 (11, 15) |
| **MARYLAND** |  |  |  |  |  |  |  |  |
| **Allegany** | 73.4 (72.9, 73.8) | 16 (14, 19) | 74 (73.5, 74.4) | 21 (19, 23) | 79.2 (78.7, 79.6) | 18 (16, 20) | 79.7 (79.2, 80.2) | 23 (20, 25) |
| **Anne Arundel** | 74.9 (74.7, 75.1) | 9 (9, 11) | 75.7 (75.5, 75.9) | 12 (12, 13) | 79.1 (78.9, 79.3) | 19 (18, 20) | 80.2 (80, 80.4) | 20 (19, 21) |
| **Baltimore City** | 64.2 (64.1, 64.4) | 51 (51, 51) | 66.7 (66.6, 66.9) | 51 (51, 51) | 73.6 (73.5, 73.8) | 45 (44, 46) | 75.6 (75.4, 75.8) | 41 (40, 42) |
| **Baltimore Cnty** | 74.4 (74.3, 74.6) | 12 (11, 12) | 75.1 (74.9, 75.2) | 15 (15, 16) | 79.5 (79.4, 79.7) | 17 (16, 17) | 80.3 (80.1, 80.4) | 19 (18, 20) |
| **Calvert** | 74.8 (74.4, 75.2) | 10 (8, 12) | 75 (74.6, 75.3) | 16 (14, 18) | 80.3 (79.8, 80.8) | 12 (10, 15) | 79.7 (79.3, 80.1) | 22 (20, 25) |
| **Caroline** | 72.1 (71.5, 72.5) | 23 (21, 25) | 73.2 (72.6, 73.6) | 24 (22, 28) | 78.2 (77.6, 78.6) | 22 (21, 24) | 78.7 (78.1, 79.2) | 27 (25, 30) |
| **Carroll** | 75.7 (75.3, 76) | 6 (4, 8) | 76.6 (76.2, 76.9) | 9 (8, 10) | 79.9 (79.6, 80.2) | 15 (13, 16) | 80.5 (80.1, 80.8) | 18 (16, 21) |
| **Cecil** | 73.3 (72.8, 73.7) | 17 (15, 19) | 73.8 (73.3, 74.2) | 21 (19, 24) | 78.8 (78.4, 79.3) | 20 (18, 22) | 79.4 (78.9, 79.9) | 24 (22, 27) |
| **Charles** | 74.2 (73.8, 74.5) | 13 (11, 15) | 74.7 (74.3, 75) | 17 (16, 19) | 78.8 (78.4, 79.1) | 20 (19, 22) | 79.2 (78.8, 79.5) | 25 (24, 27) |
| **Dorchester** | 71.5 (70.9, 71.9) | 25 (23, 27) | 72.5 (71.9, 72.9) | 28 (26, 30) | 77.7 (77.2, 78.1) | 24 (23, 26) | 78.7 (78.2, 79.2) | 27 (25, 29) |
| **Frederick** | 76 (75.6, 76.3) | 5 (3, 6) | 76.8 (76.5, 77.1) | 8 (7, 9) | 80.9 (80.6, 81.3) | 9 (7, 11) | 81.2 (80.9, 81.5) | 14 (13, 16) |
| **Garrett** | 74.4 (73.7, 74.9) | 12 (9, 15) | 75 (74.3, 75.6) | 16 (13, 19) | 79.3 (78.7, 79.9) | 18 (15, 20) | 80.2 (79.5, 80.8) | 20 (16, 24) |
| **Harford** | 74.9 (74.6, 75.2) | 9 (8, 11) | 75.6 (75.3, 75.8) | 13 (12, 15) | 79.7 (79.4, 80) | 15 (14, 17) | 80.6 (80.3, 80.8) | 18 (16, 20) |
| **Howard** | 77.4 (77.1, 77.7) | -1 (-2, 0) | 79.8 (79.4, 80.1) | -6 (-8, -3) | 81 (80.7, 81.3) | 8 (7, 10) | 82.6 (82.3, 82.9) | 8 (6, 9) |
| **Kent** | 74 (73.4, 74.4) | 13 (12, 16) | 74.5 (73.9, 75) | 18 (16, 21) | 79.6 (79, 80.1) | 16 (13, 19) | 80.6 (79.9, 81.1) | 18 (15, 21) |
| **Montgomery** | 79.1 (78.9, 79.2) | -8 (-9, -7) | 80.7 (80.5, 80.8) | -12 (-13, -11) | 82.5 (82.3, 82.7) | 1 (0, 2) | 84.5 (84.3, 84.7) | -3 (-5, -2) |
| **Prince George's** | 72.2 (72, 72.3) | 22 (22, 23) | 73.5 (73.3, 73.6) | 23 (22, 24) | 77.7 (77.6, 77.9) | 24 (23, 25) | 79.2 (79, 79.4) | 25 (25, 26) |
| **Queen Anne's** | 75.2 (74.6, 75.6) | 8 (6, 11) | 76.1 (75.6, 76.6) | 11 (9, 13) | 80.1 (79.6, 80.6) | 13 (10, 16) | 81.8 (81.1, 82.4) | 11 (9, 15) |
| **Somerset** | 70.7 (70.2, 71.1) | 28 (27, 32) | 72 (71.5, 72.4) | 30 (28, 32) | 77.4 (76.9, 77.8) | 25 (24, 27) | 77.4 (76.8, 77.9) | 32 (31, 34) |
| **St. Mary’s** | 74.4 (73.9, 74.7) | 12 (10, 14) | 75.5 (75, 75.8) | 14 (12, 16) | 79.6 (79.1, 80) | 16 (14, 19) | 80.3 (79.8, 80.8) | 19 (16, 22) |
| **Talbot** | 75.6 (75.1, 76) | 6 (4, 8) | 76.4 (75.8, 76.8) | 10 (8, 12) | 80.3 (79.8, 80.7) | 12 (10, 15) | 81.7 (81.2, 82.2) | 12 (9, 14) |
| **Washington** | 74.8 (74.4, 75.1) | 10 (8, 12) | 74.6 (74.2, 74.9) | 18 (16, 20) | 79.4 (78.9, 79.7) | 18 (16, 19) | 79.7 (79.2, 80) | 23 (21, 25) |
| **Wicomico** | 72.6 (72.2, 72.9) | 20 (19, 22) | 73 (72.6, 73.3) | 25 (24, 27) | 78.1 (77.7, 78.5) | 23 (21, 24) | 78.9 (78.5, 79.3) | 27 (25, 28) |
| **Worcester** | 73.6 (73.1, 74) | 15 (13, 18) | 75.3 (74.7, 75.7) | 14 (12, 17) | 79.4 (79, 79.8) | 17 (15, 19) | 80.7 (80.2, 81.2) | 17 (14, 20) |
| **MASSACHUSETTS** |  |  |  |  |  |  |  |  |
| **Barnstable** | 77.2 (76.8, 77.4) | 0 (-1, 1) | 78.1 (77.7, 78.3) | 4 (3, 5) | 82 (81.7, 82.3) | 3 (2, 5) | 82.9 (82.5, 83.2) | 7 (5, 8) |
| **Berkshire** | 75.3 (75, 75.6) | 7 (6, 9) | 76.9 (76.5, 77.2) | 8 (7, 10) | 80.9 (80.6, 81.2) | 9 (7, 11) | 81.1 (80.7, 81.5) | 15 (13, 17) |
| **Bristol** | 74.8 (74.6, 75) | 10 (9, 11) | 75.8 (75.6, 76) | 12 (11, 13) | 80.7 (80.5, 80.9) | 10 (9, 11) | 81.4 (81.2, 81.6) | 13 (12, 14) |
| **Dukes** | 76.5 (75.8, 77.1) | 2 (0, 5) | 78.2 (77.4, 78.9) | 3 (0, 6) | 81.3 (80.5, 81.9) | 7 (4, 11) | 82.9 (82.1, 83.7) | 6 (2, 10) |
| **Essex** | 75.9 (75.7, 76.1) | 5 (4, 6) | 77.4 (77.2, 77.6) | 6 (6, 7) | 80.9 (80.7, 81.1) | 9 (8, 10) | 81.7 (81.5, 81.9) | 12 (11, 13) |
| **Franklin** | 75.8 (75.3, 76.1) | 5 (4, 8) | 77.1 (76.6, 77.5) | 7 (6, 9) | 81.3 (80.8, 81.7) | 7 (5, 10) | 81.3 (80.8, 81.8) | 14 (11, 16) |
| **Hampden** | 73.8 (73.6, 74) | 14 (13, 15) | 75.2 (75, 75.4) | 15 (14, 16) | 79.5 (79.3, 79.7) | 17 (16, 18) | 80.5 (80.3, 80.7) | 18 (17, 19) |
| **Hampshire** | 76.2 (75.8, 76.5) | 4 (2, 5) | 77.5 (77.1, 77.8) | 6 (5, 7) | 81.1 (80.7, 81.4) | 8 (6, 10) | 81.6 (81.1, 82) | 12 (10, 15) |
| **Middlesex** | 77 (76.8, 77.1) | 1 (0, 1) | 78.8 (78.7, 79) | 0 (-1, 1) | 81.5 (81.4, 81.6) | 6 (5, 6) | 82.9 (82.7, 83) | 7 (6, 7) |
| **Nantucket** | 77.2 (76.8, 77.4) | 0 (-1, 1) | 78.1 (77.7, 78.3) | 4 (3, 5) | 82 (81.7, 82.3) | 3 (2, 5) | 82.9 (82.5, 83.2) | 7 (5, 8) |
| **Norfolk** | 77.4 (77.2, 77.6) | -1 (-1, 0) | 78.2 (78, 78.4) | 3 (2, 4) | 81.6 (81.4, 81.8) | 5 (4, 6) | 82.6 (82.4, 82.8) | 8 (7, 9) |
| **Plymouth** | 75.1 (74.9, 75.3) | 8 (7, 9) | 76.3 (76.1, 76.5) | 10 (9, 11) | 80.2 (80, 80.4) | 13 (11, 14) | 81.3 (81, 81.5) | 14 (13, 15) |
| **Suffolk** | 73.8 (73.6, 73.9) | 15 (14, 15) | 75.8 (75.6, 75.9) | 12 (12, 13) | 79.5 (79.4, 79.7) | 17 (16, 17) | 81.9 (81.7, 82.1) | 11 (10, 12) |
| **Worcester** | 75.3 (75.2, 75.5) | 7 (6, 8) | 76.4 (76.2, 76.6) | 10 (9, 11) | 80.3 (80.1, 80.5) | 12 (11, 13) | 81.2 (81, 81.4) | 14 (13, 15) |
| **MICHIGAN** |  |  |  |  |  |  |  |  |
| **Alcona** | 73.5 (72.8, 74) | 16 (13, 19) | 75.2 (74.3, 75.9) | 15 (12, 19) | 80.3 (79.5, 81) | 12 (8, 17) | 79.6 (78.8, 80.4) | 23 (19, 27) |
| **Alger** | 74.1 (73.5, 74.7) | 13 (11, 16) | 74.7 (74, 75.3) | 17 (14, 20) | 79.3 (78.6, 79.9) | 18 (15, 21) | 80 (79.2, 80.6) | 21 (17, 25) |
| **Allegan** | 74.7 (74.3, 75.1) | 10 (8, 12) | 76 (75.6, 76.4) | 11 (10, 13) | 79.4 (79, 79.8) | 17 (15, 19) | 80.9 (80.4, 81.4) | 16 (13, 19) |
| **Alpena** | 74 (73.4, 74.5) | 13 (11, 16) | 75.4 (74.7, 75.9) | 14 (12, 17) | 80 (79.4, 80.6) | 14 (10, 17) | 81.2 (80.4, 81.9) | 14 (11, 18) |
| **Antrim** | 74.9 (74.2, 75.3) | 10 (7, 13) | 76.2 (75.5, 76.8) | 10 (8, 14) | 80.3 (79.6, 80.9) | 12 (9, 16) | 80.9 (80.1, 81.6) | 16 (12, 20) |
| **Arenac** | 73.7 (72.9, 74.2) | 15 (13, 19) | 74.1 (73.4, 74.7) | 20 (18, 23) | 78.7 (78.1, 79.3) | 20 (18, 23) | 79.6 (78.8, 80.4) | 23 (19, 27) |
| **Baraga** | 73.3 (72.7, 73.8) | 17 (15, 20) | 75.1 (74.3, 75.6) | 16 (13, 19) | 80.2 (79.5, 80.7) | 13 (10, 17) | 80.9 (80.2, 81.5) | 16 (13, 20) |
| **Barry** | 75 (74.4, 75.4) | 9 (7, 12) | 75.4 (74.8, 75.8) | 14 (12, 17) | 80 (79.4, 80.4) | 14 (11, 17) | 80.7 (80.1, 81.3) | 17 (14, 21) |
| **Bay** | 74.2 (73.8, 74.5) | 13 (11, 15) | 74.7 (74.2, 75.1) | 17 (16, 19) | 80.3 (79.8, 80.7) | 12 (10, 15) | 80.4 (80, 80.9) | 19 (16, 21) |
| **Benzie** | 75.4 (74.6, 75.9) | 7 (5, 11) | 75.7 (74.9, 76.4) | 13 (10, 17) | 80.4 (79.6, 81.1) | 12 (8, 16) | 80.9 (80, 81.7) | 16 (12, 21) |
| **Berrien** | 73.3 (73, 73.6) | 17 (15, 18) | 74.8 (74.4, 75.1) | 17 (16, 19) | 78.9 (78.6, 79.2) | 19 (18, 21) | 79.8 (79.4, 80.1) | 22 (21, 24) |
| **Branch** | 73.7 (73, 74.1) | 15 (13, 18) | 74.7 (74, 75.2) | 17 (15, 20) | 79.5 (78.8, 79.9) | 17 (14, 20) | 79.6 (78.9, 80.1) | 23 (20, 27) |
| **Calhoun** | 72.3 (71.9, 72.6) | 22 (20, 23) | 73.1 (72.7, 73.4) | 25 (23, 27) | 78.4 (78.1, 78.7) | 22 (20, 23) | 79.1 (78.7, 79.4) | 26 (24, 28) |
| **Cass** | 73.8 (73.2, 74.2) | 15 (13, 17) | 74.6 (74, 75) | 18 (16, 20) | 79.4 (78.8, 79.8) | 17 (15, 20) | 79.6 (79.1, 80.1) | 23 (20, 26) |
| **Charlevoix** | 75.4 (74.7, 75.9) | 7 (5, 11) | 76.8 (76, 77.4) | 8 (6, 11) | 80.7 (80, 81.4) | 10 (6, 14) | 81.4 (80.5, 82.1) | 13 (10, 18) |
| **Cheboygan** | 74.7 (74, 75.2) | 10 (8, 13) | 75.8 (75, 76.3) | 12 (10, 16) | 79.9 (79.3, 80.5) | 15 (11, 18) | 80.6 (79.8, 81.2) | 18 (14, 22) |
| **Chippewa** | 75.1 (74.6, 75.5) | 8 (6, 11) | 75.9 (75.3, 76.4) | 12 (10, 14) | 80.1 (79.4, 80.5) | 14 (11, 17) | 80.7 (80, 81.3) | 17 (14, 21) |
| **Clare** | 72.1 (71.4, 72.6) | 23 (21, 25) | 72.7 (72, 73.2) | 27 (24, 30) | 78.6 (78, 79.1) | 21 (19, 23) | 79 (78.3, 79.6) | 26 (23, 29) |
| **Clinton** | 76.3 (75.8, 76.7) | 3 (2, 5) | 77 (76.5, 77.4) | 8 (6, 9) | 80.3 (79.8, 80.8) | 12 (9, 15) | 81.5 (80.9, 82.1) | 13 (10, 16) |
| **Crawford** | 73.8 (73, 74.4) | 15 (12, 18) | 74.1 (73.2, 74.7) | 20 (17, 24) | 79.5 (78.7, 80.2) | 17 (13, 20) | 79.2 (78.3, 79.9) | 25 (22, 29) |
| **Delta** | 75.1 (74.5, 75.6) | 8 (6, 11) | 76.5 (75.8, 77) | 9 (8, 12) | 80.6 (80, 81.1) | 11 (8, 14) | 80.9 (80.2, 81.5) | 16 (13, 20) |
| **Dickinson** | 75.3 (74.5, 75.7) | 8 (5, 11) | 76.5 (75.8, 77.1) | 9 (7, 12) | 81 (80.3, 81.6) | 8 (5, 12) | 81.9 (81.1, 82.6) | 11 (8, 15) |
| **Eaton** | 75.7 (75.2, 76.1) | 6 (4, 8) | 76 (75.6, 76.3) | 11 (10, 13) | 80.2 (79.7, 80.6) | 13 (11, 16) | 80.4 (80, 80.8) | 19 (16, 21) |
| **Emmet** | 75.6 (75, 76.1) | 6 (4, 9) | 77.7 (77, 78.2) | 5 (3, 8) | 80.7 (80.1, 81.3) | 10 (7, 13) | 81.6 (80.9, 82.3) | 12 (9, 16) |
| **Genesee** | 72 (71.8, 72.2) | 23 (22, 24) | 73.2 (73, 73.5) | 24 (23, 25) | 77.5 (77.3, 77.7) | 25 (24, 26) | 78.6 (78.3, 78.8) | 28 (27, 29) |
| **Gladwin** | 73.5 (72.8, 74) | 16 (14, 19) | 74.2 (73.5, 74.8) | 20 (17, 23) | 79.8 (79.1, 80.4) | 15 (12, 19) | 79.6 (78.9, 80.2) | 23 (20, 27) |
| **Gogebic** | 74.3 (73.6, 74.7) | 12 (10, 15) | 75 (74.3, 75.5) | 16 (14, 19) | 79.7 (79.1, 80.2) | 16 (13, 19) | 80.7 (80, 81.3) | 17 (14, 21) |
| **Grand Traverse** | 76 (75.5, 76.5) | 4 (3, 6) | 77.7 (77.2, 78.1) | 5 (3, 7) | 80.9 (80.4, 81.3) | 9 (7, 12) | 82 (81.4, 82.4) | 11 (8, 13) |
| **Gratiot** | 74.3 (73.7, 74.8) | 12 (10, 15) | 75.2 (74.6, 75.7) | 15 (13, 18) | 79.7 (79.1, 80.2) | 16 (13, 19) | 79.6 (78.9, 80.1) | 23 (20, 27) |
| **Hillsdale** | 74.1 (73.5, 74.6) | 13 (11, 16) | 75.2 (74.6, 75.7) | 15 (12, 18) | 79.6 (79, 80.2) | 16 (13, 19) | 80.5 (79.8, 81.1) | 18 (15, 22) |
| **Houghton** | 74.5 (73.9, 75) | 11 (9, 14) | 75.2 (74.5, 75.6) | 15 (13, 18) | 79.8 (79.2, 80.3) | 15 (12, 18) | 80.1 (79.4, 80.7) | 20 (17, 24) |
| **Huron** | 74.4 (73.8, 74.9) | 12 (9, 15) | 75.1 (74.4, 75.7) | 15 (13, 19) | 80 (79.4, 80.6) | 14 (11, 17) | 80 (79.3, 80.6) | 21 (17, 25) |
| **Ingham** | 74.7 (74.4, 74.9) | 10 (9, 12) | 76.1 (75.8, 76.4) | 11 (10, 12) | 79.9 (79.6, 80.2) | 15 (13, 16) | 80.4 (80, 80.7) | 19 (17, 21) |
| **Ionia** | 74.7 (74.2, 75.1) | 10 (9, 13) | 75.1 (74.6, 75.5) | 15 (14, 18) | 79.4 (78.9, 79.9) | 17 (15, 20) | 80 (79.4, 80.5) | 21 (18, 24) |
| **Iosco** | 73.7 (73, 74.2) | 15 (13, 18) | 73.5 (72.8, 74) | 23 (21, 26) | 79.9 (79.2, 80.4) | 15 (12, 18) | 79.5 (78.8, 80.2) | 24 (20, 27) |
| **Iron** | 73.3 (72.7, 73.8) | 17 (15, 20) | 75.1 (74.3, 75.6) | 16 (13, 19) | 80.2 (79.5, 80.7) | 13 (10, 17) | 80.9 (80.2, 81.5) | 16 (13, 20) |
| **Isabella** | 74.8 (74.3, 75.2) | 10 (8, 12) | 75.5 (74.9, 75.9) | 14 (12, 16) | 80.1 (79.5, 80.7) | 13 (10, 17) | 80.4 (79.8, 81) | 19 (15, 22) |
| **Jackson** | 73.6 (73.3, 73.9) | 15 (14, 17) | 74.4 (74, 74.7) | 19 (18, 20) | 79.5 (79.2, 79.9) | 17 (15, 18) | 79.3 (79, 79.7) | 25 (23, 26) |
| **Kalamazoo** | 75.1 (74.7, 75.3) | 9 (7, 10) | 75.7 (75.4, 76) | 12 (11, 14) | 79.9 (79.6, 80.2) | 14 (13, 16) | 80.8 (80.4, 81) | 17 (15, 19) |
| **Kalkaska** | 73.9 (73.1, 74.5) | 14 (11, 18) | 74.8 (73.9, 75.5) | 17 (14, 21) | 79.3 (78.5, 80) | 18 (14, 21) | 80 (79.1, 80.8) | 21 (16, 26) |
| **Kent** | 75.3 (75, 75.5) | 8 (7, 9) | 76.5 (76.3, 76.7) | 9 (9, 10) | 80.5 (80.2, 80.7) | 11 (10, 13) | 81 (80.8, 81.2) | 15 (14, 16) |
| **Keweenaw** | 74.5 (73.9, 75) | 11 (9, 14) | 75.2 (74.5, 75.6) | 15 (13, 18) | 79.8 (79.2, 80.3) | 15 (12, 18) | 80.1 (79.4, 80.7) | 20 (17, 24) |
| **Lake** | 72.5 (71.9, 73) | 21 (19, 23) | 74.1 (73.4, 74.6) | 20 (18, 23) | 79.1 (78.5, 79.6) | 19 (16, 21) | 78.9 (78.3, 79.4) | 27 (24, 29) |
| **Lapeer** | 74.9 (74.5, 75.3) | 9 (7, 11) | 76.1 (75.6, 76.5) | 11 (10, 13) | 79.1 (78.6, 79.5) | 19 (17, 21) | 80.1 (79.5, 80.6) | 21 (18, 24) |
| **Leelanau** | 77 (76.4, 77.6) | 0 (-1, 3) | 78.6 (77.8, 79.3) | 1 (-2, 5) | 81.4 (80.7, 82) | 6 (3, 10) | 82.8 (82, 83.6) | 7 (3, 10) |
| **Lenawee** | 74.7 (74.3, 75.1) | 10 (8, 12) | 76.2 (75.7, 76.6) | 11 (9, 13) | 79.7 (79.2, 80.1) | 16 (14, 18) | 79.8 (79.4, 80.3) | 22 (20, 24) |
| **Livingston** | 76.1 (75.7, 76.5) | 4 (2, 6) | 77.2 (76.8, 77.6) | 7 (6, 8) | 80 (79.6, 80.4) | 14 (12, 16) | 81.2 (80.8, 81.6) | 14 (12, 17) |
| **Luce** | 74.3 (73.7, 74.8) | 12 (10, 15) | 75 (74.2, 75.5) | 16 (13, 19) | 79.9 (79.3, 80.5) | 15 (11, 18) | 80.3 (79.5, 81) | 19 (15, 24) |
| **Mackinac** | 74.3 (73.7, 74.8) | 12 (10, 15) | 75 (74.2, 75.5) | 16 (13, 19) | 79.9 (79.3, 80.5) | 15 (11, 18) | 80.3 (79.5, 81) | 19 (15, 24) |
| **Macomb** | 74.7 (74.6, 74.9) | 10 (9, 11) | 75.6 (75.4, 75.7) | 13 (12, 14) | 79.9 (79.7, 80) | 15 (14, 16) | 80.5 (80.3, 80.7) | 18 (17, 19) |
| **Manistee** | 74.1 (73.4, 74.6) | 13 (11, 16) | 75.3 (74.6, 75.9) | 14 (12, 18) | 79.7 (79.1, 80.3) | 16 (12, 19) | 80.8 (80, 81.5) | 16 (13, 21) |
| **Marquette** | 74.9 (74.4, 75.3) | 9 (7, 12) | 76.3 (75.7, 76.7) | 10 (9, 13) | 79.6 (79.1, 80.1) | 16 (14, 19) | 80.4 (79.9, 80.9) | 19 (16, 22) |
| **Mason** | 74.8 (74.1, 75.3) | 10 (7, 13) | 75.5 (74.7, 76) | 14 (11, 17) | 79.6 (78.9, 80.1) | 16 (13, 20) | 80.5 (79.7, 81.1) | 18 (15, 23) |
| **Mecosta** | 74.4 (73.9, 74.9) | 12 (10, 14) | 75.3 (74.8, 75.9) | 14 (12, 17) | 79.3 (78.8, 79.8) | 18 (15, 20) | 80.6 (79.9, 81.2) | 18 (14, 22) |
| **Menominee** | 74.5 (73.9, 75.1) | 11 (9, 14) | 76.2 (75.5, 76.8) | 10 (8, 14) | 80 (79.4, 80.6) | 14 (11, 17) | 81 (80.2, 81.7) | 15 (12, 20) |
| **Midland** | 76.2 (75.7, 76.6) | 4 (2, 6) | 77.1 (76.6, 77.6) | 7 (6, 9) | 81.5 (81, 82) | 5 (3, 8) | 81.2 (80.7, 81.7) | 14 (12, 17) |
| **Missaukee** | 73.5 (72.8, 74) | 16 (13, 20) | 75.1 (74.3, 75.8) | 15 (12, 19) | 80 (79.2, 80.6) | 14 (10, 18) | 79 (78.2, 79.6) | 26 (23, 29) |
| **Monroe** | 74.2 (73.8, 74.5) | 12 (11, 14) | 74.6 (74.2, 74.9) | 18 (16, 19) | 79.3 (79, 79.7) | 18 (16, 19) | 79.7 (79.3, 80) | 23 (21, 25) |
| **Montcalm** | 73 (72.5, 73.5) | 18 (16, 21) | 74.4 (73.9, 74.9) | 19 (17, 21) | 78.9 (78.3, 79.3) | 20 (18, 22) | 79 (78.5, 79.5) | 26 (24, 28) |
| **Montmorency** | 73.2 (72.5, 73.7) | 17 (15, 21) | 74.7 (74, 75.3) | 17 (14, 21) | 79.8 (79.1, 80.3) | 15 (12, 19) | 80.4 (79.6, 81) | 19 (15, 23) |
| **Muskegon** | 73.2 (72.8, 73.4) | 18 (16, 19) | 74.3 (74, 74.6) | 19 (18, 21) | 79 (78.6, 79.3) | 19 (18, 21) | 79.3 (78.9, 79.6) | 25 (23, 27) |
| **Newaygo** | 73.7 (73.2, 74.2) | 15 (12, 17) | 75 (74.4, 75.5) | 16 (13, 19) | 79.4 (78.8, 79.9) | 17 (15, 20) | 79.8 (79.2, 80.4) | 22 (19, 25) |
| **Oakland** | 76 (75.9, 76.2) | 4 (4, 5) | 77.3 (77.1, 77.4) | 7 (6, 7) | 80.3 (80.1, 80.4) | 12 (12, 13) | 81.5 (81.3, 81.6) | 13 (12, 14) |
| **Oceana** | 73.4 (72.7, 74) | 16 (14, 20) | 75.4 (74.7, 76) | 14 (11, 18) | 80.1 (79.4, 80.8) | 13 (10, 17) | 80.9 (80, 81.6) | 16 (12, 21) |
| **Ogemaw** | 72.3 (71.6, 72.8) | 22 (19, 25) | 73.1 (72.4, 73.6) | 25 (22, 28) | 79.3 (78.6, 79.8) | 18 (15, 21) | 78.6 (77.9, 79.2) | 28 (25, 30) |
| **Ontonagon** | 74.3 (73.6, 74.7) | 12 (10, 15) | 75 (74.3, 75.5) | 16 (14, 19) | 79.7 (79.1, 80.2) | 16 (13, 19) | 80.7 (80, 81.3) | 17 (14, 21) |
| **Osceola** | 72.5 (71.9, 73) | 21 (19, 23) | 74.1 (73.4, 74.6) | 20 (18, 23) | 79.1 (78.5, 79.6) | 19 (16, 21) | 78.9 (78.3, 79.4) | 27 (24, 29) |
| **Oscoda** | 73.5 (72.8, 74) | 16 (13, 19) | 75.2 (74.3, 75.9) | 15 (12, 19) | 80.3 (79.5, 81) | 12 (8, 17) | 79.6 (78.8, 80.4) | 23 (19, 27) |
| **Otsego** | 75.5 (74.7, 76.2) | 6 (4, 10) | 76 (75.1, 76.6) | 12 (9, 15) | 79.9 (79.2, 80.6) | 14 (11, 18) | 80.4 (79.6, 81.1) | 19 (15, 23) |
| **Ottawa** | 76.6 (76.3, 76.9) | 2 (1, 3) | 78.8 (78.4, 79.1) | 0 (-2, 2) | 81.7 (81.3, 82) | 5 (3, 7) | 82.4 (82, 82.8) | 8 (7, 10) |
| **Presque Isle** | 73.2 (72.5, 73.7) | 17 (15, 21) | 74.7 (74, 75.3) | 17 (14, 21) | 79.8 (79.1, 80.3) | 15 (12, 19) | 80.4 (79.6, 81) | 19 (15, 23) |
| **Roscommon** | 73.4 (72.7, 73.9) | 16 (14, 20) | 73.8 (73.1, 74.3) | 21 (19, 25) | 79.3 (78.7, 79.8) | 18 (15, 20) | 79.9 (79.1, 80.5) | 22 (18, 26) |
| **Saginaw** | 73.5 (73.2, 73.7) | 16 (15, 17) | 73.5 (73.2, 73.8) | 23 (22, 24) | 79.4 (79, 79.6) | 18 (16, 19) | 79.1 (78.8, 79.4) | 26 (24, 27) |
| **Sanilac** | 73.5 (72.9, 73.9) | 16 (14, 19) | 75.2 (74.6, 75.7) | 15 (12, 18) | 79.2 (78.7, 79.7) | 18 (16, 21) | 79.5 (78.9, 80.1) | 24 (21, 27) |
| **Schoolcraft** | 74.1 (73.5, 74.7) | 13 (11, 16) | 74.7 (74, 75.3) | 17 (14, 20) | 79.3 (78.6, 79.9) | 18 (15, 21) | 80 (79.2, 80.6) | 21 (17, 25) |
| **Shiawassee** | 74.2 (73.7, 74.6) | 12 (11, 15) | 75.2 (74.6, 75.6) | 15 (13, 18) | 79.8 (79.2, 80.2) | 15 (13, 18) | 80.5 (79.8, 81) | 18 (15, 22) |
| **St. Clair** | 73.9 (73.5, 74.2) | 14 (13, 16) | 75.5 (75.1, 75.8) | 14 (12, 15) | 79.3 (78.9, 79.7) | 18 (16, 19) | 79.8 (79.4, 80.2) | 22 (20, 24) |
| **St. Joseph** | 73.2 (72.7, 73.6) | 17 (15, 20) | 73.5 (73, 74) | 23 (21, 26) | 79 (78.5, 79.5) | 19 (17, 21) | 79.8 (79.1, 80.3) | 22 (19, 26) |
| **Tuscola** | 73.9 (73.4, 74.3) | 14 (12, 16) | 74.7 (74.1, 75.1) | 17 (15, 20) | 79.4 (78.8, 79.8) | 17 (15, 20) | 79.7 (79.1, 80.1) | 23 (20, 26) |
| **Van Buren** | 73.7 (73.2, 74.1) | 15 (13, 17) | 73.9 (73.4, 74.3) | 21 (19, 23) | 79.3 (78.8, 79.7) | 18 (16, 20) | 79.6 (79, 80) | 23 (21, 26) |
| **Washtenaw** | 76.5 (76.2, 76.7) | 3 (2, 4) | 77.9 (77.7, 78.2) | 4 (3, 5) | 80.6 (80.3, 80.9) | 10 (9, 12) | 81.7 (81.4, 82) | 12 (11, 13) |
| **Wayne** | 70.3 (70.2, 70.4) | 31 (30, 32) | 71.9 (71.8, 71.9) | 30 (30, 31) | 76.7 (76.6, 76.8) | 28 (28, 28) | 78 (77.9, 78) | 30 (30, 31) |
| **Wexford** | 73.8 (73.1, 74.3) | 15 (12, 18) | 74.9 (74.2, 75.5) | 16 (13, 20) | 79.7 (79, 80.3) | 16 (12, 19) | 79.9 (79.2, 80.5) | 22 (18, 25) |
| **MINNESOTA** |  |  |  |  |  |  |  |  |
| **Aitkin** | 74.7 (73.9, 75.3) | 10 (8, 14) | 76.2 (75.4, 76.9) | 10 (8, 14) | 80.3 (79.6, 80.9) | 12 (9, 16) | 81.6 (80.7, 82.3) | 12 (9, 17) |
| **Anoka** | 76.3 (76, 76.6) | 3 (2, 4) | 78.3 (77.9, 78.6) | 3 (1, 4) | 81.1 (80.8, 81.5) | 8 (6, 9) | 82.4 (82, 82.8) | 9 (7, 10) |
| **Becker** | 74.4 (73.8, 74.8) | 12 (10, 14) | 75.7 (75, 76.2) | 13 (11, 16) | 80.9 (80.2, 81.4) | 9 (6, 13) | 81.2 (80.5, 81.8) | 14 (11, 18) |
| **Beltrami** | 74.2 (73.5, 74.6) | 13 (11, 16) | 74.6 (73.9, 75.1) | 18 (15, 21) | 80.5 (79.9, 81) | 11 (8, 15) | 81 (80.3, 81.6) | 15 (12, 19) |
| **Benton** | 74.9 (74.3, 75.4) | 9 (7, 12) | 75.4 (74.9, 76) | 14 (12, 17) | 80.4 (79.8, 81) | 12 (9, 15) | 80.2 (79.6, 80.8) | 20 (16, 23) |
| **Big Stone** | 74.7 (74, 75.2) | 10 (8, 14) | 76.5 (75.7, 77.2) | 9 (7, 13) | 81 (80.3, 81.6) | 8 (5, 12) | 82.2 (81.3, 82.9) | 9 (6, 14) |
| **Blue Earth** | 75.9 (75.3, 76.3) | 5 (3, 7) | 77.4 (76.8, 77.9) | 6 (4, 8) | 81.2 (80.6, 81.7) | 7 (5, 11) | 81.6 (80.9, 82.2) | 12 (9, 16) |
| **Brown** | 76 (75.3, 76.5) | 4 (2, 7) | 77.4 (76.6, 78) | 6 (4, 9) | 81.4 (80.7, 82) | 6 (3, 10) | 82 (81.2, 82.7) | 10 (7, 14) |
| **Carlton** | 74.5 (73.9, 74.9) | 11 (9, 14) | 74.9 (74.3, 75.4) | 16 (14, 19) | 80.6 (80, 81.2) | 10 (7, 14) | 81 (80.3, 81.7) | 15 (12, 19) |
| **Carver** | 76.9 (76.4, 77.4) | 1 (-1, 3) | 78.6 (78, 79.1) | 1 (-1, 4) | 81.7 (81.1, 82.3) | 5 (2, 8) | 83 (82.3, 83.6) | 6 (3, 9) |
| **Cass** | 74.4 (73.8, 74.9) | 12 (9, 15) | 75.3 (74.6, 75.9) | 14 (12, 18) | 80.5 (79.9, 81.1) | 11 (8, 15) | 81.1 (80.3, 81.8) | 15 (11, 19) |
| **Chippewa** | 75 (74.4, 75.5) | 9 (6, 12) | 76.8 (76.2, 77.4) | 8 (6, 11) | 81.8 (81.1, 82.4) | 4 (1, 8) | 81.7 (81, 82.4) | 11 (8, 15) |
| **Chisago** | 76 (75.3, 76.4) | 5 (3, 7) | 76.7 (76, 77.2) | 9 (7, 11) | 80.5 (79.8, 81) | 11 (8, 15) | 81 (80.2, 81.5) | 15 (12, 20) |
| **Clay** | 75.8 (75.3, 76.3) | 5 (3, 8) | 77.3 (76.7, 77.9) | 7 (5, 9) | 81.6 (80.9, 82.2) | 5 (3, 9) | 82.6 (81.9, 83.3) | 8 (4, 11) |
| **Clearwater** | 74.4 (73.7, 74.9) | 12 (9, 15) | 74.9 (74.2, 75.4) | 16 (14, 20) | 80.3 (79.7, 80.9) | 12 (9, 16) | 81 (80.2, 81.7) | 15 (12, 20) |
| **Cook** | 75.7 (74.9, 76.3) | 6 (3, 9) | 76.3 (75.5, 76.9) | 10 (8, 13) | 80.4 (79.7, 81) | 12 (8, 16) | 81.7 (80.8, 82.5) | 12 (8, 16) |
| **Cottonwood** | 75.1 (74.4, 75.6) | 8 (6, 12) | 76.3 (75.6, 76.9) | 10 (8, 13) | 80.9 (80.3, 81.6) | 9 (5, 12) | 81.8 (81, 82.5) | 11 (8, 15) |
| **Crow Wing** | 76 (75.4, 76.5) | 4 (3, 7) | 77.3 (76.7, 77.9) | 6 (5, 9) | 81.4 (80.8, 82) | 6 (4, 9) | 81.8 (81.2, 82.3) | 11 (9, 14) |
| **Dakota** | 77.6 (77.2, 77.9) | -1 (-3, 0) | 78.7 (78.4, 79) | 1 (-1, 2) | 81.7 (81.3, 82) | 5 (3, 7) | 81.7 (81.3, 82) | 12 (10, 13) |
| **Dodge** | 75.9 (75.2, 76.5) | 5 (2, 8) | 77.5 (76.7, 78.3) | 6 (3, 9) | 81 (80.3, 81.8) | 8 (4, 13) | 82.3 (81.3, 83.2) | 9 (5, 14) |
| **Douglas** | 76.2 (75.6, 76.7) | 4 (2, 6) | 78 (77.3, 78.6) | 4 (1, 7) | 81.5 (80.9, 82.1) | 6 (3, 9) | 82.8 (82, 83.5) | 7 (4, 10) |
| **Faribault** | 75.5 (74.8, 76.1) | 6 (4, 10) | 76 (75.3, 76.6) | 11 (9, 14) | 80.5 (79.8, 81) | 11 (8, 15) | 81.8 (81, 82.5) | 11 (8, 15) |
| **Fillmore** | 75.1 (74.4, 75.6) | 9 (6, 12) | 76.4 (75.7, 77) | 10 (8, 13) | 81.3 (80.6, 81.9) | 6 (4, 10) | 82 (81.2, 82.7) | 10 (7, 14) |
| **Freeborn** | 75.6 (75.1, 76.2) | 6 (4, 9) | 76.6 (76, 77.2) | 9 (7, 11) | 80.9 (80.3, 81.5) | 9 (6, 12) | 81.7 (81, 82.3) | 12 (9, 15) |
| **Goodhue** | 76 (75.4, 76.4) | 4 (3, 7) | 77.7 (77, 78.2) | 5 (3, 8) | 80.8 (80.3, 81.3) | 9 (7, 13) | 82 (81.3, 82.5) | 10 (8, 14) |
| **Grant** | 75.4 (74.9, 75.7) | 7 (5, 10) | 76.5 (76, 77) | 9 (8, 11) | 81.4 (80.9, 81.8) | 6 (4, 9) | 82.4 (81.8, 82.9) | 9 (6, 11) |
| **Hennepin** | 76.3 (76.2, 76.5) | 3 (2, 4) | 78.1 (78, 78.3) | 3 (3, 4) | 81.2 (81.1, 81.4) | 7 (6, 8) | 82.4 (82.2, 82.6) | 9 (8, 9) |
| **Houston** | 75.8 (75.1, 76.3) | 5 (3, 9) | 76.9 (76.1, 77.6) | 8 (6, 11) | 81.2 (80.5, 81.9) | 7 (4, 11) | 81.8 (81, 82.6) | 11 (8, 15) |
| **Hubbard** | 74.5 (73.8, 75) | 11 (9, 14) | 76 (75.3, 76.6) | 11 (9, 14) | 79.9 (79.3, 80.5) | 14 (11, 18) | 81.5 (80.7, 82.2) | 13 (10, 17) |
| **Isanti** | 75.8 (75.2, 76.4) | 5 (3, 8) | 76.7 (76.1, 77.3) | 9 (7, 11) | 81.2 (80.6, 81.9) | 7 (4, 11) | 81.7 (81, 82.5) | 12 (8, 15) |
| **Itasca** | 75 (74.4, 75.5) | 9 (7, 12) | 76 (75.4, 76.5) | 11 (9, 14) | 80.3 (79.7, 80.8) | 12 (9, 16) | 81.1 (80.4, 81.7) | 15 (12, 18) |
| **Jackson** | 75.9 (75.2, 76.3) | 5 (3, 8) | 77.5 (76.8, 78.1) | 6 (4, 8) | 81.7 (81, 82.3) | 5 (2, 8) | 82.9 (82, 83.6) | 7 (3, 10) |
| **Kanabec** | 74.5 (73.7, 75.2) | 11 (8, 15) | 76.2 (75.3, 77) | 11 (8, 15) | 80.2 (79.4, 80.9) | 13 (9, 17) | 80.7 (79.8, 81.6) | 17 (12, 22) |
| **Kandiyohi** | 76.1 (75.5, 76.5) | 4 (2, 7) | 77.2 (76.5, 77.7) | 7 (5, 9) | 81.2 (80.6, 81.8) | 7 (4, 10) | 82.5 (81.8, 83.2) | 8 (5, 11) |
| **Kittson** | 74.8 (73.9, 75.4) | 10 (7, 14) | 75.3 (74.5, 75.9) | 15 (12, 18) | 80.6 (79.9, 81.3) | 11 (7, 15) | 82.4 (81.4, 83.3) | 9 (5, 13) |
| **Koochiching** | 74.5 (73.8, 75) | 11 (9, 15) | 75.3 (74.5, 75.9) | 15 (12, 18) | 79.9 (79.2, 80.4) | 15 (12, 18) | 81.1 (80.3, 81.8) | 15 (11, 19) |
| **Lac Qui Parle** | 74.7 (74, 75.2) | 10 (8, 14) | 76.5 (75.7, 77.2) | 9 (7, 13) | 81 (80.3, 81.6) | 8 (5, 12) | 82.2 (81.3, 82.9) | 9 (6, 14) |
| **Lake** | 75.7 (74.9, 76.3) | 6 (3, 9) | 76.3 (75.5, 76.9) | 10 (8, 13) | 80.4 (79.7, 81) | 12 (8, 16) | 81.7 (80.8, 82.5) | 12 (8, 16) |
| **Lake Of Woods** | 74.5 (73.8, 75) | 11 (9, 15) | 75.3 (74.5, 75.9) | 15 (12, 18) | 79.9 (79.2, 80.4) | 15 (12, 18) | 81.1 (80.3, 81.8) | 15 (11, 19) |
| **Le Sueur** | 75.9 (75.1, 76.4) | 5 (3, 8) | 77.6 (76.8, 78.3) | 5 (3, 8) | 81.3 (80.6, 81.9) | 7 (4, 10) | 81.9 (81.1, 82.7) | 11 (7, 15) |
| **Lincoln** | 74.9 (74.2, 75.5) | 9 (6, 13) | 75.1 (74.3, 75.8) | 15 (12, 19) | 80.4 (79.8, 81.1) | 11 (8, 15) | 81.8 (81, 82.6) | 11 (8, 15) |
| **Lyon** | 75.5 (74.8, 76) | 7 (4, 10) | 76.9 (76.1, 77.5) | 8 (6, 11) | 81 (80.3, 81.6) | 8 (5, 12) | 81.6 (80.8, 82.3) | 12 (9, 17) |
| **Mahnomen** | 74.4 (73.7, 74.9) | 12 (9, 15) | 74.9 (74.2, 75.4) | 16 (14, 20) | 80.3 (79.7, 80.9) | 12 (9, 16) | 81 (80.2, 81.7) | 15 (12, 20) |
| **Marshall** | 74.8 (73.9, 75.4) | 10 (7, 14) | 75.3 (74.5, 75.9) | 15 (12, 18) | 80.6 (79.9, 81.3) | 11 (7, 15) | 82.4 (81.4, 83.3) | 9 (5, 13) |
| **Martin** | 75.5 (74.8, 76) | 6 (4, 10) | 76.5 (75.8, 77.1) | 9 (7, 12) | 81.1 (80.4, 81.6) | 8 (5, 12) | 82.8 (82, 83.6) | 7 (3, 11) |
| **McLeod** | 76 (75.3, 76.5) | 4 (2, 7) | 77.3 (76.6, 77.8) | 7 (5, 9) | 81.7 (81, 82.3) | 5 (2, 8) | 82.4 (81.7, 83.1) | 8 (5, 12) |
| **Meeker** | 75 (74.3, 75.6) | 9 (6, 12) | 76.4 (75.6, 77) | 10 (8, 13) | 81 (80.2, 81.6) | 8 (5, 13) | 80.8 (80.1, 81.5) | 16 (13, 21) |
| **Mille Lacs** | 74.7 (74, 75.3) | 10 (8, 13) | 75.1 (74.4, 75.6) | 16 (13, 19) | 80.1 (79.5, 80.7) | 13 (10, 17) | 80.6 (79.8, 81.3) | 18 (14, 22) |
| **Morrison** | 74.9 (74.2, 75.4) | 10 (7, 13) | 75 (74.4, 75.5) | 16 (14, 19) | 81.1 (80.4, 81.7) | 8 (5, 12) | 81.1 (80.3, 81.8) | 15 (11, 19) |
| **Mower** | 75.8 (75.2, 76.2) | 5 (3, 8) | 77.6 (76.9, 78.2) | 5 (3, 8) | 81.8 (81.1, 82.3) | 4 (2, 8) | 82.6 (81.8, 83.2) | 8 (5, 11) |
| **Murray** | 75.6 (74.9, 76.1) | 6 (4, 10) | 75.8 (75.1, 76.4) | 12 (10, 15) | 81.2 (80.5, 81.8) | 7 (4, 11) | 82.1 (81.2, 82.8) | 10 (7, 14) |
| **Nicollet** | 77 (76.3, 77.6) | 1 (-1, 3) | 78 (77.3, 78.7) | 4 (1, 7) | 81.2 (80.5, 81.8) | 7 (4, 11) | 82.1 (81.3, 82.8) | 10 (7, 14) |
| **Nobles** | 75.9 (75.2, 76.3) | 5 (3, 8) | 77.5 (76.8, 78.1) | 6 (4, 8) | 81.7 (81, 82.3) | 5 (2, 8) | 82.9 (82, 83.6) | 7 (3, 10) |
| **Norman** | 74.4 (73.8, 74.8) | 12 (10, 14) | 75.7 (75, 76.2) | 13 (11, 16) | 80.9 (80.2, 81.4) | 9 (6, 13) | 81.2 (80.5, 81.8) | 14 (11, 18) |
| **Olmsted** | 77 (76.6, 77.4) | 0 (-1, 2) | 78.3 (77.9, 78.7) | 3 (1, 5) | 82.5 (82, 83) | 1 (-1, 3) | 83.7 (83.1, 84.1) | 3 (-1, 5) |
| **Otter Tail** | 75.4 (74.9, 75.7) | 7 (5, 10) | 76.5 (76, 77) | 9 (8, 11) | 81.4 (80.9, 81.8) | 6 (4, 9) | 82.4 (81.8, 82.9) | 9 (6, 11) |
| **Pennington** | 74.4 (73.7, 74.9) | 12 (9, 15) | 74.9 (74.2, 75.4) | 16 (14, 20) | 80.3 (79.7, 80.9) | 12 (9, 16) | 81 (80.2, 81.7) | 15 (12, 20) |
| **Pine** | 73.9 (73.3, 74.4) | 14 (12, 17) | 75.3 (74.6, 75.9) | 14 (12, 18) | 80.2 (79.5, 80.8) | 13 (9, 17) | 80.1 (79.4, 80.9) | 20 (16, 24) |
| **Pipestone** | 74.9 (74.2, 75.5) | 9 (6, 13) | 75.1 (74.3, 75.8) | 15 (12, 19) | 80.4 (79.8, 81.1) | 11 (8, 15) | 81.8 (81, 82.6) | 11 (8, 15) |
| **Polk** | 74.1 (73.6, 74.6) | 13 (11, 15) | 75.3 (74.8, 75.8) | 14 (12, 17) | 80.9 (80.3, 81.4) | 9 (6, 12) | 81.4 (80.7, 82.1) | 13 (10, 17) |
| **Pope** | 75.5 (74.8, 76) | 7 (4, 10) | 77.8 (77, 78.4) | 5 (2, 8) | 81.1 (80.4, 81.7) | 8 (5, 11) | 81.9 (81.1, 82.7) | 11 (7, 15) |
| **Ramsey** | 75.4 (75.1, 75.6) | 7 (6, 8) | 77.4 (77.2, 77.7) | 6 (5, 7) | 80.1 (79.9, 80.4) | 13 (12, 14) | 82.5 (82.2, 82.7) | 8 (7, 9) |
| **Red Lake** | 74.1 (73.6, 74.6) | 13 (11, 15) | 75.3 (74.8, 75.8) | 14 (12, 17) | 80.9 (80.3, 81.4) | 9 (6, 12) | 81.4 (80.7, 82.1) | 13 (10, 17) |
| **Redwood** | 74.8 (74.1, 75.3) | 10 (7, 13) | 76 (75.2, 76.6) | 11 (9, 15) | 81.4 (80.7, 82) | 6 (3, 10) | 81.1 (80.4, 81.8) | 15 (11, 19) |
| **Renville** | 74.9 (74.2, 75.4) | 9 (7, 13) | 76.2 (75.5, 76.8) | 10 (8, 14) | 80.7 (80, 81.4) | 10 (6, 14) | 80.9 (80, 81.6) | 16 (12, 21) |
| **Rice** | 76.1 (75.5, 76.5) | 4 (3, 6) | 77.3 (76.7, 77.7) | 7 (5, 9) | 81.7 (81.1, 82.2) | 5 (2, 8) | 82 (81.4, 82.6) | 10 (8, 13) |
| **Rock** | 75.6 (74.9, 76.1) | 6 (4, 10) | 75.8 (75.1, 76.4) | 12 (10, 15) | 81.2 (80.5, 81.8) | 7 (4, 11) | 82.1 (81.2, 82.8) | 10 (7, 14) |
| **Roseau** | 74.6 (73.9, 75.2) | 11 (8, 14) | 75.9 (75.1, 76.5) | 12 (9, 15) | 80.9 (80.1, 81.6) | 9 (5, 13) | 81.3 (80.5, 82.1) | 14 (10, 18) |
| **Scott** | 76.7 (76.1, 77.1) | 2 (0, 4) | 78.3 (77.7, 78.7) | 3 (1, 5) | 81.4 (80.8, 82) | 6 (3, 9) | 82.4 (81.7, 83.1) | 8 (6, 12) |
| **Sherburne** | 76 (75.4, 76.4) | 4 (3, 7) | 77.3 (76.8, 77.8) | 7 (5, 8) | 80.5 (79.9, 81) | 11 (8, 15) | 80.8 (80.2, 81.3) | 16 (14, 20) |
| **Sibley** | 74.9 (74.1, 75.4) | 10 (7, 13) | 76.6 (75.8, 77.3) | 9 (7, 12) | 80.9 (80.2, 81.7) | 9 (5, 13) | 81.5 (80.6, 82.4) | 12 (9, 18) |
| **St. Louis** | 74.8 (74.5, 75.1) | 10 (8, 11) | 75.8 (75.5, 76.1) | 12 (11, 13) | 80.1 (79.8, 80.4) | 14 (12, 15) | 80.9 (80.6, 81.3) | 16 (14, 18) |
| **Stearns** | 76.5 (76.1, 76.9) | 2 (1, 4) | 78.8 (78.3, 79.2) | 0 (-2, 3) | 82.8 (82.2, 83.2) | 0 (-2, 2) | 83.9 (83.3, 84.5) | 1 (-3, 4) |
| **Steele** | 76.3 (75.7, 76.9) | 3 (1, 6) | 77.7 (77, 78.3) | 5 (3, 8) | 81 (80.4, 81.6) | 8 (5, 12) | 82.5 (81.7, 83.2) | 8 (5, 12) |
| **Stevens** | 75.5 (74.8, 76) | 7 (4, 10) | 77.8 (77, 78.4) | 5 (2, 8) | 81.1 (80.4, 81.7) | 8 (5, 11) | 81.9 (81.1, 82.7) | 11 (7, 15) |
| **Swift** | 75 (74.4, 75.5) | 9 (6, 12) | 76.8 (76.2, 77.4) | 8 (6, 11) | 81.8 (81.1, 82.4) | 4 (1, 8) | 81.7 (81, 82.4) | 11 (8, 15) |
| **Todd** | 74.9 (74.3, 75.4) | 9 (7, 12) | 75.7 (75, 76.3) | 12 (10, 16) | 80.4 (79.8, 81) | 11 (8, 15) | 81.1 (80.3, 81.8) | 15 (11, 19) |
| **Traverse** | 74.7 (74, 75.2) | 10 (8, 14) | 76.5 (75.7, 77.2) | 9 (7, 13) | 81 (80.3, 81.6) | 8 (5, 12) | 82.2 (81.3, 82.9) | 9 (6, 14) |
| **Wabasha** | 75.8 (75.1, 76.3) | 5 (3, 8) | 77.1 (76.3, 77.7) | 7 (5, 10) | 81.3 (80.5, 81.9) | 7 (4, 11) | 81.8 (80.9, 82.6) | 11 (8, 16) |
| **Wadena** | 74.5 (73.8, 75) | 11 (9, 14) | 76 (75.3, 76.6) | 11 (9, 14) | 79.9 (79.3, 80.5) | 14 (11, 18) | 81.5 (80.7, 82.2) | 13 (10, 17) |
| **Waseca** | 75.4 (74.6, 75.9) | 7 (5, 11) | 77.5 (76.7, 78.3) | 6 (3, 9) | 81.1 (80.4, 81.9) | 8 (4, 12) | 81.3 (80.5, 82.1) | 14 (10, 18) |
| **Washington** | 77.5 (77.1, 77.9) | -1 (-2, 0) | 78.8 (78.4, 79.1) | 0 (-2, 2) | 81.6 (81.2, 82) | 5 (3, 7) | 82 (81.6, 82.4) | 10 (8, 12) |
| **Watonwan** | 75.1 (74.4, 75.6) | 8 (6, 12) | 76.3 (75.6, 76.9) | 10 (8, 13) | 80.9 (80.3, 81.6) | 9 (5, 12) | 81.8 (81, 82.5) | 11 (8, 15) |
| **Wilkin** | 75.4 (74.9, 75.7) | 7 (5, 10) | 76.5 (76, 77) | 9 (8, 11) | 81.4 (80.9, 81.8) | 6 (4, 9) | 82.4 (81.8, 82.9) | 9 (6, 11) |
| **Winona** | 75.3 (74.7, 75.7) | 8 (5, 10) | 77.3 (76.7, 77.9) | 7 (4, 9) | 81.2 (80.6, 81.8) | 7 (4, 10) | 82.2 (81.5, 82.9) | 10 (7, 13) |
| **Wright** | 76 (75.5, 76.4) | 4 (3, 6) | 77.8 (77.3, 78.2) | 5 (3, 7) | 81 (80.4, 81.5) | 9 (6, 12) | 82.1 (81.4, 82.7) | 10 (7, 13) |
| **Yell. Medicine** | 74.8 (74.1, 75.3) | 10 (7, 13) | 76 (75.2, 76.6) | 11 (9, 15) | 81.4 (80.7, 82) | 6 (3, 10) | 81.1 (80.4, 81.8) | 15 (11, 19) |
| **MISSISSIPPI** |  |  |  |  |  |  |  |  |
| **Adams** | 69.8 (69.2, 70.1) | 37 (33, 44) | 70.8 (70.2, 71.2) | 35 (33, 39) | 76.1 (75.6, 76.4) | 31 (29, 33) | 77.6 (77.1, 78) | 31 (30, 33) |
| **Alcorn** | 70.8 (70.3, 71.3) | 28 (26, 31) | 71.9 (71.2, 72.4) | 30 (28, 33) | 78 (77.4, 78.4) | 23 (22, 25) | 77.9 (77.3, 78.3) | 31 (29, 33) |
| **Amite** | 69 (68.3, 69.4) | 46 (43, 51) | 69.3 (68.6, 69.8) | 51 (44, 51) | 76.8 (76.2, 77.2) | 28 (26, 30) | 76.1 (75.4, 76.5) | 38 (36, 42) |
| **Attala** | 68.5 (67.8, 68.9) | 49 (47, 51) | 68.2 (67.6, 68.6) | 51 (51, 51) | 77.2 (76.6, 77.6) | 26 (24, 28) | 77.3 (76.6, 77.8) | 33 (31, 35) |
| **Benton** | 70.4 (69.8, 70.8) | 31 (28, 37) | 70.6 (69.9, 71) | 36 (34, 42) | 77.5 (77, 77.9) | 25 (23, 27) | 77.9 (77.2, 78.5) | 30 (28, 33) |
| **Bolivar** | 67.6 (66.9, 68) | 51 (51, 51) | 67.9 (67.2, 68.3) | 51 (51, 51) | 75.1 (74.6, 75.5) | 37 (34, 40) | 75.2 (74.6, 75.7) | 43 (40, 47) |
| **Calhoun** | 70.1 (69.4, 70.6) | 33 (29, 42) | 70.6 (69.8, 71.1) | 36 (34, 44) | 77.6 (77, 78.1) | 24 (23, 27) | 77.9 (77.1, 78.4) | 31 (28, 33) |
| **Carroll** | 69.3 (68.6, 69.6) | 44 (39, 48) | 70.7 (70.1, 71.2) | 36 (33, 40) | 77.3 (76.8, 77.7) | 26 (24, 28) | 77.9 (77.2, 78.4) | 30 (29, 33) |
| **Chickasaw** | 68.5 (67.9, 68.9) | 49 (46, 51) | 69.8 (69.2, 70.3) | 43 (38, 51) | 76.7 (76.1, 77.1) | 28 (26, 31) | 77.5 (76.9, 78) | 32 (30, 34) |
| **Choctaw** | 70.8 (70.1, 71.3) | 28 (26, 32) | 71.1 (70.4, 71.6) | 33 (31, 37) | 77.2 (76.5, 77.6) | 26 (24, 29) | 77.3 (76.6, 77.8) | 33 (31, 36) |
| **Claiborne** | 66.5 (65.7, 67.1) | 51 (51, 51) | 67.8 (67, 68.4) | 51 (51, 51) | 74.3 (73.5, 74.9) | 42 (39, 45) | 74.5 (73.7, 75.2) | 48 (44, 51) |
| **Clarke** | 70.6 (69.9, 71) | 29 (27, 35) | 71.2 (70.4, 71.6) | 33 (31, 37) | 77.4 (76.9, 77.9) | 25 (24, 27) | 77.6 (76.9, 78) | 32 (30, 34) |
| **Clay** | 69.1 (68.4, 69.5) | 45 (41, 50) | 71 (70.3, 71.5) | 34 (32, 38) | 76.7 (76, 77.1) | 28 (26, 31) | 77.8 (77.1, 78.3) | 31 (29, 33) |
| **Coahoma** | 66.7 (66, 67.1) | 51 (51, 51) | 66.8 (66, 67.3) | 51 (51, 51) | 74.7 (74.1, 75.1) | 40 (37, 43) | 75 (74.4, 75.5) | 44 (41, 49) |
| **Copiah** | 69.4 (68.8, 69.9) | 42 (36, 47) | 69.6 (69, 70) | 47 (41, 51) | 76.4 (75.9, 76.9) | 29 (27, 32) | 76.7 (76.1, 77.2) | 35 (33, 38) |
| **Covington** | 69.1 (68.4, 69.5) | 46 (41, 50) | 69.2 (68.6, 69.6) | 51 (46, 51) | 77.4 (76.8, 77.9) | 25 (23, 28) | 77 (76.4, 77.5) | 34 (32, 36) |
| **De Soto** | 72.6 (72.2, 72.9) | 20 (19, 22) | 73.9 (73.5, 74.3) | 21 (19, 23) | 78.9 (78.4, 79.3) | 20 (18, 22) | 78.8 (78.4, 79.2) | 27 (25, 28) |
| **Forrest** | 70.4 (70, 70.7) | 30 (29, 34) | 71.9 (71.4, 72.2) | 30 (29, 32) | 76.8 (76.4, 77.1) | 28 (26, 29) | 77.5 (77.1, 77.9) | 32 (30, 33) |
| **Franklin** | 69.8 (69.2, 70.1) | 37 (33, 44) | 70.8 (70.2, 71.2) | 35 (33, 39) | 76.1 (75.6, 76.4) | 31 (29, 33) | 77.6 (77.1, 78) | 31 (30, 33) |
| **George** | 70.8 (70.2, 71.3) | 28 (26, 32) | 70.4 (69.8, 70.9) | 37 (35, 44) | 77.4 (76.8, 78) | 25 (23, 27) | 77 (76.4, 77.6) | 34 (32, 36) |
| **Greene** | 70.5 (69.9, 70.9) | 30 (28, 36) | 70.7 (70.1, 71.1) | 36 (33, 40) | 77.3 (76.7, 77.8) | 26 (24, 28) | 76.6 (76, 77.1) | 35 (33, 38) |
| **Grenada** | 69.2 (68.6, 69.6) | 45 (39, 48) | 68.8 (68.1, 69.3) | 51 (51, 51) | 76.6 (76.1, 77.1) | 28 (26, 31) | 76.7 (76, 77.2) | 35 (33, 38) |
| **Hancock** | 72.3 (71.7, 72.7) | 22 (20, 24) | 73.9 (73.2, 74.4) | 21 (19, 24) | 78.4 (77.8, 78.9) | 21 (19, 24) | 79.7 (79, 80.2) | 23 (20, 26) |
| **Harrison** | 71.1 (70.9, 71.4) | 26 (25, 28) | 71 (70.7, 71.3) | 34 (33, 36) | 77.1 (76.8, 77.4) | 26 (25, 28) | 78.6 (78.3, 78.9) | 28 (27, 29) |
| **Hinds** | 69.6 (69.3, 69.8) | 40 (36, 44) | 71.1 (70.8, 71.3) | 34 (33, 35) | 76.7 (76.4, 76.9) | 28 (27, 29) | 79 (78.6, 79.3) | 26 (25, 28) |
| **Holmes** | 64.6 (63.8, 65.2) | 51 (51, 51) | 65.9 (65.1, 66.5) | 51 (51, 51) | 74 (73.4, 74.5) | 43 (41, 46) | 73.5 (72.7, 74) | 51 (50, 51) |
| **Humphreys** | 66.5 (65.7, 67) | 51 (51, 51) | 66.5 (65.6, 67) | 51 (51, 51) | 74.3 (73.7, 74.8) | 42 (39, 45) | 74.1 (73.3, 74.7) | 50 (47, 51) |
| **Issaquena** | 68.4 (67.7, 68.8) | 50 (47, 51) | 69.9 (69.2, 70.3) | 43 (38, 51) | 75.5 (75, 75.9) | 34 (32, 38) | 75.9 (75.3, 76.4) | 39 (36, 43) |
| **Itawamba** | 71 (70.3, 71.5) | 27 (25, 31) | 71.6 (70.8, 72.1) | 32 (29, 35) | 78.3 (77.7, 78.9) | 22 (20, 24) | 77.3 (76.6, 77.9) | 33 (31, 35) |
| **Jackson** | 71.4 (71.1, 71.7) | 25 (24, 27) | 72.1 (71.7, 72.4) | 30 (28, 31) | 77.5 (77.1, 77.8) | 25 (24, 26) | 77.7 (77.3, 78) | 31 (30, 33) |
| **Jasper** | 68.6 (67.9, 69) | 48 (46, 51) | 70.5 (69.7, 71) | 37 (34, 45) | 75.8 (75.2, 76.3) | 32 (30, 36) | 77.2 (76.5, 77.7) | 33 (31, 36) |
| **Jefferson** | 66.5 (65.7, 67.1) | 51 (51, 51) | 67.8 (67, 68.4) | 51 (51, 51) | 74.3 (73.5, 74.9) | 42 (39, 45) | 74.5 (73.7, 75.2) | 48 (44, 51) |
| **Jefferson Davis** | 69.1 (68.5, 69.5) | 45 (41, 49) | 69.9 (69.3, 70.4) | 42 (38, 51) | 76.3 (75.7, 76.7) | 30 (28, 33) | 76.9 (76.3, 77.4) | 34 (32, 37) |
| **Jones** | 71.3 (70.8, 71.6) | 26 (24, 28) | 71.8 (71.4, 72.2) | 31 (29, 33) | 78.1 (77.7, 78.5) | 23 (21, 24) | 77.9 (77.5, 78.3) | 30 (29, 32) |
| **Kemper** | 67.8 (67.1, 68.3) | 51 (51, 51) | 68.9 (68.1, 69.4) | 51 (50, 51) | 75.9 (75.3, 76.3) | 32 (29, 36) | 76.5 (75.8, 77) | 36 (34, 40) |
| **Lafayette** | 72.5 (72, 72.9) | 21 (19, 23) | 72.5 (72, 72.8) | 28 (26, 30) | 78.9 (78.3, 79.3) | 20 (18, 22) | 79.4 (78.8, 80) | 24 (21, 27) |
| **Lamar** | 73.5 (72.9, 73.9) | 16 (14, 19) | 74 (73.4, 74.4) | 21 (19, 24) | 79.2 (78.6, 79.7) | 18 (15, 21) | 78.9 (78.3, 79.4) | 27 (25, 29) |
| **Lauderdale** | 70.3 (69.9, 70.6) | 31 (29, 36) | 70 (69.6, 70.4) | 41 (38, 46) | 77.2 (76.8, 77.5) | 26 (25, 28) | 77.1 (76.7, 77.4) | 33 (32, 35) |
| **Lawrence** | 69.1 (68.5, 69.5) | 45 (41, 49) | 69.9 (69.3, 70.4) | 42 (38, 51) | 76.3 (75.7, 76.7) | 30 (28, 33) | 76.9 (76.3, 77.4) | 34 (32, 37) |
| **Leake** | 69.1 (68.4, 69.5) | 46 (42, 49) | 68.4 (67.8, 68.9) | 51 (51, 51) | 76.4 (75.9, 76.9) | 29 (27, 32) | 76.2 (75.5, 76.7) | 37 (35, 41) |
| **Lee** | 71 (70.5, 71.3) | 27 (26, 30) | 70.9 (70.5, 71.2) | 35 (33, 37) | 78.4 (77.9, 78.7) | 22 (20, 23) | 77 (76.5, 77.4) | 34 (32, 36) |
| **Leflore** | 67.9 (67.3, 68.3) | 51 (51, 51) | 68.5 (67.8, 68.9) | 51 (51, 51) | 75.3 (74.7, 75.7) | 36 (33, 39) | 75.5 (74.9, 76) | 41 (38, 45) |
| **Lincoln** | 70.7 (70.1, 71) | 29 (27, 33) | 71.8 (71.2, 72.2) | 31 (29, 33) | 77.5 (76.9, 77.9) | 25 (23, 27) | 77.9 (77.4, 78.4) | 30 (29, 32) |
| **Lowndes** | 70.7 (70.3, 71.1) | 28 (27, 31) | 72.5 (72.1, 72.9) | 28 (26, 30) | 77.9 (77.5, 78.3) | 23 (22, 25) | 78.4 (77.9, 78.8) | 29 (27, 31) |
| **Madison** | 72 (71.6, 72.3) | 23 (21, 25) | 69.9 (69.5, 70.2) | 42 (39, 48) | 77.8 (77.4, 78.2) | 24 (22, 25) | 75.9 (75.6, 76.2) | 39 (37, 41) |
| **Marion** | 68.7 (68.1, 69.1) | 48 (45, 51) | 68.7 (68, 69.1) | 51 (51, 51) | 76.7 (76.1, 77.1) | 28 (26, 31) | 76.2 (75.6, 76.6) | 37 (35, 41) |
| **Marshall** | 68.3 (67.7, 68.6) | 51 (48, 51) | 68.5 (68, 68.9) | 51 (51, 51) | 75.7 (75.2, 76.2) | 33 (30, 36) | 76 (75.5, 76.4) | 38 (36, 41) |
| **Monroe** | 69.9 (69.4, 70.3) | 35 (31, 43) | 71.2 (70.6, 71.6) | 33 (32, 36) | 77.4 (76.8, 77.8) | 25 (24, 27) | 78.3 (77.7, 78.8) | 29 (27, 31) |
| **Montgomery** | 69.3 (68.6, 69.6) | 44 (39, 48) | 70.7 (70.1, 71.2) | 36 (33, 40) | 77.3 (76.8, 77.7) | 26 (24, 28) | 77.9 (77.2, 78.4) | 30 (29, 33) |
| **Neshoba** | 71.4 (70.8, 71.8) | 26 (24, 28) | 71.1 (70.5, 71.6) | 34 (32, 37) | 77.7 (77.2, 78.2) | 24 (22, 26) | 77.3 (76.7, 77.8) | 32 (31, 35) |
| **Newton** | 70.3 (69.7, 70.8) | 31 (28, 39) | 71.8 (71.1, 72.2) | 31 (29, 34) | 77.3 (76.7, 77.8) | 26 (24, 28) | 77.9 (77.3, 78.4) | 30 (28, 33) |
| **Noxubee** | 67.8 (67.1, 68.3) | 51 (51, 51) | 68.9 (68.1, 69.4) | 51 (50, 51) | 75.9 (75.3, 76.3) | 32 (29, 36) | 76.5 (75.8, 77) | 36 (34, 40) |
| **Oktibbeha** | 72.2 (71.6, 72.5) | 22 (21, 24) | 73.9 (73.3, 74.3) | 21 (19, 24) | 77.9 (77.3, 78.2) | 24 (22, 26) | 79.3 (78.7, 79.8) | 25 (22, 28) |
| **Panola** | 68.3 (67.7, 68.7) | 51 (48, 51) | 68.8 (68.2, 69.2) | 51 (51, 51) | 75.9 (75.4, 76.3) | 32 (30, 35) | 75.7 (75.2, 76.1) | 40 (38, 44) |
| **Pearl River** | 71.2 (70.7, 71.6) | 26 (24, 29) | 71.8 (71.2, 72.2) | 31 (29, 33) | 78.1 (77.6, 78.5) | 23 (21, 25) | 78 (77.4, 78.5) | 30 (28, 32) |
| **Perry** | 70.5 (69.9, 70.9) | 30 (28, 36) | 70.7 (70.1, 71.1) | 36 (33, 40) | 77.3 (76.7, 77.8) | 26 (24, 28) | 76.6 (76, 77.1) | 35 (33, 38) |
| **Pike** | 69.7 (69.1, 70.1) | 38 (33, 45) | 69.9 (69.3, 70.3) | 43 (38, 51) | 76.3 (75.8, 76.7) | 30 (28, 32) | 76.5 (76, 76.9) | 36 (34, 38) |
| **Pontotoc** | 71 (70.4, 71.5) | 27 (25, 30) | 72.1 (71.4, 72.6) | 30 (27, 32) | 78.5 (77.9, 79.1) | 21 (19, 23) | 79.4 (78.7, 80.1) | 24 (20, 27) |
| **Prentiss** | 70.6 (69.9, 71.1) | 29 (27, 36) | 71.3 (70.6, 71.8) | 33 (30, 36) | 78.4 (77.7, 78.9) | 22 (20, 24) | 78.9 (78, 79.5) | 27 (24, 30) |
| **Quitman** | 65.9 (65.1, 66.4) | 51 (51, 51) | 66 (65.2, 66.5) | 51 (51, 51) | 74.1 (73.4, 74.6) | 43 (40, 46) | 74.1 (73.4, 74.6) | 50 (47, 51) |
| **Rankin** | 73.2 (72.8, 73.6) | 17 (16, 19) | 74.8 (74.3, 75.2) | 17 (15, 19) | 79.1 (78.7, 79.5) | 19 (17, 20) | 80.9 (80.4, 81.3) | 16 (14, 19) |
| **Scott** | 68.9 (68.3, 69.3) | 47 (44, 51) | 69.3 (68.6, 69.7) | 51 (44, 51) | 76.6 (76, 77) | 28 (27, 31) | 77.8 (77.1, 78.3) | 31 (29, 33) |
| **Sharkey** | 66.5 (65.7, 67) | 51 (51, 51) | 66.5 (65.6, 67) | 51 (51, 51) | 74.3 (73.7, 74.8) | 42 (39, 45) | 74.1 (73.3, 74.7) | 50 (47, 51) |
| **Simpson** | 69.6 (69, 70) | 40 (34, 46) | 70.3 (69.7, 70.8) | 38 (35, 45) | 76.5 (76, 76.9) | 29 (27, 31) | 76.4 (75.9, 76.9) | 36 (34, 39) |
| **Smith** | 71 (70.2, 71.4) | 27 (25, 32) | 69.6 (68.9, 70.1) | 46 (40, 51) | 77.7 (77.1, 78.2) | 24 (22, 27) | 77 (76.3, 77.5) | 34 (32, 37) |
| **Stone** | 70.8 (70.2, 71.3) | 28 (26, 32) | 70.4 (69.8, 70.9) | 37 (35, 44) | 77.4 (76.8, 78) | 25 (23, 27) | 77 (76.4, 77.6) | 34 (32, 36) |
| **Sunflower** | 67.3 (66.7, 67.8) | 51 (51, 51) | 67.1 (66.4, 67.5) | 51 (51, 51) | 74.5 (73.9, 74.9) | 41 (38, 44) | 73.6 (72.9, 74) | 51 (50, 51) |
| **Tallahatchie** | 68.1 (67.4, 68.5) | 51 (49, 51) | 68.6 (67.9, 69.1) | 51 (51, 51) | 76.8 (76.3, 77.2) | 27 (26, 30) | 76.3 (75.7, 76.8) | 36 (35, 40) |
| **Tate** | 70.9 (70.3, 71.4) | 28 (25, 31) | 72 (71.3, 72.5) | 30 (28, 33) | 77.4 (76.9, 77.9) | 25 (24, 27) | 77.2 (76.6, 77.6) | 33 (31, 35) |
| **Tippah** | 70.4 (69.8, 70.8) | 31 (28, 37) | 70.6 (69.9, 71) | 36 (34, 42) | 77.5 (77, 77.9) | 25 (23, 27) | 77.9 (77.2, 78.5) | 30 (28, 33) |
| **Tishomingo** | 70.4 (69.7, 71) | 30 (27, 38) | 70.8 (70, 71.4) | 35 (32, 40) | 78.3 (77.7, 78.9) | 22 (20, 24) | 77.8 (77.1, 78.4) | 31 (28, 33) |
| **Tunica** | 65.9 (65.1, 66.4) | 51 (51, 51) | 66 (65.2, 66.5) | 51 (51, 51) | 74.1 (73.4, 74.6) | 43 (40, 46) | 74.1 (73.4, 74.6) | 50 (47, 51) |
| **Union** | 71.7 (71, 72.1) | 24 (22, 27) | 72.3 (71.6, 72.8) | 29 (27, 32) | 78.6 (78, 79.1) | 21 (19, 23) | 78.4 (77.8, 79) | 28 (26, 31) |
| **Walthall** | 69.2 (68.5, 69.7) | 45 (39, 49) | 69.9 (69.2, 70.4) | 42 (37, 51) | 76.4 (75.8, 76.8) | 29 (27, 32) | 76.9 (76.2, 77.5) | 34 (32, 37) |
| **Warren** | 70.6 (70.1, 70.9) | 29 (27, 33) | 71.1 (70.6, 71.4) | 34 (32, 36) | 77.3 (76.8, 77.6) | 26 (25, 28) | 77.3 (76.8, 77.7) | 33 (31, 35) |
| **Washington** | 67.2 (66.7, 67.6) | 51 (51, 51) | 68.1 (67.6, 68.5) | 51 (51, 51) | 75.1 (74.6, 75.4) | 37 (35, 40) | 75.3 (74.9, 75.7) | 42 (40, 46) |
| **Wayne** | 69.3 (68.6, 69.7) | 44 (38, 48) | 71.1 (70.4, 71.6) | 34 (32, 37) | 76.5 (75.9, 76.9) | 29 (27, 32) | 77.2 (76.5, 77.7) | 33 (31, 36) |
| **Webster** | 70.8 (70.1, 71.3) | 28 (26, 32) | 71.1 (70.4, 71.6) | 33 (31, 37) | 77.2 (76.5, 77.6) | 26 (24, 29) | 77.3 (76.6, 77.8) | 33 (31, 36) |
| **Wilkinson** | 69 (68.3, 69.4) | 46 (43, 51) | 69.3 (68.6, 69.8) | 51 (44, 51) | 76.8 (76.2, 77.2) | 28 (26, 30) | 76.1 (75.4, 76.5) | 38 (36, 42) |
| **Winston** | 70.6 (70, 71.1) | 29 (27, 34) | 70.4 (69.7, 70.8) | 37 (35, 45) | 77.3 (76.7, 77.7) | 26 (24, 28) | 78.6 (77.9, 79.2) | 28 (25, 30) |
| **Yalobusha** | 68.1 (67.4, 68.5) | 51 (49, 51) | 68.6 (67.9, 69.1) | 51 (51, 51) | 76.8 (76.3, 77.2) | 27 (26, 30) | 76.3 (75.7, 76.8) | 36 (35, 40) |
| **Yazoo** | 68.4 (67.7, 68.8) | 50 (47, 51) | 69.9 (69.2, 70.3) | 43 (38, 51) | 75.5 (75, 75.9) | 34 (32, 38) | 75.9 (75.3, 76.4) | 39 (36, 43) |
| **MISSOURI** |  |  |  |  |  |  |  |  |
| **Adair** | 73.8 (73.2, 74.3) | 14 (12, 17) | 74.8 (74.1, 75.3) | 17 (14, 20) | 80.5 (79.8, 81) | 11 (8, 15) | 80.2 (79.4, 80.7) | 20 (17, 24) |
| **Andrew** | 74.1 (73.4, 74.6) | 13 (11, 16) | 74.8 (74.2, 75.3) | 17 (14, 20) | 80.1 (79.5, 80.7) | 13 (10, 17) | 79.6 (78.8, 80.2) | 23 (20, 27) |
| **Atchison** | 74.9 (74.3, 75.4) | 9 (7, 12) | 76.2 (75.5, 76.7) | 10 (9, 13) | 80.5 (79.8, 81) | 11 (8, 15) | 80.7 (79.9, 81.2) | 17 (14, 21) |
| **Audrain** | 73.2 (72.5, 73.7) | 17 (15, 21) | 73.6 (72.9, 74.2) | 22 (20, 26) | 79.9 (79.3, 80.5) | 14 (11, 18) | 79.3 (78.5, 79.9) | 25 (22, 28) |
| **Barry** | 73.1 (72.5, 73.7) | 18 (15, 21) | 72.7 (72.1, 73.3) | 27 (24, 30) | 79.5 (78.9, 80) | 17 (14, 20) | 79.2 (78.5, 79.8) | 25 (22, 28) |
| **Barton** | 73.4 (72.7, 73.9) | 17 (14, 20) | 73.9 (73.2, 74.5) | 21 (18, 24) | 79.1 (78.5, 79.7) | 19 (16, 21) | 80 (79.2, 80.7) | 21 (17, 25) |
| **Bates** | 73.1 (72.3, 73.7) | 18 (15, 22) | 73.8 (73, 74.4) | 21 (19, 25) | 79.4 (78.7, 80) | 17 (14, 20) | 79.5 (78.7, 80.2) | 24 (20, 27) |
| **Benton** | 73 (72.2, 73.6) | 18 (15, 22) | 73 (72.2, 73.6) | 25 (22, 29) | 78.5 (77.9, 79.1) | 21 (19, 23) | 78.8 (78.1, 79.5) | 27 (24, 30) |
| **Bollinger** | 71.9 (71.1, 72.4) | 23 (21, 27) | 72.7 (71.9, 73.3) | 27 (24, 30) | 78.6 (77.9, 79.2) | 21 (18, 23) | 78.7 (77.9, 79.3) | 27 (25, 30) |
| **Boone** | 75 (74.6, 75.3) | 9 (7, 11) | 76.5 (76.1, 76.9) | 9 (8, 11) | 80.3 (79.9, 80.7) | 12 (10, 15) | 80.5 (80, 80.9) | 18 (16, 21) |
| **Buchanan** | 73.2 (72.8, 73.6) | 17 (15, 19) | 73.8 (73.4, 74.2) | 21 (20, 23) | 79.3 (78.9, 79.7) | 18 (16, 20) | 79.6 (79.1, 80) | 23 (21, 26) |
| **Butler** | 70.9 (70.4, 71.3) | 28 (26, 31) | 70.7 (70.1, 71.2) | 36 (33, 40) | 77.7 (77.2, 78.1) | 24 (23, 26) | 77.8 (77.2, 78.2) | 31 (29, 33) |
| **Caldwell** | 73.6 (72.8, 74.2) | 15 (13, 19) | 74.5 (73.7, 75.1) | 18 (15, 22) | 80.1 (79.4, 80.7) | 14 (10, 17) | 80.4 (79.5, 81.1) | 19 (15, 24) |
| **Callaway** | 73.6 (73, 74.1) | 15 (13, 18) | 73.9 (73.3, 74.4) | 21 (19, 24) | 79.3 (78.7, 79.9) | 18 (15, 20) | 80.1 (79.3, 80.7) | 21 (17, 25) |
| **Camden** | 75.1 (74.5, 75.7) | 8 (6, 11) | 75 (74.3, 75.6) | 16 (13, 19) | 80.6 (80, 81.2) | 10 (7, 14) | 81 (80.2, 81.6) | 15 (12, 20) |
| **Cape Girardeau** | 74.1 (73.6, 74.4) | 13 (12, 15) | 75 (74.5, 75.4) | 16 (14, 18) | 80.1 (79.6, 80.5) | 14 (11, 16) | 80.2 (79.7, 80.7) | 20 (17, 23) |
| **Carroll** | 73.6 (72.8, 74.2) | 15 (13, 19) | 74.5 (73.7, 75.1) | 18 (15, 22) | 80.1 (79.4, 80.7) | 14 (10, 17) | 80.4 (79.5, 81.1) | 19 (15, 24) |
| **Carter** | 70.3 (69.5, 70.8) | 31 (28, 42) | 70.4 (69.7, 71.1) | 37 (34, 46) | 77.2 (76.6, 77.7) | 26 (24, 28) | 77.3 (76.6, 77.9) | 32 (30, 35) |
| **Cass** | 74.9 (74.4, 75.3) | 9 (7, 12) | 75 (74.6, 75.4) | 16 (14, 18) | 79.9 (79.4, 80.4) | 14 (12, 17) | 79.4 (79, 79.9) | 24 (22, 26) |
| **Cedar** | 73.4 (72.7, 73.9) | 17 (14, 20) | 73.9 (73.2, 74.5) | 21 (18, 24) | 79.1 (78.5, 79.7) | 19 (16, 21) | 80 (79.2, 80.7) | 21 (17, 25) |
| **Chariton** | 73.9 (73.2, 74.4) | 14 (12, 17) | 75.2 (74.4, 75.8) | 15 (12, 19) | 79.8 (79.2, 80.4) | 15 (12, 18) | 79.9 (79.2, 80.6) | 21 (18, 25) |
| **Christian** | 74.6 (74, 75.1) | 11 (9, 14) | 75.1 (74.5, 75.5) | 15 (13, 18) | 80.5 (79.9, 81.1) | 11 (8, 15) | 80.1 (79.5, 80.6) | 20 (17, 24) |
| **Clark** | 73.2 (72.4, 73.7) | 17 (15, 21) | 74 (73.3, 74.6) | 20 (18, 24) | 79.3 (78.7, 79.9) | 18 (15, 21) | 80.2 (79.4, 81) | 20 (16, 24) |
| **Clay** | 75.1 (74.7, 75.4) | 9 (7, 10) | 76.1 (75.7, 76.4) | 11 (10, 12) | 79.6 (79.2, 79.9) | 16 (15, 18) | 80.3 (79.9, 80.6) | 20 (18, 22) |
| **Clinton** | 74.2 (73.4, 74.8) | 13 (10, 16) | 74.2 (73.5, 74.8) | 19 (17, 23) | 80 (79.2, 80.6) | 14 (10, 18) | 80.1 (79.3, 80.8) | 20 (16, 25) |
| **Cole** | 74.4 (73.9, 74.7) | 12 (10, 14) | 75.7 (75.2, 76.2) | 12 (11, 15) | 79.8 (79.3, 80.2) | 15 (13, 18) | 80.5 (79.9, 81.1) | 18 (15, 21) |
| **Cooper** | 74.1 (73.5, 74.6) | 13 (11, 16) | 74.4 (73.7, 74.8) | 19 (17, 22) | 79.3 (78.7, 79.8) | 18 (15, 20) | 79.9 (79.2, 80.5) | 22 (18, 25) |
| **Crawford** | 73.1 (72.4, 73.7) | 18 (15, 21) | 73.1 (72.3, 73.7) | 25 (22, 29) | 78.8 (78.1, 79.3) | 20 (18, 23) | 79.2 (78.4, 79.9) | 25 (22, 29) |
| **Dade** | 72.6 (72, 73.1) | 20 (18, 23) | 73.7 (73, 74.3) | 22 (19, 25) | 79.7 (79.1, 80.2) | 16 (13, 19) | 79 (78.4, 79.7) | 26 (23, 29) |
| **Dallas** | 73.1 (72.3, 73.7) | 18 (15, 22) | 73.1 (72.2, 73.8) | 25 (22, 29) | 78.8 (78.1, 79.5) | 20 (17, 23) | 78.7 (77.8, 79.4) | 28 (24, 31) |
| **Daviess** | 73.3 (72.6, 73.8) | 17 (14, 20) | 74.9 (74.1, 75.5) | 16 (13, 20) | 79.8 (79.2, 80.3) | 15 (12, 18) | 80.2 (79.4, 80.9) | 20 (16, 24) |
| **De Kalb** | 74.1 (73.4, 74.6) | 13 (11, 16) | 74.8 (74.2, 75.3) | 17 (14, 20) | 80.1 (79.5, 80.7) | 13 (10, 17) | 79.6 (78.8, 80.2) | 23 (20, 27) |
| **Dent** | 72.3 (71.6, 72.9) | 22 (19, 25) | 72.8 (71.9, 73.5) | 26 (23, 30) | 78.1 (77.5, 78.7) | 23 (20, 25) | 78.6 (77.8, 79.4) | 28 (24, 31) |
| **Douglas** | 72.8 (71.9, 73.3) | 20 (17, 23) | 72.5 (71.7, 73.1) | 28 (25, 31) | 79.3 (78.6, 79.8) | 18 (15, 21) | 79.3 (78.4, 79.9) | 25 (21, 28) |
| **Dunklin** | 70.2 (69.5, 70.6) | 32 (29, 41) | 69.8 (69.2, 70.4) | 43 (38, 51) | 77.2 (76.7, 77.7) | 26 (24, 28) | 76.6 (76, 77.1) | 35 (33, 38) |
| **Franklin** | 73.6 (73.1, 73.9) | 16 (14, 18) | 74.2 (73.8, 74.6) | 19 (18, 22) | 78.9 (78.4, 79.3) | 20 (18, 21) | 79.4 (78.9, 79.8) | 24 (22, 27) |
| **Gasconade** | 73 (72.3, 73.5) | 18 (16, 22) | 73.7 (72.9, 74.2) | 22 (19, 26) | 79.5 (78.9, 80) | 17 (14, 20) | 79.1 (78.4, 79.7) | 26 (23, 28) |
| **Gentry** | 73.3 (72.6, 73.8) | 17 (14, 20) | 74.9 (74.1, 75.5) | 16 (13, 20) | 79.8 (79.2, 80.3) | 15 (12, 18) | 80.2 (79.4, 80.9) | 20 (16, 24) |
| **Greene** | 73.7 (73.4, 74) | 15 (14, 16) | 74.7 (74.4, 75) | 17 (16, 19) | 80.1 (79.8, 80.4) | 13 (12, 15) | 80.7 (80.3, 81) | 17 (15, 19) |
| **Grundy** | 73 (72.2, 73.5) | 18 (16, 22) | 73.9 (73.1, 74.5) | 21 (18, 25) | 79.5 (78.9, 80.1) | 17 (14, 20) | 80 (79.2, 80.7) | 21 (17, 25) |
| **Harrison** | 73.3 (72.6, 73.8) | 17 (14, 20) | 74.9 (74.1, 75.5) | 16 (13, 20) | 79.8 (79.2, 80.3) | 15 (12, 18) | 80.2 (79.4, 80.9) | 20 (16, 24) |
| **Henry** | 73.6 (72.9, 74.1) | 15 (13, 19) | 73.7 (72.9, 74.2) | 22 (19, 26) | 79.5 (78.8, 80) | 17 (14, 20) | 78.9 (78.2, 79.4) | 27 (24, 29) |
| **Hickory** | 72.5 (71.7, 73) | 21 (18, 24) | 72.8 (72, 73.4) | 26 (23, 30) | 79.5 (78.8, 80.1) | 17 (14, 20) | 79 (78.3, 79.7) | 26 (23, 29) |
| **Holt** | 74.9 (74.3, 75.4) | 9 (7, 12) | 76.2 (75.5, 76.7) | 10 (9, 13) | 80.5 (79.8, 81) | 11 (8, 15) | 80.7 (79.9, 81.2) | 17 (14, 21) |
| **Howard** | 73.9 (73.2, 74.4) | 14 (12, 17) | 75.2 (74.4, 75.8) | 15 (12, 19) | 79.8 (79.2, 80.4) | 15 (12, 18) | 79.9 (79.2, 80.6) | 21 (18, 25) |
| **Howell** | 72.8 (72.1, 73.2) | 20 (17, 22) | 72.2 (71.5, 72.7) | 29 (27, 32) | 79 (78.4, 79.5) | 19 (17, 21) | 79 (78.3, 79.5) | 26 (24, 29) |
| **Iron** | 70.3 (69.5, 70.8) | 31 (28, 42) | 70.4 (69.7, 71.1) | 37 (34, 46) | 77.2 (76.6, 77.7) | 26 (24, 28) | 77.3 (76.6, 77.9) | 32 (30, 35) |
| **Jackson** | 72.3 (72.2, 72.5) | 21 (21, 22) | 73.2 (73.1, 73.4) | 24 (23, 25) | 78.5 (78.4, 78.7) | 21 (20, 22) | 79.2 (79, 79.4) | 25 (24, 26) |
| **Jasper** | 73.1 (72.6, 73.4) | 18 (16, 20) | 73.1 (72.7, 73.5) | 25 (23, 27) | 78.7 (78.3, 79.1) | 20 (19, 22) | 78.9 (78.5, 79.3) | 26 (25, 28) |
| **Jefferson** | 73 (72.6, 73.3) | 18 (17, 20) | 74.1 (73.8, 74.4) | 20 (19, 22) | 78.3 (78, 78.6) | 22 (21, 23) | 78.4 (78.1, 78.7) | 28 (27, 30) |
| **Johnson** | 75 (74.3, 75.4) | 9 (7, 12) | 74.6 (74, 75) | 18 (16, 20) | 80.1 (79.5, 80.7) | 13 (10, 17) | 80.1 (79.4, 80.7) | 21 (17, 24) |
| **Knox** | 73.5 (72.9, 74) | 16 (13, 19) | 73.8 (73.2, 74.3) | 21 (19, 25) | 79.3 (78.8, 79.8) | 18 (15, 20) | 79.8 (79.1, 80.5) | 22 (18, 26) |
| **Laclede** | 72.8 (72.1, 73.3) | 19 (17, 22) | 73.4 (72.7, 73.9) | 23 (21, 27) | 78.3 (77.7, 78.8) | 22 (20, 24) | 78.8 (78.1, 79.4) | 27 (24, 30) |
| **Lafayette** | 74 (73.4, 74.5) | 13 (11, 16) | 73.9 (73.2, 74.4) | 21 (19, 24) | 79.5 (78.9, 80) | 17 (14, 20) | 79.9 (79.3, 80.6) | 21 (18, 25) |
| **Lawrence** | 72.9 (72.2, 73.4) | 19 (16, 22) | 73.3 (72.6, 73.8) | 24 (22, 27) | 79.4 (78.8, 80) | 17 (14, 20) | 79.4 (78.7, 80) | 24 (21, 27) |
| **Lewis** | 73.2 (72.4, 73.7) | 17 (15, 21) | 74 (73.3, 74.6) | 20 (18, 24) | 79.3 (78.7, 79.9) | 18 (15, 21) | 80.2 (79.4, 81) | 20 (16, 24) |
| **Lincoln** | 73.9 (73.2, 74.4) | 14 (12, 17) | 73.7 (73, 74.1) | 22 (20, 25) | 79.4 (78.8, 80) | 17 (14, 20) | 79 (78.4, 79.5) | 26 (24, 29) |
| **Linn** | 73.7 (73.1, 74.2) | 15 (13, 18) | 74.2 (73.5, 74.8) | 20 (17, 23) | 80.4 (79.7, 80.9) | 12 (9, 16) | 80.2 (79.4, 80.8) | 20 (16, 24) |
| **Livingston** | 73.7 (73.1, 74.2) | 15 (13, 18) | 74.2 (73.5, 74.8) | 20 (17, 23) | 80.4 (79.7, 80.9) | 12 (9, 16) | 80.2 (79.4, 80.8) | 20 (16, 24) |
| **Macon** | 73.5 (72.9, 74) | 16 (13, 19) | 73.8 (73.2, 74.3) | 21 (19, 25) | 79.3 (78.8, 79.8) | 18 (15, 20) | 79.8 (79.1, 80.5) | 22 (18, 26) |
| **Madison** | 71.9 (71.1, 72.4) | 23 (21, 27) | 72.7 (71.9, 73.3) | 27 (24, 30) | 78.6 (77.9, 79.2) | 21 (18, 23) | 78.7 (77.9, 79.3) | 27 (25, 30) |
| **Maries** | 73.5 (72.8, 74.1) | 16 (13, 19) | 74.3 (73.5, 75) | 19 (16, 23) | 79.4 (78.8, 80.1) | 17 (14, 20) | 79.8 (79, 80.6) | 22 (18, 26) |
| **Marion** | 73.6 (72.9, 74.1) | 15 (13, 19) | 74 (73.3, 74.5) | 21 (18, 24) | 79.9 (79.3, 80.4) | 15 (12, 18) | 79.2 (78.6, 79.8) | 25 (22, 28) |
| **McDonald** | 71.8 (71, 72.3) | 24 (22, 27) | 71.9 (71.1, 72.4) | 30 (28, 34) | 78.2 (77.5, 78.7) | 22 (20, 25) | 77.8 (77, 78.4) | 31 (28, 34) |
| **Mercer** | 73.8 (73.2, 74.3) | 14 (12, 17) | 74.8 (74.1, 75.3) | 17 (14, 20) | 80.5 (79.8, 81) | 11 (8, 15) | 80.2 (79.4, 80.7) | 20 (17, 24) |
| **Miller** | 72.5 (71.8, 73) | 21 (18, 24) | 73.6 (72.8, 74.2) | 22 (20, 26) | 79 (78.4, 79.7) | 19 (16, 22) | 78.9 (78.1, 79.6) | 27 (23, 30) |
| **Mississippi** | 69.9 (69.3, 70.3) | 35 (31, 44) | 69.9 (69.4, 70.4) | 42 (37, 50) | 77.2 (76.6, 77.6) | 26 (25, 28) | 76.9 (76.4, 77.4) | 34 (32, 36) |
| **Moniteau** | 74.1 (73.5, 74.6) | 13 (11, 16) | 74.4 (73.7, 74.8) | 19 (17, 22) | 79.3 (78.7, 79.8) | 18 (15, 20) | 79.9 (79.2, 80.5) | 22 (18, 25) |
| **Monroe** | 73.2 (72.4, 73.8) | 17 (15, 21) | 74.4 (73.6, 75) | 19 (16, 22) | 80 (79.3, 80.6) | 14 (10, 18) | 80.1 (79.3, 80.8) | 20 (16, 25) |
| **Montgomery** | 73 (72.3, 73.5) | 18 (16, 22) | 73.7 (72.9, 74.2) | 22 (19, 26) | 79.5 (78.9, 80) | 17 (14, 20) | 79.1 (78.4, 79.7) | 26 (23, 28) |
| **Morgan** | 72.3 (71.5, 72.8) | 22 (19, 25) | 73.5 (72.6, 74.1) | 23 (20, 27) | 78.9 (78.2, 79.5) | 20 (17, 22) | 79.6 (78.8, 80.3) | 23 (19, 27) |
| **New Madrid** | 69.9 (69.3, 70.3) | 35 (31, 44) | 69.9 (69.4, 70.4) | 42 (37, 50) | 77.2 (76.6, 77.6) | 26 (25, 28) | 76.9 (76.4, 77.4) | 34 (32, 36) |
| **Newton** | 73.4 (72.9, 73.9) | 16 (14, 19) | 74.7 (74.1, 75.2) | 17 (15, 20) | 79.7 (79.2, 80.2) | 16 (13, 18) | 80 (79.4, 80.5) | 21 (18, 24) |
| **Nodaway** | 74.9 (74.3, 75.4) | 9 (7, 12) | 76.2 (75.5, 76.7) | 10 (9, 13) | 80.5 (79.8, 81) | 11 (8, 15) | 80.7 (79.9, 81.2) | 17 (14, 21) |
| **Oregon** | 72 (71.3, 72.6) | 23 (20, 26) | 71.9 (71.1, 72.5) | 30 (28, 34) | 79 (78.3, 79.6) | 19 (16, 22) | 78.7 (77.9, 79.4) | 27 (24, 30) |
| **Osage** | 73.5 (72.8, 74.1) | 16 (13, 19) | 74.3 (73.5, 75) | 19 (16, 23) | 79.4 (78.8, 80.1) | 17 (14, 20) | 79.8 (79, 80.6) | 22 (18, 26) |
| **Ozark** | 72.8 (71.9, 73.3) | 20 (17, 23) | 72.5 (71.7, 73.1) | 28 (25, 31) | 79.3 (78.6, 79.8) | 18 (15, 21) | 79.3 (78.4, 79.9) | 25 (21, 28) |
| **Pemiscot** | 68.3 (67.6, 68.8) | 51 (47, 51) | 68.5 (67.7, 69) | 51 (51, 51) | 75.8 (75.3, 76.2) | 32 (30, 36) | 76.5 (75.8, 77) | 36 (34, 39) |
| **Perry** | 73.7 (72.9, 74.2) | 15 (12, 19) | 74.8 (73.9, 75.4) | 17 (14, 21) | 79.1 (78.4, 79.6) | 19 (16, 22) | 80 (79.2, 80.8) | 21 (17, 25) |
| **Pettis** | 73.1 (72.5, 73.6) | 18 (15, 21) | 74.7 (74, 75.3) | 17 (14, 20) | 79.5 (78.9, 80) | 17 (14, 19) | 79.7 (79, 80.3) | 23 (19, 26) |
| **Phelps** | 73.4 (72.9, 73.8) | 16 (14, 19) | 74.4 (73.9, 74.9) | 19 (16, 21) | 79 (78.5, 79.5) | 19 (17, 21) | 79.2 (78.6, 79.7) | 25 (23, 28) |
| **Pike** | 72.9 (72.2, 73.4) | 19 (16, 22) | 73.8 (73.1, 74.4) | 21 (19, 25) | 79.5 (78.8, 80.1) | 17 (13, 20) | 78.8 (78, 79.4) | 27 (24, 30) |
| **Platte** | 76.5 (76, 76.9) | 2 (1, 4) | 76.8 (76.3, 77.3) | 8 (7, 10) | 80.4 (79.8, 80.8) | 12 (9, 15) | 81.5 (80.9, 82) | 13 (10, 16) |
| **Polk** | 72.6 (72, 73.1) | 20 (18, 23) | 73.7 (73, 74.3) | 22 (19, 25) | 79.7 (79.1, 80.2) | 16 (13, 19) | 79 (78.4, 79.7) | 26 (23, 29) |
| **Pulaski** | 73.6 (73, 74.1) | 16 (13, 18) | 74.6 (74, 75.2) | 18 (15, 21) | 78.9 (78.3, 79.5) | 20 (17, 22) | 79.5 (78.8, 80.2) | 24 (20, 27) |
| **Putnam** | 73.8 (73.2, 74.3) | 14 (12, 17) | 74.8 (74.1, 75.3) | 17 (14, 20) | 80.5 (79.8, 81) | 11 (8, 15) | 80.2 (79.4, 80.7) | 20 (17, 24) |
| **Ralls** | 73.2 (72.4, 73.8) | 17 (15, 21) | 74.4 (73.6, 75) | 19 (16, 22) | 80 (79.3, 80.6) | 14 (10, 18) | 80.1 (79.3, 80.8) | 20 (16, 25) |
| **Randolph** | 72.7 (72.1, 73.2) | 20 (17, 22) | 73.7 (73, 74.3) | 22 (19, 25) | 78.8 (78.2, 79.3) | 20 (18, 22) | 79.9 (79.1, 80.6) | 22 (18, 26) |
| **Ray** | 73.5 (72.8, 74) | 16 (13, 19) | 74.8 (73.9, 75.4) | 17 (14, 21) | 79.4 (78.7, 80) | 17 (14, 20) | 79.6 (78.9, 80.3) | 23 (19, 27) |
| **Reynolds** | 70.3 (69.5, 70.8) | 31 (28, 42) | 70.4 (69.7, 71.1) | 37 (34, 46) | 77.2 (76.6, 77.7) | 26 (24, 28) | 77.3 (76.6, 77.9) | 32 (30, 35) |
| **Ripley** | 70.9 (70.4, 71.3) | 28 (26, 31) | 70.7 (70.1, 71.2) | 36 (33, 40) | 77.7 (77.2, 78.1) | 24 (23, 26) | 77.8 (77.2, 78.2) | 31 (29, 33) |
| **Saline** | 73 (72.3, 73.4) | 19 (16, 22) | 74 (73.3, 74.5) | 21 (18, 24) | 79.1 (78.5, 79.6) | 19 (16, 21) | 78.6 (77.9, 79.1) | 28 (26, 30) |
| **Schuyler** | 73.8 (73.2, 74.3) | 14 (12, 17) | 74.8 (74.1, 75.3) | 17 (14, 20) | 80.5 (79.8, 81) | 11 (8, 15) | 80.2 (79.4, 80.7) | 20 (17, 24) |
| **Scotland** | 73.2 (72.4, 73.7) | 17 (15, 21) | 74 (73.3, 74.6) | 20 (18, 24) | 79.3 (78.7, 79.9) | 18 (15, 21) | 80.2 (79.4, 81) | 20 (16, 24) |
| **Scott** | 71.9 (71.2, 72.3) | 23 (22, 26) | 73 (72.3, 73.5) | 25 (23, 29) | 78.3 (77.8, 78.7) | 22 (20, 24) | 79 (78.4, 79.5) | 26 (24, 29) |
| **Shannon** | 72 (71.3, 72.6) | 23 (20, 26) | 71.9 (71.1, 72.5) | 30 (28, 34) | 79 (78.3, 79.6) | 19 (16, 22) | 78.7 (77.9, 79.4) | 27 (24, 30) |
| **Shelby** | 73.5 (72.9, 74) | 16 (13, 19) | 73.8 (73.2, 74.3) | 21 (19, 25) | 79.3 (78.8, 79.8) | 18 (15, 20) | 79.8 (79.1, 80.5) | 22 (18, 26) |
| **St. Charles** | 75.9 (75.6, 76.2) | 5 (4, 6) | 77.5 (77.2, 77.8) | 6 (5, 7) | 80.3 (79.9, 80.6) | 12 (11, 15) | 81.1 (80.8, 81.4) | 15 (13, 16) |
| **St. Clair** | 72.5 (71.7, 73) | 21 (18, 24) | 72.8 (72, 73.4) | 26 (23, 30) | 79.5 (78.8, 80.1) | 17 (14, 20) | 79 (78.3, 79.7) | 26 (23, 29) |
| **St. Francois** | 71.8 (71.3, 72.3) | 23 (22, 26) | 72.1 (71.5, 72.5) | 29 (28, 32) | 77.7 (77.1, 78.1) | 24 (23, 26) | 77.6 (77.1, 78.1) | 31 (30, 33) |
| **St. Louis City** | 67.1 (66.9, 67.3) | 51 (51, 51) | 69.6 (69.3, 69.7) | 47 (44, 51) | 75.2 (75, 75.4) | 36 (35, 38) | 77.7 (77.5, 77.9) | 31 (30, 32) |
| **St. Louis Cnty** | 74.9 (74.8, 75) | 9 (9, 10) | 76.1 (76, 76.2) | 11 (10, 12) | 79.8 (79.7, 80) | 15 (14, 16) | 80.8 (80.7, 81) | 16 (15, 17) |
| **Ste. Genevieve** | 73.9 (73.1, 74.5) | 14 (11, 18) | 74.8 (74, 75.5) | 17 (14, 21) | 79.7 (78.9, 80.3) | 16 (12, 19) | 80.2 (79.2, 81) | 20 (15, 25) |
| **Stoddard** | 71.5 (70.9, 72) | 25 (23, 28) | 71.8 (71.2, 72.4) | 31 (28, 33) | 78.5 (77.9, 79) | 21 (19, 23) | 78.2 (77.6, 78.8) | 29 (27, 32) |
| **Stone** | 74.9 (74.2, 75.4) | 9 (7, 13) | 75.1 (74.3, 75.7) | 15 (13, 19) | 80.4 (79.7, 80.9) | 12 (9, 16) | 81.2 (80.4, 81.8) | 14 (11, 19) |
| **Sullivan** | 73 (72.2, 73.5) | 18 (16, 22) | 73.9 (73.1, 74.5) | 21 (18, 25) | 79.5 (78.9, 80.1) | 17 (14, 20) | 80 (79.2, 80.7) | 21 (17, 25) |
| **Taney** | 73.5 (72.8, 74) | 16 (14, 19) | 74.5 (73.9, 75.1) | 18 (15, 21) | 80.1 (79.5, 80.6) | 14 (10, 17) | 79.9 (79.3, 80.5) | 22 (18, 25) |
| **Texas** | 72.5 (71.7, 73) | 21 (18, 24) | 72 (71.2, 72.5) | 30 (28, 33) | 79.4 (78.7, 79.9) | 17 (14, 20) | 78.5 (77.8, 79.1) | 28 (26, 31) |
| **Vernon** | 73.1 (72.3, 73.6) | 18 (15, 22) | 73.2 (72.3, 73.8) | 24 (21, 29) | 79.3 (78.6, 79.9) | 18 (15, 21) | 78.6 (77.8, 79.2) | 28 (25, 31) |
| **Warren** | 74 (73.3, 74.5) | 14 (11, 17) | 75.3 (74.5, 76) | 14 (12, 18) | 79.8 (79.2, 80.4) | 15 (12, 18) | 80.2 (79.5, 80.9) | 20 (16, 24) |
| **Washington** | 70.6 (69.9, 71.2) | 29 (26, 35) | 70.4 (69.7, 71) | 37 (34, 46) | 77.2 (76.6, 77.8) | 26 (24, 28) | 77.3 (76.5, 77.9) | 33 (30, 36) |
| **Wayne** | 71.5 (70.9, 72) | 25 (23, 28) | 71.8 (71.2, 72.4) | 31 (28, 33) | 78.5 (77.9, 79) | 21 (19, 23) | 78.2 (77.6, 78.8) | 29 (27, 32) |
| **Webster** | 73.4 (72.7, 73.9) | 16 (14, 20) | 73.5 (72.8, 74) | 23 (20, 26) | 78.6 (78, 79.1) | 21 (19, 23) | 79.7 (78.9, 80.4) | 23 (19, 27) |
| **Worth** | 73.3 (72.6, 73.8) | 17 (14, 20) | 74.9 (74.1, 75.5) | 16 (13, 20) | 79.8 (79.2, 80.3) | 15 (12, 18) | 80.2 (79.4, 80.9) | 20 (16, 24) |
| **Wright** | 71.8 (71.1, 72.4) | 23 (21, 27) | 72.1 (71.3, 72.8) | 29 (26, 33) | 79.1 (78.3, 79.7) | 19 (16, 22) | 78.8 (77.9, 79.5) | 27 (24, 30) |
| **MONTANA** |  |  |  |  |  |  |  |  |
| **Beaverhead** | 74.2 (73.5, 74.7) | 13 (10, 16) | 76.6 (75.8, 77.3) | 9 (7, 12) | 79.7 (79, 80.3) | 16 (12, 19) | 79.8 (79, 80.5) | 22 (18, 26) |
| **Big Horn** | 73.3 (72.6, 73.7) | 17 (15, 21) | 74.6 (73.9, 75.1) | 18 (15, 21) | 80.2 (79.5, 80.8) | 13 (9, 17) | 80.3 (79.5, 81) | 19 (15, 24) |
| **Blaine** | 73.1 (72.5, 73.5) | 18 (16, 21) | 74.4 (73.8, 74.9) | 19 (16, 22) | 79.8 (79.2, 80.2) | 15 (13, 18) | 80.2 (79.6, 80.8) | 20 (16, 23) |
| **Broadwater** | 74.8 (74, 75.3) | 10 (7, 13) | 75.9 (75.1, 76.5) | 12 (9, 15) | 80.5 (79.7, 81.2) | 11 (7, 16) | 80.9 (80, 81.7) | 16 (12, 21) |
| **Carbon** | 73.3 (72.6, 73.7) | 17 (15, 21) | 74.6 (73.9, 75.1) | 18 (15, 21) | 80.2 (79.5, 80.8) | 13 (9, 17) | 80.3 (79.5, 81) | 19 (15, 24) |
| **Carter** | 73.8 (73, 74.3) | 15 (12, 18) | 75 (74.1, 75.6) | 16 (13, 20) | 79.8 (79.1, 80.4) | 15 (12, 19) | 80.7 (79.8, 81.5) | 17 (13, 22) |
| **Cascade** | 74.5 (74.1, 74.9) | 11 (9, 13) | 75.6 (75.1, 76.1) | 13 (11, 15) | 80 (79.5, 80.4) | 14 (12, 17) | 81.2 (80.6, 81.7) | 14 (12, 18) |
| **Chouteau** | 73.1 (72.5, 73.5) | 18 (16, 21) | 74.4 (73.8, 74.9) | 19 (16, 22) | 79.8 (79.2, 80.2) | 15 (13, 18) | 80.2 (79.6, 80.8) | 20 (16, 23) |
| **Custer** | 73.8 (73, 74.3) | 15 (12, 18) | 75 (74.1, 75.6) | 16 (13, 20) | 79.8 (79.1, 80.4) | 15 (12, 19) | 80.7 (79.8, 81.5) | 17 (13, 22) |
| **Daniels** | 74.3 (73.6, 74.8) | 12 (10, 16) | 73.6 (72.8, 74.1) | 22 (20, 27) | 80.4 (79.7, 81) | 12 (8, 16) | 79.5 (78.7, 80.1) | 24 (20, 27) |
| **Dawson** | 73.9 (73.2, 74.5) | 14 (11, 17) | 75.3 (74.5, 75.9) | 15 (12, 18) | 80.6 (80, 81.3) | 10 (6, 14) | 80.5 (79.7, 81.3) | 18 (14, 23) |
| **Deer Lodge** | 74.2 (73.5, 74.7) | 13 (10, 16) | 76.6 (75.8, 77.3) | 9 (7, 12) | 79.7 (79, 80.3) | 16 (12, 19) | 79.8 (79, 80.5) | 22 (18, 26) |
| **Fallon** | 74.5 (73.8, 75.1) | 11 (9, 15) | 75.7 (74.9, 76.4) | 12 (10, 16) | 80.5 (79.8, 81.1) | 11 (8, 15) | 81 (80.2, 81.7) | 15 (12, 20) |
| **Fergus** | 73.9 (73.2, 74.5) | 14 (11, 17) | 75.3 (74.4, 75.9) | 15 (12, 19) | 80.1 (79.4, 80.8) | 13 (10, 17) | 80.8 (79.9, 81.5) | 16 (13, 21) |
| **Flathead** | 74.8 (74.3, 75.2) | 10 (8, 12) | 75.5 (75, 75.8) | 14 (12, 16) | 80.6 (80.1, 81.1) | 10 (8, 13) | 80.5 (80, 81) | 18 (15, 21) |
| **Gallatin** | 76.5 (76, 76.9) | 2 (1, 4) | 78.3 (77.7, 78.8) | 3 (0, 5) | 81.2 (80.6, 81.7) | 7 (5, 10) | 82.4 (81.8, 83) | 8 (6, 11) |
| **Garfield** | 74.5 (73.8, 75.1) | 11 (9, 15) | 75.7 (74.9, 76.4) | 12 (10, 16) | 80.5 (79.8, 81.1) | 11 (8, 15) | 81 (80.2, 81.7) | 15 (12, 20) |
| **Glacier** | 73.1 (72.5, 73.5) | 18 (16, 21) | 74.4 (73.8, 74.9) | 19 (16, 22) | 79.8 (79.2, 80.2) | 15 (13, 18) | 80.2 (79.6, 80.8) | 20 (16, 23) |
| **Golden Valley** | 74.2 (73.4, 74.7) | 13 (10, 16) | 75.8 (75.1, 76.4) | 12 (10, 16) | 80 (79.3, 80.6) | 14 (10, 18) | 80.9 (80.1, 81.6) | 16 (12, 20) |
| **Granite** | 74.8 (74.3, 75.2) | 10 (8, 12) | 75.5 (75, 75.8) | 14 (12, 16) | 80.6 (80.1, 81.1) | 10 (8, 13) | 80.5 (80, 81) | 18 (15, 21) |
| **Hill** | 74.2 (73.5, 74.7) | 13 (10, 16) | 74.6 (73.9, 75.2) | 18 (15, 21) | 79.9 (79.3, 80.5) | 15 (11, 18) | 80.7 (80, 81.4) | 17 (13, 21) |
| **Jefferson** | 74.8 (74, 75.3) | 10 (7, 13) | 75.9 (75.1, 76.5) | 12 (9, 15) | 80.5 (79.7, 81.2) | 11 (7, 16) | 80.9 (80, 81.7) | 16 (12, 21) |
| **Judith Basin** | 74.5 (74.1, 74.9) | 11 (9, 13) | 75.6 (75.1, 76.1) | 13 (11, 15) | 80 (79.5, 80.4) | 14 (12, 17) | 81.2 (80.6, 81.7) | 14 (12, 18) |
| **Lake** | 74.1 (73.4, 74.6) | 13 (11, 16) | 75.9 (75.2, 76.5) | 12 (10, 15) | 79.7 (79.1, 80.3) | 16 (12, 19) | 80.6 (79.8, 81.4) | 17 (13, 22) |
| **Lewis And Clark** | 75.2 (74.6, 75.6) | 8 (6, 11) | 76.7 (76.1, 77.2) | 9 (7, 11) | 80.4 (79.8, 80.9) | 12 (9, 15) | 80.5 (79.9, 81) | 18 (15, 21) |
| **Liberty** | 74.2 (73.5, 74.7) | 13 (10, 16) | 74.6 (73.9, 75.2) | 18 (15, 21) | 79.9 (79.3, 80.5) | 15 (11, 18) | 80.7 (80, 81.4) | 17 (13, 21) |
| **Lincoln** | 73.5 (72.8, 73.9) | 16 (14, 19) | 74.7 (74, 75.2) | 17 (15, 21) | 79.2 (78.6, 79.8) | 18 (15, 21) | 79.8 (79.1, 80.4) | 22 (19, 26) |
| **Madison** | 73.5 (72.9, 73.9) | 16 (14, 19) | 74.6 (74, 75.1) | 18 (15, 21) | 79.1 (78.6, 79.6) | 19 (16, 21) | 79.8 (79.2, 80.3) | 22 (19, 25) |
| **McCone** | 74.5 (73.8, 75.1) | 11 (9, 15) | 75.7 (74.9, 76.4) | 12 (10, 16) | 80.5 (79.8, 81.1) | 11 (8, 15) | 81 (80.2, 81.7) | 15 (12, 20) |
| **Meagher** | 74.8 (74, 75.3) | 10 (7, 13) | 75.9 (75.1, 76.5) | 12 (9, 15) | 80.5 (79.7, 81.2) | 11 (7, 16) | 80.9 (80, 81.7) | 16 (12, 21) |
| **Mineral** | 75.7 (75.2, 76.1) | 6 (4, 8) | 76.7 (76.3, 77.1) | 9 (7, 10) | 81.1 (80.6, 81.5) | 8 (6, 11) | 81 (80.5, 81.4) | 15 (13, 18) |
| **Missoula** | 75.7 (75.2, 76.1) | 6 (4, 8) | 76.7 (76.3, 77.1) | 9 (7, 10) | 81.1 (80.6, 81.5) | 8 (6, 11) | 81 (80.5, 81.4) | 15 (13, 18) |
| **Musselshell** | 74.2 (73.4, 74.7) | 13 (10, 16) | 75.8 (75.1, 76.4) | 12 (10, 16) | 80 (79.3, 80.6) | 14 (10, 18) | 80.9 (80.1, 81.6) | 16 (12, 20) |
| **Park** | 76.5 (76, 76.9) | 2 (1, 4) | 78.3 (77.7, 78.8) | 3 (0, 5) | 81.2 (80.6, 81.7) | 7 (5, 10) | 82.4 (81.8, 83) | 8 (6, 11) |
| **Petroleum** | 75.3 (74.8, 75.6) | 8 (6, 10) | 76.1 (75.6, 76.4) | 11 (10, 13) | 80.7 (80.2, 81.1) | 10 (8, 13) | 81 (80.5, 81.4) | 15 (13, 18) |
| **Phillips** | 73.9 (73.2, 74.5) | 14 (11, 17) | 75.3 (74.4, 75.9) | 15 (12, 19) | 80.1 (79.4, 80.8) | 13 (10, 17) | 80.8 (79.9, 81.5) | 16 (13, 21) |
| **Pondera** | 73.1 (72.5, 73.5) | 18 (16, 21) | 74.4 (73.8, 74.9) | 19 (16, 22) | 79.8 (79.2, 80.2) | 15 (13, 18) | 80.2 (79.6, 80.8) | 20 (16, 23) |
| **Powder River** | 73.8 (73, 74.3) | 15 (12, 18) | 75 (74.1, 75.6) | 16 (13, 20) | 79.8 (79.1, 80.4) | 15 (12, 19) | 80.7 (79.8, 81.5) | 17 (13, 22) |
| **Powell** | 74.8 (74.3, 75.2) | 10 (8, 12) | 75.5 (75, 75.8) | 14 (12, 16) | 80.6 (80.1, 81.1) | 10 (8, 13) | 80.5 (80, 81) | 18 (15, 21) |
| **Prairie** | 74.5 (73.8, 75.1) | 11 (9, 15) | 75.7 (74.9, 76.4) | 12 (10, 16) | 80.5 (79.8, 81.1) | 11 (8, 15) | 81 (80.2, 81.7) | 15 (12, 20) |
| **Ravalli** | 75.8 (75.1, 76.3) | 5 (3, 8) | 77.1 (76.5, 77.7) | 7 (5, 10) | 81 (80.3, 81.6) | 8 (5, 12) | 81.3 (80.6, 81.9) | 14 (11, 18) |
| **Richland** | 73.9 (73.2, 74.5) | 14 (11, 17) | 75.3 (74.5, 75.9) | 15 (12, 18) | 80.6 (80, 81.3) | 10 (6, 14) | 80.5 (79.7, 81.3) | 18 (14, 23) |
| **Roosevelt** | 74.3 (73.6, 74.8) | 12 (10, 16) | 73.6 (72.8, 74.1) | 22 (20, 27) | 80.4 (79.7, 81) | 12 (8, 16) | 79.5 (78.7, 80.1) | 24 (20, 27) |
| **Rosebud** | 75.3 (74.8, 75.6) | 8 (6, 10) | 76.1 (75.6, 76.4) | 11 (10, 13) | 80.7 (80.2, 81.1) | 10 (8, 13) | 81 (80.5, 81.4) | 15 (13, 18) |
| **Sanders** | 73.5 (72.8, 73.9) | 16 (14, 19) | 74.7 (74, 75.2) | 17 (15, 21) | 79.2 (78.6, 79.8) | 18 (15, 21) | 79.8 (79.1, 80.4) | 22 (19, 26) |
| **Sheridan** | 74.3 (73.6, 74.8) | 12 (10, 16) | 73.6 (72.8, 74.1) | 22 (20, 27) | 80.4 (79.7, 81) | 12 (8, 16) | 79.5 (78.7, 80.1) | 24 (20, 27) |
| **Silver Bow** | 73.5 (72.9, 73.9) | 16 (14, 19) | 74.6 (74, 75.1) | 18 (15, 21) | 79.1 (78.6, 79.6) | 19 (16, 21) | 79.8 (79.2, 80.3) | 22 (19, 25) |
| **Stillwater** | 74.2 (73.4, 74.7) | 13 (10, 16) | 75.8 (75.1, 76.4) | 12 (10, 16) | 80 (79.3, 80.6) | 14 (10, 18) | 80.9 (80.1, 81.6) | 16 (12, 20) |
| **Sweet Grass** | 74.2 (73.4, 74.7) | 13 (10, 16) | 75.8 (75.1, 76.4) | 12 (10, 16) | 80 (79.3, 80.6) | 14 (10, 18) | 80.9 (80.1, 81.6) | 16 (12, 20) |
| **Teton** | 73.1 (72.5, 73.5) | 18 (16, 21) | 74.4 (73.8, 74.9) | 19 (16, 22) | 79.8 (79.2, 80.2) | 15 (13, 18) | 80.2 (79.6, 80.8) | 20 (16, 23) |
| **Toole** | 74.2 (73.5, 74.7) | 13 (10, 16) | 74.6 (73.9, 75.2) | 18 (15, 21) | 79.9 (79.3, 80.5) | 15 (11, 18) | 80.7 (80, 81.4) | 17 (13, 21) |
| **Treasure** | 75.3 (74.8, 75.6) | 8 (6, 10) | 76.1 (75.6, 76.4) | 11 (10, 13) | 80.7 (80.2, 81.1) | 10 (8, 13) | 81 (80.5, 81.4) | 15 (13, 18) |
| **Valley** | 74.5 (73.8, 75.1) | 11 (9, 15) | 75.7 (74.9, 76.4) | 12 (10, 16) | 80.5 (79.8, 81.1) | 11 (8, 15) | 81 (80.2, 81.7) | 15 (12, 20) |
| **Wheatland** | 74.2 (73.4, 74.7) | 13 (10, 16) | 75.8 (75.1, 76.4) | 12 (10, 16) | 80 (79.3, 80.6) | 14 (10, 18) | 80.9 (80.1, 81.6) | 16 (12, 20) |
| **Wibaux** | 74.5 (73.8, 75.1) | 11 (9, 15) | 75.7 (74.9, 76.4) | 12 (10, 16) | 80.5 (79.8, 81.1) | 11 (8, 15) | 81 (80.2, 81.7) | 15 (12, 20) |
| **Yellowstone** | 75.3 (74.8, 75.6) | 8 (6, 10) | 76.1 (75.6, 76.4) | 11 (10, 13) | 80.7 (80.2, 81.1) | 10 (8, 13) | 81 (80.5, 81.4) | 15 (13, 18) |
| **Yellowst. Nat.** | 76.5 (76, 76.9) | 2 (1, 4) | 78.3 (77.7, 78.8) | 3 (0, 5) | 81.2 (80.6, 81.7) | 7 (5, 10) | 82.4 (81.8, 83) | 8 (6, 11) |
| **NEBRASKA** |  |  |  |  |  |  |  |  |
| **Adams** | 74.8 (74.2, 75.3) | 10 (7, 13) | 76.8 (76.1, 77.3) | 8 (7, 11) | 81.3 (80.7, 81.9) | 7 (4, 10) | 81.8 (81.1, 82.4) | 11 (9, 15) |
| **Antelope** | 75.2 (74.5, 75.8) | 8 (5, 11) | 75.3 (74.5, 75.9) | 15 (12, 18) | 80.5 (79.9, 81.1) | 11 (8, 15) | 81.4 (80.7, 82.1) | 13 (10, 17) |
| **Arthur** | 74.6 (73.8, 75.1) | 11 (8, 14) | 76.1 (75.3, 76.8) | 11 (8, 14) | 80.4 (79.7, 81.1) | 11 (8, 16) | 80.2 (79.4, 81) | 20 (15, 24) |
| **Banner** | 74.5 (73.8, 75) | 11 (9, 15) | 75.8 (75, 76.4) | 12 (10, 16) | 80.4 (79.7, 81) | 12 (8, 16) | 80.7 (80, 81.4) | 17 (13, 21) |
| **Blaine** | 75 (74.3, 75.6) | 9 (6, 12) | 75.6 (75, 76.2) | 13 (11, 16) | 80.6 (79.9, 81.2) | 11 (7, 15) | 81.2 (80.4, 81.9) | 14 (11, 19) |
| **Boone** | 74.9 (74.2, 75.4) | 9 (7, 13) | 75.6 (74.9, 76.2) | 13 (11, 16) | 80.5 (79.8, 81.1) | 11 (8, 15) | 80.5 (79.7, 81.3) | 18 (14, 22) |
| **Box Butte** | 74.6 (74, 75.2) | 11 (8, 14) | 75.4 (74.7, 76) | 14 (11, 17) | 80.6 (79.9, 81.1) | 11 (8, 15) | 80.3 (79.6, 80.9) | 19 (16, 23) |
| **Boyd** | 75 (74.2, 75.5) | 9 (7, 13) | 75.6 (74.8, 76.2) | 13 (10, 17) | 81 (80.3, 81.6) | 8 (5, 13) | 80.9 (80.1, 81.6) | 16 (12, 20) |
| **Brown** | 75 (74.3, 75.6) | 9 (6, 12) | 75.6 (75, 76.2) | 13 (11, 16) | 80.6 (79.9, 81.2) | 11 (7, 15) | 81.2 (80.4, 81.9) | 14 (11, 19) |
| **Buffalo** | 75.6 (75.1, 76.2) | 6 (4, 9) | 76.7 (76.1, 77.3) | 9 (7, 11) | 80.6 (80, 81.2) | 10 (7, 14) | 81.7 (80.9, 82.3) | 12 (9, 16) |
| **Burt** | 74 (73.3, 74.5) | 13 (11, 17) | 74.2 (73.4, 74.7) | 20 (18, 23) | 79.9 (79.3, 80.5) | 14 (11, 18) | 79.8 (79.1, 80.4) | 22 (18, 26) |
| **Butler** | 75 (74.2, 75.5) | 9 (6, 12) | 75.5 (74.7, 76.1) | 14 (11, 18) | 80.6 (79.9, 81.2) | 10 (7, 15) | 81.1 (80.2, 81.8) | 15 (11, 20) |
| **Cass** | 75.4 (74.7, 76) | 7 (4, 10) | 76.1 (75.4, 76.7) | 11 (9, 14) | 80.9 (80.2, 81.5) | 9 (6, 13) | 81.2 (80.4, 81.8) | 14 (11, 19) |
| **Cedar** | 75.6 (74.9, 76.2) | 6 (4, 9) | 76.5 (75.8, 77.1) | 9 (7, 12) | 81.2 (80.5, 81.9) | 7 (4, 11) | 82 (81.1, 82.8) | 10 (7, 15) |
| **Chase** | 75.2 (74.6, 75.7) | 8 (5, 11) | 76.1 (75.4, 76.6) | 11 (9, 14) | 80.6 (80, 81.1) | 10 (8, 14) | 81.4 (80.7, 82) | 13 (10, 17) |
| **Cherry** | 75 (74.3, 75.6) | 9 (6, 12) | 75.6 (75, 76.2) | 13 (11, 16) | 80.6 (79.9, 81.2) | 11 (7, 15) | 81.2 (80.4, 81.9) | 14 (11, 19) |
| **Cheyenne** | 74.5 (73.8, 75) | 11 (9, 15) | 75.8 (75, 76.4) | 12 (10, 16) | 80.4 (79.7, 81) | 12 (8, 16) | 80.7 (80, 81.4) | 17 (13, 21) |
| **Clay** | 74.8 (74.1, 75.3) | 10 (7, 13) | 75.9 (75.3, 76.4) | 12 (10, 15) | 80.7 (80.1, 81.3) | 10 (7, 13) | 80.7 (80.1, 81.3) | 17 (14, 21) |
| **Colfax** | 75 (74.2, 75.5) | 9 (6, 12) | 75.5 (74.7, 76.1) | 14 (11, 18) | 80.6 (79.9, 81.2) | 10 (7, 15) | 81.1 (80.2, 81.8) | 15 (11, 20) |
| **Cuming** | 75.5 (74.7, 76.1) | 7 (4, 10) | 75.7 (75, 76.4) | 12 (10, 16) | 80.6 (79.9, 81.2) | 11 (7, 15) | 81.6 (80.7, 82.4) | 12 (8, 17) |
| **Custer** | 75 (74.3, 75.6) | 9 (6, 12) | 75.6 (75, 76.2) | 13 (11, 16) | 80.6 (79.9, 81.2) | 11 (7, 15) | 81.2 (80.4, 81.9) | 14 (11, 19) |
| **Dakota** | 73.7 (73, 74.2) | 15 (12, 18) | 74.8 (74, 75.4) | 17 (14, 20) | 79.1 (78.5, 79.7) | 19 (16, 21) | 79.6 (78.8, 80.2) | 23 (20, 27) |
| **Dawes** | 74.6 (74, 75.2) | 11 (8, 14) | 75.4 (74.7, 76) | 14 (11, 17) | 80.6 (79.9, 81.1) | 11 (8, 15) | 80.3 (79.6, 80.9) | 19 (16, 23) |
| **Dawson** | 74 (73.3, 74.5) | 14 (11, 17) | 75.6 (75, 76.2) | 13 (11, 16) | 80.3 (79.6, 80.8) | 12 (9, 16) | 80.9 (80.2, 81.5) | 16 (13, 20) |
| **Deuel** | 75.2 (74.6, 75.7) | 8 (5, 11) | 76.1 (75.4, 76.6) | 11 (9, 14) | 80.6 (80, 81.1) | 10 (8, 14) | 81.4 (80.7, 82) | 13 (10, 17) |
| **Dixon** | 73.7 (73, 74.2) | 15 (12, 18) | 74.8 (74, 75.4) | 17 (14, 20) | 79.1 (78.5, 79.7) | 19 (16, 21) | 79.6 (78.8, 80.2) | 23 (20, 27) |
| **Dodge** | 75.4 (74.8, 75.9) | 7 (5, 10) | 75.2 (74.6, 75.8) | 15 (12, 18) | 80.4 (79.9, 81) | 11 (8, 15) | 81.5 (80.8, 82.3) | 13 (9, 16) |
| **Douglas** | 74.1 (73.8, 74.3) | 13 (12, 14) | 75.6 (75.4, 75.8) | 13 (12, 14) | 79.5 (79.3, 79.7) | 17 (16, 18) | 80.7 (80.5, 81) | 17 (15, 18) |
| **Dundy** | 75.2 (74.6, 75.7) | 8 (5, 11) | 76.1 (75.4, 76.6) | 11 (9, 14) | 80.6 (80, 81.1) | 10 (8, 14) | 81.4 (80.7, 82) | 13 (10, 17) |
| **Fillmore** | 74.8 (74.1, 75.3) | 10 (7, 13) | 75.9 (75.3, 76.4) | 12 (10, 15) | 80.7 (80.1, 81.3) | 10 (7, 13) | 80.7 (80.1, 81.3) | 17 (14, 21) |
| **Franklin** | 74.8 (74.1, 75.4) | 10 (7, 13) | 75.6 (74.9, 76.2) | 13 (11, 16) | 80.7 (80, 81.2) | 10 (7, 14) | 80.3 (79.6, 80.9) | 19 (16, 23) |
| **Frontier** | 74.9 (74.1, 75.4) | 10 (7, 13) | 76 (75.2, 76.6) | 11 (9, 15) | 80.6 (79.9, 81.2) | 10 (7, 15) | 81 (80.1, 81.6) | 16 (12, 20) |
| **Furnas** | 74.8 (74.1, 75.4) | 10 (7, 13) | 75.6 (74.9, 76.2) | 13 (11, 16) | 80.7 (80, 81.2) | 10 (7, 14) | 80.3 (79.6, 80.9) | 19 (16, 23) |
| **Gage** | 74.3 (73.6, 74.8) | 12 (10, 16) | 76.3 (75.5, 76.9) | 10 (8, 14) | 80.5 (79.8, 81) | 11 (8, 15) | 80.9 (80.2, 81.5) | 16 (13, 20) |
| **Garden** | 74.6 (73.8, 75.1) | 11 (8, 14) | 76.1 (75.3, 76.8) | 11 (8, 14) | 80.4 (79.7, 81.1) | 11 (8, 16) | 80.2 (79.4, 81) | 20 (15, 24) |
| **Garfield** | 74.6 (73.9, 75.1) | 11 (8, 14) | 76.2 (75.4, 76.8) | 10 (8, 14) | 80.8 (80.2, 81.4) | 9 (6, 13) | 80.7 (79.9, 81.4) | 17 (13, 21) |
| **Gosper** | 74 (73.3, 74.5) | 14 (11, 17) | 75.6 (75, 76.2) | 13 (11, 16) | 80.3 (79.6, 80.8) | 12 (9, 16) | 80.9 (80.2, 81.5) | 16 (13, 20) |
| **Grant** | 74.6 (73.8, 75.1) | 11 (8, 14) | 76.1 (75.3, 76.8) | 11 (8, 14) | 80.4 (79.7, 81.1) | 11 (8, 16) | 80.2 (79.4, 81) | 20 (15, 24) |
| **Greeley** | 74.6 (73.9, 75.1) | 11 (8, 14) | 76.2 (75.4, 76.8) | 10 (8, 14) | 80.8 (80.2, 81.4) | 9 (6, 13) | 80.7 (79.9, 81.4) | 17 (13, 21) |
| **Hall** | 74.1 (73.5, 74.5) | 13 (11, 16) | 75.1 (74.5, 75.5) | 16 (14, 18) | 80.6 (80, 81.1) | 10 (8, 14) | 80.8 (80.2, 81.3) | 16 (13, 20) |
| **Hamilton** | 75.3 (74.5, 75.8) | 8 (5, 11) | 77.1 (76.2, 77.7) | 7 (5, 10) | 80.7 (80, 81.3) | 10 (7, 14) | 81.3 (80.5, 82.1) | 14 (10, 18) |
| **Harlan** | 74.8 (74.1, 75.4) | 10 (7, 13) | 75.6 (74.9, 76.2) | 13 (11, 16) | 80.7 (80, 81.2) | 10 (7, 14) | 80.3 (79.6, 80.9) | 19 (16, 23) |
| **Hayes** | 75.2 (74.6, 75.7) | 8 (5, 11) | 76.1 (75.4, 76.6) | 11 (9, 14) | 80.6 (80, 81.1) | 10 (8, 14) | 81.4 (80.7, 82) | 13 (10, 17) |
| **Hitchcock** | 74.9 (74.1, 75.4) | 10 (7, 13) | 76 (75.2, 76.6) | 11 (9, 15) | 80.6 (79.9, 81.2) | 10 (7, 15) | 81 (80.1, 81.6) | 16 (12, 20) |
| **Holt** | 75 (74.2, 75.5) | 9 (7, 13) | 75.6 (74.8, 76.2) | 13 (10, 17) | 81 (80.3, 81.6) | 8 (5, 13) | 80.9 (80.1, 81.6) | 16 (12, 20) |
| **Hooker** | 74.6 (73.8, 75.1) | 11 (8, 14) | 76.1 (75.3, 76.8) | 11 (8, 14) | 80.4 (79.7, 81.1) | 11 (8, 16) | 80.2 (79.4, 81) | 20 (15, 24) |
| **Howard** | 74.6 (73.9, 75.1) | 11 (8, 14) | 76.2 (75.4, 76.8) | 10 (8, 14) | 80.8 (80.2, 81.4) | 9 (6, 13) | 80.7 (79.9, 81.4) | 17 (13, 21) |
| **Jefferson** | 74.8 (74.1, 75.3) | 10 (7, 13) | 75.9 (75.3, 76.4) | 12 (10, 15) | 80.7 (80.1, 81.3) | 10 (7, 13) | 80.7 (80.1, 81.3) | 17 (14, 21) |
| **Johnson** | 73.9 (73.3, 74.4) | 14 (12, 17) | 75.4 (74.7, 76) | 14 (11, 17) | 81 (80.4, 81.6) | 8 (5, 12) | 80.8 (80.2, 81.5) | 16 (13, 20) |
| **Kearney** | 74.8 (74.1, 75.4) | 10 (7, 13) | 75.6 (74.9, 76.2) | 13 (11, 16) | 80.7 (80, 81.2) | 10 (7, 14) | 80.3 (79.6, 80.9) | 19 (16, 23) |
| **Keith** | 74.6 (73.8, 75.1) | 11 (8, 14) | 76.1 (75.3, 76.8) | 11 (8, 14) | 80.4 (79.7, 81.1) | 11 (8, 16) | 80.2 (79.4, 81) | 20 (15, 24) |
| **Keya Paha** | 75 (74.2, 75.5) | 9 (7, 13) | 75.6 (74.8, 76.2) | 13 (10, 17) | 81 (80.3, 81.6) | 8 (5, 13) | 80.9 (80.1, 81.6) | 16 (12, 20) |
| **Kimball** | 74.5 (73.8, 75) | 11 (9, 15) | 75.8 (75, 76.4) | 12 (10, 16) | 80.4 (79.7, 81) | 12 (8, 16) | 80.7 (80, 81.4) | 17 (13, 21) |
| **Knox** | 75.2 (74.5, 75.8) | 8 (5, 11) | 75.3 (74.5, 75.9) | 15 (12, 18) | 80.5 (79.9, 81.1) | 11 (8, 15) | 81.4 (80.7, 82.1) | 13 (10, 17) |
| **Lancaster** | 76.3 (76, 76.6) | 3 (2, 4) | 77.8 (77.5, 78.2) | 5 (3, 6) | 81.3 (81, 81.7) | 7 (5, 8) | 82.3 (82, 82.7) | 9 (7, 11) |
| **Lincoln** | 75.2 (74.6, 75.7) | 8 (5, 11) | 76.1 (75.4, 76.6) | 11 (9, 14) | 80.6 (80, 81.1) | 10 (8, 14) | 81.4 (80.7, 82) | 13 (10, 17) |
| **Logan** | 74.6 (73.8, 75.1) | 11 (8, 14) | 76.1 (75.3, 76.8) | 11 (8, 14) | 80.4 (79.7, 81.1) | 11 (8, 16) | 80.2 (79.4, 81) | 20 (15, 24) |
| **Loup** | 75 (74.3, 75.6) | 9 (6, 12) | 75.6 (75, 76.2) | 13 (11, 16) | 80.6 (79.9, 81.2) | 11 (7, 15) | 81.2 (80.4, 81.9) | 14 (11, 19) |
| **Madison** | 74.7 (74.1, 75.2) | 10 (8, 13) | 75.6 (74.9, 76.1) | 13 (11, 16) | 80.6 (79.9, 81.1) | 11 (8, 14) | 80.8 (80.1, 81.4) | 16 (13, 21) |
| **McPherson** | 74.6 (73.8, 75.1) | 11 (8, 14) | 76.1 (75.3, 76.8) | 11 (8, 14) | 80.4 (79.7, 81.1) | 11 (8, 16) | 80.2 (79.4, 81) | 20 (15, 24) |
| **Merrick** | 74.9 (74.2, 75.4) | 9 (7, 13) | 75.6 (74.9, 76.2) | 13 (11, 16) | 80.5 (79.8, 81.1) | 11 (8, 15) | 80.5 (79.7, 81.3) | 18 (14, 22) |
| **Morrill** | 73.9 (73.3, 74.3) | 14 (12, 17) | 74.4 (73.8, 74.9) | 19 (16, 21) | 79.4 (78.9, 79.8) | 17 (15, 20) | 80.9 (80.2, 81.5) | 16 (13, 20) |
| **Nance** | 74.9 (74.2, 75.4) | 9 (7, 13) | 75.6 (74.9, 76.2) | 13 (11, 16) | 80.5 (79.8, 81.1) | 11 (8, 15) | 80.5 (79.7, 81.3) | 18 (14, 22) |
| **Nemaha** | 73.9 (73.3, 74.4) | 14 (12, 17) | 75.4 (74.7, 76) | 14 (11, 17) | 81 (80.4, 81.6) | 8 (5, 12) | 80.8 (80.2, 81.5) | 16 (13, 20) |
| **Nuckolls** | 74.8 (74.2, 75.3) | 10 (7, 13) | 76.8 (76.1, 77.3) | 8 (7, 11) | 81.3 (80.7, 81.9) | 7 (4, 10) | 81.8 (81.1, 82.4) | 11 (9, 15) |
| **Otoe** | 75.2 (74.4, 75.7) | 8 (5, 12) | 76.6 (75.8, 77.2) | 9 (7, 12) | 80.1 (79.4, 80.6) | 14 (10, 17) | 80.9 (80, 81.5) | 16 (13, 21) |
| **Pawnee** | 73.9 (73.3, 74.4) | 14 (12, 17) | 75.4 (74.7, 76) | 14 (11, 17) | 81 (80.4, 81.6) | 8 (5, 12) | 80.8 (80.2, 81.5) | 16 (13, 20) |
| **Perkins** | 75.2 (74.6, 75.7) | 8 (5, 11) | 76.1 (75.4, 76.6) | 11 (9, 14) | 80.6 (80, 81.1) | 10 (8, 14) | 81.4 (80.7, 82) | 13 (10, 17) |
| **Phelps** | 74 (73.3, 74.5) | 14 (11, 17) | 75.6 (75, 76.2) | 13 (11, 16) | 80.3 (79.6, 80.8) | 12 (9, 16) | 80.9 (80.2, 81.5) | 16 (13, 20) |
| **Pierce** | 75.2 (74.5, 75.8) | 8 (5, 11) | 75.3 (74.5, 75.9) | 15 (12, 18) | 80.5 (79.9, 81.1) | 11 (8, 15) | 81.4 (80.7, 82.1) | 13 (10, 17) |
| **Platte** | 75.2 (74.5, 75.7) | 8 (5, 11) | 77.2 (76.4, 77.8) | 7 (5, 10) | 80.9 (80.2, 81.6) | 9 (5, 13) | 82.1 (81.3, 82.9) | 10 (7, 14) |
| **Polk** | 75.3 (74.5, 75.8) | 8 (5, 11) | 77.1 (76.2, 77.7) | 7 (5, 10) | 80.7 (80, 81.3) | 10 (7, 14) | 81.3 (80.5, 82.1) | 14 (10, 18) |
| **Red Willow** | 74.9 (74.1, 75.4) | 10 (7, 13) | 76 (75.2, 76.6) | 11 (9, 15) | 80.6 (79.9, 81.2) | 10 (7, 15) | 81 (80.1, 81.6) | 16 (12, 20) |
| **Richardson** | 73.9 (73.3, 74.4) | 14 (12, 17) | 75.4 (74.7, 76) | 14 (11, 17) | 81 (80.4, 81.6) | 8 (5, 12) | 80.8 (80.2, 81.5) | 16 (13, 20) |
| **Rock** | 75 (74.2, 75.5) | 9 (7, 13) | 75.6 (74.8, 76.2) | 13 (10, 17) | 81 (80.3, 81.6) | 8 (5, 13) | 80.9 (80.1, 81.6) | 16 (12, 20) |
| **Saline** | 75.1 (74.4, 75.6) | 8 (6, 12) | 76.9 (76.2, 77.4) | 8 (6, 11) | 81 (80.3, 81.6) | 8 (5, 12) | 81.8 (81, 82.5) | 11 (8, 15) |
| **Sarpy** | 76.2 (75.8, 76.6) | 3 (2, 5) | 77.6 (77.1, 78) | 6 (4, 7) | 80.3 (79.7, 80.7) | 13 (10, 15) | 80.9 (80.5, 81.4) | 16 (13, 18) |
| **Saunders** | 75.5 (74.7, 76) | 7 (5, 10) | 76.6 (75.7, 77.2) | 9 (7, 13) | 81 (80.2, 81.5) | 8 (5, 13) | 81.5 (80.6, 82.2) | 13 (9, 18) |
| **Scotts Bluff** | 73.9 (73.3, 74.3) | 14 (12, 17) | 74.4 (73.8, 74.9) | 19 (16, 21) | 79.4 (78.9, 79.8) | 17 (15, 20) | 80.9 (80.2, 81.5) | 16 (13, 20) |
| **Seward** | 75.5 (74.8, 76) | 7 (4, 10) | 76.7 (75.9, 77.4) | 9 (6, 12) | 81 (80.2, 81.7) | 8 (5, 13) | 81.5 (80.6, 82.3) | 13 (9, 18) |
| **Sheridan** | 73.9 (73.3, 74.3) | 14 (12, 17) | 74.4 (73.8, 74.9) | 19 (16, 21) | 79.4 (78.9, 79.8) | 17 (15, 20) | 80.9 (80.2, 81.5) | 16 (13, 20) |
| **Sherman** | 74.6 (73.9, 75.1) | 11 (8, 14) | 76.2 (75.4, 76.8) | 10 (8, 14) | 80.8 (80.2, 81.4) | 9 (6, 13) | 80.7 (79.9, 81.4) | 17 (13, 21) |
| **Sioux** | 74.6 (74, 75.2) | 11 (8, 14) | 75.4 (74.7, 76) | 14 (11, 17) | 80.6 (79.9, 81.1) | 11 (8, 15) | 80.3 (79.6, 80.9) | 19 (16, 23) |
| **Stanton** | 75.5 (74.7, 76.1) | 7 (4, 10) | 75.7 (75, 76.4) | 12 (10, 16) | 80.6 (79.9, 81.2) | 11 (7, 15) | 81.6 (80.7, 82.4) | 12 (8, 17) |
| **Thayer** | 74.8 (74.1, 75.3) | 10 (7, 13) | 75.9 (75.3, 76.4) | 12 (10, 15) | 80.7 (80.1, 81.3) | 10 (7, 13) | 80.7 (80.1, 81.3) | 17 (14, 21) |
| **Thomas** | 75 (74.3, 75.6) | 9 (6, 12) | 75.6 (75, 76.2) | 13 (11, 16) | 80.6 (79.9, 81.2) | 11 (7, 15) | 81.2 (80.4, 81.9) | 14 (11, 19) |
| **Thurston** | 74 (73.3, 74.5) | 13 (11, 17) | 74.2 (73.4, 74.7) | 20 (18, 23) | 79.9 (79.3, 80.5) | 14 (11, 18) | 79.8 (79.1, 80.4) | 22 (18, 26) |
| **Valley** | 74.6 (73.9, 75.1) | 11 (8, 14) | 76.2 (75.4, 76.8) | 10 (8, 14) | 80.8 (80.2, 81.4) | 9 (6, 13) | 80.7 (79.9, 81.4) | 17 (13, 21) |
| **Washington** | 75.8 (75.1, 76.4) | 5 (3, 8) | 77 (76.2, 77.6) | 8 (5, 11) | 80.4 (79.7, 81) | 12 (8, 16) | 81.2 (80.3, 81.8) | 14 (11, 19) |
| **Wayne** | 75.6 (74.9, 76.2) | 6 (4, 9) | 76.5 (75.8, 77.1) | 9 (7, 12) | 81.2 (80.5, 81.9) | 7 (4, 11) | 82 (81.1, 82.8) | 10 (7, 15) |
| **Webster** | 74.8 (74.2, 75.3) | 10 (7, 13) | 76.8 (76.1, 77.3) | 8 (7, 11) | 81.3 (80.7, 81.9) | 7 (4, 10) | 81.8 (81.1, 82.4) | 11 (9, 15) |
| **Wheeler** | 74.6 (73.9, 75.1) | 11 (8, 14) | 76.2 (75.4, 76.8) | 10 (8, 14) | 80.8 (80.2, 81.4) | 9 (6, 13) | 80.7 (79.9, 81.4) | 17 (13, 21) |
| **York** | 75.1 (74.4, 75.6) | 8 (6, 12) | 76.9 (76.2, 77.4) | 8 (6, 11) | 81 (80.3, 81.6) | 8 (5, 12) | 81.8 (81, 82.5) | 11 (8, 15) |
| **NEVADA** |  |  |  |  |  |  |  |  |
| **Carson City** | 73.8 (73.3, 74.2) | 14 (13, 17) | 74 (73.5, 74.4) | 21 (19, 23) | 79.5 (79, 80) | 17 (14, 19) | 79.6 (79.1, 80.1) | 23 (20, 26) |
| **Churchill** | 74.2 (73.5, 74.7) | 13 (10, 16) | 75.1 (74.4, 75.6) | 16 (13, 19) | 79.8 (79.1, 80.4) | 15 (12, 19) | 80.1 (79.4, 80.7) | 20 (17, 24) |
| **Clark** | 73.1 (73, 73.3) | 18 (17, 18) | 74.1 (73.9, 74.2) | 20 (20, 21) | 78.6 (78.5, 78.7) | 21 (20, 21) | 79.7 (79.6, 79.8) | 23 (22, 23) |
| **Douglas** | 77.7 (77.1, 78.3) | -2 (-4, 0) | 79.1 (78.5, 79.7) | -1 (-5, 2) | 81.1 (80.5, 81.7) | 8 (4, 11) | 82.7 (82, 83.3) | 7 (4, 10) |
| **Elko** | 74.3 (73.7, 74.8) | 12 (10, 15) | 75 (74.3, 75.6) | 16 (13, 19) | 80.1 (79.3, 80.7) | 14 (10, 18) | 79.7 (79, 80.3) | 23 (19, 26) |
| **Esmeralda** | 72.8 (72.2, 73.3) | 19 (17, 22) | 72.2 (71.6, 72.6) | 29 (27, 32) | 79.3 (78.7, 79.8) | 18 (15, 20) | 78.8 (78.1, 79.3) | 27 (25, 30) |
| **Eureka** | 73.4 (72.7, 74) | 16 (14, 20) | 74.9 (74.1, 75.5) | 16 (13, 20) | 79.6 (78.9, 80.2) | 16 (13, 20) | 80.3 (79.5, 81.1) | 19 (15, 24) |
| **Humboldt** | 73.5 (72.8, 74.1) | 16 (13, 19) | 74.7 (74, 75.3) | 17 (14, 21) | 79.8 (79.1, 80.5) | 15 (11, 19) | 80.1 (79.2, 80.9) | 21 (16, 25) |
| **Lander** | 73.4 (72.7, 74) | 16 (14, 20) | 74.9 (74.1, 75.5) | 16 (13, 20) | 79.6 (78.9, 80.2) | 16 (13, 20) | 80.3 (79.5, 81.1) | 19 (15, 24) |
| **Lincoln** | 73.1 (73, 73.3) | 18 (17, 18) | 74.1 (73.9, 74.2) | 20 (20, 21) | 78.6 (78.5, 78.7) | 21 (20, 21) | 79.7 (79.6, 79.8) | 23 (22, 23) |
| **Lyon** | 74.3 (73.7, 74.7) | 12 (10, 15) | 74.5 (73.9, 74.9) | 18 (16, 21) | 79.1 (78.5, 79.7) | 19 (16, 21) | 79.9 (79.2, 80.5) | 22 (18, 25) |
| **Mineral** | 72.8 (72.2, 73.3) | 19 (17, 22) | 72.2 (71.6, 72.6) | 29 (27, 32) | 79.3 (78.7, 79.8) | 18 (15, 20) | 78.8 (78.1, 79.3) | 27 (25, 30) |
| **Nye** | 72.8 (72.2, 73.3) | 19 (17, 22) | 72.2 (71.6, 72.6) | 29 (27, 32) | 79.3 (78.7, 79.8) | 18 (15, 20) | 78.8 (78.1, 79.3) | 27 (25, 30) |
| **Pershing** | 73.5 (72.8, 74.1) | 16 (13, 19) | 74.7 (74, 75.3) | 17 (14, 21) | 79.8 (79.1, 80.5) | 15 (11, 19) | 80.1 (79.2, 80.9) | 21 (16, 25) |
| **Storey** | 74.3 (73.7, 74.7) | 12 (10, 15) | 74.5 (73.9, 74.9) | 18 (16, 21) | 79.1 (78.5, 79.7) | 19 (16, 21) | 79.9 (79.2, 80.5) | 22 (18, 25) |
| **Washoe** | 73.9 (73.6, 74.1) | 14 (13, 15) | 75.2 (74.9, 75.4) | 15 (14, 16) | 79.1 (78.8, 79.4) | 19 (17, 20) | 80.3 (80, 80.5) | 19 (18, 21) |
| **White Pine** | 73.4 (72.7, 74) | 16 (14, 20) | 74.9 (74.1, 75.5) | 16 (13, 20) | 79.6 (78.9, 80.2) | 16 (13, 20) | 80.3 (79.5, 81.1) | 19 (15, 24) |
| **NEW HAMPSHIRE** |  |  |  |  |  |  |  |  |
| **Belknap** | 75.4 (74.8, 75.8) | 7 (5, 10) | 76 (75.4, 76.4) | 12 (10, 14) | 80.3 (79.8, 80.8) | 12 (10, 15) | 82 (81.3, 82.5) | 11 (8, 14) |
| **Carroll** | 75.9 (75.3, 76.5) | 5 (3, 7) | 77.1 (76.4, 77.6) | 7 (5, 10) | 80.7 (80.1, 81.3) | 10 (7, 13) | 82.4 (81.6, 83) | 9 (6, 12) |
| **Cheshire** | 75.8 (75.3, 76.2) | 5 (4, 8) | 77.4 (76.9, 77.9) | 6 (4, 8) | 80.6 (80.1, 81.1) | 10 (8, 13) | 81.4 (80.8, 81.9) | 13 (11, 16) |
| **Coos** | 73.7 (73.1, 74.3) | 15 (12, 18) | 74.3 (73.6, 74.8) | 19 (17, 22) | 79.6 (79, 80.1) | 16 (13, 19) | 80.4 (79.7, 81) | 18 (15, 23) |
| **Grafton** | 76.2 (75.8, 76.6) | 3 (2, 5) | 78.8 (78.3, 79.3) | 0 (-2, 3) | 81 (80.5, 81.5) | 8 (6, 11) | 82 (81.5, 82.5) | 10 (8, 13) |
| **Hillsborough** | 76 (75.7, 76.2) | 4 (3, 6) | 77.3 (77, 77.6) | 7 (6, 8) | 80.7 (80.5, 81) | 10 (8, 11) | 81.4 (81.1, 81.7) | 13 (12, 15) |
| **Merrimack** | 75.3 (74.8, 75.6) | 8 (6, 10) | 77.1 (76.7, 77.5) | 7 (6, 9) | 80.9 (80.5, 81.3) | 9 (7, 11) | 81.4 (81, 81.8) | 13 (11, 15) |
| **Rockingham** | 76.4 (76.1, 76.7) | 3 (2, 4) | 77.8 (77.4, 78.1) | 5 (4, 6) | 80.7 (80.4, 81) | 10 (8, 12) | 81.7 (81.3, 82) | 12 (11, 14) |
| **Strafford** | 75.6 (75.2, 75.9) | 6 (5, 8) | 76.5 (76.1, 76.8) | 9 (8, 11) | 80.6 (80.1, 81) | 11 (9, 13) | 81 (80.5, 81.4) | 15 (13, 18) |
| **Sullivan** | 74.7 (74.1, 75.2) | 10 (8, 13) | 75.6 (74.9, 76.1) | 13 (11, 17) | 80.4 (79.8, 80.9) | 12 (9, 15) | 81.2 (80.5, 81.8) | 14 (11, 18) |
| **NEW JERSEY** |  |  |  |  |  |  |  |  |
| **Atlantic** | 72.8 (72.5, 73) | 20 (18, 21) | 73.6 (73.3, 73.8) | 22 (21, 24) | 78.7 (78.4, 78.9) | 20 (20, 21) | 79.8 (79.5, 80) | 22 (21, 24) |
| **Bergen** | 77.5 (77.3, 77.6) | -1 (-2, -1) | 79.3 (79.1, 79.4) | -2 (-3, -1) | 82 (81.8, 82.2) | 3 (3, 4) | 83.5 (83.3, 83.7) | 3 (2, 4) |
| **Burlington** | 75 (74.8, 75.2) | 9 (8, 10) | 76.5 (76.3, 76.7) | 9 (9, 10) | 79.9 (79.7, 80.1) | 14 (13, 16) | 81.3 (81.1, 81.5) | 14 (13, 15) |
| **Camden** | 73 (72.8, 73.2) | 18 (18, 19) | 74 (73.8, 74.2) | 20 (20, 21) | 78.7 (78.5, 78.9) | 20 (20, 21) | 79.7 (79.5, 79.9) | 22 (21, 24) |
| **Cape May** | 73.7 (73.3, 74) | 15 (14, 17) | 74.2 (73.8, 74.6) | 19 (18, 22) | 79.9 (79.6, 80.3) | 14 (13, 16) | 80.2 (79.8, 80.5) | 20 (18, 22) |
| **Cumberland** | 72.1 (71.8, 72.4) | 22 (21, 24) | 72.7 (72.4, 72.9) | 27 (26, 28) | 77.6 (77.3, 77.9) | 24 (23, 26) | 78.6 (78.3, 79) | 28 (26, 29) |
| **Essex** | 71.9 (71.8, 72.1) | 23 (23, 24) | 73.8 (73.6, 73.9) | 22 (21, 22) | 77.7 (77.6, 77.8) | 24 (24, 25) | 79.8 (79.6, 80) | 22 (21, 23) |
| **Gloucester** | 74.1 (73.9, 74.4) | 13 (12, 14) | 74.6 (74.3, 74.8) | 18 (17, 19) | 79.2 (78.9, 79.4) | 18 (17, 20) | 80 (79.7, 80.3) | 21 (19, 23) |
| **Hudson** | 73.8 (73.6, 73.9) | 14 (14, 15) | 75.8 (75.6, 76) | 12 (11, 13) | 79.4 (79.2, 79.5) | 17 (17, 18) | 81.3 (81.1, 81.5) | 14 (13, 15) |
| **Hunterdon** | 77.7 (77.3, 78.1) | -2 (-3, 0) | 79.4 (79, 79.8) | -3 (-6, -1) | 81.2 (80.7, 81.5) | 7 (5, 10) | 82.7 (82.1, 83.1) | 7 (5, 10) |
| **Mercer** | 74.4 (74.2, 74.6) | 12 (11, 12) | 76.2 (76, 76.4) | 10 (10, 11) | 79.5 (79.3, 79.7) | 17 (16, 18) | 81.4 (81.1, 81.6) | 13 (12, 15) |
| **Middlesex** | 75.7 (75.5, 75.8) | 6 (5, 6) | 78 (77.9, 78.2) | 4 (3, 5) | 80.5 (80.3, 80.7) | 11 (10, 12) | 82.4 (82.2, 82.6) | 9 (8, 9) |
| **Monmouth** | 75.5 (75.3, 75.7) | 7 (6, 7) | 77.3 (77.1, 77.5) | 7 (6, 7) | 79.8 (79.7, 80) | 15 (14, 16) | 81.6 (81.4, 81.8) | 12 (11, 13) |
| **Morris** | 77.5 (77.3, 77.7) | -1 (-2, 0) | 79.5 (79.2, 79.7) | -4 (-5, -2) | 81.1 (80.8, 81.3) | 8 (7, 9) | 82.2 (82, 82.5) | 9 (8, 10) |
| **Ocean** | 75 (74.8, 75.2) | 9 (8, 10) | 76 (75.8, 76.2) | 11 (11, 12) | 80.7 (80.5, 80.9) | 10 (9, 11) | 81.8 (81.6, 82.1) | 11 (10, 12) |
| **Passaic** | 74.5 (74.3, 74.7) | 11 (10, 12) | 76.3 (76.1, 76.5) | 10 (10, 11) | 79.6 (79.4, 79.7) | 16 (15, 18) | 81.1 (80.8, 81.3) | 15 (14, 16) |
| **Salem** | 73.1 (72.6, 73.4) | 18 (16, 20) | 73.4 (72.8, 73.7) | 24 (22, 26) | 78.4 (78, 78.8) | 21 (20, 23) | 78.7 (78.2, 79.1) | 27 (26, 29) |
| **Somerset** | 77.3 (77, 77.5) | 0 (-1, 1) | 78.7 (78.5, 79) | 1 (-1, 2) | 81.5 (81.2, 81.8) | 6 (4, 7) | 82.5 (82.2, 82.8) | 8 (7, 9) |
| **Sussex** | 75.7 (75.3, 76.1) | 6 (4, 7) | 77.2 (76.7, 77.5) | 7 (6, 9) | 80.2 (79.8, 80.6) | 13 (10, 15) | 80.6 (80.2, 81) | 18 (15, 20) |
| **Union** | 75 (74.9, 75.2) | 9 (8, 10) | 77.2 (77, 77.4) | 7 (6, 8) | 80.2 (80, 80.4) | 13 (12, 14) | 81.1 (80.9, 81.3) | 15 (14, 16) |
| **Warren** | 75.3 (74.9, 75.6) | 7 (6, 10) | 76.7 (76.3, 77.1) | 9 (7, 10) | 80.3 (79.9, 80.7) | 12 (10, 15) | 80.7 (80.3, 81.1) | 17 (15, 20) |
| **NEW MEXICO** |  |  |  |  |  |  |  |  |
| **Bernalillo** | 74.9 (74.7, 75.2) | 9 (8, 10) | 74.8 (74.6, 75) | 17 (16, 18) | 80.5 (80.3, 80.8) | 11 (10, 12) | 81.1 (80.9, 81.3) | 15 (14, 16) |
| **Catron** | 72.7 (71.8, 73.3) | 20 (17, 24) | 73.3 (72.4, 73.9) | 24 (21, 28) | 80.2 (79.4, 80.8) | 13 (9, 17) | 79.8 (78.9, 80.5) | 22 (18, 27) |
| **Cebola** | 73 (72.3, 73.4) | 18 (16, 21) | 73.4 (72.7, 73.9) | 23 (21, 27) | 79.1 (78.5, 79.6) | 19 (16, 21) | 80.4 (79.6, 80.9) | 19 (16, 23) |
| **Chaves** | 73.2 (72.6, 73.6) | 17 (15, 20) | 73 (72.4, 73.5) | 25 (23, 28) | 79.2 (78.6, 79.6) | 18 (16, 21) | 79.7 (79.1, 80.2) | 23 (20, 26) |
| **Colfax** | 75 (74.2, 75.6) | 9 (6, 13) | 75.4 (74.5, 76.1) | 14 (11, 18) | 80.6 (79.9, 81.3) | 10 (7, 15) | 80.3 (79.5, 80.9) | 19 (16, 24) |
| **Curry** | 73.3 (72.7, 73.7) | 17 (15, 20) | 74.2 (73.5, 74.7) | 20 (18, 23) | 79.4 (78.8, 79.9) | 17 (15, 20) | 79.1 (78.5, 79.6) | 26 (23, 28) |
| **De Baca** | 72.5 (71.7, 73.1) | 21 (18, 24) | 72.9 (72, 73.6) | 26 (22, 30) | 79.3 (78.5, 79.9) | 18 (15, 21) | 79.6 (78.7, 80.3) | 23 (19, 27) |
| **Dona Ana** | 74.9 (74.5, 75.3) | 9 (7, 11) | 76.3 (75.9, 76.6) | 10 (9, 12) | 80.3 (79.9, 80.7) | 12 (10, 15) | 80.9 (80.4, 81.3) | 16 (14, 18) |
| **Eddy** | 73 (72.4, 73.4) | 18 (16, 21) | 73.8 (73.1, 74.3) | 21 (19, 25) | 79.2 (78.6, 79.7) | 18 (16, 21) | 80.1 (79.5, 80.7) | 20 (17, 24) |
| **Grant** | 73.1 (72.4, 73.6) | 18 (15, 21) | 74.7 (73.9, 75.4) | 17 (14, 21) | 79.6 (79, 80.2) | 16 (13, 19) | 80.6 (79.9, 81.3) | 17 (14, 22) |
| **Guadalupe** | 72.5 (71.7, 73.1) | 21 (18, 24) | 72.9 (72, 73.6) | 26 (22, 30) | 79.3 (78.5, 79.9) | 18 (15, 21) | 79.6 (78.7, 80.3) | 23 (19, 27) |
| **Harding** | 75 (74.2, 75.6) | 9 (6, 13) | 75.4 (74.5, 76.1) | 14 (11, 18) | 80.6 (79.9, 81.3) | 10 (7, 15) | 80.3 (79.5, 80.9) | 19 (16, 24) |
| **Hidalgo** | 72.4 (71.7, 72.9) | 21 (19, 24) | 73.2 (72.4, 73.8) | 24 (21, 28) | 78.8 (78.1, 79.4) | 20 (17, 23) | 79.8 (78.9, 80.5) | 22 (18, 26) |
| **Lea** | 72.4 (71.9, 72.8) | 21 (19, 23) | 73.2 (72.7, 73.7) | 24 (22, 27) | 78.4 (77.9, 78.9) | 21 (20, 23) | 78.9 (78.4, 79.5) | 26 (24, 29) |
| **Lincoln** | 74.8 (74.1, 75.4) | 10 (7, 13) | 76.3 (75.3, 76.9) | 10 (8, 14) | 80.6 (79.9, 81.3) | 10 (7, 15) | 81.8 (80.8, 82.5) | 11 (8, 16) |
| **Los Alamos** | 78.6 (77.8, 79.2) | -6 (-9, -2) | 80.1 (79.3, 80.8) | -8 (-13, -2) | 81.8 (81, 82.5) | 4 (1, 8) | 83 (82.1, 83.8) | 6 (2, 10) |
| **Luna** | 72.4 (71.7, 72.9) | 21 (19, 24) | 73.2 (72.4, 73.8) | 24 (21, 28) | 78.8 (78.1, 79.4) | 20 (17, 23) | 79.8 (78.9, 80.5) | 22 (18, 26) |
| **McKinley** | 71.6 (71.1, 71.9) | 25 (23, 27) | 71.5 (70.9, 71.9) | 32 (30, 35) | 79.5 (79, 79.9) | 17 (15, 19) | 79.5 (79, 80) | 24 (21, 26) |
| **Mora** | 74.3 (73.7, 74.8) | 12 (10, 15) | 75.4 (74.7, 76) | 14 (11, 17) | 80.8 (80.2, 81.4) | 9 (6, 13) | 81.8 (81, 82.5) | 11 (8, 15) |
| **Otero** | 74.4 (73.9, 74.8) | 12 (10, 14) | 75.6 (75, 76.1) | 13 (11, 16) | 79.7 (79.1, 80.2) | 16 (13, 19) | 79.7 (79.1, 80.2) | 23 (20, 26) |
| **Quay** | 72.5 (71.7, 73.1) | 21 (18, 24) | 72.9 (72, 73.6) | 26 (22, 30) | 79.3 (78.5, 79.9) | 18 (15, 21) | 79.6 (78.7, 80.3) | 23 (19, 27) |
| **Rio Arriba** | 70.7 (69.9, 71.3) | 29 (26, 35) | 71 (70.3, 71.6) | 34 (32, 38) | 79.7 (79, 80.3) | 16 (12, 19) | 79.5 (78.8, 80.1) | 24 (21, 27) |
| **Roosevelt** | 73.8 (73.1, 74.4) | 14 (12, 18) | 73.6 (72.8, 74.2) | 22 (20, 26) | 79.7 (79, 80.3) | 16 (12, 19) | 79.3 (78.5, 80) | 25 (21, 28) |
| **San Juan** | 73.6 (73.2, 73.9) | 16 (14, 17) | 73.9 (73.5, 74.3) | 21 (19, 23) | 79.6 (79.2, 80.1) | 16 (14, 18) | 80.1 (79.7, 80.5) | 20 (18, 23) |
| **San Miguel** | 72.5 (71.8, 73) | 21 (18, 24) | 72.7 (71.9, 73.2) | 27 (24, 30) | 79.2 (78.6, 79.8) | 18 (15, 21) | 79.8 (79, 80.5) | 22 (18, 26) |
| **Sandoval** | 75.7 (75.2, 76.1) | 6 (4, 8) | 76.4 (75.9, 76.8) | 10 (8, 12) | 81.2 (80.7, 81.7) | 7 (5, 10) | 81.1 (80.6, 81.6) | 15 (12, 17) |
| **Santa Fe** | 76.1 (75.6, 76.5) | 4 (3, 6) | 78.1 (77.6, 78.6) | 3 (1, 5) | 81.5 (81, 81.9) | 6 (4, 8) | 82.9 (82.3, 83.4) | 6 (4, 9) |
| **Sierra** | 72.7 (71.8, 73.3) | 20 (17, 24) | 73.3 (72.4, 73.9) | 24 (21, 28) | 80.2 (79.4, 80.8) | 13 (9, 17) | 79.8 (78.9, 80.5) | 22 (18, 27) |
| **Socorro** | 72.6 (71.8, 73.1) | 20 (18, 24) | 74.3 (73.3, 74.9) | 19 (16, 24) | 79.4 (78.6, 80) | 17 (14, 21) | 79.4 (78.5, 80.1) | 24 (20, 28) |
| **Taos** | 74.3 (73.7, 74.8) | 12 (10, 15) | 75.4 (74.7, 76) | 14 (11, 17) | 80.8 (80.2, 81.4) | 9 (6, 13) | 81.8 (81, 82.5) | 11 (8, 15) |
| **Torrance** | 73.4 (72.6, 74) | 16 (13, 20) | 74.3 (73.5, 75) | 19 (16, 23) | 79.8 (79, 80.5) | 15 (11, 19) | 79.4 (78.5, 80.2) | 24 (20, 28) |
| **Union** | 75 (74.2, 75.6) | 9 (6, 13) | 75.4 (74.5, 76.1) | 14 (11, 18) | 80.6 (79.9, 81.3) | 10 (7, 15) | 80.3 (79.5, 80.9) | 19 (16, 24) |
| **Valencia** | 73.2 (72.6, 73.6) | 17 (15, 20) | 73.4 (72.9, 73.9) | 23 (21, 26) | 79.8 (79.2, 80.3) | 15 (12, 18) | 79.6 (78.9, 80.1) | 23 (21, 26) |
| **NEW YORK** |  |  |  |  |  |  |  |  |
| **Albany** | 75.2 (74.9, 75.4) | 8 (7, 9) | 76.1 (75.9, 76.4) | 11 (10, 12) | 80.3 (80, 80.5) | 13 (11, 14) | 80.8 (80.5, 81.1) | 16 (15, 18) |
| **Allegany** | 74 (73.5, 74.4) | 13 (12, 16) | 75.6 (74.9, 76.1) | 13 (11, 16) | 79.9 (79.3, 80.4) | 15 (12, 18) | 79.9 (79.2, 80.4) | 22 (19, 25) |
| **Bronx** | 72 (71.9, 72.1) | 23 (23, 23) | 73.9 (73.8, 74.1) | 21 (20, 21) | 78.3 (78.2, 78.4) | 22 (21, 22) | 80.5 (80.4, 80.7) | 18 (17, 19) |
| **Broome** | 75.1 (74.8, 75.4) | 8 (7, 10) | 75.5 (75.1, 75.8) | 14 (12, 15) | 80.1 (79.8, 80.4) | 13 (12, 15) | 81.2 (80.9, 81.6) | 14 (12, 16) |
| **Cattaraugus** | 73.9 (73.4, 74.3) | 14 (12, 16) | 74.5 (74, 74.9) | 18 (16, 21) | 79.2 (78.7, 79.6) | 18 (16, 20) | 80 (79.4, 80.4) | 21 (19, 24) |
| **Cayuga** | 74.7 (74.3, 75) | 10 (9, 12) | 76 (75.5, 76.3) | 11 (10, 14) | 80.2 (79.8, 80.6) | 13 (10, 15) | 80.8 (80.2, 81.2) | 17 (14, 20) |
| **Chautauqua** | 74.6 (74.2, 74.9) | 11 (9, 12) | 75.7 (75.2, 76) | 13 (11, 15) | 80.2 (79.7, 80.5) | 13 (11, 15) | 80.4 (80, 80.8) | 18 (16, 21) |
| **Chemung** | 74.5 (74.1, 74.8) | 11 (10, 13) | 75.4 (75, 75.8) | 14 (12, 16) | 79.6 (79.2, 79.9) | 16 (15, 18) | 80 (79.6, 80.4) | 21 (19, 23) |
| **Chenango** | 74.2 (73.6, 74.6) | 13 (11, 15) | 74.9 (74.3, 75.3) | 16 (14, 19) | 79.7 (79.2, 80.2) | 16 (13, 18) | 79.8 (79.2, 80.2) | 22 (20, 25) |
| **Clinton** | 73.9 (73.5, 74.3) | 14 (12, 16) | 75.8 (75.2, 76.3) | 12 (10, 15) | 79.6 (79.1, 80.1) | 16 (13, 18) | 80.5 (80, 81) | 18 (15, 21) |
| **Columbia** | 75.2 (74.7, 75.5) | 8 (6, 10) | 75.9 (75.4, 76.3) | 12 (10, 14) | 79.8 (79.4, 80.2) | 15 (13, 18) | 80.6 (80.1, 81.1) | 17 (15, 20) |
| **Cortland** | 74.4 (73.9, 74.8) | 12 (10, 14) | 75.2 (74.6, 75.7) | 15 (13, 18) | 79.6 (79.1, 80.1) | 16 (14, 19) | 80.2 (79.5, 80.7) | 20 (17, 24) |
| **Delaware** | 75.1 (74.5, 75.5) | 9 (7, 11) | 75 (74.4, 75.5) | 16 (13, 19) | 80.7 (80.2, 81.3) | 10 (7, 13) | 80 (79.4, 80.4) | 21 (18, 24) |
| **Dutchess** | 75.6 (75.4, 75.9) | 6 (5, 7) | 77.4 (77.1, 77.6) | 6 (5, 7) | 80.2 (79.9, 80.4) | 13 (12, 15) | 81.6 (81.3, 81.9) | 12 (11, 14) |
| **Erie** | 74.3 (74.2, 74.5) | 12 (11, 13) | 75.3 (75.1, 75.4) | 15 (14, 15) | 79.5 (79.3, 79.6) | 17 (16, 18) | 80.2 (80, 80.4) | 20 (19, 21) |
| **Essex** | 74.7 (74.1, 75.1) | 11 (9, 13) | 75.6 (75.1, 76.1) | 13 (11, 16) | 79.9 (79.3, 80.4) | 15 (12, 18) | 80.7 (80.1, 81.3) | 17 (14, 21) |
| **Franklin** | 73.3 (72.8, 73.7) | 17 (15, 19) | 75 (74.4, 75.4) | 16 (14, 19) | 79.2 (78.7, 79.7) | 18 (16, 20) | 80.1 (79.4, 80.6) | 21 (17, 24) |
| **Fulton** | 74.8 (74.2, 75.2) | 10 (8, 13) | 75.2 (74.6, 75.6) | 15 (13, 18) | 80 (79.4, 80.4) | 14 (12, 17) | 81.1 (80.5, 81.6) | 15 (12, 18) |
| **Genesee** | 74.6 (74.1, 75) | 11 (9, 13) | 76.3 (75.7, 76.7) | 10 (9, 13) | 80.3 (79.8, 80.8) | 12 (10, 15) | 81.2 (80.6, 81.7) | 14 (12, 18) |
| **Greene** | 73.9 (73.4, 74.4) | 14 (12, 16) | 73.8 (73.3, 74.3) | 21 (19, 24) | 79.6 (79.1, 80.1) | 16 (13, 19) | 80 (79.3, 80.5) | 21 (18, 25) |
| **Hamilton** | 74.7 (74.1, 75.1) | 11 (9, 13) | 75.6 (75.1, 76.1) | 13 (11, 16) | 79.9 (79.3, 80.4) | 15 (12, 18) | 80.7 (80.1, 81.3) | 17 (14, 21) |
| **Herkimer** | 74.9 (74.4, 75.3) | 10 (7, 12) | 75.4 (74.9, 75.9) | 14 (12, 16) | 79.9 (79.4, 80.3) | 15 (12, 17) | 79.8 (79.3, 80.3) | 22 (19, 25) |
| **Jefferson** | 74.8 (74.4, 75.2) | 10 (8, 12) | 75.8 (75.3, 76.1) | 12 (11, 15) | 79.5 (79.1, 80) | 17 (14, 19) | 80.7 (80.2, 81.2) | 17 (14, 20) |
| **Kings** | 73.9 (73.8, 74) | 14 (13, 14) | 76.8 (76.7, 76.9) | 8 (8, 9) | 79.6 (79.5, 79.7) | 16 (16, 17) | 82.3 (82.2, 82.4) | 9 (9, 10) |
| **Lewis** | 74.5 (73.8, 75) | 11 (9, 14) | 75.4 (74.7, 76) | 14 (11, 18) | 80.2 (79.5, 80.8) | 13 (10, 17) | 81 (80.2, 81.8) | 15 (11, 20) |
| **Livingston** | 75 (74.5, 75.4) | 9 (7, 11) | 76.5 (75.9, 76.9) | 10 (8, 12) | 79.5 (79, 79.9) | 17 (14, 19) | 81.2 (80.6, 81.7) | 14 (12, 18) |
| **Madison** | 75.4 (74.9, 75.8) | 7 (5, 9) | 76 (75.5, 76.5) | 11 (9, 13) | 80.1 (79.6, 80.6) | 13 (11, 16) | 80.5 (79.9, 81) | 18 (15, 21) |
| **Monroe** | 75.6 (75.4, 75.8) | 6 (5, 7) | 76.9 (76.7, 77.1) | 8 (7, 9) | 80.4 (80.2, 80.6) | 12 (11, 13) | 81.4 (81.2, 81.6) | 13 (12, 14) |
| **Montgomery** | 74.7 (74.1, 75.2) | 10 (8, 13) | 74.3 (73.7, 74.7) | 19 (17, 22) | 80 (79.4, 80.4) | 14 (12, 17) | 80.7 (80.1, 81.2) | 17 (14, 21) |
| **Nassau** | 77.2 (77, 77.3) | 0 (0, 1) | 79.4 (79.2, 79.5) | -3 (-4, -2) | 81.5 (81.3, 81.6) | 6 (5, 7) | 83.3 (83.2, 83.5) | 4 (4, 5) |
| **New York** | 75.5 (75.4, 75.7) | 6 (6, 7) | 78.7 (78.6, 78.9) | 1 (0, 1) | 81.2 (81.1, 81.3) | 7 (7, 8) | 83.7 (83.5, 83.8) | 3 (2, 3) |
| **Niagara** | 74.1 (73.8, 74.3) | 13 (12, 14) | 74.9 (74.6, 75.1) | 17 (15, 18) | 79.4 (79.1, 79.7) | 17 (16, 19) | 80.1 (79.7, 80.4) | 21 (19, 23) |
| **Oneida** | 74.7 (74.4, 74.9) | 11 (9, 12) | 75.5 (75.2, 75.8) | 13 (12, 15) | 80 (79.7, 80.3) | 14 (12, 16) | 80.7 (80.4, 81) | 17 (15, 19) |
| **Onondaga** | 75.1 (74.9, 75.3) | 8 (7, 9) | 76.3 (76, 76.5) | 10 (9, 11) | 80 (79.7, 80.2) | 14 (13, 16) | 81.3 (81.1, 81.6) | 14 (12, 15) |
| **Ontario** | 74.9 (74.5, 75.3) | 9 (8, 11) | 76.9 (76.4, 77.3) | 8 (7, 10) | 80.7 (80.3, 81.1) | 10 (8, 13) | 81.7 (81.2, 82.1) | 12 (10, 14) |
| **Orange** | 74.9 (74.7, 75.2) | 9 (8, 11) | 76.3 (76, 76.6) | 10 (9, 11) | 79.5 (79.2, 79.8) | 17 (15, 18) | 80.9 (80.6, 81.2) | 16 (14, 18) |
| **Orleans** | 73.4 (72.9, 73.9) | 16 (14, 19) | 75.5 (74.9, 75.9) | 14 (12, 17) | 78.8 (78.2, 79.3) | 20 (18, 22) | 80.6 (79.9, 81.2) | 17 (14, 21) |
| **Oswego** | 73.6 (73.2, 73.9) | 15 (14, 17) | 74.9 (74.5, 75.3) | 16 (15, 18) | 79 (78.6, 79.3) | 19 (18, 21) | 79.3 (78.8, 79.7) | 25 (23, 27) |
| **Otsego** | 75 (74.5, 75.4) | 9 (7, 11) | 75.7 (75.2, 76.2) | 12 (11, 15) | 80.7 (80.2, 81.2) | 10 (7, 13) | 81.3 (80.8, 81.8) | 14 (11, 17) |
| **Putnam** | 76.9 (76.4, 77.3) | 1 (0, 3) | 78.6 (78.1, 79) | 1 (-1, 4) | 80.9 (80.4, 81.3) | 9 (7, 12) | 82.6 (82, 83.1) | 8 (6, 10) |
| **Queens** | 76.5 (76.4, 76.6) | 2 (2, 3) | 79 (78.9, 79.1) | -1 (-2, 0) | 81.4 (81.3, 81.5) | 6 (5, 7) | 83.7 (83.6, 83.8) | 3 (2, 3) |
| **Rensselaer** | 74.8 (74.4, 75.1) | 10 (9, 12) | 75.8 (75.4, 76.1) | 12 (11, 14) | 79.3 (78.9, 79.6) | 18 (16, 19) | 80.3 (79.9, 80.6) | 19 (17, 22) |
| **Richmond** | 74.6 (74.3, 74.7) | 11 (10, 12) | 76.6 (76.3, 76.7) | 9 (9, 10) | 79.5 (79.3, 79.7) | 17 (16, 18) | 81.4 (81.1, 81.6) | 13 (12, 15) |
| **Rockland** | 77 (76.7, 77.2) | 1 (0, 2) | 78.7 (78.4, 79) | 1 (-1, 2) | 80.8 (80.5, 81) | 9 (8, 11) | 82.8 (82.5, 83.1) | 7 (6, 8) |
| **Saratoga** | 76.6 (76.3, 76.9) | 2 (1, 3) | 78 (77.6, 78.3) | 4 (3, 5) | 81.1 (80.7, 81.4) | 8 (6, 10) | 82.3 (81.9, 82.7) | 9 (7, 11) |
| **Schenectady** | 75.3 (74.9, 75.6) | 8 (6, 9) | 76 (75.7, 76.4) | 11 (10, 13) | 80.2 (79.8, 80.5) | 13 (11, 15) | 81.2 (80.8, 81.6) | 14 (12, 16) |
| **Schoharie** | 74.9 (74.2, 75.4) | 9 (7, 13) | 75.7 (75, 76.3) | 12 (10, 16) | 79.7 (79.1, 80.2) | 16 (13, 19) | 81.2 (80.4, 81.8) | 14 (11, 19) |
| **Schuyler** | 74.3 (73.6, 74.8) | 12 (10, 16) | 76.2 (75.4, 76.8) | 11 (8, 14) | 80.6 (79.8, 81.3) | 11 (7, 15) | 80.5 (79.6, 81.2) | 18 (14, 23) |
| **Seneca** | 74.8 (74.2, 75.3) | 10 (8, 13) | 75.4 (74.7, 75.9) | 14 (12, 17) | 79.3 (78.7, 79.8) | 18 (15, 20) | 80.4 (79.7, 81) | 19 (15, 23) |
| **St. Lawrence** | 73.8 (73.4, 74.2) | 14 (13, 16) | 74.5 (74.1, 74.9) | 18 (17, 20) | 79.4 (78.9, 79.8) | 17 (15, 19) | 79.1 (78.7, 79.5) | 26 (24, 28) |
| **Steuben** | 74.4 (74, 74.8) | 12 (10, 14) | 75.4 (75, 75.8) | 14 (12, 16) | 80.1 (79.7, 80.5) | 14 (11, 16) | 80.8 (80.3, 81.2) | 17 (14, 20) |
| **Suffolk** | 75.8 (75.6, 75.9) | 5 (5, 6) | 77.6 (77.4, 77.7) | 6 (5, 6) | 80.1 (80, 80.3) | 13 (12, 14) | 81.7 (81.6, 81.9) | 12 (11, 12) |
| **Sullivan** | 73.2 (72.7, 73.5) | 17 (16, 20) | 74.6 (74.1, 74.9) | 18 (16, 20) | 79.2 (78.7, 79.6) | 18 (16, 20) | 79.9 (79.3, 80.4) | 22 (19, 25) |
| **Tioga** | 75 (74.4, 75.4) | 9 (7, 12) | 76 (75.5, 76.5) | 11 (9, 14) | 80.3 (79.8, 80.8) | 12 (9, 15) | 80.6 (80, 81.2) | 17 (14, 21) |
| **Tompkins** | 76.7 (76.3, 77.1) | 2 (0, 3) | 77.7 (77.2, 78.2) | 5 (3, 7) | 81 (80.5, 81.5) | 8 (6, 11) | 81.4 (80.9, 81.9) | 13 (11, 16) |
| **Ulster** | 74.8 (74.5, 75.1) | 10 (8, 11) | 76.6 (76.2, 76.9) | 9 (8, 11) | 79.8 (79.4, 80) | 15 (14, 17) | 80.9 (80.5, 81.3) | 16 (14, 18) |
| **Warren** | 75.1 (74.6, 75.5) | 8 (7, 11) | 76.7 (76.1, 77.1) | 9 (7, 11) | 80.3 (79.8, 80.7) | 13 (10, 15) | 81.4 (80.8, 81.9) | 13 (11, 16) |
| **Washington** | 74.3 (73.8, 74.7) | 12 (10, 15) | 76.1 (75.5, 76.5) | 11 (9, 13) | 79.7 (79.2, 80.2) | 15 (13, 18) | 80.2 (79.7, 80.7) | 20 (17, 23) |
| **Wayne** | 74.6 (74.1, 74.9) | 11 (9, 13) | 75.6 (75.1, 76) | 13 (11, 15) | 79.7 (79.2, 80.1) | 16 (14, 18) | 80.7 (80.1, 81.2) | 17 (14, 20) |
| **Westchester** | 77 (76.9, 77.2) | 1 (0, 1) | 79.2 (79.1, 79.4) | -2 (-3, -1) | 81.9 (81.7, 82) | 4 (3, 5) | 83.3 (83.2, 83.5) | 4 (3, 5) |
| **Wyoming** | 74.1 (73.6, 74.6) | 13 (11, 16) | 74.9 (74.3, 75.4) | 16 (14, 19) | 79.9 (79.3, 80.5) | 14 (11, 18) | 80.5 (79.8, 81.2) | 18 (14, 22) |
| **Yates** | 74.2 (73.5, 74.7) | 13 (10, 16) | 76 (75.2, 76.6) | 12 (9, 15) | 79.5 (78.9, 80.1) | 17 (13, 20) | 81.5 (80.6, 82.3) | 13 (9, 17) |
| **NORTH CAROLINA** |  |  |  |  |  |  |  |  |
| **Alamance** | 72.8 (72.5, 73.1) | 19 (18, 21) | 74.2 (73.8, 74.4) | 20 (19, 22) | 78.6 (78.3, 78.9) | 21 (20, 22) | 79.4 (79, 79.7) | 24 (23, 26) |
| **Alexander** | 72.7 (72, 73.1) | 20 (18, 23) | 73.9 (73.2, 74.4) | 21 (19, 24) | 79 (78.4, 79.5) | 19 (17, 22) | 78.9 (78.3, 79.5) | 26 (24, 29) |
| **Alleghany** | 73.3 (72.6, 73.8) | 17 (15, 20) | 73.7 (73, 74.2) | 22 (20, 26) | 79.4 (78.9, 79.9) | 17 (15, 20) | 79.2 (78.5, 79.7) | 25 (23, 28) |
| **Anson** | 69.8 (69.1, 70.2) | 37 (32, 45) | 70.9 (70.2, 71.3) | 35 (33, 39) | 77 (76.4, 77.4) | 27 (25, 29) | 77.1 (76.5, 77.6) | 33 (32, 36) |
| **Ashe** | 73.3 (72.6, 73.8) | 17 (15, 20) | 73.7 (73, 74.2) | 22 (20, 26) | 79.4 (78.9, 79.9) | 17 (15, 20) | 79.2 (78.5, 79.7) | 25 (23, 28) |
| **Avery** | 72.5 (71.8, 73) | 21 (18, 24) | 73.1 (72.2, 73.6) | 25 (22, 29) | 79.4 (78.6, 79.9) | 17 (14, 21) | 79.5 (78.6, 80.1) | 24 (20, 28) |
| **Beaufort** | 71.4 (70.9, 71.7) | 26 (24, 28) | 73 (72.5, 73.4) | 25 (23, 28) | 77.6 (77.2, 77.9) | 24 (23, 26) | 78.5 (78.1, 78.9) | 28 (27, 30) |
| **Bertie** | 68.3 (67.5, 68.7) | 51 (48, 51) | 68.8 (68, 69.3) | 51 (51, 51) | 74.8 (74.2, 75.2) | 39 (36, 42) | 76.2 (75.5, 76.7) | 37 (35, 41) |
| **Bladen** | 69.4 (68.8, 69.8) | 43 (37, 47) | 69.3 (68.7, 69.6) | 51 (46, 51) | 76.3 (75.9, 76.7) | 29 (28, 32) | 76.7 (76.2, 77.1) | 35 (33, 37) |
| **Brunswick** | 72.7 (72.2, 73.1) | 20 (18, 22) | 74.4 (73.9, 74.8) | 19 (17, 21) | 79.2 (78.8, 79.6) | 18 (16, 20) | 80 (79.5, 80.4) | 21 (19, 24) |
| **Buncombe** | 73.6 (73.3, 73.8) | 16 (14, 17) | 74.6 (74.3, 74.9) | 18 (17, 19) | 79.7 (79.4, 80) | 16 (14, 17) | 80.5 (80.1, 80.8) | 18 (17, 20) |
| **Burke** | 72.6 (72.2, 73) | 20 (18, 22) | 73.3 (72.8, 73.6) | 24 (22, 26) | 78.9 (78.4, 79.2) | 20 (18, 21) | 79.3 (78.8, 79.7) | 25 (22, 27) |
| **Cabarrus** | 73.8 (73.4, 74.1) | 14 (13, 16) | 74.2 (73.9, 74.5) | 19 (18, 21) | 79.1 (78.7, 79.4) | 19 (17, 20) | 79 (78.7, 79.4) | 26 (25, 28) |
| **Caldwell** | 72.7 (72.2, 73.1) | 20 (18, 22) | 72.9 (72.4, 73.3) | 26 (24, 28) | 78.7 (78.2, 79.1) | 21 (19, 22) | 78.7 (78.2, 79.2) | 27 (25, 29) |
| **Camden** | 71.5 (70.9, 71.9) | 25 (23, 28) | 72.9 (72.3, 73.4) | 26 (23, 29) | 77.7 (77.1, 78.2) | 24 (22, 26) | 78.2 (77.6, 78.7) | 29 (27, 32) |
| **Carteret** | 73.8 (73.3, 74.2) | 14 (13, 17) | 74.6 (74, 75) | 18 (16, 20) | 79.3 (78.9, 79.7) | 18 (16, 20) | 79.8 (79.4, 80.3) | 22 (19, 25) |
| **Caswell** | 70.8 (70.3, 71.2) | 28 (26, 31) | 71.7 (71.1, 72.2) | 31 (29, 34) | 77.6 (77, 78.1) | 25 (23, 27) | 78.1 (77.4, 78.6) | 30 (28, 32) |
| **Catawba** | 72.8 (72.4, 73.1) | 19 (18, 21) | 74 (73.6, 74.3) | 21 (19, 22) | 78.9 (78.5, 79.2) | 20 (18, 21) | 79.1 (78.8, 79.4) | 26 (24, 27) |
| **Chatham** | 74.1 (73.5, 74.4) | 13 (12, 16) | 75.4 (74.8, 75.8) | 14 (12, 17) | 80.1 (79.6, 80.5) | 13 (11, 16) | 80.5 (80, 80.8) | 18 (16, 21) |
| **Cherokee** | 73.1 (72.4, 73.6) | 18 (15, 21) | 73.4 (72.7, 73.9) | 23 (21, 27) | 79.9 (79.3, 80.4) | 15 (12, 18) | 79.7 (79, 80.2) | 23 (20, 26) |
| **Chowan** | 71.2 (70.6, 71.6) | 26 (25, 29) | 73 (72.4, 73.4) | 25 (23, 28) | 78.1 (77.6, 78.5) | 23 (21, 25) | 78.8 (78.2, 79.2) | 27 (25, 29) |
| **Clay** | 73.1 (72.4, 73.6) | 18 (15, 21) | 73.4 (72.7, 73.9) | 23 (21, 27) | 79.9 (79.3, 80.4) | 15 (12, 18) | 79.7 (79, 80.2) | 23 (20, 26) |
| **Cleveland** | 71 (70.6, 71.3) | 27 (26, 29) | 71.8 (71.4, 72.1) | 31 (29, 32) | 78.1 (77.7, 78.4) | 23 (21, 24) | 78.2 (77.7, 78.5) | 29 (28, 31) |
| **Columbus** | 69.5 (69.1, 69.8) | 41 (36, 45) | 69.4 (68.9, 69.7) | 50 (45, 51) | 77 (76.6, 77.3) | 27 (26, 29) | 76.8 (76.4, 77.1) | 35 (33, 36) |
| **Craven** | 72.9 (72.5, 73.2) | 19 (17, 21) | 74.4 (74, 74.8) | 19 (17, 21) | 78.6 (78.2, 78.9) | 21 (20, 22) | 79.2 (78.8, 79.6) | 25 (23, 27) |
| **Cumberland** | 71.1 (70.9, 71.3) | 26 (26, 28) | 72.6 (72.3, 72.8) | 27 (26, 28) | 77.5 (77.3, 77.8) | 25 (24, 26) | 78.4 (78.1, 78.6) | 29 (28, 30) |
| **Currituck** | 73.3 (72.5, 73.8) | 17 (15, 21) | 73.7 (72.9, 74.3) | 22 (19, 26) | 79.6 (78.9, 80.2) | 16 (13, 20) | 79.6 (78.9, 80.3) | 23 (20, 27) |
| **Dare** | 75.2 (74.5, 75.7) | 8 (6, 11) | 75.9 (75.2, 76.4) | 12 (10, 15) | 80.1 (79.4, 80.6) | 14 (10, 17) | 80.4 (79.7, 80.9) | 19 (16, 23) |
| **Davidson** | 73.3 (73, 73.6) | 17 (15, 18) | 73 (72.7, 73.4) | 25 (24, 27) | 78.9 (78.5, 79.2) | 20 (18, 21) | 78.8 (78.4, 79.2) | 27 (25, 28) |
| **Davie** | 73.7 (73.1, 74.1) | 15 (13, 18) | 74.7 (74, 75.2) | 17 (15, 20) | 79.5 (78.8, 80) | 17 (14, 20) | 80.2 (79.5, 80.7) | 20 (17, 24) |
| **Duplin** | 70 (69.6, 70.4) | 33 (31, 40) | 71.3 (70.8, 71.6) | 33 (31, 35) | 76.9 (76.5, 77.2) | 27 (26, 29) | 78.3 (77.8, 78.7) | 29 (27, 31) |
| **Durham** | 72.7 (72.4, 72.9) | 20 (19, 21) | 74.4 (74.2, 74.6) | 19 (18, 20) | 77.8 (77.5, 78) | 24 (23, 25) | 80.1 (79.8, 80.3) | 21 (19, 22) |
| **Edgecombe** | 67.9 (67.4, 68.2) | 51 (51, 51) | 68.9 (68.3, 69.2) | 51 (51, 51) | 76 (75.6, 76.4) | 31 (29, 34) | 75.6 (75.2, 76) | 40 (38, 43) |
| **Forsyth** | 73.5 (73.2, 73.7) | 16 (15, 17) | 74.5 (74.3, 74.8) | 18 (17, 19) | 79.1 (78.8, 79.3) | 19 (18, 20) | 79.5 (79.2, 79.7) | 24 (23, 25) |
| **Franklin** | 71.9 (71.4, 72.2) | 23 (22, 25) | 72.4 (71.9, 72.7) | 28 (27, 30) | 78 (77.5, 78.3) | 23 (22, 25) | 78.8 (78.3, 79.2) | 27 (25, 29) |
| **Gaston** | 71 (70.7, 71.3) | 27 (26, 29) | 71.9 (71.6, 72.2) | 30 (29, 32) | 77.5 (77.2, 77.8) | 25 (24, 26) | 78.1 (77.8, 78.5) | 30 (28, 31) |
| **Gates** | 71.5 (70.9, 71.9) | 25 (23, 28) | 72.9 (72.3, 73.4) | 26 (23, 29) | 77.7 (77.1, 78.2) | 24 (22, 26) | 78.2 (77.6, 78.7) | 29 (27, 32) |
| **Graham** | 72.1 (71.4, 72.6) | 22 (20, 25) | 72 (71.3, 72.6) | 30 (27, 33) | 79 (78.4, 79.6) | 19 (16, 22) | 78.6 (77.8, 79.1) | 28 (26, 31) |
| **Granville** | 71.1 (70.7, 71.5) | 26 (25, 29) | 71.8 (71.3, 72.1) | 31 (29, 33) | 77.8 (77.4, 78.2) | 24 (22, 25) | 78.4 (77.9, 78.8) | 29 (27, 31) |
| **Greene** | 70.3 (69.6, 70.7) | 31 (29, 39) | 70.4 (69.7, 70.9) | 37 (35, 45) | 77 (76.5, 77.5) | 27 (25, 29) | 76.7 (76.1, 77.2) | 35 (33, 38) |
| **Guilford** | 73.4 (73.2, 73.5) | 16 (16, 18) | 74.9 (74.7, 75.1) | 16 (15, 17) | 79.3 (79.1, 79.5) | 18 (17, 19) | 80.5 (80.3, 80.7) | 18 (17, 19) |
| **Halifax** | 69.2 (68.7, 69.5) | 45 (41, 48) | 69.5 (69, 69.8) | 49 (43, 51) | 76.5 (76.1, 76.9) | 29 (27, 31) | 76.9 (76.4, 77.2) | 34 (33, 36) |
| **Harnett** | 71.5 (71.1, 71.8) | 25 (24, 27) | 72.6 (72.2, 72.9) | 27 (26, 29) | 78.1 (77.7, 78.4) | 23 (21, 24) | 78.2 (77.7, 78.5) | 29 (28, 31) |
| **Haywood** | 73.8 (73.2, 74.3) | 14 (12, 17) | 74 (73.4, 74.5) | 20 (18, 23) | 79.9 (79.4, 80.4) | 15 (12, 17) | 80.2 (79.6, 80.7) | 20 (17, 23) |
| **Henderson** | 75 (74.5, 75.4) | 9 (7, 11) | 75.6 (75, 75.9) | 13 (12, 16) | 80.7 (80.3, 81.1) | 10 (8, 12) | 80.8 (80.3, 81.2) | 16 (14, 19) |
| **Hertford** | 68.2 (67.5, 68.7) | 51 (48, 51) | 69.8 (69.2, 70.3) | 44 (38, 51) | 75.9 (75.4, 76.4) | 31 (29, 35) | 75.8 (75.2, 76.3) | 39 (37, 43) |
| **Hoke** | 70 (69.5, 70.4) | 34 (31, 41) | 71 (70.6, 71.4) | 34 (32, 36) | 76.8 (76.4, 77.2) | 27 (26, 29) | 76.9 (76.4, 77.3) | 34 (33, 36) |
| **Hyde** | 70.2 (69.6, 70.6) | 32 (29, 40) | 71.6 (70.9, 72) | 32 (30, 34) | 76.8 (76.3, 77.2) | 27 (26, 30) | 77.6 (76.9, 78) | 32 (30, 34) |
| **Iredell** | 72.8 (72.4, 73.1) | 19 (18, 21) | 73.9 (73.5, 74.2) | 21 (20, 23) | 79 (78.6, 79.3) | 19 (18, 21) | 80.2 (79.7, 80.5) | 20 (18, 23) |
| **Jackson** | 73.6 (73, 74) | 15 (13, 18) | 74.6 (73.9, 75.1) | 18 (15, 21) | 80.3 (79.7, 80.9) | 12 (9, 16) | 79.6 (79, 80.2) | 23 (20, 26) |
| **Johnston** | 71.6 (71.2, 71.8) | 25 (24, 26) | 73.2 (72.8, 73.5) | 24 (23, 26) | 78.6 (78.3, 79) | 21 (19, 22) | 79.4 (79, 79.7) | 24 (22, 26) |
| **Jones** | 70 (69.6, 70.4) | 33 (31, 40) | 71.3 (70.8, 71.6) | 33 (31, 35) | 76.9 (76.5, 77.2) | 27 (26, 29) | 78.3 (77.8, 78.7) | 29 (27, 31) |
| **Lee** | 71.5 (71, 71.8) | 25 (24, 27) | 73.3 (72.7, 73.7) | 24 (22, 27) | 78.2 (77.7, 78.6) | 22 (21, 24) | 80 (79.5, 80.5) | 21 (18, 24) |
| **Lenoir** | 69.2 (68.7, 69.5) | 45 (42, 48) | 70.6 (70.2, 70.9) | 36 (35, 39) | 76.9 (76.5, 77.2) | 27 (26, 29) | 77.8 (77.3, 78.2) | 31 (29, 33) |
| **Lincoln** | 72.8 (72.3, 73.2) | 19 (17, 22) | 73.3 (72.8, 73.7) | 24 (22, 27) | 79.1 (78.6, 79.5) | 19 (17, 21) | 78.9 (78.4, 79.3) | 27 (25, 29) |
| **Macon** | 74.3 (73.6, 74.7) | 12 (10, 15) | 74.5 (73.8, 75.1) | 18 (15, 21) | 80.1 (79.5, 80.6) | 13 (11, 17) | 80.8 (80, 81.4) | 16 (13, 21) |
| **Madison** | 72.7 (72, 73.3) | 20 (17, 23) | 73.9 (73.1, 74.5) | 21 (18, 25) | 79.5 (78.8, 80.1) | 17 (14, 20) | 79 (78.3, 79.7) | 26 (23, 29) |
| **Martin** | 68.2 (67.6, 68.5) | 51 (49, 51) | 69.5 (68.9, 69.9) | 48 (42, 51) | 75.6 (75.1, 75.9) | 34 (32, 37) | 76.4 (75.9, 76.8) | 36 (34, 39) |
| **McDowell** | 73.6 (72.9, 74.1) | 15 (13, 19) | 73.2 (72.6, 73.7) | 24 (22, 28) | 80.1 (79.5, 80.6) | 14 (10, 17) | 79.2 (78.5, 79.7) | 25 (23, 28) |
| **Mecklenburg** | 73.9 (73.7, 74.1) | 14 (13, 15) | 75.6 (75.4, 75.7) | 13 (12, 14) | 79.6 (79.4, 79.8) | 16 (15, 17) | 80.7 (80.5, 80.8) | 17 (16, 18) |
| **Mitchell** | 72.8 (72.1, 73.4) | 19 (16, 23) | 72.8 (71.9, 73.4) | 27 (23, 30) | 79.2 (78.5, 79.8) | 18 (15, 21) | 79.1 (78.3, 79.8) | 26 (22, 29) |
| **Montgomery** | 71.2 (70.6, 71.6) | 26 (24, 29) | 71.8 (71.1, 72.2) | 31 (29, 34) | 78.2 (77.7, 78.7) | 22 (21, 24) | 78 (77.4, 78.5) | 30 (28, 32) |
| **Moore** | 74.8 (74.4, 75.2) | 10 (8, 12) | 75.3 (74.8, 75.7) | 14 (13, 17) | 80.3 (79.9, 80.7) | 12 (10, 15) | 80.9 (80.5, 81.3) | 16 (14, 18) |
| **Nash** | 71.2 (70.8, 71.5) | 26 (25, 28) | 71.9 (71.5, 72.2) | 30 (29, 32) | 78 (77.6, 78.3) | 23 (22, 24) | 78.5 (78.1, 78.8) | 28 (27, 30) |
| **New Hanover** | 74.3 (74, 74.6) | 12 (11, 14) | 75.5 (75.1, 75.7) | 14 (12, 15) | 79.9 (79.6, 80.2) | 15 (13, 16) | 80.8 (80.4, 81.1) | 16 (15, 18) |
| **Northampton** | 68.7 (68, 69.1) | 48 (45, 51) | 68.9 (68.2, 69.4) | 51 (50, 51) | 76.1 (75.5, 76.5) | 31 (29, 34) | 77 (76.3, 77.4) | 34 (32, 37) |
| **Onslow** | 73.2 (72.8, 73.5) | 17 (16, 19) | 74.5 (74.1, 74.8) | 18 (17, 20) | 77.9 (77.6, 78.2) | 23 (22, 25) | 79.6 (79.2, 80) | 23 (21, 25) |
| **Orange** | 75.3 (74.9, 75.6) | 8 (6, 10) | 77.4 (77, 77.8) | 6 (5, 8) | 80.3 (79.9, 80.7) | 12 (10, 15) | 81.5 (81, 81.9) | 13 (11, 15) |
| **Pamlico** | 71.4 (70.9, 71.7) | 26 (24, 28) | 73 (72.5, 73.4) | 25 (23, 28) | 77.6 (77.2, 77.9) | 24 (23, 26) | 78.5 (78.1, 78.9) | 28 (27, 30) |
| **Pasquotank** | 71.4 (70.9, 71.7) | 25 (24, 28) | 73.7 (73.2, 74.1) | 22 (20, 24) | 77.5 (77, 77.9) | 25 (24, 27) | 78.3 (77.8, 78.7) | 29 (27, 31) |
| **Pender** | 72.7 (72.1, 73.1) | 20 (18, 22) | 74.3 (73.8, 74.8) | 19 (17, 22) | 78.8 (78.3, 79.2) | 20 (18, 22) | 79.5 (79, 80) | 24 (21, 26) |
| **Perquimans** | 71.2 (70.6, 71.6) | 26 (25, 29) | 73 (72.4, 73.4) | 25 (23, 28) | 78.1 (77.6, 78.5) | 23 (21, 25) | 78.8 (78.2, 79.2) | 27 (25, 29) |
| **Person** | 72.1 (71.6, 72.5) | 22 (21, 24) | 72.2 (71.6, 72.6) | 29 (27, 31) | 77.9 (77.4, 78.3) | 23 (22, 25) | 78.3 (77.8, 78.8) | 29 (27, 31) |
| **Pitt** | 71.6 (71.2, 71.8) | 25 (24, 26) | 72.9 (72.6, 73.2) | 26 (24, 27) | 77.7 (77.3, 78) | 24 (23, 26) | 78 (77.7, 78.3) | 30 (29, 31) |
| **Polk** | 75.1 (74.4, 75.6) | 8 (6, 12) | 74.9 (74.2, 75.5) | 16 (13, 20) | 80.2 (79.6, 80.8) | 13 (9, 16) | 80.8 (80.1, 81.4) | 16 (13, 21) |
| **Randolph** | 73.8 (73.4, 74.1) | 15 (13, 16) | 73.6 (73.2, 74) | 22 (21, 24) | 79.3 (78.9, 79.6) | 18 (16, 20) | 79.7 (79.3, 80.1) | 22 (20, 25) |
| **Richmond** | 69.9 (69.4, 70.2) | 36 (32, 43) | 70.7 (70.2, 71.1) | 35 (34, 39) | 76.6 (76.1, 76.9) | 28 (27, 30) | 77.1 (76.6, 77.5) | 33 (32, 35) |
| **Robeson** | 68.7 (68.4, 68.9) | 48 (46, 50) | 68.7 (68.4, 68.9) | 51 (51, 51) | 75.9 (75.6, 76.1) | 32 (31, 34) | 76.8 (76.5, 77) | 35 (34, 36) |
| **Rockingham** | 71.2 (70.8, 71.5) | 26 (25, 28) | 71.7 (71.3, 72) | 31 (30, 33) | 77.9 (77.5, 78.2) | 23 (22, 25) | 77.9 (77.5, 78.3) | 30 (29, 32) |
| **Rowan** | 72.3 (72, 72.6) | 22 (20, 23) | 73.4 (73, 73.7) | 23 (22, 25) | 78.8 (78.4, 79.1) | 20 (19, 22) | 79.3 (79, 79.7) | 25 (23, 26) |
| **Rutherford** | 71.2 (70.8, 71.6) | 26 (25, 28) | 71.4 (70.9, 71.8) | 32 (31, 35) | 78.8 (78.3, 79.2) | 20 (18, 22) | 78 (77.4, 78.4) | 30 (29, 32) |
| **Sampson** | 70.2 (69.7, 70.5) | 32 (30, 38) | 70.9 (70.4, 71.2) | 35 (33, 37) | 77.3 (76.9, 77.6) | 25 (24, 27) | 77.6 (77.1, 78) | 31 (30, 33) |
| **Scotland** | 69 (68.4, 69.3) | 46 (44, 49) | 70.5 (69.9, 70.9) | 37 (35, 42) | 76.4 (75.9, 76.8) | 29 (28, 32) | 76.9 (76.3, 77.3) | 34 (33, 37) |
| **Stanly** | 72.4 (71.9, 72.8) | 21 (19, 23) | 73.2 (72.6, 73.6) | 24 (23, 27) | 78.4 (77.9, 78.8) | 22 (20, 23) | 79.4 (78.9, 79.8) | 24 (22, 27) |
| **Stokes** | 72.8 (72.3, 73.3) | 19 (17, 22) | 73.1 (72.5, 73.6) | 25 (22, 28) | 79.1 (78.6, 79.5) | 19 (17, 21) | 79.5 (78.9, 80) | 24 (21, 27) |
| **Surry** | 72.2 (71.7, 72.6) | 22 (20, 24) | 72.8 (72.3, 73.3) | 26 (24, 29) | 79.4 (78.9, 79.8) | 17 (15, 20) | 79 (78.5, 79.4) | 26 (24, 28) |
| **Swain** | 72.1 (71.4, 72.6) | 22 (20, 25) | 72 (71.3, 72.6) | 30 (27, 33) | 79 (78.4, 79.6) | 19 (16, 22) | 78.6 (77.8, 79.1) | 28 (26, 31) |
| **Transylvania** | 75.3 (74.6, 75.8) | 7 (5, 11) | 75.5 (74.7, 76) | 14 (11, 17) | 80.2 (79.6, 80.6) | 13 (10, 16) | 82.1 (81.3, 82.7) | 10 (7, 14) |
| **Tyrrell** | 70.2 (69.6, 70.6) | 32 (29, 40) | 71.6 (70.9, 72) | 32 (30, 34) | 76.8 (76.3, 77.2) | 27 (26, 30) | 77.6 (76.9, 78) | 32 (30, 34) |
| **Union** | 73.5 (73.1, 73.9) | 16 (14, 18) | 75.2 (74.8, 75.6) | 15 (13, 17) | 79.3 (78.8, 79.6) | 18 (16, 20) | 79.8 (79.4, 80.1) | 22 (20, 24) |
| **Vance** | 69 (68.5, 69.4) | 46 (43, 49) | 69.5 (69, 69.9) | 49 (43, 51) | 76.1 (75.6, 76.4) | 31 (29, 33) | 76.5 (76, 76.9) | 36 (34, 38) |
| **Wake** | 75.6 (75.4, 75.8) | 6 (5, 7) | 77.6 (77.4, 77.8) | 6 (5, 6) | 80.3 (80.1, 80.5) | 12 (11, 14) | 82 (81.8, 82.3) | 10 (9, 11) |
| **Warren** | 69.9 (69.2, 70.4) | 35 (31, 44) | 70.4 (69.7, 70.9) | 37 (35, 44) | 76.6 (76, 77) | 28 (27, 31) | 76.9 (76.3, 77.4) | 34 (32, 37) |
| **Washington** | 70.2 (69.6, 70.6) | 32 (29, 40) | 71.6 (70.9, 72) | 32 (30, 34) | 76.8 (76.3, 77.2) | 27 (26, 30) | 77.6 (76.9, 78) | 32 (30, 34) |
| **Watauga** | 75.1 (74.5, 75.7) | 8 (6, 11) | 76.7 (76, 77.3) | 9 (7, 11) | 80.3 (79.7, 80.8) | 12 (9, 16) | 80.8 (80.1, 81.5) | 16 (13, 20) |
| **Wayne** | 70.9 (70.5, 71.1) | 28 (26, 30) | 71.6 (71.2, 71.9) | 32 (30, 33) | 77.2 (76.9, 77.5) | 26 (25, 27) | 77.6 (77.2, 77.9) | 31 (30, 33) |
| **Wilkes** | 72.6 (72.1, 73) | 20 (18, 23) | 72.9 (72.4, 73.4) | 26 (23, 28) | 78.9 (78.4, 79.3) | 20 (18, 21) | 79 (78.5, 79.5) | 26 (24, 28) |
| **Wilson** | 69.8 (69.3, 70.1) | 37 (33, 44) | 71.1 (70.7, 71.4) | 34 (32, 36) | 76.8 (76.4, 77.1) | 28 (27, 29) | 77.6 (77.1, 77.9) | 32 (30, 33) |
| **Yadkin** | 72.7 (72.1, 73.2) | 20 (17, 22) | 73.5 (72.8, 74) | 23 (21, 27) | 79.9 (79.3, 80.5) | 15 (11, 18) | 79.3 (78.6, 79.8) | 25 (22, 28) |
| **Yancey** | 73.4 (72.6, 74) | 16 (14, 20) | 74.5 (73.7, 75.1) | 18 (15, 22) | 80 (79.3, 80.7) | 14 (10, 18) | 79.8 (79, 80.5) | 22 (18, 26) |
| **NORTH DAKOTA** |  |  |  |  |  |  |  |  |
| **Adams** | 75.1 (74.5, 75.6) | 8 (6, 11) | 75.7 (75, 76.2) | 13 (10, 16) | 81 (80.4, 81.5) | 8 (6, 12) | 81.6 (80.9, 82.2) | 12 (10, 16) |
| **Barnes** | 74.6 (73.8, 75.1) | 11 (8, 14) | 75.9 (75.1, 76.6) | 12 (9, 15) | 80.4 (79.7, 81) | 12 (8, 16) | 81.3 (80.4, 82.1) | 14 (10, 18) |
| **Benson** | 73.9 (73.1, 74.4) | 14 (12, 18) | 74.3 (73.4, 74.9) | 19 (16, 23) | 80.1 (79.5, 80.7) | 13 (10, 17) | 81.1 (80.2, 81.9) | 15 (11, 20) |
| **Billings** | 74.2 (73.4, 74.8) | 13 (10, 16) | 75.7 (74.8, 76.4) | 13 (10, 17) | 80.5 (79.7, 81.2) | 11 (7, 15) | 82.2 (81.2, 83.1) | 10 (6, 14) |
| **Bottineau** | 74.6 (74, 75) | 11 (9, 14) | 74.4 (73.8, 74.9) | 19 (16, 22) | 80.7 (80, 81.2) | 10 (7, 14) | 80.2 (79.5, 80.8) | 20 (16, 24) |
| **Bowman** | 75.1 (74.5, 75.6) | 8 (6, 11) | 75.7 (75, 76.2) | 13 (10, 16) | 81 (80.4, 81.5) | 8 (6, 12) | 81.6 (80.9, 82.2) | 12 (10, 16) |
| **Burke** | 74.4 (73.7, 74.9) | 12 (10, 15) | 76.5 (75.6, 77.1) | 10 (7, 13) | 80.6 (80, 81.2) | 10 (7, 14) | 81.9 (81.1, 82.6) | 11 (8, 15) |
| **Burleigh** | 76.2 (75.7, 76.7) | 3 (2, 6) | 77.4 (76.8, 77.9) | 6 (5, 8) | 81.9 (81.3, 82.4) | 4 (2, 7) | 83 (82.3, 83.6) | 6 (3, 9) |
| **Cass** | 76.6 (76.1, 77) | 2 (1, 4) | 77.2 (76.8, 77.6) | 7 (5, 8) | 81.3 (80.8, 81.8) | 7 (4, 9) | 82.9 (82.3, 83.4) | 6 (4, 9) |
| **Cavalier** | 74.8 (74.1, 75.3) | 10 (8, 13) | 75 (74.2, 75.6) | 16 (13, 20) | 80.2 (79.6, 80.7) | 13 (10, 16) | 81.2 (80.5, 82) | 14 (11, 18) |
| **Dickey** | 74.6 (73.9, 75.2) | 11 (8, 14) | 75.8 (74.9, 76.4) | 12 (10, 16) | 80.8 (80.1, 81.5) | 9 (6, 14) | 82 (81.1, 82.8) | 10 (7, 15) |
| **Divide** | 74.4 (73.7, 74.9) | 12 (10, 15) | 76.5 (75.6, 77.1) | 10 (7, 13) | 80.6 (80, 81.2) | 10 (7, 14) | 81.9 (81.1, 82.6) | 11 (8, 15) |
| **Dunn** | 74.2 (73.4, 74.8) | 13 (10, 16) | 75.7 (74.8, 76.4) | 13 (10, 17) | 80.5 (79.7, 81.2) | 11 (7, 15) | 82.2 (81.2, 83.1) | 10 (6, 14) |
| **Eddy** | 74.4 (73.6, 75) | 12 (9, 15) | 75.2 (74.4, 75.8) | 15 (12, 19) | 80 (79.3, 80.6) | 14 (11, 18) | 81.1 (80.2, 81.9) | 15 (11, 20) |
| **Emmons** | 74.1 (73.5, 74.6) | 13 (11, 16) | 74.6 (74, 75.1) | 18 (15, 21) | 80.3 (79.7, 80.9) | 12 (9, 16) | 80.6 (79.8, 81.2) | 18 (14, 22) |
| **Foster** | 74.4 (73.6, 75) | 12 (9, 15) | 75.2 (74.4, 75.8) | 15 (12, 19) | 80 (79.3, 80.6) | 14 (11, 18) | 81.1 (80.2, 81.9) | 15 (11, 20) |
| **Golden Valley** | 74.2 (73.4, 74.8) | 13 (10, 16) | 75.7 (74.8, 76.4) | 13 (10, 17) | 80.5 (79.7, 81.2) | 11 (7, 15) | 82.2 (81.2, 83.1) | 10 (6, 14) |
| **Grand Forks** | 75.6 (75.1, 76.1) | 6 (4, 8) | 76.5 (76, 77) | 9 (8, 11) | 80.9 (80.4, 81.4) | 9 (6, 11) | 81.4 (80.8, 81.9) | 13 (11, 16) |
| **Grant** | 75.1 (74.5, 75.6) | 8 (6, 11) | 75.7 (75, 76.2) | 13 (10, 16) | 81 (80.4, 81.5) | 8 (6, 12) | 81.6 (80.9, 82.2) | 12 (10, 16) |
| **Griggs** | 74.4 (73.6, 75) | 12 (9, 15) | 75.2 (74.4, 75.8) | 15 (12, 19) | 80 (79.3, 80.6) | 14 (11, 18) | 81.1 (80.2, 81.9) | 15 (11, 20) |
| **Hettinger** | 75.1 (74.5, 75.6) | 8 (6, 11) | 75.7 (75, 76.2) | 13 (10, 16) | 81 (80.4, 81.5) | 8 (6, 12) | 81.6 (80.9, 82.2) | 12 (10, 16) |
| **Kidder** | 74.2 (73.5, 74.6) | 13 (11, 16) | 75.2 (74.5, 75.8) | 15 (12, 18) | 80.6 (79.9, 81.1) | 11 (8, 14) | 81.3 (80.5, 82) | 14 (10, 18) |
| **La Moure** | 74.6 (73.9, 75.2) | 11 (8, 14) | 75.8 (74.9, 76.4) | 12 (10, 16) | 80.8 (80.1, 81.5) | 9 (6, 14) | 82 (81.1, 82.8) | 10 (7, 15) |
| **Logan** | 74.2 (73.5, 74.6) | 13 (11, 16) | 75.2 (74.5, 75.8) | 15 (12, 18) | 80.6 (79.9, 81.1) | 11 (8, 14) | 81.3 (80.5, 82) | 14 (10, 18) |
| **McHenry** | 74.6 (74, 75) | 11 (9, 14) | 74.4 (73.8, 74.9) | 19 (16, 22) | 80.7 (80, 81.2) | 10 (7, 14) | 80.2 (79.5, 80.8) | 20 (16, 24) |
| **McIntosh** | 74.2 (73.5, 74.6) | 13 (11, 16) | 75.2 (74.5, 75.8) | 15 (12, 18) | 80.6 (79.9, 81.1) | 11 (8, 14) | 81.3 (80.5, 82) | 14 (10, 18) |
| **McKenzie** | 74.2 (73.4, 74.8) | 13 (10, 16) | 75.7 (74.8, 76.4) | 13 (10, 17) | 80.5 (79.7, 81.2) | 11 (7, 15) | 82.2 (81.2, 83.1) | 10 (6, 14) |
| **McLean** | 74.4 (73.6, 75) | 12 (9, 15) | 75.8 (75, 76.5) | 12 (10, 16) | 80.7 (79.9, 81.3) | 10 (7, 15) | 80.8 (80, 81.5) | 16 (13, 21) |
| **Mercer** | 74.4 (73.6, 75) | 12 (9, 15) | 75.8 (75, 76.5) | 12 (10, 16) | 80.7 (79.9, 81.3) | 10 (7, 15) | 80.8 (80, 81.5) | 16 (13, 21) |
| **Morton** | 74.1 (73.5, 74.6) | 13 (11, 16) | 74.6 (74, 75.1) | 18 (15, 21) | 80.3 (79.7, 80.9) | 12 (9, 16) | 80.6 (79.8, 81.2) | 18 (14, 22) |
| **Mountrail** | 75.2 (74.7, 75.6) | 8 (6, 10) | 75.7 (75.1, 76.1) | 13 (11, 15) | 80.9 (80.4, 81.4) | 9 (6, 12) | 81.4 (80.8, 81.9) | 13 (11, 16) |
| **Nelson** | 74.4 (73.6, 75) | 12 (9, 15) | 75.2 (74.4, 75.8) | 15 (12, 19) | 80 (79.3, 80.6) | 14 (11, 18) | 81.1 (80.2, 81.9) | 15 (11, 20) |
| **Oliver** | 76.2 (75.7, 76.7) | 3 (2, 6) | 77.4 (76.8, 77.9) | 6 (5, 8) | 81.9 (81.3, 82.4) | 4 (2, 7) | 83 (82.3, 83.6) | 6 (3, 9) |
| **Pembina** | 74.7 (74, 75.2) | 10 (8, 14) | 75.8 (75, 76.5) | 12 (10, 16) | 80.4 (79.7, 81) | 12 (8, 16) | 81.4 (80.6, 82.1) | 13 (10, 18) |
| **Pierce** | 73.9 (73.1, 74.4) | 14 (12, 18) | 74.3 (73.4, 74.9) | 19 (16, 23) | 80.1 (79.5, 80.7) | 13 (10, 17) | 81.1 (80.2, 81.9) | 15 (11, 20) |
| **Ramsey** | 74.8 (74.1, 75.3) | 10 (8, 13) | 75 (74.2, 75.6) | 16 (13, 20) | 80.2 (79.6, 80.7) | 13 (10, 16) | 81.2 (80.5, 82) | 14 (11, 18) |
| **Ransom** | 74.6 (73.8, 75.1) | 11 (8, 14) | 75.9 (75.1, 76.6) | 12 (9, 15) | 80.4 (79.7, 81) | 12 (8, 16) | 81.3 (80.4, 82.1) | 14 (10, 18) |
| **Renville** | 74.6 (74, 75) | 11 (9, 14) | 74.4 (73.8, 74.9) | 19 (16, 22) | 80.7 (80, 81.2) | 10 (7, 14) | 80.2 (79.5, 80.8) | 20 (16, 24) |
| **Richland** | 75.5 (74.7, 76) | 7 (4, 10) | 76.8 (75.9, 77.5) | 8 (6, 12) | 80.9 (80.1, 81.5) | 9 (6, 13) | 82.2 (81.2, 83) | 10 (6, 14) |
| **Rolette** | 74.6 (74, 75) | 11 (9, 14) | 74.4 (73.8, 74.9) | 19 (16, 22) | 80.7 (80, 81.2) | 10 (7, 14) | 80.2 (79.5, 80.8) | 20 (16, 24) |
| **Sargent** | 74.6 (73.9, 75.2) | 11 (8, 14) | 75.8 (74.9, 76.4) | 12 (10, 16) | 80.8 (80.1, 81.5) | 9 (6, 14) | 82 (81.1, 82.8) | 10 (7, 15) |
| **Sheridan** | 73.9 (73.1, 74.4) | 14 (12, 18) | 74.3 (73.4, 74.9) | 19 (16, 23) | 80.1 (79.5, 80.7) | 13 (10, 17) | 81.1 (80.2, 81.9) | 15 (11, 20) |
| **Sioux** | 74.1 (73.5, 74.6) | 13 (11, 16) | 74.6 (74, 75.1) | 18 (15, 21) | 80.3 (79.7, 80.9) | 12 (9, 16) | 80.6 (79.8, 81.2) | 18 (14, 22) |
| **Slope** | 75.1 (74.5, 75.6) | 8 (6, 11) | 75.7 (75, 76.2) | 13 (10, 16) | 81 (80.4, 81.5) | 8 (6, 12) | 81.6 (80.9, 82.2) | 12 (10, 16) |
| **Stark** | 75.1 (74.5, 75.6) | 8 (6, 11) | 75.7 (75, 76.2) | 13 (10, 16) | 81 (80.4, 81.5) | 8 (6, 12) | 81.6 (80.9, 82.2) | 12 (10, 16) |
| **Steele** | 74.4 (73.6, 75) | 12 (9, 15) | 75.2 (74.4, 75.8) | 15 (12, 19) | 80 (79.3, 80.6) | 14 (11, 18) | 81.1 (80.2, 81.9) | 15 (11, 20) |
| **Stutsman** | 74.2 (73.5, 74.6) | 13 (11, 16) | 75.2 (74.5, 75.8) | 15 (12, 18) | 80.6 (79.9, 81.1) | 11 (8, 14) | 81.3 (80.5, 82) | 14 (10, 18) |
| **Towner** | 74.8 (74.1, 75.3) | 10 (8, 13) | 75 (74.2, 75.6) | 16 (13, 20) | 80.2 (79.6, 80.7) | 13 (10, 16) | 81.2 (80.5, 82) | 14 (11, 18) |
| **Traill** | 75.6 (75.1, 76.1) | 6 (4, 8) | 76.5 (76, 77) | 9 (8, 11) | 80.9 (80.4, 81.4) | 9 (6, 11) | 81.4 (80.8, 81.9) | 13 (11, 16) |
| **Walsh** | 74.7 (74, 75.2) | 10 (8, 14) | 75.8 (75, 76.5) | 12 (10, 16) | 80.4 (79.7, 81) | 12 (8, 16) | 81.4 (80.6, 82.1) | 13 (10, 18) |
| **Ward** | 75.2 (74.7, 75.6) | 8 (6, 10) | 75.7 (75.1, 76.1) | 13 (11, 15) | 80.9 (80.4, 81.4) | 9 (6, 12) | 81.4 (80.8, 81.9) | 13 (11, 16) |
| **Wells** | 73.9 (73.1, 74.4) | 14 (12, 18) | 74.3 (73.4, 74.9) | 19 (16, 23) | 80.1 (79.5, 80.7) | 13 (10, 17) | 81.1 (80.2, 81.9) | 15 (11, 20) |
| **Williams** | 74.4 (73.7, 74.9) | 12 (10, 15) | 76.5 (75.6, 77.1) | 10 (7, 13) | 80.6 (80, 81.2) | 10 (7, 14) | 81.9 (81.1, 82.6) | 11 (8, 15) |
| **OHIO** |  |  |  |  |  |  |  |  |
| **Adams** | 71.4 (70.7, 71.9) | 25 (23, 29) | 72.2 (71.4, 72.7) | 29 (27, 32) | 78.7 (78, 79.2) | 21 (18, 23) | 77.8 (77.1, 78.4) | 31 (29, 34) |
| **Allen** | 74 (73.7, 74.4) | 13 (12, 15) | 74.9 (74.5, 75.3) | 16 (14, 18) | 79 (78.6, 79.3) | 19 (18, 21) | 79.7 (79.3, 80.1) | 23 (21, 25) |
| **Ashland** | 74.8 (74.2, 75.3) | 10 (7, 12) | 75.7 (75.1, 76.2) | 13 (11, 15) | 79.8 (79.3, 80.3) | 15 (12, 18) | 80.5 (79.9, 81) | 18 (15, 22) |
| **Ashtabula** | 73.1 (72.7, 73.4) | 18 (16, 20) | 73.7 (73.3, 74.1) | 22 (20, 24) | 78.7 (78.3, 79.1) | 20 (19, 22) | 79.1 (78.6, 79.5) | 26 (24, 28) |
| **Athens** | 72.7 (72.2, 73.1) | 20 (18, 22) | 73.7 (73.2, 74.2) | 22 (19, 24) | 78.5 (78, 79) | 21 (19, 23) | 79.3 (78.7, 79.8) | 25 (22, 28) |
| **Auglaize** | 75.1 (74.6, 75.6) | 8 (6, 11) | 75.8 (75.2, 76.2) | 12 (10, 15) | 80.4 (79.9, 80.9) | 11 (9, 15) | 80.9 (80.2, 81.4) | 16 (13, 20) |
| **Belmont** | 73.2 (72.7, 73.5) | 17 (16, 20) | 73.5 (73, 73.9) | 23 (21, 25) | 79.4 (79, 79.8) | 17 (15, 19) | 79.4 (78.9, 79.8) | 24 (22, 27) |
| **Brown** | 72.7 (72.1, 73.2) | 20 (17, 22) | 73.5 (72.9, 74) | 23 (20, 26) | 78.7 (78.1, 79.3) | 20 (18, 23) | 78.7 (78, 79.3) | 27 (25, 30) |
| **Butler** | 73.9 (73.6, 74.2) | 14 (13, 15) | 74.9 (74.7, 75.2) | 16 (15, 18) | 79.2 (78.9, 79.5) | 18 (17, 19) | 79 (78.8, 79.3) | 26 (25, 27) |
| **Carroll** | 74.1 (73.4, 74.6) | 13 (11, 16) | 75 (74.3, 75.6) | 16 (13, 19) | 80 (79.3, 80.6) | 14 (10, 18) | 80.2 (79.5, 80.9) | 20 (16, 24) |
| **Champaign** | 74 (73.4, 74.5) | 13 (11, 16) | 74.2 (73.6, 74.7) | 19 (17, 22) | 79.2 (78.6, 79.7) | 18 (16, 21) | 79.1 (78.5, 79.7) | 25 (23, 28) |
| **Clark** | 72.6 (72.3, 72.9) | 20 (19, 22) | 73.5 (73.2, 73.8) | 23 (21, 24) | 78.5 (78.2, 78.8) | 21 (20, 22) | 78.5 (78.2, 78.8) | 28 (27, 29) |
| **Clermont** | 73.9 (73.5, 74.2) | 14 (13, 16) | 75.6 (75.2, 76) | 13 (11, 15) | 78.5 (78.1, 78.8) | 21 (20, 23) | 79.5 (79.1, 79.8) | 24 (22, 26) |
| **Clinton** | 73.7 (73.1, 74.2) | 15 (13, 18) | 74.7 (74, 75.2) | 17 (15, 20) | 79.8 (79.1, 80.3) | 15 (12, 19) | 80 (79.3, 80.6) | 21 (18, 25) |
| **Columbiana** | 73.4 (73.1, 73.8) | 16 (14, 18) | 74.2 (73.8, 74.6) | 20 (18, 21) | 78.8 (78.4, 79.1) | 20 (19, 22) | 79.4 (79, 79.8) | 24 (22, 26) |
| **Coshocton** | 73.6 (73, 74.1) | 15 (13, 18) | 74.5 (73.9, 75.1) | 18 (16, 21) | 79 (78.4, 79.5) | 19 (17, 21) | 79.6 (78.9, 80.1) | 23 (20, 27) |
| **Crawford** | 73.5 (72.9, 74) | 16 (14, 19) | 73.8 (73.2, 74.3) | 21 (19, 24) | 79.5 (79, 80) | 17 (14, 19) | 79.6 (79.1, 80.2) | 23 (20, 26) |
| **Cuyahoga** | 73.5 (73.3, 73.6) | 16 (16, 17) | 74 (73.9, 74.1) | 21 (20, 21) | 78.9 (78.7, 79) | 20 (19, 20) | 79.7 (79.6, 79.8) | 23 (22, 23) |
| **Darke** | 74.8 (74.2, 75.2) | 10 (8, 13) | 75 (74.4, 75.4) | 16 (14, 19) | 80.1 (79.5, 80.5) | 14 (11, 17) | 81.2 (80.5, 81.8) | 14 (11, 18) |
| **Defiance** | 74.7 (74.1, 75.1) | 10 (8, 13) | 75.6 (75, 76.1) | 13 (11, 16) | 80.2 (79.6, 80.8) | 13 (9, 16) | 80.7 (80, 81.3) | 17 (14, 21) |
| **Delaware** | 76.6 (76.1, 77) | 2 (1, 4) | 77.5 (77, 77.8) | 6 (5, 7) | 80.9 (80.4, 81.3) | 9 (7, 12) | 81.2 (80.7, 81.6) | 14 (12, 17) |
| **Erie** | 74.1 (73.7, 74.5) | 13 (11, 15) | 75.3 (74.9, 75.7) | 14 (13, 16) | 79.8 (79.4, 80.3) | 15 (13, 17) | 80.1 (79.7, 80.6) | 20 (18, 23) |
| **Fairfield** | 74.8 (74.4, 75.1) | 10 (8, 12) | 75.6 (75.2, 76) | 13 (11, 15) | 79.5 (79.1, 79.9) | 17 (14, 19) | 79.8 (79.4, 80.2) | 22 (20, 24) |
| **Fayette** | 72.7 (72.1, 73.2) | 20 (17, 23) | 73.2 (72.5, 73.7) | 24 (22, 28) | 78.4 (77.8, 78.9) | 21 (19, 24) | 78.6 (77.9, 79.1) | 28 (26, 31) |
| **Franklin** | 73.4 (73.2, 73.5) | 16 (16, 17) | 73.7 (73.6, 73.9) | 22 (21, 22) | 78.2 (78.1, 78.4) | 22 (22, 23) | 79 (78.8, 79.1) | 26 (26, 27) |
| **Fulton** | 74.7 (74, 75.2) | 10 (8, 13) | 75.8 (75.2, 76.3) | 12 (10, 15) | 80.1 (79.6, 80.6) | 13 (10, 16) | 81.1 (80.4, 81.7) | 15 (12, 18) |
| **Gallia** | 72.2 (71.6, 72.7) | 22 (20, 24) | 72.1 (71.4, 72.6) | 30 (27, 32) | 78.6 (78, 79.1) | 21 (19, 23) | 79.3 (78.6, 79.9) | 25 (22, 28) |
| **Geauga** | 76.6 (76.2, 77) | 2 (1, 4) | 78.1 (77.6, 78.6) | 3 (1, 5) | 80.9 (80.4, 81.3) | 9 (7, 11) | 82.5 (81.9, 83) | 8 (6, 11) |
| **Greene** | 75.1 (74.7, 75.3) | 9 (7, 10) | 76.5 (76.1, 76.8) | 10 (8, 11) | 79.8 (79.4, 80.1) | 15 (14, 17) | 80.5 (80.1, 80.8) | 18 (16, 21) |
| **Guernsey** | 72.6 (72, 73) | 21 (18, 23) | 73.4 (72.8, 73.9) | 23 (21, 26) | 78.7 (78.2, 79.2) | 20 (18, 22) | 79.4 (78.8, 80) | 24 (21, 27) |
| **Hamilton** | 73.4 (73.2, 73.5) | 16 (16, 17) | 73.9 (73.8, 74.1) | 21 (20, 22) | 78.5 (78.4, 78.6) | 21 (21, 22) | 79.4 (79.2, 79.5) | 24 (24, 25) |
| **Hancock** | 75.5 (74.9, 75.8) | 7 (5, 9) | 76.5 (76, 77) | 9 (8, 11) | 80.4 (79.9, 80.8) | 12 (9, 15) | 81.3 (80.7, 81.8) | 13 (11, 17) |
| **Hardin** | 73.5 (72.9, 73.9) | 16 (14, 19) | 73.7 (72.9, 74.2) | 22 (20, 26) | 79.5 (78.9, 80) | 17 (14, 20) | 79.8 (79, 80.4) | 22 (18, 26) |
| **Harrison** | 73.5 (72.7, 74) | 16 (13, 20) | 73.7 (72.8, 74.3) | 22 (19, 26) | 79.1 (78.4, 79.6) | 19 (16, 22) | 79 (78.3, 79.6) | 26 (23, 29) |
| **Henry** | 74.3 (73.6, 74.9) | 12 (10, 15) | 75.6 (74.9, 76.2) | 13 (11, 16) | 80.7 (80, 81.3) | 10 (7, 14) | 81 (80.2, 81.7) | 15 (12, 20) |
| **Highland** | 72.8 (72.2, 73.3) | 19 (17, 22) | 73.1 (72.4, 73.6) | 25 (23, 28) | 79.3 (78.7, 79.8) | 18 (15, 20) | 78.9 (78.2, 79.4) | 27 (24, 29) |
| **Hocking** | 73.2 (72.6, 73.8) | 17 (15, 20) | 73.4 (72.7, 74.1) | 23 (20, 27) | 78.9 (78.3, 79.6) | 20 (16, 22) | 79 (78.3, 79.7) | 26 (23, 29) |
| **Holmes** | 73 (72.3, 73.6) | 18 (15, 22) | 73.5 (72.8, 74.1) | 23 (20, 26) | 78.4 (77.7, 79) | 22 (19, 24) | 78.5 (77.8, 79.2) | 28 (25, 31) |
| **Huron** | 74.2 (73.7, 74.6) | 13 (11, 15) | 74.6 (74, 75) | 18 (16, 21) | 79.1 (78.6, 79.6) | 19 (16, 21) | 79.4 (78.8, 79.9) | 24 (22, 27) |
| **Jackson** | 71.8 (71.1, 72.3) | 24 (22, 27) | 71.4 (70.7, 71.9) | 32 (30, 36) | 77.9 (77.3, 78.4) | 23 (22, 26) | 78.2 (77.5, 78.8) | 29 (27, 32) |
| **Jefferson** | 72.5 (72, 72.8) | 21 (19, 23) | 72.6 (72.1, 73) | 27 (25, 29) | 78.3 (77.8, 78.6) | 22 (21, 24) | 78.3 (77.9, 78.7) | 29 (27, 31) |
| **Knox** | 73.7 (73.1, 74.1) | 15 (13, 18) | 74.8 (74.2, 75.2) | 17 (15, 20) | 79.8 (79.2, 80.2) | 15 (13, 18) | 79.8 (79.2, 80.3) | 22 (19, 25) |
| **Lake** | 75.1 (74.7, 75.3) | 9 (7, 10) | 76.1 (75.8, 76.4) | 11 (10, 12) | 79.9 (79.6, 80.2) | 14 (13, 16) | 81 (80.7, 81.3) | 15 (14, 17) |
| **Lawrence** | 72 (71.5, 72.4) | 23 (21, 25) | 71.7 (71.1, 72.1) | 31 (29, 33) | 77.4 (77, 77.8) | 25 (24, 27) | 77.4 (76.9, 77.9) | 32 (31, 34) |
| **Licking** | 73.9 (73.5, 74.2) | 14 (12, 16) | 75.1 (74.7, 75.4) | 15 (14, 17) | 79 (78.6, 79.3) | 19 (18, 21) | 79.6 (79.2, 80) | 23 (21, 25) |
| **Logan** | 73.9 (73.3, 74.4) | 14 (12, 17) | 74.5 (73.9, 75) | 18 (16, 21) | 79.4 (78.8, 79.9) | 17 (15, 20) | 79.4 (78.8, 79.9) | 24 (21, 27) |
| **Lorain** | 74.6 (74.3, 74.8) | 11 (10, 12) | 75.6 (75.3, 75.8) | 13 (12, 14) | 79.3 (79, 79.5) | 18 (17, 19) | 80.3 (80, 80.6) | 19 (17, 21) |
| **Lucas** | 72.8 (72.6, 73) | 19 (19, 20) | 73.4 (73.2, 73.6) | 23 (22, 24) | 78.4 (78.2, 78.6) | 22 (21, 22) | 78.8 (78.6, 79) | 27 (26, 28) |
| **Madison** | 73.1 (72.6, 73.5) | 18 (16, 20) | 74.9 (74.2, 75.3) | 17 (14, 19) | 78.8 (78.2, 79.3) | 20 (18, 22) | 79.5 (78.9, 80.1) | 24 (20, 27) |
| **Mahoning** | 72.4 (72.2, 72.7) | 21 (20, 22) | 73.3 (73.1, 73.6) | 24 (22, 25) | 78.7 (78.5, 78.9) | 20 (19, 21) | 79.6 (79.3, 79.8) | 23 (22, 25) |
| **Marion** | 73.5 (73.1, 73.9) | 16 (14, 18) | 74 (73.5, 74.4) | 21 (19, 23) | 78.8 (78.4, 79.2) | 20 (18, 22) | 78.8 (78.3, 79.3) | 27 (25, 29) |
| **Medina** | 76.5 (76.1, 76.9) | 2 (1, 4) | 77.4 (77, 77.7) | 6 (5, 8) | 80.2 (79.8, 80.5) | 13 (11, 15) | 81.3 (80.9, 81.7) | 14 (12, 16) |
| **Meigs** | 71.3 (70.7, 71.8) | 26 (24, 29) | 71.9 (71.3, 72.4) | 30 (28, 33) | 78.5 (77.9, 79) | 21 (19, 23) | 78.5 (77.7, 79.1) | 28 (26, 31) |
| **Mercer** | 74.7 (74.1, 75.2) | 10 (8, 13) | 75.7 (75, 76.1) | 13 (11, 16) | 80.5 (79.8, 81) | 11 (8, 15) | 80.7 (80.1, 81.3) | 17 (14, 21) |
| **Miami** | 74.8 (74.4, 75.2) | 10 (8, 12) | 75.5 (75, 75.9) | 13 (12, 16) | 79.6 (79.1, 79.9) | 16 (14, 19) | 80.3 (79.8, 80.7) | 19 (17, 22) |
| **Monroe** | 73.8 (73.2, 74.3) | 14 (12, 17) | 74.4 (73.7, 75) | 19 (16, 22) | 79.3 (78.7, 79.9) | 18 (15, 20) | 79.8 (79, 80.4) | 22 (18, 26) |
| **Montgomery** | 72.9 (72.7, 73.1) | 19 (18, 20) | 73.9 (73.7, 74.1) | 21 (20, 22) | 78.5 (78.3, 78.7) | 21 (20, 22) | 79.6 (79.3, 79.7) | 23 (23, 25) |
| **Morgan** | 73.1 (72.3, 73.6) | 18 (15, 21) | 73.2 (72.4, 73.8) | 24 (21, 28) | 79.5 (78.7, 80.1) | 17 (14, 20) | 79.8 (78.9, 80.6) | 22 (17, 26) |
| **Morrow** | 74.4 (73.7, 74.9) | 12 (9, 15) | 74.5 (73.8, 75.1) | 18 (15, 21) | 79.4 (78.8, 80) | 17 (14, 20) | 79.1 (78.4, 79.7) | 26 (23, 29) |
| **Muskingum** | 73.2 (72.7, 73.5) | 17 (16, 20) | 73.9 (73.4, 74.3) | 21 (19, 23) | 79.1 (78.6, 79.5) | 19 (17, 21) | 79.3 (78.8, 79.7) | 25 (23, 27) |
| **Noble** | 73.8 (73.2, 74.3) | 14 (12, 17) | 74.4 (73.7, 75) | 19 (16, 22) | 79.3 (78.7, 79.9) | 18 (15, 20) | 79.8 (79, 80.4) | 22 (18, 26) |
| **Ottawa** | 74.9 (74.3, 75.3) | 9 (7, 12) | 75.1 (74.5, 75.5) | 16 (13, 18) | 80.3 (79.7, 80.8) | 12 (10, 16) | 79.9 (79.4, 80.4) | 21 (19, 24) |
| **Paulding** | 73.6 (72.8, 74.1) | 16 (13, 19) | 74.4 (73.7, 75) | 19 (16, 22) | 80 (79.3, 80.7) | 14 (10, 18) | 80.2 (79.4, 80.9) | 20 (16, 24) |
| **Perry** | 72.6 (72, 73.1) | 20 (18, 23) | 72.8 (72.3, 73.3) | 26 (24, 29) | 78.3 (77.7, 78.8) | 22 (20, 24) | 79.6 (78.9, 80.2) | 23 (20, 27) |
| **Pickaway** | 73.6 (73, 74) | 16 (14, 18) | 74.1 (73.5, 74.6) | 20 (18, 23) | 78.3 (77.8, 78.8) | 22 (20, 24) | 79.2 (78.5, 79.7) | 25 (23, 28) |
| **Pike** | 71.5 (70.8, 72) | 25 (23, 28) | 71.4 (70.6, 71.9) | 32 (30, 36) | 78.2 (77.6, 78.7) | 22 (20, 25) | 78.4 (77.7, 79) | 29 (26, 31) |
| **Portage** | 75.2 (74.9, 75.6) | 8 (6, 10) | 75.8 (75.5, 76.2) | 12 (11, 14) | 80 (79.6, 80.4) | 14 (12, 16) | 80.1 (79.7, 80.4) | 21 (18, 23) |
| **Preble** | 74.7 (74.1, 75.2) | 10 (8, 13) | 74.7 (74, 75.2) | 17 (15, 21) | 79.8 (79.2, 80.3) | 15 (12, 18) | 79.8 (79.1, 80.4) | 22 (19, 26) |
| **Putnam** | 75 (74.4, 75.4) | 9 (7, 12) | 76.3 (75.6, 76.8) | 10 (8, 13) | 81.5 (80.8, 82) | 6 (3, 9) | 81.6 (80.9, 82.2) | 12 (9, 16) |
| **Richland** | 73.7 (73.3, 74) | 15 (14, 17) | 74.8 (74.4, 75.1) | 17 (15, 19) | 79.3 (78.9, 79.6) | 18 (16, 20) | 79.4 (79, 79.8) | 24 (22, 26) |
| **Ross** | 72.7 (72.2, 73) | 20 (18, 22) | 73.3 (72.9, 73.7) | 24 (22, 26) | 78.7 (78.2, 79.1) | 20 (19, 22) | 78.6 (78.1, 79.1) | 28 (26, 30) |
| **Sandusky** | 74.5 (74, 74.9) | 11 (9, 13) | 74.6 (74.1, 75) | 18 (16, 20) | 79.5 (79, 79.9) | 17 (15, 19) | 79.7 (79.2, 80.2) | 23 (20, 25) |
| **Scioto** | 71.4 (70.9, 71.8) | 25 (24, 28) | 71.3 (70.8, 71.7) | 33 (31, 35) | 77.8 (77.3, 78.2) | 24 (22, 25) | 78.3 (77.7, 78.7) | 29 (27, 31) |
| **Seneca** | 74.3 (73.8, 74.7) | 12 (10, 15) | 74.8 (74.2, 75.3) | 17 (15, 20) | 79.8 (79.3, 80.3) | 15 (13, 18) | 79.9 (79.3, 80.4) | 22 (19, 25) |
| **Shelby** | 74.5 (73.9, 75) | 11 (9, 14) | 75.4 (74.8, 75.9) | 14 (12, 17) | 79.6 (79, 80.1) | 16 (14, 19) | 80.3 (79.7, 80.9) | 19 (16, 23) |
| **Stark** | 74.4 (74.1, 74.5) | 12 (11, 13) | 75.6 (75.3, 75.8) | 13 (12, 14) | 79.6 (79.4, 79.8) | 16 (15, 17) | 80.4 (80.1, 80.6) | 19 (18, 20) |
| **Summit** | 74.4 (74.2, 74.5) | 12 (11, 13) | 74.8 (74.6, 75) | 17 (16, 18) | 79.5 (79.3, 79.6) | 17 (16, 18) | 80 (79.8, 80.2) | 21 (20, 22) |
| **Trumbull** | 73.3 (73, 73.6) | 17 (16, 18) | 73.7 (73.4, 74) | 22 (21, 23) | 79 (78.7, 79.3) | 19 (18, 20) | 79.5 (79.1, 79.8) | 24 (22, 26) |
| **Tuscarawas** | 74 (73.5, 74.3) | 14 (12, 16) | 75.3 (74.8, 75.7) | 14 (13, 17) | 79.5 (79.1, 79.9) | 17 (15, 19) | 80.3 (79.8, 80.7) | 19 (17, 22) |
| **Union** | 74.6 (74, 75.1) | 11 (9, 14) | 75.3 (74.7, 75.8) | 15 (12, 18) | 79.3 (78.8, 79.8) | 18 (15, 20) | 79.8 (79.2, 80.4) | 22 (18, 25) |
| **Van Wert** | 74.4 (73.8, 74.9) | 12 (9, 14) | 75.2 (74.5, 75.7) | 15 (12, 18) | 80.3 (79.7, 80.9) | 12 (9, 16) | 80.5 (79.8, 81.1) | 18 (15, 22) |
| **Vinton** | 71.3 (70.7, 71.8) | 26 (24, 29) | 71.9 (71.3, 72.4) | 30 (28, 33) | 78.5 (77.9, 79) | 21 (19, 23) | 78.5 (77.7, 79.1) | 28 (26, 31) |
| **Warren** | 75.2 (74.8, 75.6) | 8 (6, 10) | 76.7 (76.3, 77) | 9 (8, 10) | 79.8 (79.4, 80.1) | 15 (13, 17) | 80.5 (80.1, 80.8) | 18 (16, 20) |
| **Washington** | 74 (73.5, 74.4) | 13 (12, 16) | 74.7 (74.1, 75.1) | 18 (15, 20) | 79.3 (78.8, 79.7) | 18 (16, 20) | 79.5 (79, 79.9) | 24 (21, 26) |
| **Wayne** | 74.5 (74, 74.8) | 11 (10, 13) | 75.4 (75, 75.8) | 14 (12, 16) | 79.3 (78.9, 79.7) | 18 (16, 20) | 80.4 (79.9, 80.8) | 19 (16, 22) |
| **Williams** | 74.8 (74.2, 75.3) | 10 (7, 13) | 75.8 (75.2, 76.3) | 12 (10, 15) | 80.4 (79.8, 80.9) | 12 (9, 15) | 80.7 (79.9, 81.2) | 17 (14, 22) |
| **Wood** | 75.3 (74.9, 75.6) | 7 (6, 9) | 76.4 (76, 76.8) | 10 (8, 11) | 80.2 (79.8, 80.6) | 13 (11, 15) | 81.3 (80.8, 81.8) | 14 (11, 16) |
| **Wyandot** | 74.3 (73.6, 74.8) | 12 (10, 15) | 75.3 (74.6, 76) | 14 (11, 18) | 79.7 (79, 80.3) | 16 (12, 19) | 80.2 (79.5, 80.9) | 20 (16, 24) |
| **OKLAHOMA** |  |  |  |  |  |  |  |  |
| **Adair** | 71.2 (70.5, 71.7) | 26 (24, 30) | 71.3 (70.6, 71.8) | 33 (31, 36) | 78.9 (78.3, 79.3) | 20 (18, 22) | 77.7 (77, 78.2) | 31 (29, 34) |
| **Alfalfa** | 74.3 (73.7, 74.8) | 12 (10, 15) | 74.4 (73.7, 74.9) | 19 (16, 22) | 80 (79.3, 80.6) | 14 (11, 18) | 79.7 (78.9, 80.3) | 23 (19, 26) |
| **Atoka** | 71.5 (70.9, 71.9) | 25 (23, 28) | 71.3 (70.7, 71.7) | 33 (31, 36) | 78.4 (77.9, 78.9) | 21 (20, 24) | 77.3 (76.7, 77.7) | 33 (31, 35) |
| **Beaver** | 74.3 (73.7, 74.9) | 12 (10, 15) | 74.4 (73.8, 74.9) | 19 (16, 22) | 79.5 (79, 80) | 17 (14, 19) | 79.8 (79.1, 80.4) | 22 (19, 26) |
| **Beckham** | 71.7 (71, 72.1) | 24 (22, 27) | 72 (71.3, 72.6) | 30 (27, 33) | 78.3 (77.7, 78.8) | 22 (20, 24) | 77.9 (77.2, 78.5) | 31 (28, 33) |
| **Blaine** | 72.3 (71.6, 72.8) | 21 (19, 24) | 73.6 (72.8, 74.1) | 23 (20, 26) | 79.4 (78.8, 80) | 17 (14, 20) | 79.4 (78.6, 80) | 24 (21, 28) |
| **Bryan** | 72.5 (71.8, 73) | 21 (19, 24) | 72.7 (72.1, 73.2) | 27 (24, 29) | 78.6 (78, 79.1) | 21 (19, 23) | 78 (77.4, 78.6) | 30 (28, 32) |
| **Caddo** | 71.8 (71.3, 72.2) | 24 (22, 26) | 72 (71.4, 72.4) | 30 (28, 32) | 79.1 (78.6, 79.6) | 19 (16, 21) | 78.5 (77.8, 79) | 28 (26, 31) |
| **Canadian** | 74.9 (74.4, 75.3) | 9 (7, 12) | 75.7 (75.3, 76.1) | 12 (11, 15) | 79.8 (79.3, 80.3) | 15 (13, 18) | 79.9 (79.4, 80.4) | 21 (19, 24) |
| **Carter** | 71.7 (71.2, 72.1) | 24 (22, 26) | 71.7 (71.1, 72.1) | 31 (30, 34) | 78.9 (78.4, 79.3) | 20 (18, 22) | 77.7 (77.2, 78.1) | 31 (30, 33) |
| **Cherokee** | 73.1 (72.6, 73.5) | 18 (16, 20) | 73.2 (72.6, 73.7) | 24 (22, 27) | 79.4 (78.9, 79.8) | 18 (15, 20) | 78.6 (78, 79.1) | 28 (26, 30) |
| **Choctaw** | 71.5 (70.9, 71.9) | 25 (23, 28) | 71.3 (70.7, 71.7) | 33 (31, 36) | 78.4 (77.9, 78.9) | 21 (20, 24) | 77.3 (76.7, 77.7) | 33 (31, 35) |
| **Cimarron** | 74.3 (73.7, 74.9) | 12 (10, 15) | 74.4 (73.8, 74.9) | 19 (16, 22) | 79.5 (79, 80) | 17 (14, 19) | 79.8 (79.1, 80.4) | 22 (19, 26) |
| **Cleveland** | 75.5 (75.1, 75.8) | 7 (5, 8) | 75 (74.7, 75.3) | 16 (14, 17) | 79.8 (79.4, 80.1) | 15 (13, 17) | 79.8 (79.4, 80.1) | 22 (21, 24) |
| **Coal** | 71.3 (70.5, 71.8) | 26 (24, 30) | 71.1 (70.4, 71.6) | 34 (31, 38) | 78.5 (77.8, 79) | 21 (19, 24) | 78.1 (77.3, 78.6) | 30 (28, 33) |
| **Comanche** | 72.9 (72.5, 73.2) | 19 (17, 21) | 73.2 (72.8, 73.5) | 24 (23, 26) | 78.5 (78, 78.8) | 21 (20, 23) | 78.4 (77.9, 78.7) | 29 (27, 30) |
| **Cotton** | 71.9 (71.2, 72.4) | 23 (21, 26) | 71.7 (71, 72.2) | 31 (29, 34) | 78.6 (78, 79.2) | 21 (18, 23) | 78.1 (77.4, 78.7) | 30 (28, 32) |
| **Craig** | 72.5 (71.9, 72.9) | 21 (19, 23) | 73.2 (72.5, 73.7) | 24 (22, 28) | 79.1 (78.5, 79.6) | 19 (16, 21) | 79 (78.3, 79.5) | 26 (24, 29) |
| **Creek** | 72.6 (72.1, 73) | 20 (18, 22) | 72.1 (71.6, 72.6) | 29 (28, 31) | 78.4 (78, 78.8) | 21 (20, 23) | 78.2 (77.7, 78.7) | 29 (28, 31) |
| **Custer** | 72.7 (72.1, 73.1) | 20 (18, 23) | 73.3 (72.6, 73.8) | 24 (21, 27) | 79.4 (78.8, 79.9) | 17 (15, 20) | 79.7 (79, 80.3) | 22 (19, 26) |
| **Delaware** | 73.4 (72.9, 73.8) | 16 (14, 19) | 73.3 (72.7, 73.8) | 24 (22, 27) | 79 (78.4, 79.4) | 19 (17, 21) | 79 (78.4, 79.5) | 26 (24, 29) |
| **Dewey** | 72.7 (72.1, 73.1) | 20 (18, 23) | 73.3 (72.6, 73.8) | 24 (21, 27) | 79.4 (78.8, 79.9) | 17 (15, 20) | 79.7 (79, 80.3) | 22 (19, 26) |
| **Ellis** | 74.3 (73.7, 74.9) | 12 (10, 15) | 74.4 (73.8, 74.9) | 19 (16, 22) | 79.5 (79, 80) | 17 (14, 19) | 79.8 (79.1, 80.4) | 22 (19, 26) |
| **Garfield** | 73.1 (72.6, 73.5) | 18 (16, 20) | 73.9 (73.4, 74.3) | 21 (19, 23) | 79.5 (79, 79.9) | 17 (14, 19) | 79.3 (78.8, 79.7) | 25 (23, 27) |
| **Garvin** | 71.8 (71.2, 72.3) | 24 (22, 26) | 71.6 (70.9, 72) | 32 (30, 35) | 78.5 (78, 78.9) | 21 (20, 23) | 78.1 (77.5, 78.5) | 30 (28, 32) |
| **Grady** | 72.6 (72, 73) | 20 (18, 23) | 73.1 (72.5, 73.5) | 25 (23, 28) | 79.3 (78.7, 79.8) | 18 (15, 20) | 78.9 (78.3, 79.5) | 26 (24, 29) |
| **Grant** | 74 (73.3, 74.5) | 13 (11, 17) | 74.7 (73.9, 75.4) | 17 (14, 21) | 80.3 (79.5, 80.9) | 13 (9, 17) | 80.3 (79.4, 81.1) | 20 (15, 24) |
| **Greer** | 72.5 (71.8, 73) | 21 (19, 24) | 72.4 (71.6, 73) | 28 (25, 32) | 78.4 (77.8, 78.9) | 22 (20, 24) | 78.7 (77.9, 79.4) | 27 (24, 30) |
| **Harmon** | 71.7 (71, 72.1) | 24 (22, 27) | 72 (71.3, 72.6) | 30 (27, 33) | 78.3 (77.7, 78.8) | 22 (20, 24) | 77.9 (77.2, 78.5) | 31 (28, 33) |
| **Harper** | 74.3 (73.7, 74.9) | 12 (10, 15) | 74.4 (73.8, 74.9) | 19 (16, 22) | 79.5 (79, 80) | 17 (14, 19) | 79.8 (79.1, 80.4) | 22 (19, 26) |
| **Haskell** | 72.3 (71.6, 72.8) | 22 (20, 25) | 72.3 (71.5, 72.8) | 29 (26, 32) | 78.7 (78.1, 79.3) | 20 (18, 23) | 78.8 (78, 79.4) | 27 (24, 30) |
| **Hughes** | 71 (70.5, 71.4) | 27 (25, 30) | 71.6 (71, 72.1) | 31 (29, 34) | 79 (78.4, 79.5) | 19 (17, 22) | 77.3 (76.7, 77.8) | 32 (31, 35) |
| **Jackson** | 72.9 (72.3, 73.4) | 19 (16, 22) | 73.7 (73, 74.2) | 22 (20, 25) | 79.3 (78.6, 79.8) | 18 (15, 21) | 78.1 (77.4, 78.6) | 30 (28, 32) |
| **Jefferson** | 71.9 (71.2, 72.4) | 23 (21, 26) | 71.7 (71, 72.2) | 31 (29, 34) | 78.6 (78, 79.2) | 21 (18, 23) | 78.1 (77.4, 78.7) | 30 (28, 32) |
| **Johnston** | 71.3 (70.5, 71.8) | 26 (24, 30) | 71.1 (70.4, 71.6) | 34 (31, 38) | 78.5 (77.8, 79) | 21 (19, 24) | 78.1 (77.3, 78.6) | 30 (28, 33) |
| **Kay** | 73.1 (72.6, 73.6) | 18 (16, 20) | 73 (72.4, 73.5) | 25 (23, 28) | 79.2 (78.7, 79.6) | 18 (16, 20) | 78.7 (78.1, 79.1) | 27 (26, 30) |
| **Kingfisher** | 72.3 (71.6, 72.8) | 21 (19, 24) | 73.6 (72.8, 74.1) | 23 (20, 26) | 79.4 (78.8, 80) | 17 (14, 20) | 79.4 (78.6, 80) | 24 (21, 28) |
| **Kiowa** | 72.5 (71.8, 73) | 21 (19, 24) | 72.4 (71.6, 73) | 28 (25, 32) | 78.4 (77.8, 78.9) | 22 (20, 24) | 78.7 (77.9, 79.4) | 27 (24, 30) |
| **Latimer** | 72.3 (71.6, 72.8) | 22 (20, 25) | 72.3 (71.5, 72.8) | 29 (26, 32) | 78.7 (78.1, 79.3) | 20 (18, 23) | 78.8 (78, 79.4) | 27 (24, 30) |
| **Le Flore** | 71.3 (70.7, 71.7) | 26 (24, 28) | 71.3 (70.7, 71.7) | 33 (31, 36) | 78.3 (77.7, 78.7) | 22 (20, 24) | 78.1 (77.5, 78.5) | 30 (28, 32) |
| **Lincoln** | 72.8 (72.2, 73.3) | 19 (17, 22) | 72.4 (71.7, 72.9) | 28 (26, 31) | 78.5 (77.9, 79) | 21 (19, 23) | 78.5 (77.8, 79) | 28 (26, 31) |
| **Logan** | 74 (73.5, 74.5) | 13 (11, 16) | 74.5 (73.9, 75) | 18 (16, 21) | 79.2 (78.6, 79.7) | 18 (16, 21) | 79.8 (79.1, 80.4) | 22 (19, 26) |
| **Love** | 72.8 (72, 73.3) | 20 (17, 23) | 73 (72.3, 73.6) | 25 (22, 29) | 79.6 (78.8, 80.1) | 16 (13, 20) | 78.8 (78.1, 79.4) | 27 (24, 30) |
| **Major** | 74.3 (73.7, 74.8) | 12 (10, 15) | 74.4 (73.7, 74.9) | 19 (16, 22) | 80 (79.3, 80.6) | 14 (11, 18) | 79.7 (78.9, 80.3) | 23 (19, 26) |
| **Marshall** | 72.8 (72, 73.3) | 20 (17, 23) | 73 (72.3, 73.6) | 25 (22, 29) | 79.6 (78.8, 80.1) | 16 (13, 20) | 78.8 (78.1, 79.4) | 27 (24, 30) |
| **Mayes** | 72.7 (72.1, 73.1) | 20 (18, 22) | 73.1 (72.5, 73.6) | 25 (22, 28) | 79.7 (79.1, 80.2) | 16 (13, 19) | 79.7 (79, 80.2) | 23 (20, 26) |
| **McClain** | 73.5 (72.8, 74) | 16 (13, 19) | 74.6 (73.9, 75.2) | 18 (15, 21) | 79.2 (78.6, 79.8) | 18 (15, 21) | 79.8 (79.1, 80.4) | 22 (18, 26) |
| **McCurtain** | 70.1 (69.5, 70.5) | 33 (30, 41) | 70.2 (69.6, 70.7) | 39 (36, 46) | 77.5 (77, 77.9) | 25 (23, 27) | 77.5 (76.9, 78) | 32 (30, 34) |
| **McIntosh** | 71.6 (70.9, 72.1) | 24 (23, 28) | 72.2 (71.5, 72.7) | 29 (27, 32) | 79.6 (78.9, 80.1) | 16 (13, 19) | 79.4 (78.6, 80) | 24 (21, 28) |
| **Murray** | 71.8 (71.2, 72.3) | 24 (22, 26) | 71.6 (70.9, 72) | 32 (30, 35) | 78.5 (78, 78.9) | 21 (20, 23) | 78.1 (77.5, 78.5) | 30 (28, 32) |
| **Muskogee** | 71.4 (70.9, 71.7) | 26 (24, 27) | 71.9 (71.4, 72.2) | 30 (29, 32) | 78.6 (78.2, 79) | 21 (19, 22) | 78.3 (77.8, 78.6) | 29 (28, 31) |
| **Noble** | 74 (73.3, 74.5) | 13 (11, 17) | 74.7 (73.9, 75.4) | 17 (14, 21) | 80.3 (79.5, 80.9) | 13 (9, 17) | 80.3 (79.4, 81.1) | 20 (15, 24) |
| **Nowata** | 72.5 (71.9, 72.9) | 21 (19, 23) | 73.2 (72.5, 73.7) | 24 (22, 28) | 79.1 (78.5, 79.6) | 19 (16, 21) | 79 (78.3, 79.5) | 26 (24, 29) |
| **Okfuskee** | 71 (70.5, 71.4) | 27 (25, 30) | 71.6 (71, 72.1) | 31 (29, 34) | 79 (78.4, 79.5) | 19 (17, 22) | 77.3 (76.7, 77.8) | 32 (31, 35) |
| **Oklahoma** | 71.9 (71.7, 72.1) | 23 (23, 24) | 73.2 (73, 73.4) | 24 (23, 25) | 77.6 (77.4, 77.7) | 25 (24, 25) | 78.8 (78.6, 79) | 27 (26, 28) |
| **Okmulgee** | 71.4 (70.8, 71.7) | 25 (24, 28) | 70.8 (70.2, 71.3) | 35 (33, 39) | 78.2 (77.7, 78.6) | 22 (21, 24) | 78.1 (77.6, 78.6) | 30 (28, 32) |
| **Osage** | 74.1 (73.6, 74.5) | 13 (11, 15) | 74.4 (73.8, 74.8) | 19 (17, 21) | 79.5 (79, 80) | 17 (14, 19) | 80 (79.4, 80.5) | 21 (18, 24) |
| **Ottawa** | 71.8 (71.2, 72.3) | 24 (22, 26) | 72.2 (71.6, 72.7) | 29 (27, 31) | 78.4 (77.8, 78.8) | 22 (20, 24) | 78.4 (77.8, 78.9) | 29 (27, 31) |
| **Pawnee** | 73.3 (72.6, 73.8) | 17 (14, 20) | 74 (73.2, 74.5) | 21 (18, 24) | 79.5 (78.8, 80.1) | 17 (13, 20) | 79 (78.3, 79.7) | 26 (23, 29) |
| **Payne** | 75.2 (74.7, 75.6) | 8 (6, 11) | 76.2 (75.6, 76.6) | 11 (9, 13) | 80.5 (80, 81) | 11 (8, 14) | 79.9 (79.3, 80.3) | 22 (19, 25) |
| **Pittsburg** | 71.7 (71.2, 72.2) | 24 (22, 26) | 72.2 (71.6, 72.6) | 29 (27, 32) | 79.2 (78.7, 79.6) | 18 (16, 20) | 78.4 (77.8, 78.9) | 29 (27, 31) |
| **Pontotoc** | 72.4 (71.8, 72.8) | 21 (19, 24) | 72.9 (72.2, 73.4) | 26 (23, 29) | 78.6 (78, 79) | 21 (19, 23) | 78.2 (77.6, 78.7) | 29 (27, 32) |
| **Pottawatomie** | 72.4 (71.9, 72.8) | 21 (19, 23) | 72.7 (72.3, 73.1) | 27 (25, 29) | 78.4 (77.9, 78.8) | 22 (20, 23) | 77.9 (77.4, 78.3) | 30 (29, 32) |
| **Pushmataha** | 70.1 (69.5, 70.5) | 33 (30, 41) | 70.2 (69.6, 70.7) | 39 (36, 46) | 77.5 (77, 77.9) | 25 (23, 27) | 77.5 (76.9, 78) | 32 (30, 34) |
| **Roger Mills** | 72.7 (72.1, 73.1) | 20 (18, 23) | 73.3 (72.6, 73.8) | 24 (21, 27) | 79.4 (78.8, 79.9) | 17 (15, 20) | 79.7 (79, 80.3) | 22 (19, 26) |
| **Rogers** | 74.5 (74, 74.9) | 11 (9, 13) | 75 (74.6, 75.4) | 16 (14, 18) | 80 (79.5, 80.5) | 14 (11, 17) | 79.2 (78.7, 79.6) | 25 (23, 27) |
| **Seminole** | 70.6 (69.9, 71) | 29 (27, 35) | 71.3 (70.6, 71.9) | 33 (30, 36) | 78.1 (77.5, 78.5) | 23 (21, 25) | 78.2 (77.4, 78.7) | 29 (27, 32) |
| **Sequoyah** | 72 (71.5, 72.4) | 23 (21, 25) | 71.7 (71.2, 72.1) | 31 (29, 33) | 78.9 (78.3, 79.3) | 20 (18, 22) | 78.8 (78.2, 79.3) | 27 (25, 29) |
| **Stephens** | 73.2 (72.6, 73.7) | 17 (15, 20) | 73.4 (72.8, 73.9) | 23 (21, 26) | 79.2 (78.7, 79.6) | 18 (16, 20) | 78.8 (78.2, 79.3) | 27 (25, 29) |
| **Texas** | 74.3 (73.7, 74.9) | 12 (10, 15) | 74.4 (73.8, 74.9) | 19 (16, 22) | 79.5 (79, 80) | 17 (14, 19) | 79.8 (79.1, 80.4) | 22 (19, 26) |
| **Tillman** | 71.9 (71.2, 72.4) | 23 (21, 26) | 71.7 (71, 72.2) | 31 (29, 34) | 78.6 (78, 79.2) | 21 (18, 23) | 78.1 (77.4, 78.7) | 30 (28, 32) |
| **Tulsa** | 72.9 (72.7, 73.1) | 19 (18, 20) | 73.3 (73, 73.4) | 24 (23, 25) | 78.5 (78.3, 78.7) | 21 (20, 22) | 78.2 (78, 78.4) | 29 (28, 30) |
| **Wagoner** | 74.8 (74.2, 75.2) | 10 (8, 12) | 74.8 (74.2, 75.2) | 17 (15, 20) | 79.8 (79.3, 80.3) | 15 (12, 18) | 80 (79.4, 80.5) | 21 (18, 24) |
| **Washington** | 75.1 (74.6, 75.5) | 8 (7, 11) | 75.1 (74.5, 75.5) | 15 (13, 18) | 80 (79.5, 80.5) | 14 (11, 17) | 80.3 (79.8, 80.8) | 19 (16, 22) |
| **Washita** | 71.8 (71.3, 72.2) | 24 (22, 26) | 72 (71.4, 72.4) | 30 (28, 32) | 79.1 (78.6, 79.6) | 19 (16, 21) | 78.5 (77.8, 79) | 28 (26, 31) |
| **Woods** | 74.3 (73.7, 74.8) | 12 (10, 15) | 74.4 (73.7, 74.9) | 19 (16, 22) | 80 (79.3, 80.6) | 14 (11, 18) | 79.7 (78.9, 80.3) | 23 (19, 26) |
| **Woodward** | 73.7 (73, 74.3) | 15 (12, 19) | 74.5 (73.7, 75.2) | 18 (15, 22) | 79.1 (78.4, 79.8) | 19 (15, 22) | 80.4 (79.5, 81.2) | 18 (14, 24) |
| **OREGON** |  |  |  |  |  |  |  |  |
| **Baker** | 74.6 (73.8, 75.1) | 11 (8, 14) | 75.7 (75, 76.3) | 13 (10, 16) | 80.2 (79.5, 80.8) | 13 (9, 17) | 80.2 (79.5, 80.8) | 20 (16, 24) |
| **Benton** | 77.6 (77.1, 78) | -1 (-3, 0) | 78.7 (78.2, 79.2) | 1 (-2, 3) | 81.8 (81.2, 82.2) | 4 (2, 7) | 81.9 (81.3, 82.3) | 11 (9, 14) |
| **Clackamas** | 76 (75.7, 76.3) | 4 (3, 5) | 77.8 (77.5, 78) | 5 (4, 6) | 80.6 (80.4, 80.9) | 10 (9, 12) | 81.4 (81.1, 81.7) | 13 (12, 15) |
| **Clatsop** | 74.7 (74.1, 75.2) | 10 (8, 13) | 76.6 (76, 77.2) | 9 (7, 11) | 79.7 (79.1, 80.3) | 16 (12, 19) | 80.5 (79.9, 81.1) | 18 (15, 22) |
| **Columbia** | 74.9 (74.3, 75.3) | 9 (7, 12) | 75.5 (74.9, 75.9) | 14 (12, 16) | 79.7 (79.1, 80.3) | 16 (13, 19) | 80.8 (80.1, 81.4) | 16 (13, 20) |
| **Coos** | 73.7 (73.2, 74.1) | 15 (13, 17) | 74 (73.5, 74.5) | 20 (18, 23) | 79 (78.6, 79.5) | 19 (17, 21) | 79.3 (78.8, 79.8) | 25 (22, 27) |
| **Crook** | 74 (73.2, 74.5) | 14 (11, 17) | 75.6 (74.9, 76.2) | 13 (11, 16) | 79.5 (78.9, 80.1) | 17 (13, 20) | 80.1 (79.3, 80.8) | 20 (16, 25) |
| **Curry** | 74.5 (73.7, 75) | 11 (9, 15) | 74.8 (74, 75.3) | 17 (14, 21) | 80.1 (79.4, 80.6) | 14 (10, 17) | 80.2 (79.4, 80.8) | 20 (16, 24) |
| **Deschutes** | 76.3 (75.9, 76.7) | 3 (2, 5) | 78.4 (77.9, 78.8) | 2 (0, 4) | 80.6 (80.1, 81) | 10 (8, 13) | 81.7 (81.2, 82.1) | 12 (10, 14) |
| **Douglas** | 73.9 (73.4, 74.3) | 14 (12, 16) | 74.1 (73.6, 74.5) | 20 (18, 22) | 79.5 (79, 79.9) | 17 (15, 19) | 80.7 (80.1, 81.1) | 17 (15, 20) |
| **Gilliam** | 74.1 (73.4, 74.6) | 13 (11, 16) | 75.9 (75.2, 76.5) | 12 (9, 15) | 79.9 (79.3, 80.5) | 15 (11, 18) | 80.8 (80.1, 81.5) | 16 (13, 21) |
| **Grant** | 74.3 (73.6, 74.9) | 12 (9, 16) | 75.9 (75, 76.5) | 12 (9, 16) | 79.7 (78.9, 80.3) | 16 (12, 19) | 80.4 (79.5, 81.1) | 19 (15, 24) |
| **Harney** | 74.3 (73.6, 74.9) | 12 (9, 16) | 75.9 (75, 76.5) | 12 (9, 16) | 79.7 (78.9, 80.3) | 16 (12, 19) | 80.4 (79.5, 81.1) | 19 (15, 24) |
| **Hood River** | 74.6 (73.9, 75.1) | 11 (8, 14) | 76.3 (75.5, 76.9) | 10 (8, 13) | 79.9 (79.3, 80.5) | 14 (11, 18) | 81.5 (80.7, 82.2) | 13 (9, 17) |
| **Jackson** | 75.3 (75, 75.6) | 7 (6, 9) | 76.2 (75.9, 76.6) | 10 (9, 12) | 80.1 (79.8, 80.4) | 13 (12, 15) | 81.5 (81.1, 81.8) | 13 (11, 15) |
| **Jefferson** | 73.9 (73.2, 74.5) | 14 (11, 17) | 74.6 (73.8, 75.2) | 18 (15, 21) | 79.5 (78.8, 80.1) | 17 (13, 20) | 79.2 (78.5, 79.9) | 25 (21, 28) |
| **Josephine** | 74.1 (73.6, 74.5) | 13 (11, 15) | 73.9 (73.4, 74.3) | 21 (19, 23) | 79.5 (79, 79.9) | 17 (15, 19) | 80.3 (79.8, 80.7) | 19 (17, 22) |
| **Klamath** | 73.6 (73.1, 74) | 16 (14, 18) | 74.4 (73.9, 74.9) | 19 (17, 21) | 78.9 (78.5, 79.4) | 19 (18, 21) | 78.7 (78.2, 79.1) | 27 (26, 29) |
| **Lake** | 73.6 (73.1, 74) | 16 (14, 18) | 74.4 (73.9, 74.9) | 19 (17, 21) | 78.9 (78.5, 79.4) | 19 (18, 21) | 78.7 (78.2, 79.1) | 27 (26, 29) |
| **Lane** | 75.4 (75, 75.6) | 7 (6, 9) | 76.1 (75.8, 76.4) | 11 (10, 12) | 80.3 (80, 80.6) | 12 (11, 14) | 81.1 (80.8, 81.4) | 15 (13, 16) |
| **Lincoln** | 74 (73.4, 74.4) | 14 (12, 16) | 75.5 (74.8, 76) | 14 (11, 17) | 80.4 (79.8, 80.9) | 12 (9, 15) | 80.3 (79.7, 80.9) | 19 (16, 22) |
| **Linn** | 74.9 (74.4, 75.3) | 9 (8, 12) | 75.2 (74.8, 75.6) | 15 (13, 17) | 79.7 (79.2, 80.1) | 16 (14, 18) | 80.1 (79.6, 80.5) | 21 (18, 23) |
| **Malheur** | 74.3 (73.7, 74.8) | 12 (10, 15) | 74.2 (73.5, 74.8) | 20 (17, 23) | 79.6 (78.9, 80.1) | 16 (13, 19) | 80 (79.2, 80.6) | 21 (17, 25) |
| **Marion** | 74.7 (74.4, 75) | 10 (9, 12) | 75.8 (75.5, 76.1) | 12 (11, 13) | 80.1 (79.8, 80.3) | 14 (12, 15) | 80.6 (80.3, 80.9) | 17 (16, 20) |
| **Morrow** | 74 (73.6, 74.4) | 13 (12, 16) | 75.6 (75.1, 76) | 13 (11, 16) | 79.9 (79.4, 80.4) | 15 (12, 17) | 80.5 (79.9, 81) | 18 (15, 22) |
| **Multnomah** | 73.9 (73.7, 74.1) | 14 (13, 15) | 75.3 (75.2, 75.5) | 14 (13, 15) | 79.7 (79.5, 79.9) | 16 (15, 17) | 80.2 (80, 80.4) | 20 (19, 21) |
| **Polk** | 76.5 (75.9, 76.9) | 3 (1, 5) | 77.7 (77.1, 78.2) | 5 (3, 7) | 81.2 (80.6, 81.7) | 7 (5, 10) | 82.2 (81.5, 82.7) | 10 (7, 13) |
| **Sherman** | 74.1 (73.4, 74.6) | 13 (11, 16) | 75.9 (75.2, 76.5) | 12 (9, 15) | 79.9 (79.3, 80.5) | 15 (11, 18) | 80.8 (80.1, 81.5) | 16 (13, 21) |
| **Tillamook** | 74.5 (73.8, 75) | 11 (9, 15) | 75.8 (75.1, 76.4) | 12 (10, 16) | 80.4 (79.7, 81) | 12 (8, 16) | 80.8 (80.1, 81.5) | 16 (13, 21) |
| **Umatilla** | 74 (73.6, 74.4) | 13 (12, 16) | 75.6 (75.1, 76) | 13 (11, 16) | 79.9 (79.4, 80.4) | 15 (12, 17) | 80.5 (79.9, 81) | 18 (15, 22) |
| **Union** | 74.9 (74.2, 75.3) | 10 (7, 12) | 76.3 (75.6, 76.9) | 10 (8, 13) | 81 (80.3, 81.7) | 8 (5, 12) | 80.5 (79.7, 81.2) | 18 (14, 22) |
| **Wallowa** | 74.6 (73.8, 75.1) | 11 (8, 14) | 75.7 (75, 76.3) | 13 (10, 16) | 80.2 (79.5, 80.8) | 13 (9, 17) | 80.2 (79.5, 80.8) | 20 (16, 24) |
| **Wasco** | 74.1 (73.4, 74.6) | 13 (11, 16) | 75.9 (75.2, 76.5) | 12 (9, 15) | 79.9 (79.3, 80.5) | 15 (11, 18) | 80.8 (80.1, 81.5) | 16 (13, 21) |
| **Washington** | 77.4 (77.1, 77.6) | -1 (-2, 0) | 78.9 (78.7, 79.2) | 0 (-2, 1) | 81.2 (80.9, 81.5) | 7 (6, 9) | 82.5 (82.2, 82.8) | 8 (7, 9) |
| **Wheeler** | 74 (73.6, 74.4) | 13 (12, 16) | 75.6 (75.1, 76) | 13 (11, 16) | 79.9 (79.4, 80.4) | 15 (12, 17) | 80.5 (79.9, 81) | 18 (15, 22) |
| **Yamhill** | 75.6 (75.1, 75.9) | 6 (5, 8) | 76.6 (76.1, 77) | 9 (8, 11) | 80.4 (79.9, 80.8) | 12 (9, 15) | 80.6 (80.1, 81.1) | 17 (15, 21) |
| **PENNSYLVANIA** |  |  |  |  |  |  |  |  |
| **Adams** | 75.2 (74.8, 75.6) | 8 (6, 10) | 76.1 (75.6, 76.5) | 11 (9, 13) | 80.1 (79.7, 80.5) | 13 (11, 16) | 80.5 (80, 81) | 18 (15, 21) |
| **Allegheny** | 74 (73.8, 74.1) | 14 (13, 14) | 74.7 (74.6, 74.8) | 17 (17, 18) | 79.5 (79.4, 79.6) | 17 (16, 17) | 80.2 (80.1, 80.3) | 20 (19, 21) |
| **Armstrong** | 73.9 (73.4, 74.3) | 14 (12, 16) | 74.8 (74.2, 75.2) | 17 (15, 20) | 79.4 (78.9, 79.7) | 17 (16, 20) | 79.7 (79.2, 80.2) | 22 (20, 25) |
| **Beaver** | 74.1 (73.7, 74.3) | 13 (12, 15) | 74.6 (74.2, 74.8) | 18 (17, 20) | 80 (79.7, 80.3) | 14 (13, 16) | 80.4 (80, 80.7) | 19 (17, 21) |
| **Bedford** | 74.2 (73.7, 74.7) | 13 (11, 15) | 75.2 (74.6, 75.8) | 15 (12, 18) | 80.4 (79.8, 80.9) | 12 (9, 15) | 80.6 (80, 81.1) | 18 (15, 21) |
| **Berks** | 74.7 (74.4, 74.9) | 10 (9, 12) | 76 (75.8, 76.2) | 11 (10, 12) | 80.1 (79.8, 80.3) | 14 (12, 15) | 81.5 (81.3, 81.8) | 12 (11, 14) |
| **Blair** | 73.4 (73, 73.7) | 16 (15, 18) | 73.4 (73, 73.8) | 23 (22, 25) | 79.8 (79.4, 80.1) | 15 (13, 17) | 80 (79.6, 80.4) | 21 (19, 23) |
| **Bradford** | 74.4 (73.9, 74.9) | 12 (9, 14) | 75.2 (74.7, 75.7) | 15 (12, 17) | 80.5 (80, 81) | 11 (8, 14) | 80.6 (80, 81.1) | 18 (15, 21) |
| **Bucks** | 75.6 (75.4, 75.8) | 6 (5, 7) | 77.1 (76.8, 77.2) | 7 (7, 8) | 80.1 (79.9, 80.3) | 13 (12, 15) | 81.4 (81.1, 81.5) | 13 (13, 15) |
| **Butler** | 75.4 (75, 75.7) | 7 (6, 9) | 75.9 (75.6, 76.3) | 12 (10, 13) | 80.4 (80, 80.7) | 12 (10, 14) | 80.8 (80.4, 81.1) | 16 (15, 19) |
| **Cambria** | 73.7 (73.3, 74) | 15 (14, 17) | 74.3 (74, 74.6) | 19 (18, 21) | 79.9 (79.6, 80.2) | 15 (13, 16) | 80.1 (79.7, 80.4) | 20 (18, 23) |
| **Cameron** | 74.5 (73.8, 75) | 11 (9, 15) | 75.2 (74.4, 75.9) | 15 (12, 19) | 79.9 (79.3, 80.5) | 14 (11, 18) | 80 (79.3, 80.6) | 21 (17, 25) |
| **Carbon** | 73.6 (73, 74) | 15 (14, 18) | 73.6 (73, 74) | 22 (21, 25) | 79.4 (78.9, 79.8) | 17 (15, 19) | 79.5 (79, 79.9) | 24 (22, 26) |
| **Centre** | 76.8 (76.3, 77.1) | 1 (0, 3) | 77.8 (77.3, 78.2) | 5 (3, 7) | 80.8 (80.4, 81.2) | 9 (7, 12) | 81.3 (80.9, 81.7) | 14 (12, 16) |
| **Chester** | 76.5 (76.2, 76.7) | 3 (2, 3) | 77.9 (77.6, 78.1) | 5 (4, 5) | 80.7 (80.5, 81) | 10 (9, 11) | 82.2 (82, 82.5) | 9 (8, 11) |
| **Clarion** | 74.2 (73.6, 74.6) | 13 (11, 15) | 74.9 (74.4, 75.5) | 16 (14, 19) | 79.6 (79.1, 80.1) | 16 (13, 19) | 80.3 (79.7, 80.9) | 19 (16, 23) |
| **Clearfield** | 74.2 (73.7, 74.5) | 13 (11, 15) | 74.8 (74.3, 75.2) | 17 (15, 19) | 79.9 (79.5, 80.3) | 15 (12, 17) | 80.8 (80.3, 81.3) | 16 (14, 19) |
| **Clinton** | 74 (73.4, 74.4) | 14 (12, 17) | 74.3 (73.7, 74.9) | 19 (17, 22) | 79.5 (78.9, 80) | 17 (14, 19) | 79.8 (79.1, 80.3) | 22 (19, 25) |
| **Columbia** | 74.5 (74, 74.9) | 11 (9, 13) | 74.9 (74.3, 75.3) | 17 (14, 19) | 80.6 (80.1, 81.1) | 10 (8, 13) | 80.8 (80.2, 81.2) | 16 (14, 20) |
| **Crawford** | 74.2 (73.7, 74.6) | 13 (11, 15) | 74.8 (74.2, 75.2) | 17 (15, 19) | 79.3 (78.9, 79.7) | 18 (16, 20) | 80.1 (79.6, 80.5) | 21 (18, 23) |
| **Cumberland** | 76.2 (75.9, 76.4) | 4 (3, 5) | 77 (76.7, 77.3) | 8 (7, 9) | 80.8 (80.5, 81.1) | 9 (8, 11) | 81.3 (81, 81.6) | 13 (12, 15) |
| **Dauphin** | 73.7 (73.4, 73.9) | 15 (14, 16) | 74.8 (74.6, 75.1) | 17 (16, 18) | 79.4 (79.1, 79.6) | 17 (16, 19) | 80.1 (79.8, 80.4) | 20 (19, 22) |
| **Delaware** | 74.2 (74, 74.3) | 13 (12, 14) | 74.8 (74.6, 74.9) | 17 (16, 18) | 79.5 (79.4, 79.7) | 17 (16, 18) | 80 (79.8, 80.2) | 21 (20, 22) |
| **Elk** | 73.8 (73.2, 74.3) | 14 (12, 17) | 74.9 (74.3, 75.3) | 17 (14, 19) | 79.9 (79.3, 80.4) | 15 (12, 18) | 80.7 (80, 81.3) | 17 (14, 21) |
| **Erie** | 74 (73.8, 74.3) | 13 (12, 15) | 75.3 (75, 75.6) | 14 (13, 16) | 79.3 (79, 79.6) | 18 (16, 19) | 80.3 (80, 80.6) | 19 (17, 21) |
| **Fayette** | 72.7 (72.3, 73) | 20 (18, 22) | 73.4 (73, 73.7) | 23 (22, 25) | 79 (78.7, 79.3) | 19 (18, 21) | 80 (79.6, 80.4) | 21 (19, 23) |
| **Forest** | 73.8 (73.2, 74.3) | 14 (12, 17) | 74.9 (74.3, 75.3) | 17 (14, 19) | 79.9 (79.3, 80.4) | 15 (12, 18) | 80.7 (80, 81.3) | 17 (14, 21) |
| **Franklin** | 75 (74.6, 75.3) | 9 (7, 11) | 76.1 (75.7, 76.4) | 11 (10, 13) | 80.6 (80.2, 81) | 10 (8, 13) | 81.4 (81, 81.8) | 13 (11, 15) |
| **Fulton** | 73.7 (72.9, 74.3) | 15 (12, 19) | 74.6 (73.8, 75.3) | 18 (14, 22) | 79.9 (79.2, 80.6) | 15 (11, 18) | 80.4 (79.5, 81.1) | 19 (15, 24) |
| **Greene** | 73.3 (72.7, 73.7) | 17 (15, 20) | 74.7 (74, 75.2) | 17 (15, 20) | 78.5 (77.9, 78.9) | 21 (19, 23) | 79.8 (79.1, 80.3) | 22 (19, 26) |
| **Huntingdon** | 73.9 (73.3, 74.3) | 14 (12, 17) | 74.6 (74.1, 75.1) | 18 (15, 20) | 80.3 (79.7, 80.8) | 12 (9, 16) | 79.8 (79.2, 80.4) | 22 (19, 25) |
| **Indiana** | 74.5 (74, 74.8) | 11 (10, 13) | 76.2 (75.7, 76.6) | 11 (9, 13) | 79.7 (79.3, 80.1) | 16 (13, 18) | 81 (80.4, 81.4) | 15 (13, 19) |
| **Jefferson** | 74.6 (74, 75.1) | 11 (9, 13) | 74.9 (74.3, 75.4) | 16 (14, 19) | 79.2 (78.6, 79.6) | 18 (16, 21) | 80.3 (79.7, 80.8) | 19 (16, 23) |
| **Juniata** | 73.7 (73, 74.2) | 15 (13, 18) | 74.6 (73.8, 75.1) | 18 (15, 22) | 79.5 (78.8, 80.1) | 17 (13, 20) | 80.8 (79.9, 81.6) | 16 (12, 21) |
| **Lackawanna** | 73.5 (73.2, 73.7) | 16 (15, 17) | 74 (73.6, 74.3) | 21 (19, 22) | 79.6 (79.3, 79.9) | 16 (15, 18) | 80.3 (79.9, 80.5) | 20 (18, 22) |
| **Lancaster** | 76.1 (75.8, 76.3) | 4 (3, 5) | 76.8 (76.5, 77) | 8 (8, 9) | 80.9 (80.6, 81.1) | 9 (8, 10) | 81.4 (81.1, 81.6) | 13 (12, 15) |
| **Lawrence** | 73.3 (72.9, 73.6) | 17 (15, 19) | 74.5 (74.1, 74.9) | 18 (16, 20) | 80.2 (79.8, 80.5) | 13 (11, 15) | 80.6 (80.1, 80.9) | 18 (16, 20) |
| **Lebanon** | 74.9 (74.4, 75.2) | 10 (8, 12) | 75.7 (75.3, 76) | 13 (11, 15) | 80.6 (80.1, 80.9) | 11 (9, 13) | 80.9 (80.5, 81.3) | 16 (14, 18) |
| **Lehigh** | 75 (74.7, 75.2) | 9 (8, 10) | 76.2 (75.9, 76.4) | 11 (10, 12) | 80.5 (80.2, 80.7) | 11 (10, 13) | 81.8 (81.5, 82) | 11 (10, 13) |
| **Luzerne** | 72.7 (72.4, 72.9) | 20 (19, 21) | 73.3 (73, 73.5) | 24 (23, 25) | 79.4 (79.1, 79.6) | 17 (16, 19) | 79.8 (79.5, 80.1) | 22 (21, 24) |
| **Lycoming** | 74.7 (74.3, 75) | 10 (9, 12) | 74.9 (74.5, 75.2) | 16 (15, 18) | 79.6 (79.2, 80) | 16 (14, 18) | 80 (79.6, 80.4) | 21 (19, 23) |
| **McKean** | 74.2 (73.7, 74.6) | 13 (11, 15) | 74.3 (73.7, 74.7) | 19 (17, 22) | 79.2 (78.6, 79.6) | 18 (16, 21) | 80 (79.4, 80.5) | 21 (18, 24) |
| **Mercer** | 74.2 (73.7, 74.5) | 13 (11, 15) | 74.7 (74.2, 75) | 18 (16, 20) | 79.5 (79.1, 79.8) | 17 (15, 19) | 80.2 (79.7, 80.5) | 20 (18, 23) |
| **Mifflin** | 74.1 (73.6, 74.6) | 13 (11, 16) | 74.7 (74.1, 75.2) | 17 (15, 20) | 79.6 (79, 80.1) | 16 (14, 19) | 80.3 (79.6, 80.9) | 19 (16, 23) |
| **Monroe** | 74.8 (74.4, 75.1) | 10 (8, 12) | 75.6 (75.2, 75.9) | 13 (12, 15) | 80 (79.6, 80.3) | 14 (12, 16) | 80.4 (80, 80.8) | 18 (16, 21) |
| **Montgomery** | 76.4 (76.3, 76.6) | 3 (2, 3) | 77.7 (77.5, 77.8) | 5 (5, 6) | 81.2 (81, 81.3) | 7 (7, 8) | 82 (81.8, 82.2) | 10 (9, 11) |
| **Montour** | 74.6 (73.9, 75.1) | 11 (9, 14) | 75.4 (74.6, 75.9) | 14 (12, 18) | 79.8 (79.2, 80.4) | 15 (12, 18) | 80.5 (79.7, 81.1) | 18 (15, 22) |
| **Northampton** | 75.7 (75.4, 75.9) | 6 (5, 7) | 76.5 (76.2, 76.7) | 9 (9, 11) | 81 (80.7, 81.2) | 8 (7, 10) | 81.8 (81.5, 82.1) | 11 (10, 13) |
| **Northumberlnd** | 73.4 (73, 73.7) | 16 (15, 18) | 74.3 (73.8, 74.6) | 19 (18, 21) | 79.1 (78.7, 79.5) | 19 (17, 20) | 80.2 (79.8, 80.6) | 20 (17, 22) |
| **Perry** | 73.9 (73.3, 74.4) | 14 (12, 17) | 74.9 (74.3, 75.4) | 16 (14, 19) | 79.4 (78.8, 79.9) | 17 (15, 20) | 79.8 (79.2, 80.4) | 22 (19, 25) |
| **Philadelphia** | 68.8 (68.7, 68.9) | 47 (47, 48) | 69.2 (69.1, 69.3) | 51 (51, 51) | 76.2 (76.1, 76.3) | 30 (30, 31) | 77.6 (77.5, 77.7) | 32 (31, 32) |
| **Pike** | 75.2 (74.5, 75.7) | 8 (6, 11) | 77.3 (76.7, 77.9) | 6 (4, 9) | 80.5 (79.9, 81) | 11 (8, 14) | 82.3 (81.6, 83) | 9 (6, 12) |
| **Potter** | 74.5 (73.8, 75) | 11 (9, 15) | 75.2 (74.4, 75.9) | 15 (12, 19) | 79.9 (79.3, 80.5) | 14 (11, 18) | 80 (79.3, 80.6) | 21 (17, 25) |
| **Schuylkill** | 72.8 (72.4, 73.1) | 19 (18, 21) | 73.2 (72.8, 73.5) | 25 (23, 26) | 79.1 (78.8, 79.4) | 19 (17, 20) | 79.5 (79.1, 79.8) | 24 (22, 26) |
| **Snyder** | 74.9 (74.2, 75.4) | 10 (7, 12) | 75.1 (74.4, 75.6) | 15 (13, 19) | 79.9 (79.3, 80.5) | 15 (11, 18) | 81.2 (80.4, 81.9) | 14 (11, 19) |
| **Somerset** | 73.8 (73.3, 74.2) | 14 (13, 17) | 74.6 (74.2, 75) | 18 (16, 20) | 80.1 (79.6, 80.4) | 14 (11, 16) | 80.8 (80.3, 81.3) | 16 (14, 19) |
| **Sullivan** | 74 (73.4, 74.5) | 13 (11, 16) | 74.8 (74.1, 75.3) | 17 (14, 20) | 79.6 (79, 80.1) | 16 (14, 19) | 80.7 (80, 81.3) | 17 (14, 21) |
| **Susquehanna** | 73.9 (73.3, 74.4) | 14 (12, 17) | 74.7 (74, 75.2) | 18 (15, 20) | 79.9 (79.3, 80.3) | 15 (12, 18) | 80.1 (79.5, 80.7) | 21 (17, 24) |
| **Tioga** | 74.4 (73.8, 74.8) | 12 (10, 15) | 75.8 (75.2, 76.3) | 12 (10, 15) | 79.4 (78.9, 79.9) | 17 (15, 20) | 80.4 (79.8, 80.9) | 19 (16, 22) |
| **Union** | 74.8 (74.2, 75.3) | 10 (8, 13) | 76 (75.4, 76.6) | 11 (9, 14) | 79.7 (79.2, 80.3) | 15 (12, 18) | 80.5 (79.9, 81.2) | 18 (14, 22) |
| **Venango** | 73.5 (73, 74) | 16 (14, 18) | 74.5 (73.9, 75) | 18 (16, 21) | 79.5 (79, 79.9) | 17 (14, 19) | 80.1 (79.5, 80.6) | 21 (17, 24) |
| **Warren** | 74.3 (73.7, 74.7) | 12 (10, 15) | 75.4 (74.7, 75.9) | 14 (12, 17) | 79.8 (79.3, 80.3) | 15 (12, 18) | 79.9 (79.3, 80.4) | 21 (19, 25) |
| **Washington** | 74.4 (74.1, 74.6) | 12 (11, 13) | 75.1 (74.8, 75.4) | 15 (14, 17) | 79.7 (79.4, 80) | 16 (14, 17) | 80.6 (80.2, 80.8) | 18 (16, 20) |
| **Wayne** | 74 (73.4, 74.4) | 13 (12, 16) | 74.3 (73.7, 74.7) | 19 (17, 22) | 79.6 (79, 80) | 16 (14, 19) | 80 (79.4, 80.5) | 21 (18, 24) |
| **Westmoreland** | 74.9 (74.7, 75.1) | 9 (8, 11) | 75.8 (75.5, 76) | 12 (11, 13) | 80.1 (79.8, 80.3) | 14 (12, 15) | 80.9 (80.6, 81.1) | 16 (15, 17) |
| **Wyoming** | 74 (73.4, 74.5) | 13 (11, 16) | 74.8 (74.1, 75.3) | 17 (14, 20) | 79.6 (79, 80.1) | 16 (14, 19) | 80.7 (80, 81.3) | 17 (14, 21) |
| **York** | 75.3 (75.1, 75.6) | 7 (6, 9) | 76.1 (75.9, 76.4) | 11 (10, 12) | 80.6 (80.4, 80.9) | 10 (9, 12) | 81.6 (81.3, 81.9) | 12 (11, 14) |
| **RHODE ISLAND** |  |  |  |  |  |  |  |  |
| **Bristol** | 75.9 (75.3, 76.3) | 5 (3, 7) | 77 (76.4, 77.4) | 8 (6, 10) | 82 (81.5, 82.6) | 3 (1, 6) | 82.6 (82, 83.2) | 8 (5, 10) |
| **Kent** | 75.1 (74.7, 75.4) | 8 (7, 10) | 75.9 (75.5, 76.2) | 12 (11, 13) | 80.3 (79.9, 80.5) | 13 (11, 15) | 81 (80.6, 81.3) | 15 (14, 17) |
| **Newport** | 76.8 (76.3, 77.2) | 1 (0, 3) | 77.9 (77.4, 78.3) | 4 (3, 6) | 81.6 (81.2, 82.1) | 5 (3, 7) | 81.7 (81.2, 82.2) | 12 (10, 14) |
| **Providence** | 74.8 (74.6, 74.9) | 10 (9, 11) | 75.7 (75.5, 75.9) | 13 (12, 14) | 80.5 (80.2, 80.6) | 11 (10, 13) | 81.4 (81.1, 81.6) | 13 (12, 15) |
| **Washington** | 76.7 (76.3, 77.1) | 2 (0, 3) | 78.1 (77.7, 78.5) | 4 (2, 5) | 81.2 (80.8, 81.7) | 7 (5, 9) | 82 (81.6, 82.5) | 10 (8, 12) |
| **SOUTH CAROLINA** |  |  |  |  |  |  |  |  |
| **Abbeville** | 71.6 (71.1, 72) | 25 (23, 27) | 72 (71.4, 72.4) | 30 (28, 32) | 77.7 (77.3, 78.1) | 24 (23, 26) | 78.1 (77.6, 78.6) | 30 (28, 31) |
| **Aiken** | 72.3 (72, 72.6) | 22 (21, 23) | 73.6 (73.2, 73.8) | 23 (21, 24) | 78.3 (78, 78.5) | 22 (21, 23) | 79.2 (78.8, 79.5) | 25 (24, 27) |
| **Allendale** | 68 (67.3, 68.5) | 51 (49, 51) | 68.7 (67.9, 69.2) | 51 (51, 51) | 75.4 (74.7, 75.8) | 35 (32, 39) | 76.2 (75.5, 76.6) | 37 (35, 41) |
| **Anderson** | 71.2 (70.9, 71.5) | 26 (25, 28) | 71.8 (71.5, 72.1) | 31 (29, 32) | 78.7 (78.4, 79) | 20 (19, 22) | 79 (78.6, 79.3) | 26 (25, 28) |
| **Bamberg** | 68 (67.3, 68.5) | 51 (49, 51) | 68.7 (67.9, 69.2) | 51 (51, 51) | 75.4 (74.7, 75.8) | 35 (32, 39) | 76.2 (75.5, 76.6) | 37 (35, 41) |
| **Barnwell** | 70.1 (69.4, 70.5) | 33 (30, 43) | 70.4 (69.8, 70.9) | 37 (35, 44) | 77 (76.4, 77.4) | 27 (25, 29) | 76.5 (75.8, 76.9) | 36 (34, 39) |
| **Beaufort** | 76.2 (75.9, 76.5) | 3 (2, 5) | 78 (77.6, 78.3) | 4 (3, 6) | 80.6 (80.2, 80.9) | 11 (9, 13) | 83.3 (82.8, 83.7) | 5 (3, 7) |
| **Berkeley** | 72.2 (71.8, 72.4) | 22 (21, 24) | 73.9 (73.5, 74.2) | 21 (20, 23) | 78.2 (77.9, 78.5) | 22 (21, 24) | 79.2 (78.7, 79.5) | 25 (24, 27) |
| **Calhoun** | 70.5 (69.8, 70.9) | 30 (28, 36) | 71.9 (71.2, 72.3) | 30 (28, 33) | 77.7 (77.1, 78.2) | 24 (22, 26) | 78.1 (77.5, 78.6) | 30 (28, 32) |
| **Charleston** | 72.3 (72.1, 72.5) | 22 (21, 23) | 73.8 (73.5, 74) | 22 (21, 23) | 78.9 (78.7, 79.1) | 20 (19, 21) | 80 (79.8, 80.2) | 21 (20, 22) |
| **Cherokee** | 70.6 (70.2, 71) | 29 (27, 32) | 70.1 (69.6, 70.4) | 40 (37, 47) | 77.6 (77.1, 78) | 25 (23, 26) | 76.7 (76.2, 77.1) | 35 (33, 37) |
| **Chester** | 69.6 (69, 70) | 40 (34, 46) | 70.2 (69.5, 70.6) | 39 (36, 48) | 76.4 (75.9, 76.8) | 29 (27, 32) | 77.9 (77.2, 78.3) | 31 (29, 33) |
| **Chesterfield** | 68.7 (68.2, 69.1) | 48 (45, 51) | 69.7 (69.3, 70.1) | 44 (40, 51) | 76 (75.6, 76.4) | 31 (29, 34) | 76.8 (76.3, 77.2) | 34 (33, 36) |
| **Clarendon** | 69.3 (68.7, 69.7) | 44 (38, 48) | 70.8 (70.2, 71.2) | 35 (33, 39) | 76.1 (75.6, 76.4) | 31 (29, 34) | 77.4 (76.8, 77.9) | 32 (30, 34) |
| **Colleton** | 69.7 (69.2, 70.1) | 37 (33, 45) | 69.7 (69.2, 70.1) | 45 (40, 51) | 76.7 (76.2, 77.1) | 28 (26, 30) | 77.4 (76.8, 77.8) | 32 (31, 34) |
| **Darlington** | 69.5 (69, 69.8) | 42 (37, 46) | 69.9 (69.4, 70.3) | 42 (38, 49) | 75.8 (75.4, 76.1) | 32 (31, 35) | 76.8 (76.3, 77.1) | 35 (33, 37) |
| **Dillon** | 67.5 (66.9, 67.9) | 51 (51, 51) | 67.9 (67.3, 68.4) | 51 (51, 51) | 74.9 (74.4, 75.2) | 38 (36, 41) | 75.4 (74.8, 75.8) | 42 (39, 46) |
| **Dorchester** | 72.2 (71.8, 72.5) | 22 (21, 24) | 74.1 (73.7, 74.4) | 20 (19, 22) | 78 (77.6, 78.3) | 23 (22, 25) | 79.4 (78.9, 79.7) | 25 (23, 26) |
| **Edgefield** | 70.8 (70.2, 71.2) | 28 (26, 32) | 72 (71.4, 72.4) | 30 (28, 32) | 77.4 (76.8, 77.8) | 25 (24, 27) | 77.6 (76.9, 78) | 32 (30, 34) |
| **Fairfield** | 68.7 (68.1, 69.1) | 48 (45, 51) | 69 (68.3, 69.5) | 51 (49, 51) | 75.3 (74.7, 75.6) | 36 (33, 39) | 76.8 (76.1, 77.2) | 35 (33, 38) |
| **Florence** | 69 (68.7, 69.3) | 46 (44, 48) | 70.7 (70.4, 71) | 35 (34, 37) | 76.6 (76.3, 76.9) | 28 (27, 30) | 77.1 (76.8, 77.4) | 33 (32, 35) |
| **Georgetown** | 71 (70.5, 71.4) | 27 (25, 30) | 73.7 (73.2, 74.1) | 22 (20, 24) | 78.1 (77.7, 78.5) | 23 (21, 24) | 79 (78.5, 79.3) | 26 (25, 28) |
| **Greenville** | 73.3 (73, 73.5) | 17 (16, 18) | 74.1 (73.9, 74.3) | 20 (19, 21) | 78.9 (78.6, 79.1) | 20 (19, 21) | 79.9 (79.6, 80.1) | 22 (21, 23) |
| **Greenwood** | 71.2 (70.7, 71.5) | 26 (25, 28) | 72.5 (72, 72.8) | 28 (26, 30) | 77.9 (77.5, 78.3) | 23 (22, 25) | 78.5 (78, 78.8) | 28 (27, 30) |
| **Hampton** | 69.7 (69.1, 70.2) | 38 (32, 46) | 69.9 (69.3, 70.4) | 42 (38, 51) | 76.4 (75.8, 76.8) | 29 (28, 32) | 76.7 (76.1, 77.2) | 35 (33, 38) |
| **Horry** | 72.2 (71.9, 72.5) | 22 (21, 23) | 73.7 (73.4, 74) | 22 (20, 23) | 79.1 (78.8, 79.4) | 19 (17, 20) | 79.8 (79.4, 80.1) | 22 (21, 24) |
| **Jasper** | 70 (69.3, 70.5) | 34 (30, 43) | 71.1 (70.4, 71.6) | 34 (32, 37) | 76.7 (76.1, 77.1) | 28 (26, 31) | 77 (76.4, 77.5) | 34 (32, 36) |
| **Kershaw** | 71.5 (71, 71.9) | 25 (23, 27) | 73 (72.5, 73.4) | 25 (23, 28) | 78.1 (77.6, 78.4) | 23 (21, 25) | 78.9 (78.4, 79.3) | 27 (25, 29) |
| **Lancaster** | 70.4 (69.9, 70.7) | 30 (29, 35) | 72.9 (72.3, 73.2) | 26 (24, 29) | 77.8 (77.2, 78.1) | 24 (23, 26) | 79.1 (78.5, 79.5) | 26 (24, 28) |
| **Laurens** | 71.2 (70.7, 71.5) | 26 (25, 29) | 71.2 (70.7, 71.5) | 33 (32, 36) | 77.5 (77.1, 77.9) | 25 (23, 26) | 77.7 (77.3, 78.1) | 31 (30, 33) |
| **Lee** | 67.6 (66.9, 68.1) | 51 (51, 51) | 68.3 (67.6, 68.7) | 51 (51, 51) | 75.5 (74.9, 75.9) | 34 (32, 39) | 75.4 (74.7, 75.9) | 42 (39, 47) |
| **Lexington** | 73.3 (73, 73.5) | 17 (16, 18) | 74.5 (74.1, 74.8) | 18 (17, 20) | 79.3 (79, 79.7) | 18 (16, 19) | 80 (79.6, 80.3) | 21 (19, 23) |
| **Marion** | 67.4 (66.8, 67.8) | 51 (51, 51) | 68.8 (68.2, 69.3) | 51 (51, 51) | 75.4 (74.9, 75.7) | 35 (33, 39) | 76 (75.4, 76.4) | 38 (36, 42) |
| **Marlboro** | 66.1 (65.5, 66.5) | 51 (51, 51) | 68.1 (67.4, 68.5) | 51 (51, 51) | 74.2 (73.7, 74.5) | 42 (41, 45) | 75.3 (74.6, 75.7) | 43 (40, 47) |
| **McCormick** | 71.6 (71.1, 72) | 25 (23, 27) | 72 (71.4, 72.4) | 30 (28, 32) | 77.7 (77.3, 78.1) | 24 (23, 26) | 78.1 (77.6, 78.6) | 30 (28, 31) |
| **Newberry** | 71.3 (70.8, 71.7) | 26 (24, 28) | 71.7 (71.2, 72.1) | 31 (29, 33) | 78.4 (77.8, 78.8) | 22 (20, 24) | 78.5 (78, 78.9) | 28 (26, 30) |
| **Oconee** | 73.6 (73, 74) | 15 (14, 18) | 74 (73.4, 74.4) | 21 (19, 23) | 79.9 (79.3, 80.3) | 15 (12, 18) | 79.9 (79.4, 80.4) | 21 (19, 24) |
| **Orangeburg** | 68.7 (68.3, 69.1) | 48 (46, 51) | 69.4 (68.9, 69.7) | 50 (45, 51) | 76.7 (76.2, 77) | 28 (27, 30) | 76.9 (76.5, 77.3) | 34 (32, 36) |
| **Pickens** | 73.6 (73.2, 74) | 15 (14, 17) | 74.2 (73.8, 74.6) | 20 (18, 22) | 78.9 (78.5, 79.2) | 20 (18, 21) | 79.7 (79.2, 80.1) | 23 (20, 25) |
| **Richland** | 71.5 (71.3, 71.7) | 25 (24, 26) | 73.1 (72.9, 73.3) | 25 (24, 26) | 77.6 (77.4, 77.8) | 24 (24, 25) | 78.8 (78.5, 79) | 27 (26, 28) |
| **Saluda** | 71.3 (70.7, 71.8) | 26 (24, 29) | 72.6 (72, 73.1) | 27 (25, 30) | 77.8 (77.2, 78.3) | 24 (22, 26) | 78.2 (77.6, 78.7) | 29 (27, 32) |
| **Spartanburg** | 71.4 (71.1, 71.6) | 25 (25, 27) | 72 (71.8, 72.3) | 30 (29, 31) | 77.6 (77.3, 77.8) | 25 (24, 26) | 78.4 (78.1, 78.6) | 29 (28, 30) |
| **Sumter** | 70.7 (70.4, 71) | 28 (27, 31) | 71.6 (71.2, 71.9) | 32 (30, 33) | 77.3 (76.9, 77.6) | 26 (25, 27) | 78.5 (78.1, 78.9) | 28 (27, 30) |
| **Union** | 70 (69.5, 70.4) | 34 (30, 42) | 69.3 (68.8, 69.7) | 51 (45, 51) | 76.7 (76.2, 77.1) | 28 (27, 30) | 77.1 (76.5, 77.5) | 33 (32, 36) |
| **Williamsburg** | 67.5 (66.9, 68) | 51 (51, 51) | 67.8 (67.2, 68.3) | 51 (51, 51) | 76.1 (75.6, 76.6) | 31 (29, 34) | 75.6 (75, 76.1) | 41 (38, 45) |
| **York** | 72.8 (72.4, 73) | 20 (18, 21) | 74 (73.7, 74.3) | 20 (19, 22) | 78.6 (78.3, 78.9) | 21 (20, 22) | 79.2 (78.9, 79.5) | 25 (24, 27) |
| **SOUTH DAKOTA** |  |  |  |  |  |  |  |  |
| **Aurora** | 74.2 (73.4, 74.7) | 13 (10, 16) | 74.9 (74.1, 75.6) | 16 (13, 20) | 80.7 (80, 81.4) | 10 (6, 14) | 81.2 (80.4, 81.9) | 14 (11, 19) |
| **Beadle** | 74.6 (73.9, 75.1) | 11 (9, 14) | 75.8 (75, 76.4) | 12 (10, 16) | 81 (80.4, 81.6) | 8 (5, 12) | 81.4 (80.6, 82.1) | 13 (10, 17) |
| **Bennett** | 70.9 (70.1, 71.4) | 28 (25, 33) | 71 (70.1, 71.6) | 34 (32, 40) | 79.2 (78.5, 79.7) | 18 (16, 21) | 78.3 (77.5, 78.8) | 29 (27, 32) |
| **Bon Homme** | 74.5 (73.8, 75) | 11 (9, 14) | 75 (74.2, 75.6) | 16 (13, 19) | 80.3 (79.5, 80.9) | 13 (9, 17) | 80.9 (80, 81.6) | 16 (12, 21) |
| **Brookings** | 76.1 (75.4, 76.6) | 4 (2, 7) | 77 (76.3, 77.7) | 8 (5, 10) | 81.4 (80.7, 82.2) | 6 (3, 10) | 81.8 (81, 82.6) | 11 (8, 15) |
| **Brown** | 75.5 (75, 76) | 6 (4, 9) | 76.3 (75.7, 76.8) | 10 (8, 13) | 80.7 (80.1, 81.1) | 10 (8, 14) | 82.4 (81.6, 83) | 9 (6, 12) |
| **Brule** | 74.5 (73.7, 75) | 11 (9, 15) | 75 (74.2, 75.6) | 16 (13, 20) | 80.9 (80.1, 81.6) | 9 (5, 13) | 80.8 (80, 81.5) | 16 (13, 21) |
| **Buffalo** | 74.6 (73.9, 75.1) | 11 (9, 14) | 75.8 (75, 76.4) | 12 (10, 16) | 81 (80.4, 81.6) | 8 (5, 12) | 81.4 (80.6, 82.1) | 13 (10, 17) |
| **Butte** | 74.5 (73.9, 74.9) | 11 (9, 14) | 74.8 (74.1, 75.3) | 17 (14, 20) | 80.7 (80.1, 81.3) | 10 (7, 13) | 80.5 (79.9, 81.1) | 18 (15, 22) |
| **Campbell** | 74.2 (73.4, 74.7) | 13 (10, 16) | 74.1 (73.3, 74.8) | 20 (17, 24) | 80.2 (79.5, 80.8) | 13 (9, 17) | 80.3 (79.5, 81) | 19 (15, 24) |
| **Charles Mix** | 74.5 (73.8, 75) | 11 (9, 14) | 75 (74.2, 75.6) | 16 (13, 19) | 80.3 (79.5, 80.9) | 13 (9, 17) | 80.9 (80, 81.6) | 16 (12, 21) |
| **Clark** | 74.9 (74.1, 75.4) | 10 (7, 13) | 74.9 (74.1, 75.5) | 16 (13, 20) | 81.1 (80.3, 81.7) | 8 (5, 12) | 80.6 (79.7, 81.2) | 18 (14, 22) |
| **Clay** | 75.6 (75, 76.1) | 6 (4, 9) | 77.4 (76.7, 78) | 6 (4, 9) | 80.9 (80.2, 81.5) | 9 (6, 13) | 82.7 (81.7, 83.5) | 7 (4, 12) |
| **Codington** | 75.1 (74.6, 75.6) | 8 (6, 11) | 76.2 (75.6, 76.8) | 11 (9, 13) | 81 (80.4, 81.5) | 8 (6, 12) | 82.2 (81.5, 82.9) | 9 (6, 13) |
| **Corson** | 73 (72.3, 73.4) | 19 (16, 22) | 72.9 (72.1, 73.4) | 26 (23, 30) | 79.8 (79.1, 80.4) | 15 (11, 19) | 79.9 (79, 80.6) | 22 (17, 26) |
| **Custer** | 70.7 (70, 71.1) | 29 (26, 34) | 71.1 (70.4, 71.6) | 34 (32, 38) | 78.7 (78.1, 79.3) | 20 (18, 23) | 78.5 (77.8, 79.1) | 28 (26, 31) |
| **Davison** | 75.4 (74.7, 75.9) | 7 (5, 10) | 76.2 (75.5, 76.7) | 11 (9, 14) | 80.9 (80.2, 81.4) | 9 (6, 13) | 81.6 (80.9, 82.3) | 12 (9, 16) |
| **Day** | 74.3 (73.7, 74.8) | 12 (10, 15) | 74.9 (74.2, 75.4) | 16 (14, 20) | 80.4 (79.8, 81) | 12 (8, 15) | 81.2 (80.4, 81.9) | 14 (11, 19) |
| **Deuel** | 75.1 (74.6, 75.6) | 8 (6, 11) | 76.2 (75.6, 76.8) | 11 (9, 13) | 81 (80.4, 81.5) | 8 (6, 12) | 82.2 (81.5, 82.9) | 9 (6, 13) |
| **Dewey** | 73 (72.3, 73.4) | 19 (16, 22) | 72.9 (72.1, 73.4) | 26 (23, 30) | 79.8 (79.1, 80.4) | 15 (11, 19) | 79.9 (79, 80.6) | 22 (17, 26) |
| **Douglas** | 74.2 (73.4, 74.7) | 13 (10, 16) | 74.9 (74.1, 75.6) | 16 (13, 20) | 80.7 (80, 81.4) | 10 (6, 14) | 81.2 (80.4, 81.9) | 14 (11, 19) |
| **Edmunds** | 74.2 (73.4, 74.7) | 13 (10, 16) | 74.1 (73.3, 74.8) | 20 (17, 24) | 80.2 (79.5, 80.8) | 13 (9, 17) | 80.3 (79.5, 81) | 19 (15, 24) |
| **Fall River** | 70.7 (70, 71.1) | 29 (26, 34) | 71.1 (70.4, 71.6) | 34 (32, 38) | 78.7 (78.1, 79.3) | 20 (18, 23) | 78.5 (77.8, 79.1) | 28 (26, 31) |
| **Faulk** | 75.5 (75, 76) | 6 (4, 9) | 76.3 (75.7, 76.8) | 10 (8, 13) | 80.7 (80.1, 81.1) | 10 (8, 14) | 82.4 (81.6, 83) | 9 (6, 12) |
| **Grant** | 75.1 (74.6, 75.6) | 8 (6, 11) | 76.2 (75.6, 76.8) | 11 (9, 13) | 81 (80.4, 81.5) | 8 (6, 12) | 82.2 (81.5, 82.9) | 9 (6, 13) |
| **Gregory** | 74.5 (73.7, 75) | 11 (9, 15) | 75 (74.2, 75.6) | 16 (13, 20) | 80.9 (80.1, 81.6) | 9 (5, 13) | 80.8 (80, 81.5) | 16 (13, 21) |
| **Haakon** | 75.3 (74.8, 75.7) | 7 (6, 10) | 76.6 (76.1, 77) | 9 (8, 11) | 80.5 (80, 80.9) | 11 (9, 14) | 81.5 (81, 82) | 12 (10, 15) |
| **Hamlin** | 74.9 (74.1, 75.4) | 10 (7, 13) | 74.9 (74.1, 75.5) | 16 (13, 20) | 81.1 (80.3, 81.7) | 8 (5, 12) | 80.6 (79.7, 81.2) | 18 (14, 22) |
| **Hand** | 74.6 (73.9, 75.1) | 11 (9, 14) | 75.8 (75, 76.4) | 12 (10, 16) | 81 (80.4, 81.6) | 8 (5, 12) | 81.4 (80.6, 82.1) | 13 (10, 17) |
| **Hanson** | 75.4 (74.7, 75.9) | 7 (5, 10) | 76.2 (75.5, 76.7) | 11 (9, 14) | 80.9 (80.2, 81.4) | 9 (6, 13) | 81.6 (80.9, 82.3) | 12 (9, 16) |
| **Harding** | 74.5 (73.9, 74.9) | 11 (9, 14) | 74.8 (74.1, 75.3) | 17 (14, 20) | 80.7 (80.1, 81.3) | 10 (7, 13) | 80.5 (79.9, 81.1) | 18 (15, 22) |
| **Hughes** | 75 (74.2, 75.5) | 9 (6, 13) | 76.2 (75.4, 76.9) | 11 (8, 14) | 81 (80.3, 81.7) | 8 (5, 13) | 81.5 (80.7, 82.2) | 13 (9, 17) |
| **Hutchinson** | 74.2 (73.4, 74.7) | 13 (10, 16) | 74.9 (74.1, 75.6) | 16 (13, 20) | 80.7 (80, 81.4) | 10 (6, 14) | 81.2 (80.4, 81.9) | 14 (11, 19) |
| **Hyde** | 75.5 (75, 76) | 6 (4, 9) | 76.3 (75.7, 76.8) | 10 (8, 13) | 80.7 (80.1, 81.1) | 10 (8, 14) | 82.4 (81.6, 83) | 9 (6, 12) |
| **Jackson** | 70.9 (70.1, 71.4) | 28 (25, 33) | 71 (70.1, 71.6) | 34 (32, 40) | 79.2 (78.5, 79.7) | 18 (16, 21) | 78.3 (77.5, 78.8) | 29 (27, 32) |
| **Jerauld** | 74.6 (73.9, 75.1) | 11 (9, 14) | 75.8 (75, 76.4) | 12 (10, 16) | 81 (80.4, 81.6) | 8 (5, 12) | 81.4 (80.6, 82.1) | 13 (10, 17) |
| **Jones** | 75.3 (74.8, 75.7) | 7 (6, 10) | 76.6 (76.1, 77) | 9 (8, 11) | 80.5 (80, 80.9) | 11 (9, 14) | 81.5 (81, 82) | 12 (10, 15) |
| **Kingsbury** | 74.9 (74.1, 75.4) | 10 (7, 13) | 74.9 (74.1, 75.5) | 16 (13, 20) | 81.1 (80.3, 81.7) | 8 (5, 12) | 80.6 (79.7, 81.2) | 18 (14, 22) |
| **Lake** | 75.2 (74.5, 75.8) | 8 (5, 11) | 76.8 (76, 77.4) | 8 (6, 11) | 81.3 (80.6, 82) | 7 (3, 11) | 82.3 (81.3, 83) | 9 (6, 14) |
| **Lawrence** | 75.2 (74.5, 75.8) | 8 (5, 11) | 77 (76.2, 77.6) | 8 (5, 11) | 80.4 (79.7, 81) | 12 (8, 16) | 81.3 (80.5, 82.1) | 14 (10, 18) |
| **Lincoln** | 76.4 (75.7, 77) | 3 (0, 6) | 77.3 (76.6, 77.9) | 6 (4, 9) | 80.9 (80.2, 81.6) | 9 (5, 13) | 81.4 (80.5, 82.3) | 13 (9, 18) |
| **Lyman** | 75 (74.2, 75.5) | 9 (6, 13) | 76.2 (75.4, 76.9) | 11 (8, 14) | 81 (80.3, 81.7) | 8 (5, 13) | 81.5 (80.7, 82.2) | 13 (9, 17) |
| **Marshall** | 74.3 (73.7, 74.8) | 12 (10, 15) | 74.9 (74.2, 75.4) | 16 (14, 20) | 80.4 (79.8, 81) | 12 (8, 15) | 81.2 (80.4, 81.9) | 14 (11, 19) |
| **McCook** | 75.4 (74.7, 75.9) | 7 (5, 10) | 76.2 (75.5, 76.7) | 11 (9, 14) | 80.9 (80.2, 81.4) | 9 (6, 13) | 81.6 (80.9, 82.3) | 12 (9, 16) |
| **McPherson** | 74.2 (73.4, 74.7) | 13 (10, 16) | 74.1 (73.3, 74.8) | 20 (17, 24) | 80.2 (79.5, 80.8) | 13 (9, 17) | 80.3 (79.5, 81) | 19 (15, 24) |
| **Meade** | 74.5 (73.9, 74.9) | 11 (9, 14) | 74.8 (74.1, 75.3) | 17 (14, 20) | 80.7 (80.1, 81.3) | 10 (7, 13) | 80.5 (79.9, 81.1) | 18 (15, 22) |
| **Mellette** | 70.9 (70.1, 71.4) | 28 (25, 33) | 71 (70.1, 71.6) | 34 (32, 40) | 79.2 (78.5, 79.7) | 18 (16, 21) | 78.3 (77.5, 78.8) | 29 (27, 32) |
| **Miner** | 75.4 (74.7, 75.9) | 7 (5, 10) | 76.2 (75.5, 76.7) | 11 (9, 14) | 80.9 (80.2, 81.4) | 9 (6, 13) | 81.6 (80.9, 82.3) | 12 (9, 16) |
| **Minnehaha** | 75.4 (74.9, 75.7) | 7 (6, 9) | 77.1 (76.7, 77.4) | 7 (6, 9) | 81.2 (80.8, 81.6) | 7 (5, 9) | 82.3 (81.8, 82.7) | 9 (7, 11) |
| **Moody** | 75.2 (74.5, 75.8) | 8 (5, 11) | 76.8 (76, 77.4) | 8 (6, 11) | 81.3 (80.6, 82) | 7 (3, 11) | 82.3 (81.3, 83) | 9 (6, 14) |
| **Pennington** | 75.3 (74.8, 75.7) | 7 (6, 10) | 76.6 (76.1, 77) | 9 (8, 11) | 80.5 (80, 80.9) | 11 (9, 14) | 81.5 (81, 82) | 12 (10, 15) |
| **Perkins** | 74.5 (73.9, 74.9) | 11 (9, 14) | 74.8 (74.1, 75.3) | 17 (14, 20) | 80.7 (80.1, 81.3) | 10 (7, 13) | 80.5 (79.9, 81.1) | 18 (15, 22) |
| **Potter** | 73 (72.3, 73.4) | 19 (16, 22) | 72.9 (72.1, 73.4) | 26 (23, 30) | 79.8 (79.1, 80.4) | 15 (11, 19) | 79.9 (79, 80.6) | 22 (17, 26) |
| **Roberts** | 74.3 (73.7, 74.8) | 12 (10, 15) | 74.9 (74.2, 75.4) | 16 (14, 20) | 80.4 (79.8, 81) | 12 (8, 15) | 81.2 (80.4, 81.9) | 14 (11, 19) |
| **Sanborn** | 74.6 (73.9, 75.1) | 11 (9, 14) | 75.8 (75, 76.4) | 12 (10, 16) | 81 (80.4, 81.6) | 8 (5, 12) | 81.4 (80.6, 82.1) | 13 (10, 17) |
| **Shannon** | 70.7 (70, 71.1) | 29 (26, 34) | 71.1 (70.4, 71.6) | 34 (32, 38) | 78.7 (78.1, 79.3) | 20 (18, 23) | 78.5 (77.8, 79.1) | 28 (26, 31) |
| **Spink** | 74.3 (73.7, 74.8) | 12 (10, 15) | 74.9 (74.2, 75.4) | 16 (14, 20) | 80.4 (79.8, 81) | 12 (8, 15) | 81.2 (80.4, 81.9) | 14 (11, 19) |
| **Stanley** | 75.3 (74.8, 75.7) | 7 (6, 10) | 76.6 (76.1, 77) | 9 (8, 11) | 80.5 (80, 80.9) | 11 (9, 14) | 81.5 (81, 82) | 12 (10, 15) |
| **Sully** | 75.3 (74.8, 75.7) | 7 (6, 10) | 76.6 (76.1, 77) | 9 (8, 11) | 80.5 (80, 80.9) | 11 (9, 14) | 81.5 (81, 82) | 12 (10, 15) |
| **Todd** | 70.9 (70.1, 71.4) | 28 (25, 33) | 71 (70.1, 71.6) | 34 (32, 40) | 79.2 (78.5, 79.7) | 18 (16, 21) | 78.3 (77.5, 78.8) | 29 (27, 32) |
| **Tripp** | 74.5 (73.7, 75) | 11 (9, 15) | 75 (74.2, 75.6) | 16 (13, 20) | 80.9 (80.1, 81.6) | 9 (5, 13) | 80.8 (80, 81.5) | 16 (13, 21) |
| **Turner** | 75.8 (75.1, 76.3) | 5 (3, 8) | 76.9 (76.2, 77.5) | 8 (6, 11) | 80.6 (80, 81.2) | 10 (7, 14) | 81.5 (80.7, 82.1) | 13 (10, 17) |
| **Union** | 75.6 (75, 76.1) | 6 (4, 9) | 77.4 (76.7, 78) | 6 (4, 9) | 80.9 (80.2, 81.5) | 9 (6, 13) | 82.7 (81.7, 83.5) | 7 (4, 12) |
| **Walworth** | 74.2 (73.4, 74.7) | 13 (10, 16) | 74.1 (73.3, 74.8) | 20 (17, 24) | 80.2 (79.5, 80.8) | 13 (9, 17) | 80.3 (79.5, 81) | 19 (15, 24) |
| **Yankton** | 75.8 (75.1, 76.3) | 5 (3, 8) | 76.9 (76.2, 77.5) | 8 (6, 11) | 80.6 (80, 81.2) | 10 (7, 14) | 81.5 (80.7, 82.1) | 13 (10, 17) |
| **Ziebach** | 73 (72.3, 73.4) | 19 (16, 22) | 72.9 (72.1, 73.4) | 26 (23, 30) | 79.8 (79.1, 80.4) | 15 (11, 19) | 79.9 (79, 80.6) | 22 (17, 26) |
| **TENNESSEE** |  |  |  |  |  |  |  |  |
| **Anderson** | 73.5 (73.1, 73.9) | 16 (14, 18) | 73.7 (73.2, 74.1) | 22 (20, 24) | 79.4 (78.9, 79.8) | 17 (15, 20) | 79.2 (78.7, 79.7) | 25 (23, 27) |
| **Bedford** | 71.9 (71.3, 72.3) | 23 (21, 26) | 71.9 (71.3, 72.4) | 30 (28, 33) | 78.4 (77.9, 78.9) | 21 (20, 23) | 79 (78.3, 79.6) | 26 (23, 29) |
| **Benton** | 70.9 (70.1, 71.5) | 28 (25, 33) | 70.6 (69.7, 71.2) | 36 (33, 45) | 79 (78.3, 79.6) | 19 (16, 22) | 77.8 (76.9, 78.4) | 31 (29, 34) |
| **Bledsoe** | 71.7 (71, 72.2) | 24 (22, 27) | 72.7 (71.9, 73.3) | 27 (24, 30) | 78.8 (78.1, 79.4) | 20 (17, 23) | 79.1 (78.2, 79.8) | 26 (22, 29) |
| **Blount** | 73.5 (73.1, 73.9) | 16 (14, 18) | 74.1 (73.6, 74.4) | 20 (19, 22) | 79.3 (78.9, 79.7) | 18 (16, 20) | 79.6 (79.1, 80) | 23 (21, 26) |
| **Bradley** | 72.6 (72.1, 73) | 20 (18, 23) | 73.3 (72.7, 73.7) | 24 (22, 27) | 78.5 (78, 78.9) | 21 (20, 23) | 79.2 (78.7, 79.6) | 25 (23, 28) |
| **Campbell** | 71.1 (70.5, 71.6) | 27 (24, 30) | 70.9 (70.2, 71.4) | 35 (32, 39) | 78 (77.4, 78.5) | 23 (21, 25) | 77.4 (76.7, 77.9) | 32 (30, 35) |
| **Cannon** | 71.2 (70.6, 71.7) | 26 (24, 29) | 71.5 (70.8, 72.1) | 32 (30, 35) | 78.4 (77.8, 78.9) | 21 (19, 24) | 78.4 (77.7, 79) | 29 (26, 31) |
| **Carroll** | 71 (70.4, 71.5) | 27 (25, 31) | 70.1 (69.5, 70.6) | 39 (36, 49) | 77.7 (77.2, 78.2) | 24 (22, 26) | 77.2 (76.5, 77.7) | 33 (31, 36) |
| **Carter** | 71.8 (71.3, 72.2) | 24 (22, 26) | 72.4 (71.9, 72.9) | 28 (26, 30) | 78.6 (78.1, 79.1) | 21 (19, 23) | 78.6 (78, 79.1) | 28 (26, 30) |
| **Cheatham** | 73.5 (72.8, 74) | 16 (13, 19) | 73.5 (72.8, 74.1) | 23 (20, 26) | 78.3 (77.7, 78.8) | 22 (20, 24) | 78.8 (78.1, 79.4) | 27 (25, 30) |
| **Chester** | 71.7 (71, 72.3) | 24 (22, 27) | 72.9 (72.1, 73.5) | 26 (23, 30) | 78.6 (77.9, 79.2) | 21 (18, 23) | 78.2 (77.4, 78.8) | 29 (27, 32) |
| **Claiborne** | 70.8 (70.1, 71.4) | 28 (25, 33) | 71.1 (70.3, 71.7) | 34 (31, 38) | 77.8 (77.2, 78.3) | 24 (22, 26) | 77.7 (77, 78.4) | 31 (29, 34) |
| **Clay** | 70.5 (69.9, 71) | 30 (27, 35) | 71.2 (70.5, 71.8) | 33 (31, 37) | 77.9 (77.3, 78.4) | 23 (21, 26) | 77.1 (76.4, 77.7) | 34 (31, 36) |
| **Cocke** | 70.3 (69.6, 70.8) | 31 (28, 40) | 69.9 (69.2, 70.5) | 42 (37, 51) | 77.3 (76.7, 77.8) | 26 (24, 28) | 76.7 (76, 77.3) | 35 (33, 38) |
| **Coffee** | 72.3 (71.7, 72.8) | 22 (20, 24) | 72.3 (71.6, 72.7) | 29 (27, 31) | 79.4 (78.8, 79.8) | 17 (15, 20) | 78.5 (77.9, 79) | 28 (26, 30) |
| **Crockett** | 71.2 (70.4, 71.7) | 26 (24, 30) | 70.7 (69.9, 71.3) | 36 (33, 43) | 78 (77.4, 78.6) | 23 (21, 25) | 77.4 (76.8, 78) | 32 (30, 35) |
| **Cumberland** | 73.9 (73.3, 74.5) | 14 (11, 17) | 74.7 (74, 75.3) | 17 (15, 20) | 79.8 (79.3, 80.3) | 15 (12, 18) | 80.6 (80, 81.2) | 17 (14, 21) |
| **Davidson** | 71.7 (71.6, 71.9) | 24 (23, 25) | 73.3 (73.1, 73.5) | 24 (23, 25) | 78.3 (78.1, 78.5) | 22 (21, 23) | 79.2 (79, 79.4) | 25 (24, 26) |
| **De Kalb** | 71.2 (70.6, 71.7) | 26 (24, 29) | 71.5 (70.8, 72.1) | 32 (30, 35) | 78.4 (77.8, 78.9) | 21 (19, 24) | 78.4 (77.7, 79) | 29 (26, 31) |
| **Decatur** | 70.7 (69.9, 71.3) | 29 (26, 36) | 70.6 (69.8, 71.2) | 36 (33, 44) | 77.8 (77.1, 78.3) | 24 (22, 26) | 78.2 (77.4, 78.9) | 29 (27, 32) |
| **Dickson** | 72.6 (72, 73.1) | 20 (18, 23) | 72.4 (71.8, 72.9) | 28 (26, 31) | 79.2 (78.6, 79.7) | 18 (16, 21) | 78.8 (78.2, 79.4) | 27 (25, 29) |
| **Dyer** | 70.1 (69.5, 70.5) | 33 (30, 42) | 71.2 (70.6, 71.6) | 33 (31, 36) | 77.7 (77.1, 78.1) | 24 (23, 26) | 77.7 (77.1, 78.2) | 31 (29, 33) |
| **Fayette** | 70.9 (70.3, 71.4) | 27 (26, 31) | 72.3 (71.7, 72.8) | 29 (26, 31) | 77.8 (77.2, 78.2) | 24 (22, 26) | 78.3 (77.7, 78.7) | 29 (27, 31) |
| **Fentress** | 70.7 (69.8, 71.4) | 29 (25, 36) | 69.5 (68.5, 70.3) | 49 (38, 51) | 77 (76.3, 77.7) | 27 (24, 29) | 77.1 (76.3, 77.9) | 33 (31, 37) |
| **Franklin** | 72.8 (72.1, 73.2) | 20 (17, 22) | 73.5 (72.8, 74.1) | 23 (20, 26) | 79.1 (78.5, 79.6) | 19 (16, 21) | 78.9 (78.2, 79.4) | 27 (24, 29) |
| **Gibson** | 70.2 (69.7, 70.5) | 32 (30, 39) | 70.3 (69.7, 70.6) | 38 (36, 44) | 77.7 (77.3, 78.1) | 24 (23, 26) | 77.5 (77, 77.9) | 32 (31, 34) |
| **Giles** | 72.4 (71.8, 72.8) | 21 (19, 24) | 72.2 (71.5, 72.6) | 29 (27, 32) | 78.8 (78.2, 79.2) | 20 (18, 22) | 78.9 (78.2, 79.4) | 27 (24, 29) |
| **Grainger** | 71 (70.3, 71.5) | 27 (25, 31) | 71.2 (70.5, 71.7) | 33 (31, 37) | 77.9 (77.3, 78.5) | 23 (21, 25) | 77.2 (76.6, 77.9) | 33 (31, 35) |
| **Greene** | 71.3 (70.7, 71.7) | 26 (24, 28) | 72.6 (72.1, 73) | 27 (25, 30) | 78.4 (77.9, 78.8) | 22 (20, 23) | 77.8 (77.2, 78.2) | 31 (29, 33) |
| **Grundy** | 70.2 (69.3, 70.8) | 32 (28, 43) | 68.8 (67.8, 69.4) | 51 (49, 51) | 77.4 (76.7, 78) | 25 (23, 28) | 77.9 (77, 78.6) | 31 (28, 34) |
| **Hamblen** | 72 (71.5, 72.4) | 23 (21, 25) | 72.4 (71.8, 72.8) | 28 (26, 31) | 78.6 (78.1, 79) | 21 (19, 23) | 78.3 (77.7, 78.7) | 29 (27, 31) |
| **Hamilton** | 72.6 (72.4, 72.8) | 20 (19, 21) | 73.4 (73.1, 73.6) | 23 (22, 25) | 78.6 (78.4, 78.8) | 21 (20, 22) | 79.5 (79.3, 79.8) | 24 (22, 25) |
| **Hancock** | 71 (70.3, 71.5) | 27 (25, 31) | 71.2 (70.5, 71.7) | 33 (31, 37) | 77.9 (77.3, 78.5) | 23 (21, 25) | 77.2 (76.6, 77.9) | 33 (31, 35) |
| **Hardeman** | 69.7 (69, 70.1) | 39 (33, 46) | 70.7 (70.1, 71.1) | 36 (34, 40) | 77 (76.4, 77.4) | 27 (25, 29) | 77.2 (76.6, 77.7) | 33 (31, 36) |
| **Hardin** | 71.7 (71, 72.2) | 24 (22, 27) | 71.5 (70.7, 72) | 32 (30, 36) | 78.3 (77.8, 78.8) | 22 (20, 24) | 78.1 (77.5, 78.7) | 30 (27, 32) |
| **Hawkins** | 72.2 (71.6, 72.6) | 22 (20, 24) | 72.6 (72, 73.1) | 27 (25, 30) | 79.2 (78.6, 79.7) | 18 (16, 21) | 78.6 (78, 79.1) | 28 (26, 30) |
| **Haywood** | 69.1 (68.4, 69.5) | 45 (41, 49) | 68.8 (68.1, 69.3) | 51 (51, 51) | 76.6 (76, 77) | 28 (27, 31) | 77 (76.3, 77.4) | 34 (32, 37) |
| **Henderson** | 70.4 (69.7, 70.9) | 30 (27, 38) | 71.3 (70.6, 71.9) | 33 (30, 36) | 78.4 (77.7, 78.9) | 22 (19, 24) | 77.3 (76.6, 77.9) | 33 (30, 35) |
| **Henry** | 71.7 (71.1, 72.2) | 24 (22, 27) | 71.6 (70.9, 72.1) | 32 (30, 35) | 78.4 (77.8, 78.9) | 22 (20, 24) | 77.9 (77.3, 78.4) | 30 (28, 33) |
| **Hickman** | 71 (70.4, 71.6) | 27 (24, 31) | 71.7 (71, 72.3) | 31 (29, 34) | 78 (77.4, 78.6) | 23 (21, 25) | 79 (78.1, 79.7) | 26 (22, 30) |
| **Houston** | 72.2 (71.5, 72.8) | 22 (20, 25) | 72.4 (71.6, 73) | 28 (25, 32) | 79 (78.3, 79.6) | 19 (16, 22) | 78.3 (77.5, 78.8) | 29 (27, 32) |
| **Humphreys** | 72.9 (72.2, 73.5) | 19 (16, 22) | 72.1 (71.2, 72.7) | 30 (27, 33) | 79 (78.4, 79.7) | 19 (16, 22) | 78.5 (77.7, 79.2) | 28 (25, 31) |
| **Jackson** | 71.1 (70.4, 71.7) | 27 (24, 30) | 71.3 (70.6, 72) | 33 (30, 36) | 78.5 (77.8, 79.1) | 21 (19, 24) | 77.6 (76.9, 78.2) | 31 (29, 34) |
| **Jefferson** | 72.5 (71.9, 73) | 21 (18, 23) | 73 (72.4, 73.5) | 25 (23, 28) | 79 (78.4, 79.6) | 19 (16, 21) | 78.7 (78, 79.2) | 27 (25, 30) |
| **Johnson** | 71 (70.3, 71.6) | 27 (25, 31) | 70.8 (70, 71.4) | 35 (32, 41) | 77.6 (76.9, 78.2) | 24 (22, 27) | 77.4 (76.5, 78.1) | 32 (30, 36) |
| **Knox** | 73.2 (73, 73.4) | 17 (16, 18) | 74.3 (74.1, 74.5) | 19 (18, 20) | 79.1 (78.9, 79.3) | 19 (18, 20) | 79.5 (79.3, 79.7) | 24 (23, 25) |
| **Lake** | 71.4 (70.9, 71.8) | 25 (24, 28) | 71.8 (71.2, 72.2) | 31 (29, 33) | 77.9 (77.4, 78.3) | 24 (22, 25) | 77.9 (77.3, 78.4) | 30 (29, 33) |
| **Lauderdale** | 69 (68.4, 69.4) | 46 (43, 50) | 69.8 (69.2, 70.2) | 44 (39, 51) | 76.8 (76.3, 77.3) | 27 (26, 30) | 76.4 (75.8, 76.9) | 36 (34, 40) |
| **Lawrence** | 71.4 (70.8, 71.9) | 25 (23, 28) | 71.5 (70.8, 72) | 32 (30, 35) | 79.2 (78.6, 79.7) | 18 (16, 21) | 78.6 (77.9, 79.1) | 28 (26, 30) |
| **Lewis** | 71.4 (70.7, 71.8) | 25 (23, 29) | 71.1 (70.5, 71.6) | 33 (31, 37) | 78.5 (77.9, 79.1) | 21 (19, 24) | 77.6 (76.9, 78.2) | 31 (29, 34) |
| **Lincoln** | 72 (71.4, 72.4) | 23 (21, 25) | 72.8 (72.2, 73.3) | 26 (24, 29) | 78.8 (78.3, 79.2) | 20 (18, 22) | 79 (78.3, 79.4) | 26 (24, 29) |
| **Loudon** | 73.6 (73, 74.1) | 15 (13, 18) | 74.2 (73.6, 74.8) | 19 (17, 22) | 79.4 (78.8, 79.9) | 17 (14, 20) | 80 (79.4, 80.5) | 21 (18, 24) |
| **Macon** | 71.7 (71, 72.2) | 24 (22, 27) | 71 (70.3, 71.5) | 34 (32, 38) | 78.1 (77.4, 78.6) | 23 (21, 25) | 77.4 (76.7, 78) | 32 (30, 35) |
| **Madison** | 71.9 (71.5, 72.2) | 23 (22, 25) | 72.7 (72.3, 73.1) | 27 (25, 29) | 78.3 (77.9, 78.6) | 22 (21, 24) | 78.9 (78.5, 79.2) | 27 (25, 28) |
| **Marion** | 70.7 (70, 71.2) | 29 (26, 34) | 71.2 (70.4, 71.7) | 33 (31, 37) | 78.2 (77.5, 78.7) | 22 (20, 25) | 77.8 (77.1, 78.4) | 31 (29, 33) |
| **Marshall** | 72.3 (71.6, 72.8) | 22 (19, 25) | 72.4 (71.7, 73) | 28 (25, 31) | 79.2 (78.6, 79.8) | 18 (15, 21) | 79.1 (78.4, 79.7) | 26 (22, 29) |
| **Maury** | 72.4 (72, 72.8) | 21 (20, 23) | 72.8 (72.3, 73.2) | 26 (24, 28) | 78.7 (78.3, 79.1) | 20 (19, 22) | 78.5 (78, 78.8) | 28 (27, 30) |
| **McMinn** | 71.8 (71.2, 72.2) | 24 (22, 26) | 71.9 (71.3, 72.3) | 30 (29, 33) | 78.7 (78.2, 79.2) | 20 (18, 22) | 78.3 (77.7, 78.8) | 29 (27, 31) |
| **McNairy** | 70.8 (70.1, 71.3) | 28 (26, 33) | 71.4 (70.7, 72) | 32 (30, 36) | 78.5 (77.8, 79) | 21 (19, 24) | 78.4 (77.7, 79) | 28 (26, 31) |
| **Meigs** | 71.7 (71.1, 72.2) | 24 (22, 27) | 71.1 (70.4, 71.6) | 34 (31, 37) | 78.6 (78, 79.1) | 21 (18, 23) | 78 (77.4, 78.6) | 30 (28, 32) |
| **Monroe** | 71.6 (71, 72.1) | 24 (22, 27) | 72.5 (71.9, 73) | 28 (25, 30) | 78.3 (77.7, 78.8) | 22 (20, 24) | 78.7 (78, 79.2) | 28 (25, 30) |
| **Montgomery** | 73.1 (72.6, 73.4) | 18 (16, 20) | 74.3 (73.8, 74.6) | 19 (18, 21) | 78.7 (78.3, 79.1) | 20 (19, 22) | 79.1 (78.7, 79.4) | 26 (24, 28) |
| **Moore** | 72 (71.4, 72.4) | 23 (21, 25) | 72.8 (72.2, 73.3) | 26 (24, 29) | 78.8 (78.3, 79.2) | 20 (18, 22) | 79 (78.3, 79.4) | 26 (24, 29) |
| **Morgan** | 71.6 (70.9, 72.2) | 24 (22, 27) | 71.5 (70.8, 72.1) | 32 (30, 35) | 78.3 (77.6, 78.9) | 22 (19, 25) | 77.7 (76.9, 78.4) | 31 (29, 34) |
| **Obion** | 71.4 (70.9, 71.8) | 25 (24, 28) | 71.8 (71.2, 72.2) | 31 (29, 33) | 77.9 (77.4, 78.3) | 24 (22, 25) | 77.9 (77.3, 78.4) | 30 (29, 33) |
| **Overton** | 70.5 (69.9, 71) | 30 (27, 35) | 71.2 (70.5, 71.8) | 33 (31, 37) | 77.9 (77.3, 78.4) | 23 (21, 26) | 77.1 (76.4, 77.7) | 34 (31, 36) |
| **Perry** | 70.7 (69.9, 71.3) | 29 (26, 36) | 70.6 (69.8, 71.2) | 36 (33, 44) | 77.8 (77.1, 78.3) | 24 (22, 26) | 78.2 (77.4, 78.9) | 29 (27, 32) |
| **Pickett** | 70.5 (69.9, 71) | 30 (27, 35) | 71.2 (70.5, 71.8) | 33 (31, 37) | 77.9 (77.3, 78.4) | 23 (21, 26) | 77.1 (76.4, 77.7) | 34 (31, 36) |
| **Polk** | 71.5 (70.7, 72.1) | 25 (22, 29) | 70.9 (69.9, 71.6) | 35 (32, 42) | 77.3 (76.7, 77.9) | 26 (24, 28) | 77.7 (76.9, 78.4) | 31 (29, 34) |
| **Putnam** | 72.6 (72.1, 73) | 20 (18, 23) | 73.4 (72.8, 73.8) | 23 (21, 26) | 79.2 (78.7, 79.6) | 18 (16, 21) | 79 (78.4, 79.4) | 26 (24, 28) |
| **Rhea** | 71.7 (71.1, 72.2) | 24 (22, 27) | 71.1 (70.4, 71.6) | 34 (31, 37) | 78.6 (78, 79.1) | 21 (18, 23) | 78 (77.4, 78.6) | 30 (28, 32) |
| **Roane** | 72.5 (72, 72.9) | 21 (19, 23) | 73.2 (72.5, 73.7) | 24 (22, 28) | 79.2 (78.6, 79.7) | 18 (16, 21) | 79.4 (78.7, 79.9) | 24 (22, 27) |
| **Robertson** | 72.9 (72.4, 73.3) | 19 (17, 21) | 72.8 (72.3, 73.2) | 26 (24, 29) | 78.3 (77.9, 78.7) | 22 (20, 24) | 78.5 (77.9, 78.9) | 28 (26, 30) |
| **Rutherford** | 73.9 (73.5, 74.2) | 14 (13, 16) | 75.2 (74.9, 75.6) | 15 (13, 17) | 79.1 (78.7, 79.4) | 19 (17, 20) | 79.5 (79.1, 79.8) | 24 (22, 26) |
| **Scott** | 70.6 (69.9, 71.2) | 29 (26, 36) | 70.7 (69.9, 71.3) | 36 (33, 43) | 77.7 (77, 78.3) | 24 (22, 27) | 77.1 (76.3, 77.8) | 33 (31, 37) |
| **Sequatchie** | 71.7 (71, 72.2) | 24 (22, 27) | 72.7 (71.9, 73.3) | 27 (24, 30) | 78.8 (78.1, 79.4) | 20 (17, 23) | 79.1 (78.2, 79.8) | 26 (22, 29) |
| **Sevier** | 73.6 (73.1, 74.1) | 15 (13, 18) | 73.6 (73.1, 74.1) | 22 (20, 25) | 79.3 (78.8, 79.7) | 18 (16, 20) | 79.6 (79.1, 80.1) | 23 (20, 26) |
| **Shelby** | 70 (69.9, 70.2) | 34 (32, 35) | 71.4 (71.2, 71.5) | 33 (32, 33) | 76.4 (76.3, 76.6) | 29 (29, 30) | 77.7 (77.6, 77.8) | 31 (31, 32) |
| **Smith** | 71.1 (70.4, 71.7) | 27 (24, 30) | 71.3 (70.6, 72) | 33 (30, 36) | 78.5 (77.8, 79.1) | 21 (19, 24) | 77.6 (76.9, 78.2) | 31 (29, 34) |
| **Stewart** | 72.2 (71.5, 72.8) | 22 (20, 25) | 72.4 (71.6, 73) | 28 (25, 32) | 79 (78.3, 79.6) | 19 (16, 22) | 78.3 (77.5, 78.8) | 29 (27, 32) |
| **Sullivan** | 73 (72.6, 73.3) | 18 (17, 20) | 72.6 (72.2, 73) | 27 (25, 29) | 79.2 (78.9, 79.6) | 18 (16, 20) | 79 (78.6, 79.3) | 26 (25, 28) |
| **Sumner** | 73.4 (73, 73.8) | 16 (15, 18) | 74.9 (74.5, 75.2) | 16 (15, 18) | 79.5 (79.1, 79.9) | 17 (15, 19) | 79.4 (79, 79.7) | 24 (23, 26) |
| **Tipton** | 72 (71.5, 72.4) | 23 (21, 25) | 72.2 (71.7, 72.6) | 29 (27, 31) | 77.6 (77.1, 78) | 24 (23, 26) | 78 (77.4, 78.4) | 30 (28, 32) |
| **Trousdale** | 71.7 (71, 72.2) | 24 (22, 27) | 71 (70.3, 71.5) | 34 (32, 38) | 78.1 (77.4, 78.6) | 23 (21, 25) | 77.4 (76.7, 78) | 32 (30, 35) |
| **Unicoi** | 72.3 (71.5, 73) | 21 (19, 25) | 71.4 (70.6, 72) | 32 (30, 36) | 79.3 (78.6, 79.9) | 18 (15, 21) | 78.6 (77.9, 79.3) | 28 (25, 31) |
| **Union** | 70.8 (70.1, 71.4) | 28 (25, 33) | 71.1 (70.2, 71.8) | 34 (31, 39) | 78.2 (77.4, 78.8) | 22 (20, 25) | 77.9 (77, 78.7) | 30 (27, 34) |
| **Van Buren** | 71.3 (70.6, 71.7) | 26 (24, 29) | 71.6 (70.9, 72.1) | 32 (30, 35) | 78.2 (77.6, 78.7) | 22 (20, 25) | 78.2 (77.5, 78.7) | 29 (27, 32) |
| **Warren** | 72.6 (72, 73.1) | 20 (18, 23) | 71.4 (70.8, 71.9) | 32 (30, 35) | 78.9 (78.3, 79.4) | 20 (17, 22) | 78 (77.3, 78.5) | 30 (28, 32) |
| **Washington** | 72.6 (72.2, 73) | 20 (18, 22) | 72.5 (72, 72.8) | 28 (26, 30) | 79 (78.6, 79.3) | 19 (18, 21) | 79.6 (79.1, 79.9) | 23 (22, 26) |
| **Wayne** | 71.4 (70.7, 71.8) | 25 (23, 29) | 71.1 (70.5, 71.6) | 33 (31, 37) | 78.5 (77.9, 79.1) | 21 (19, 24) | 77.6 (76.9, 78.2) | 31 (29, 34) |
| **Weakley** | 72.7 (72, 73.1) | 20 (18, 23) | 72.9 (72.2, 73.4) | 26 (23, 29) | 78.5 (77.9, 79) | 21 (19, 23) | 78.4 (77.7, 78.9) | 29 (27, 31) |
| **White** | 71.3 (70.6, 71.7) | 26 (24, 29) | 71.6 (70.9, 72.1) | 32 (30, 35) | 78.2 (77.6, 78.7) | 22 (20, 25) | 78.2 (77.5, 78.7) | 29 (27, 32) |
| **Williamson** | 76.5 (76, 76.9) | 2 (1, 4) | 77.9 (77.4, 78.2) | 5 (3, 6) | 81 (80.6, 81.4) | 8 (6, 11) | 82 (81.6, 82.5) | 10 (8, 12) |
| **Wilson** | 73.4 (72.9, 73.7) | 16 (15, 19) | 74.7 (74.2, 75.1) | 17 (15, 19) | 79.5 (79, 79.9) | 17 (15, 19) | 78.9 (78.5, 79.3) | 26 (25, 28) |
| **TEXAS** |  |  |  |  |  |  |  |  |
| **Anderson** | 68.9 (68.4, 69.2) | 47 (45, 50) | 69.8 (69.3, 70.1) | 44 (39, 51) | 77.6 (77.1, 78.1) | 24 (23, 26) | 77.8 (77.2, 78.3) | 31 (29, 33) |
| **Andrews** | 72.5 (71.9, 73) | 21 (18, 23) | 73.9 (73.2, 74.5) | 21 (18, 25) | 78.3 (77.7, 78.9) | 22 (20, 24) | 78.9 (78.2, 79.5) | 27 (24, 29) |
| **Angelina** | 72.3 (71.8, 72.6) | 22 (20, 24) | 73.1 (72.6, 73.5) | 25 (23, 27) | 78.1 (77.7, 78.4) | 23 (21, 24) | 78.7 (78.2, 79.1) | 27 (26, 29) |
| **Aransas** | 73.3 (72.5, 73.9) | 17 (14, 21) | 73.9 (73.1, 74.6) | 21 (18, 25) | 79.7 (79, 80.3) | 16 (12, 19) | 81.3 (80.4, 82.1) | 14 (10, 18) |
| **Archer** | 74 (73.3, 74.5) | 14 (11, 17) | 74.9 (74.2, 75.6) | 16 (13, 20) | 79.9 (79.2, 80.6) | 14 (10, 18) | 81.1 (80.2, 81.9) | 15 (11, 20) |
| **Armstrong** | 73.6 (72.9, 74.1) | 15 (13, 19) | 74.6 (73.8, 75.2) | 18 (15, 21) | 79.5 (78.9, 80.1) | 17 (14, 20) | 79.8 (79, 80.4) | 22 (18, 26) |
| **Atascosa** | 73.1 (72.5, 73.6) | 18 (15, 21) | 74.6 (74, 75.1) | 18 (16, 21) | 79.6 (79, 80.1) | 16 (14, 19) | 80.4 (79.7, 81) | 19 (15, 23) |
| **Austin** | 73.4 (72.8, 73.9) | 16 (14, 19) | 74.5 (73.8, 75.1) | 18 (16, 21) | 79.9 (79.3, 80.5) | 14 (11, 18) | 80.2 (79.5, 80.8) | 20 (16, 24) |
| **Bailey** | 73.2 (72.7, 73.7) | 17 (15, 20) | 73.1 (72.5, 73.6) | 25 (23, 28) | 79.2 (78.6, 79.8) | 18 (15, 21) | 78.8 (78.1, 79.4) | 27 (24, 30) |
| **Bandera** | 75.9 (75.1, 76.5) | 5 (2, 8) | 76.4 (75.6, 77) | 10 (8, 13) | 80.6 (79.8, 81.2) | 11 (7, 15) | 81.2 (80.3, 82.1) | 14 (10, 19) |
| **Bastrop** | 74 (73.5, 74.4) | 13 (12, 16) | 74 (73.5, 74.5) | 20 (18, 23) | 79.9 (79.3, 80.4) | 14 (12, 18) | 79.3 (78.8, 79.8) | 25 (22, 27) |
| **Baylor** | 72.8 (72, 73.3) | 19 (17, 23) | 72.6 (71.7, 73.2) | 27 (24, 31) | 79.1 (78.5, 79.7) | 19 (16, 21) | 79 (78.1, 79.6) | 26 (23, 30) |
| **Bee** | 73.1 (72.5, 73.6) | 18 (15, 21) | 74.2 (73.5, 74.7) | 20 (17, 23) | 79.1 (78.4, 79.6) | 19 (16, 22) | 79.4 (78.6, 80) | 24 (21, 28) |
| **Bell** | 73.7 (73.4, 74) | 15 (14, 16) | 74.5 (74.2, 74.7) | 18 (17, 20) | 79.6 (79.2, 79.9) | 16 (15, 18) | 79.7 (79.3, 79.9) | 23 (21, 25) |
| **Bexar** | 74.1 (74, 74.2) | 13 (12, 14) | 74.9 (74.7, 75) | 17 (16, 17) | 79.7 (79.5, 79.8) | 16 (15, 17) | 80.7 (80.5, 80.8) | 17 (16, 18) |
| **Blanco** | 75.3 (74.6, 75.8) | 7 (5, 11) | 76.6 (75.8, 77.2) | 9 (7, 12) | 80.8 (80.2, 81.4) | 9 (6, 13) | 81.3 (80.6, 82) | 14 (10, 18) |
| **Borden** | 72.4 (71.8, 72.8) | 21 (19, 24) | 72.4 (71.8, 72.8) | 28 (26, 31) | 78.1 (77.4, 78.5) | 23 (21, 25) | 77.8 (77.1, 78.3) | 31 (29, 33) |
| **Bosque** | 73.1 (72.4, 73.6) | 18 (16, 21) | 73.8 (73.1, 74.4) | 21 (19, 25) | 79.2 (78.6, 79.7) | 18 (16, 21) | 79.5 (78.7, 80) | 24 (21, 27) |
| **Bowie** | 71.9 (71.6, 72.2) | 23 (22, 25) | 72.4 (72, 72.7) | 28 (27, 30) | 78.3 (77.9, 78.6) | 22 (21, 24) | 78.4 (78, 78.8) | 29 (27, 30) |
| **Brazoria** | 73.8 (73.5, 74.1) | 14 (13, 16) | 75.3 (75, 75.6) | 14 (13, 16) | 78.9 (78.6, 79.2) | 20 (18, 21) | 78.8 (78.5, 79.1) | 27 (26, 28) |
| **Brazos** | 75.5 (75.1, 75.8) | 7 (5, 9) | 77.1 (76.6, 77.5) | 7 (6, 9) | 80.2 (79.7, 80.6) | 13 (11, 16) | 80.4 (79.9, 80.7) | 19 (17, 22) |
| **Brewster** | 73.5 (72.6, 74.1) | 16 (13, 20) | 75 (74.2, 75.7) | 16 (12, 20) | 79.6 (78.8, 80.4) | 16 (12, 20) | 80.8 (79.9, 81.6) | 16 (12, 22) |
| **Briscoe** | 73.6 (72.9, 74.1) | 15 (13, 19) | 74.6 (73.8, 75.2) | 18 (15, 21) | 79.5 (78.9, 80.1) | 17 (14, 20) | 79.8 (79, 80.4) | 22 (18, 26) |
| **Brooks** | 72 (71.3, 72.6) | 23 (21, 26) | 73.4 (72.5, 74) | 23 (20, 28) | 78.9 (78.2, 79.5) | 20 (17, 22) | 78.6 (77.8, 79.2) | 28 (25, 31) |
| **Brown** | 72.9 (72.3, 73.3) | 19 (17, 21) | 72.4 (71.8, 72.8) | 28 (26, 31) | 79 (78.4, 79.5) | 19 (17, 21) | 78.9 (78.2, 79.4) | 27 (24, 29) |
| **Burleson** | 72.9 (72.2, 73.4) | 19 (16, 22) | 74.7 (74, 75.4) | 17 (14, 21) | 79.3 (78.7, 79.9) | 18 (15, 21) | 79.4 (78.7, 80) | 24 (21, 27) |
| **Burnet** | 74 (73.3, 74.5) | 14 (11, 17) | 76.9 (76.2, 77.5) | 8 (6, 11) | 80.1 (79.5, 80.6) | 14 (11, 17) | 82.3 (81.5, 83) | 9 (6, 12) |
| **Caldwell** | 73.5 (72.8, 74) | 16 (14, 19) | 73.5 (72.8, 74) | 23 (21, 26) | 78.9 (78.3, 79.3) | 20 (18, 22) | 79.4 (78.7, 80) | 24 (21, 27) |
| **Calhoun** | 73.1 (72.4, 73.7) | 18 (15, 21) | 73.8 (73, 74.4) | 21 (19, 26) | 79.2 (78.5, 79.9) | 18 (15, 21) | 78.9 (78.1, 79.5) | 27 (24, 30) |
| **Callahan** | 73 (72.3, 73.5) | 18 (16, 22) | 73.1 (72.4, 73.6) | 25 (22, 28) | 79.5 (78.8, 80) | 17 (14, 20) | 79.5 (78.7, 80.1) | 24 (21, 28) |
| **Cameron** | 76 (75.6, 76.3) | 4 (3, 6) | 76.9 (76.5, 77.2) | 8 (7, 9) | 81.3 (81, 81.6) | 7 (5, 8) | 83 (82.6, 83.4) | 6 (4, 8) |
| **Camp** | 71.1 (70.5, 71.5) | 27 (25, 30) | 71.3 (70.7, 71.7) | 33 (31, 36) | 77.9 (77.3, 78.3) | 23 (22, 25) | 78.3 (77.6, 78.7) | 29 (27, 31) |
| **Carson** | 73.6 (72.9, 74.1) | 15 (13, 19) | 74.6 (73.8, 75.2) | 18 (15, 21) | 79.5 (78.9, 80.1) | 17 (14, 20) | 79.8 (79, 80.4) | 22 (18, 26) |
| **Cass** | 71.2 (70.6, 71.5) | 26 (25, 29) | 71.7 (71.1, 72.1) | 31 (30, 34) | 78 (77.6, 78.4) | 23 (22, 25) | 78.4 (77.9, 78.9) | 28 (27, 30) |
| **Castro** | 73.4 (72.6, 73.9) | 16 (14, 20) | 73.7 (72.8, 74.4) | 22 (19, 26) | 79.2 (78.5, 79.8) | 18 (15, 21) | 79.3 (78.4, 80.1) | 25 (21, 28) |
| **Chambers** | 73.5 (72.9, 74) | 16 (14, 19) | 74.1 (73.4, 74.7) | 20 (17, 23) | 79.1 (78.5, 79.7) | 19 (16, 21) | 79.8 (79, 80.4) | 22 (19, 26) |
| **Cherokee** | 71.9 (71.4, 72.3) | 23 (22, 25) | 71.9 (71.3, 72.3) | 30 (29, 33) | 78.5 (78, 78.8) | 21 (20, 23) | 79 (78.4, 79.5) | 26 (24, 29) |
| **Childress** | 71.6 (71, 72.1) | 24 (22, 27) | 72.2 (71.5, 72.8) | 29 (27, 32) | 78 (77.4, 78.6) | 23 (21, 25) | 77.9 (77.2, 78.6) | 30 (28, 33) |
| **Clay** | 72.3 (71.6, 72.7) | 22 (20, 25) | 73.2 (72.5, 73.7) | 24 (22, 28) | 78.7 (78.1, 79.1) | 20 (19, 23) | 79.6 (78.9, 80.2) | 23 (20, 27) |
| **Cochran** | 73.2 (72.7, 73.7) | 17 (15, 20) | 73.1 (72.5, 73.6) | 25 (23, 28) | 79.2 (78.6, 79.8) | 18 (15, 21) | 78.8 (78.1, 79.4) | 27 (24, 30) |
| **Coke** | 73 (72.3, 73.6) | 18 (16, 22) | 73.2 (72.3, 73.8) | 24 (21, 28) | 79.7 (79, 80.3) | 16 (12, 19) | 78.8 (78, 79.4) | 27 (24, 30) |
| **Coleman** | 73 (72.3, 73.5) | 18 (16, 22) | 73.1 (72.4, 73.6) | 25 (22, 28) | 79.5 (78.8, 80) | 17 (14, 20) | 79.5 (78.7, 80.1) | 24 (21, 28) |
| **Collin** | 77.8 (77.5, 78.1) | -2 (-3, -1) | 79.7 (79.4, 80) | -5 (-7, -3) | 81.5 (81.2, 81.8) | 6 (4, 7) | 82.5 (82.2, 82.8) | 8 (7, 9) |
| **Collingsworth** | 73.3 (72.7, 73.7) | 17 (15, 20) | 73.2 (72.6, 73.7) | 24 (22, 27) | 79.4 (78.8, 79.9) | 17 (15, 20) | 78.9 (78.3, 79.5) | 27 (24, 29) |
| **Colorado** | 72.9 (72.2, 73.4) | 19 (16, 22) | 73.5 (72.7, 74) | 23 (21, 27) | 78.6 (78, 79.1) | 21 (19, 23) | 79.3 (78.6, 79.9) | 25 (21, 28) |
| **Comal** | 75.6 (75.1, 76) | 6 (4, 8) | 76.8 (76.3, 77.2) | 8 (7, 10) | 81 (80.4, 81.4) | 8 (6, 11) | 81.8 (81.2, 82.3) | 11 (9, 14) |
| **Comanche** | 72.9 (72.3, 73.4) | 19 (16, 22) | 72.9 (72.2, 73.4) | 26 (23, 29) | 78.7 (78.1, 79.1) | 21 (19, 23) | 79.5 (78.9, 80.1) | 24 (21, 27) |
| **Concho** | 72.5 (71.7, 73) | 21 (18, 24) | 73.6 (72.8, 74.2) | 22 (20, 26) | 79.2 (78.5, 79.9) | 18 (15, 21) | 78.3 (77.5, 78.9) | 29 (26, 32) |
| **Cooke** | 74.1 (73.5, 74.5) | 13 (11, 16) | 74.8 (74.1, 75.3) | 17 (14, 20) | 79.7 (79.1, 80.1) | 16 (13, 19) | 80.6 (79.9, 81.2) | 17 (14, 22) |
| **Coryell** | 73.4 (72.9, 73.8) | 16 (14, 19) | 74.9 (74.3, 75.4) | 16 (14, 19) | 78.8 (78.2, 79.4) | 20 (18, 22) | 79.8 (79.2, 80.4) | 22 (19, 25) |
| **Cottle** | 72.6 (71.9, 73.1) | 20 (18, 23) | 73.2 (72.4, 73.8) | 24 (21, 28) | 79 (78.4, 79.6) | 19 (16, 22) | 78.7 (78, 79.4) | 27 (24, 30) |
| **Crane** | 72.2 (71.5, 72.8) | 22 (20, 25) | 73.4 (72.6, 74) | 23 (20, 27) | 77.8 (77.1, 78.4) | 24 (22, 26) | 78.5 (77.7, 79.1) | 28 (26, 31) |
| **Crockett** | 72.2 (71.5, 72.8) | 22 (20, 25) | 73.4 (72.6, 74) | 23 (20, 27) | 77.8 (77.1, 78.4) | 24 (22, 26) | 78.5 (77.7, 79.1) | 28 (26, 31) |
| **Crosby** | 72.6 (72.1, 73.1) | 20 (18, 23) | 72.9 (72.2, 73.4) | 26 (23, 29) | 78.9 (78.4, 79.5) | 19 (17, 22) | 78.7 (78.1, 79.3) | 27 (25, 30) |
| **Culberson** | 75.3 (75, 75.5) | 8 (7, 9) | 76.3 (76.1, 76.5) | 10 (9, 11) | 80.7 (80.4, 80.9) | 10 (9, 11) | 82 (81.8, 82.3) | 10 (9, 11) |
| **Dallam** | 73.4 (72.7, 73.9) | 16 (14, 20) | 73.7 (73, 74.3) | 22 (19, 25) | 79.3 (78.6, 79.8) | 18 (15, 21) | 78.9 (78.1, 79.5) | 27 (24, 30) |
| **Dallas** | 74 (73.9, 74.1) | 14 (13, 14) | 75.1 (75, 75.2) | 15 (15, 16) | 79.2 (79, 79.3) | 18 (18, 19) | 80.3 (80.2, 80.4) | 19 (19, 20) |
| **Dawson** | 73.2 (72.7, 73.7) | 17 (15, 20) | 73.1 (72.5, 73.6) | 25 (23, 28) | 79.2 (78.6, 79.8) | 18 (15, 21) | 78.8 (78.1, 79.4) | 27 (24, 30) |
| **De Witt** | 72.1 (71.5, 72.6) | 22 (20, 25) | 73.3 (72.5, 73.8) | 24 (21, 28) | 78.4 (77.8, 78.9) | 22 (20, 24) | 79.2 (78.5, 79.8) | 25 (22, 28) |
| **Deaf Smith** | 72.4 (71.7, 73) | 21 (18, 24) | 72.4 (71.6, 73.1) | 28 (25, 31) | 78.6 (77.9, 79.2) | 21 (18, 24) | 79.1 (78.2, 79.8) | 26 (22, 29) |
| **Delta** | 73.2 (72.4, 73.7) | 17 (15, 21) | 73.8 (73.1, 74.4) | 21 (19, 25) | 79.6 (78.9, 80.1) | 16 (13, 19) | 80 (79.2, 80.6) | 21 (17, 25) |
| **Denton** | 76.3 (76, 76.6) | 3 (2, 4) | 77.3 (77, 77.6) | 7 (6, 8) | 80.2 (79.8, 80.5) | 13 (11, 15) | 80.9 (80.6, 81.2) | 16 (14, 17) |
| **Dickens** | 72.6 (71.9, 73.1) | 20 (18, 23) | 73.2 (72.4, 73.8) | 24 (21, 28) | 79 (78.4, 79.6) | 19 (16, 22) | 78.7 (78, 79.4) | 27 (24, 30) |
| **Dimmit** | 69.9 (69.1, 70.5) | 35 (30, 45) | 72.2 (71.3, 72.8) | 29 (26, 33) | 77.9 (77.2, 78.5) | 24 (21, 26) | 78.1 (77.3, 78.8) | 30 (27, 33) |
| **Donley** | 73.6 (72.9, 74.1) | 15 (13, 19) | 74.6 (73.8, 75.2) | 18 (15, 21) | 79.5 (78.9, 80.1) | 17 (14, 20) | 79.8 (79, 80.4) | 22 (18, 26) |
| **Duval** | 72 (71.3, 72.6) | 23 (21, 26) | 73.4 (72.5, 74) | 23 (20, 28) | 78.9 (78.2, 79.5) | 20 (17, 22) | 78.6 (77.8, 79.2) | 28 (25, 31) |
| **Eastland** | 72.9 (72.3, 73.4) | 19 (16, 22) | 72.9 (72.2, 73.4) | 26 (23, 29) | 78.7 (78.1, 79.1) | 21 (19, 23) | 79.5 (78.9, 80.1) | 24 (21, 27) |
| **Ector** | 72.3 (71.9, 72.6) | 22 (20, 23) | 72.2 (71.8, 72.5) | 29 (28, 31) | 78.3 (77.9, 78.6) | 22 (21, 23) | 78 (77.6, 78.4) | 30 (29, 32) |
| **Edwards** | 72.8 (72, 73.3) | 20 (17, 23) | 73.7 (72.9, 74.3) | 22 (19, 26) | 79.4 (78.7, 80) | 17 (14, 20) | 79.9 (79.1, 80.6) | 21 (17, 26) |
| **El Paso** | 75.3 (75, 75.5) | 8 (7, 9) | 76.3 (76.1, 76.5) | 10 (9, 11) | 80.7 (80.4, 80.9) | 10 (9, 11) | 82 (81.8, 82.3) | 10 (9, 11) |
| **Ellis** | 73.1 (72.6, 73.4) | 18 (16, 20) | 75 (74.6, 75.3) | 16 (14, 18) | 79 (78.5, 79.3) | 19 (18, 21) | 78.9 (78.5, 79.2) | 27 (25, 28) |
| **Erath** | 74.1 (73.5, 74.6) | 13 (11, 16) | 73.9 (73.3, 74.5) | 21 (18, 24) | 79.8 (79.1, 80.3) | 15 (12, 19) | 79.6 (78.9, 80.2) | 23 (20, 27) |
| **Falls** | 70.6 (69.9, 71.1) | 29 (27, 35) | 70.9 (70.2, 71.4) | 35 (33, 39) | 77.5 (77, 78) | 25 (23, 27) | 76.7 (76, 77.2) | 35 (33, 38) |
| **Fannin** | 72 (71.5, 72.4) | 23 (21, 25) | 72.9 (72.3, 73.3) | 26 (24, 29) | 79.1 (78.6, 79.6) | 19 (16, 21) | 78.4 (77.8, 79) | 28 (26, 31) |
| **Fayette** | 74 (73.3, 74.5) | 14 (11, 17) | 74.1 (73.4, 74.6) | 20 (18, 23) | 80.2 (79.6, 80.7) | 13 (10, 16) | 79.9 (79.2, 80.5) | 21 (18, 25) |
| **Fisher** | 72.6 (72, 73.1) | 20 (18, 23) | 72.9 (72.3, 73.5) | 26 (23, 29) | 78.6 (78, 79.2) | 21 (18, 23) | 78.9 (78.1, 79.6) | 27 (23, 30) |
| **Floyd** | 73.3 (72.8, 73.8) | 17 (15, 19) | 73.8 (73.2, 74.3) | 21 (19, 24) | 79.7 (79.1, 80.2) | 16 (13, 19) | 79 (78.4, 79.6) | 26 (23, 29) |
| **Foard** | 72.6 (71.9, 73.1) | 20 (18, 23) | 73.2 (72.4, 73.8) | 24 (21, 28) | 79 (78.4, 79.6) | 19 (16, 22) | 78.7 (78, 79.4) | 27 (24, 30) |
| **Fort Bend** | 76.9 (76.6, 77.1) | 1 (0, 2) | 77.8 (77.5, 78) | 5 (4, 6) | 80.7 (80.4, 81) | 10 (8, 12) | 81.8 (81.5, 82.1) | 11 (10, 13) |
| **Franklin** | 73.2 (72.4, 73.7) | 17 (15, 21) | 73.8 (73.1, 74.4) | 21 (19, 25) | 79.6 (78.9, 80.1) | 16 (13, 19) | 80 (79.2, 80.6) | 21 (17, 25) |
| **Freestone** | 72.2 (71.5, 72.7) | 22 (20, 25) | 73.4 (72.7, 73.9) | 23 (21, 27) | 78.8 (78.2, 79.4) | 20 (17, 22) | 79.1 (78.5, 79.7) | 25 (22, 28) |
| **Frio** | 71.4 (70.7, 72) | 25 (23, 29) | 72.9 (72.1, 73.5) | 26 (23, 29) | 78.5 (77.8, 79.1) | 21 (19, 24) | 78.7 (77.8, 79.4) | 27 (24, 31) |
| **Gaines** | 72.5 (71.9, 73) | 21 (18, 23) | 73.9 (73.2, 74.5) | 21 (18, 25) | 78.3 (77.7, 78.9) | 22 (20, 24) | 78.9 (78.2, 79.5) | 27 (24, 29) |
| **Galveston** | 72.8 (72.5, 73) | 19 (18, 21) | 73.4 (73.1, 73.6) | 23 (22, 25) | 78.2 (77.9, 78.4) | 22 (21, 23) | 78.7 (78.4, 78.9) | 27 (27, 29) |
| **Garza** | 72.9 (72.2, 73.4) | 19 (16, 22) | 73.2 (72.4, 73.7) | 24 (22, 28) | 79 (78.3, 79.6) | 19 (16, 22) | 79.3 (78.6, 80) | 25 (21, 28) |
| **Gillespie** | 75.1 (74.5, 75.6) | 8 (6, 11) | 76.4 (75.7, 76.9) | 10 (8, 13) | 81 (80.3, 81.5) | 8 (6, 12) | 81.6 (80.9, 82.2) | 12 (9, 16) |
| **Glasscock** | 74.8 (74.4, 75.2) | 10 (8, 12) | 75.9 (75.4, 76.3) | 12 (10, 14) | 79.8 (79.4, 80.2) | 15 (13, 17) | 81.1 (80.6, 81.5) | 15 (13, 17) |
| **Goliad** | 73.1 (72.2, 73.6) | 18 (15, 22) | 73.3 (72.4, 73.9) | 24 (21, 28) | 79.2 (78.6, 79.8) | 18 (15, 21) | 79.8 (78.9, 80.5) | 22 (18, 27) |
| **Gonzales** | 71.8 (71.2, 72.3) | 24 (22, 26) | 72.1 (71.5, 72.6) | 29 (27, 32) | 79.1 (78.5, 79.6) | 19 (16, 21) | 79.1 (78.4, 79.7) | 26 (23, 29) |
| **Gray** | 73.3 (72.7, 73.7) | 17 (15, 20) | 73.2 (72.6, 73.7) | 24 (22, 27) | 79.4 (78.8, 79.9) | 17 (15, 20) | 78.9 (78.3, 79.5) | 27 (24, 29) |
| **Grayson** | 72.6 (72.2, 72.9) | 20 (19, 22) | 73.6 (73.2, 73.9) | 22 (21, 24) | 79.4 (79, 79.8) | 17 (15, 19) | 79.1 (78.7, 79.5) | 26 (24, 27) |
| **Gregg** | 72.2 (71.8, 72.5) | 22 (21, 24) | 71.8 (71.4, 72.1) | 31 (30, 33) | 78.1 (77.7, 78.4) | 23 (22, 24) | 78.7 (78.2, 79) | 28 (26, 29) |
| **Grimes** | 71.7 (71, 72.1) | 24 (22, 27) | 73.1 (72.5, 73.6) | 25 (22, 28) | 77.9 (77.3, 78.4) | 23 (21, 26) | 78.5 (77.8, 79.1) | 28 (26, 31) |
| **Guadalupe** | 74.9 (74.4, 75.3) | 9 (8, 12) | 76.5 (76, 76.9) | 9 (8, 12) | 80.3 (79.8, 80.7) | 12 (10, 15) | 81.1 (80.5, 81.6) | 15 (12, 18) |
| **Hale** | 73.3 (72.8, 73.8) | 17 (15, 19) | 73.8 (73.2, 74.3) | 21 (19, 24) | 79.7 (79.1, 80.2) | 16 (13, 19) | 79 (78.4, 79.6) | 26 (23, 29) |
| **Hall** | 71.6 (71, 72.1) | 24 (22, 27) | 72.2 (71.5, 72.8) | 29 (27, 32) | 78 (77.4, 78.6) | 23 (21, 25) | 77.9 (77.2, 78.6) | 30 (28, 33) |
| **Hamilton** | 73.1 (72.4, 73.6) | 18 (16, 21) | 73.8 (73.1, 74.4) | 21 (19, 25) | 79.2 (78.6, 79.7) | 18 (16, 21) | 79.5 (78.7, 80) | 24 (21, 27) |
| **Hansford** | 72.8 (72, 73.5) | 19 (16, 23) | 74.2 (73.3, 74.9) | 20 (16, 24) | 78.9 (78.2, 79.5) | 20 (17, 22) | 79.8 (78.9, 80.6) | 22 (18, 27) |
| **Hardeman** | 71.6 (71, 72.1) | 24 (22, 27) | 72.2 (71.5, 72.8) | 29 (27, 32) | 78 (77.4, 78.6) | 23 (21, 25) | 77.9 (77.2, 78.6) | 30 (28, 33) |
| **Hardin** | 72.1 (71.6, 72.6) | 22 (20, 25) | 73.5 (72.9, 73.9) | 23 (21, 26) | 78.5 (78, 78.9) | 21 (19, 23) | 78.3 (77.7, 78.7) | 29 (27, 31) |
| **Harris** | 74 (73.9, 74.1) | 14 (13, 14) | 75.8 (75.7, 75.8) | 12 (12, 13) | 79 (78.9, 79.1) | 19 (19, 20) | 80.5 (80.4, 80.6) | 18 (17, 19) |
| **Harrison** | 72 (71.6, 72.4) | 23 (21, 25) | 72.3 (71.8, 72.6) | 29 (27, 31) | 78 (77.6, 78.4) | 23 (22, 24) | 78.3 (77.8, 78.7) | 29 (27, 31) |
| **Hartley** | 73.4 (72.7, 73.9) | 16 (14, 20) | 73.7 (73, 74.3) | 22 (19, 25) | 79.3 (78.6, 79.8) | 18 (15, 21) | 78.9 (78.1, 79.5) | 27 (24, 30) |
| **Haskell** | 72.8 (72, 73.3) | 19 (17, 23) | 72.6 (71.7, 73.2) | 27 (24, 31) | 79.1 (78.5, 79.7) | 19 (16, 21) | 79 (78.1, 79.6) | 26 (23, 30) |
| **Hays** | 75.7 (75.2, 76.1) | 6 (4, 8) | 77 (76.5, 77.4) | 8 (6, 10) | 80.5 (80, 81) | 11 (8, 14) | 80.8 (80.2, 81.3) | 16 (14, 20) |
| **Hemphill** | 73.8 (73.1, 74.3) | 14 (12, 18) | 73.8 (73.1, 74.3) | 21 (19, 25) | 79.1 (78.5, 79.6) | 19 (16, 21) | 79.3 (78.6, 79.9) | 25 (22, 28) |
| **Henderson** | 72.3 (71.8, 72.7) | 22 (20, 24) | 73 (72.5, 73.4) | 25 (23, 28) | 78.5 (78, 78.9) | 21 (20, 23) | 79.2 (78.7, 79.7) | 25 (23, 28) |
| **Hidalgo** | 76.7 (76.4, 76.9) | 2 (1, 3) | 77.8 (77.5, 78) | 5 (4, 6) | 82 (81.7, 82.4) | 3 (2, 5) | 83.4 (83.1, 83.7) | 4 (2, 6) |
| **Hill** | 71.7 (71, 72.1) | 24 (22, 27) | 72.7 (72, 73.2) | 27 (25, 30) | 78.5 (77.9, 78.9) | 21 (19, 23) | 79.1 (78.4, 79.7) | 26 (23, 29) |
| **Hockley** | 72.6 (72.1, 73.1) | 20 (18, 23) | 72.9 (72.2, 73.4) | 26 (23, 29) | 78.9 (78.4, 79.5) | 19 (17, 22) | 78.7 (78.1, 79.3) | 27 (25, 30) |
| **Hood** | 74.5 (73.9, 75) | 11 (9, 14) | 75.7 (75.1, 76.2) | 13 (10, 15) | 80.1 (79.6, 80.7) | 13 (10, 16) | 80 (79.4, 80.6) | 21 (18, 24) |
| **Hopkins** | 72.3 (71.7, 72.7) | 22 (20, 24) | 73.6 (72.9, 74.1) | 22 (20, 26) | 79 (78.5, 79.5) | 19 (17, 21) | 78.9 (78.2, 79.4) | 27 (24, 29) |
| **Houston** | 71 (70.4, 71.4) | 27 (25, 31) | 72.3 (71.7, 72.8) | 28 (26, 31) | 77.5 (76.9, 77.9) | 25 (23, 27) | 77.8 (77.1, 78.3) | 31 (29, 33) |
| **Howard** | 72.4 (71.8, 72.8) | 21 (19, 24) | 72.4 (71.8, 72.8) | 28 (26, 31) | 78.1 (77.4, 78.5) | 23 (21, 25) | 77.8 (77.1, 78.3) | 31 (29, 33) |
| **Hudspeth** | 75.3 (75, 75.5) | 8 (7, 9) | 76.3 (76.1, 76.5) | 10 (9, 11) | 80.7 (80.4, 80.9) | 10 (9, 11) | 82 (81.8, 82.3) | 10 (9, 11) |
| **Hunt** | 72.5 (72.1, 72.9) | 21 (19, 23) | 73.1 (72.6, 73.5) | 25 (23, 27) | 79.1 (78.6, 79.5) | 19 (17, 21) | 78.7 (78.2, 79.2) | 27 (25, 29) |
| **Hutchinson** | 73.8 (73.1, 74.3) | 14 (12, 18) | 73.8 (73.1, 74.3) | 21 (19, 25) | 79.1 (78.5, 79.6) | 19 (16, 21) | 79.3 (78.6, 79.9) | 25 (22, 28) |
| **Irion** | 73.2 (72.8, 73.6) | 17 (15, 19) | 74.3 (73.9, 74.7) | 19 (17, 21) | 79.3 (78.8, 79.7) | 18 (16, 20) | 79.9 (79.4, 80.3) | 22 (19, 24) |
| **Jack** | 74 (73.3, 74.5) | 14 (11, 17) | 74.9 (74.2, 75.6) | 16 (13, 20) | 79.9 (79.2, 80.6) | 14 (10, 18) | 81.1 (80.2, 81.9) | 15 (11, 20) |
| **Jackson** | 72.9 (72.2, 73.4) | 19 (16, 22) | 73.7 (72.9, 74.3) | 22 (19, 26) | 79.2 (78.5, 79.7) | 18 (16, 21) | 79.7 (79, 80.4) | 23 (19, 26) |
| **Jasper** | 71.3 (70.7, 71.7) | 26 (24, 29) | 72.9 (72.3, 73.3) | 26 (24, 29) | 78.2 (77.7, 78.7) | 22 (21, 24) | 78.2 (77.6, 78.7) | 29 (27, 32) |
| **Jeff Davis** | 75.3 (75, 75.5) | 8 (7, 9) | 76.3 (76.1, 76.5) | 10 (9, 11) | 80.7 (80.4, 80.9) | 10 (9, 11) | 82 (81.8, 82.3) | 10 (9, 11) |
| **Jefferson** | 72.2 (72, 72.4) | 22 (21, 23) | 72.4 (72.2, 72.7) | 28 (27, 29) | 77.8 (77.6, 78) | 24 (23, 25) | 78.4 (78.1, 78.6) | 29 (28, 30) |
| **Jim Hogg** | 72 (71.3, 72.6) | 23 (21, 26) | 73.4 (72.5, 74) | 23 (20, 28) | 78.9 (78.2, 79.5) | 20 (17, 22) | 78.6 (77.8, 79.2) | 28 (25, 31) |
| **Jim Wells** | 72.1 (71.4, 72.5) | 23 (21, 25) | 72.8 (72, 73.4) | 26 (23, 30) | 79.2 (78.5, 79.7) | 18 (15, 21) | 79.8 (79, 80.4) | 22 (18, 26) |
| **Johnson** | 73.4 (73, 73.8) | 16 (15, 18) | 74.2 (73.8, 74.5) | 19 (18, 21) | 78.7 (78.2, 79) | 21 (19, 22) | 78.8 (78.3, 79.1) | 27 (26, 29) |
| **Jones** | 72.6 (72, 73.1) | 20 (18, 23) | 72.9 (72.3, 73.5) | 26 (23, 29) | 78.6 (78, 79.2) | 21 (18, 23) | 78.9 (78.1, 79.6) | 27 (23, 30) |
| **Karnes** | 71.8 (71.2, 72.3) | 24 (22, 26) | 72.1 (71.5, 72.6) | 29 (27, 32) | 79.1 (78.5, 79.6) | 19 (16, 21) | 79.1 (78.4, 79.7) | 26 (23, 29) |
| **Kaufman** | 72.3 (71.9, 72.7) | 21 (20, 23) | 72.7 (72.2, 73) | 27 (25, 29) | 78.2 (77.8, 78.6) | 22 (21, 24) | 77.6 (77.1, 77.9) | 32 (30, 33) |
| **Kendall** | 75.6 (74.9, 76.1) | 6 (4, 9) | 76.5 (75.8, 77) | 9 (8, 12) | 80.5 (79.8, 81) | 11 (8, 15) | 81.2 (80.5, 81.9) | 14 (11, 18) |
| **Kenedy** | 72.2 (71.4, 72.8) | 22 (20, 25) | 73.8 (72.9, 74.5) | 21 (18, 26) | 78.2 (77.5, 78.9) | 22 (20, 25) | 78.4 (77.5, 79.2) | 29 (25, 32) |
| **Kent** | 72.6 (72, 73.1) | 20 (18, 23) | 72.9 (72.3, 73.5) | 26 (23, 29) | 78.6 (78, 79.2) | 21 (18, 23) | 78.9 (78.1, 79.6) | 27 (23, 30) |
| **Kerr** | 75.1 (74.5, 75.6) | 8 (6, 11) | 75.5 (74.9, 76) | 14 (11, 17) | 80.8 (80.3, 81.3) | 9 (7, 12) | 81.5 (80.9, 82.1) | 13 (10, 16) |
| **Kimble** | 72.9 (72.2, 73.3) | 19 (17, 22) | 75.5 (74.7, 76) | 14 (11, 17) | 79 (78.4, 79.5) | 19 (17, 22) | 81 (80.3, 81.6) | 15 (12, 20) |
| **King** | 72.6 (71.9, 73.1) | 20 (18, 23) | 73.2 (72.4, 73.8) | 24 (21, 28) | 79 (78.4, 79.6) | 19 (16, 22) | 78.7 (78, 79.4) | 27 (24, 30) |
| **Kinney** | 71.6 (71, 72.2) | 24 (22, 27) | 74.2 (73.5, 74.8) | 20 (17, 23) | 78.5 (77.9, 79.1) | 21 (19, 23) | 80.1 (79.3, 80.8) | 20 (17, 25) |
| **Kleberg** | 73.1 (72.4, 73.6) | 18 (16, 21) | 73.6 (72.9, 74.2) | 22 (20, 26) | 78.7 (78.1, 79.3) | 20 (18, 23) | 79.6 (78.8, 80.2) | 23 (20, 27) |
| **Knox** | 72.8 (72, 73.3) | 19 (17, 23) | 72.6 (71.7, 73.2) | 27 (24, 31) | 79.1 (78.5, 79.7) | 19 (16, 21) | 79 (78.1, 79.6) | 26 (23, 30) |
| **La Salle** | 71.4 (70.7, 72) | 25 (23, 29) | 72.9 (72.1, 73.5) | 26 (23, 29) | 78.5 (77.8, 79.1) | 21 (19, 24) | 78.7 (77.8, 79.4) | 27 (24, 31) |
| **Lamar** | 72.5 (71.9, 72.9) | 21 (19, 23) | 72.5 (71.9, 72.9) | 28 (26, 30) | 78.4 (77.9, 78.7) | 22 (20, 23) | 78.2 (77.6, 78.6) | 29 (28, 31) |
| **Lamb** | 72.1 (71.3, 72.7) | 22 (20, 26) | 72.1 (71.3, 72.7) | 29 (27, 33) | 78.6 (77.9, 79.2) | 21 (18, 23) | 78.6 (77.7, 79.3) | 28 (25, 31) |
| **Lampasas** | 73.8 (73.1, 74.4) | 14 (12, 18) | 75.4 (74.6, 76) | 14 (11, 18) | 80.3 (79.5, 81) | 12 (8, 17) | 79.8 (79, 80.4) | 22 (18, 26) |
| **Lavaca** | 73.1 (72.4, 73.5) | 18 (16, 21) | 74.2 (73.5, 74.8) | 19 (17, 23) | 79.7 (79.1, 80.2) | 16 (13, 19) | 80.6 (79.9, 81.2) | 17 (14, 22) |
| **Lee** | 73.4 (72.7, 73.9) | 16 (14, 20) | 74.6 (73.9, 75.2) | 18 (15, 21) | 78.6 (78, 79.1) | 21 (19, 23) | 80.2 (79.5, 80.9) | 20 (16, 24) |
| **Leon** | 72.5 (71.7, 73) | 21 (18, 24) | 73.8 (73, 74.4) | 22 (19, 25) | 79.1 (78.5, 79.6) | 19 (16, 21) | 79.3 (78.5, 79.9) | 25 (22, 28) |
| **Liberty** | 70.7 (70.3, 71.1) | 28 (27, 31) | 71 (70.6, 71.4) | 34 (32, 36) | 77.3 (76.9, 77.7) | 25 (24, 27) | 77.3 (76.8, 77.7) | 33 (31, 34) |
| **Limestone** | 70.2 (69.6, 70.6) | 32 (29, 40) | 71 (70.3, 71.5) | 34 (32, 38) | 77.1 (76.6, 77.6) | 26 (25, 28) | 77.7 (77, 78.2) | 31 (29, 34) |
| **Lipscomb** | 73.8 (73.1, 74.3) | 14 (12, 18) | 73.8 (73.1, 74.3) | 21 (19, 25) | 79.1 (78.5, 79.6) | 19 (16, 21) | 79.3 (78.6, 79.9) | 25 (22, 28) |
| **Live Oak** | 73.1 (72.5, 73.6) | 18 (15, 21) | 74.6 (74, 75.1) | 18 (16, 21) | 79.6 (79, 80.1) | 16 (14, 19) | 80.4 (79.7, 81) | 19 (15, 23) |
| **Llano** | 75.3 (74.6, 75.8) | 7 (5, 11) | 76.6 (75.8, 77.2) | 9 (7, 12) | 80.8 (80.2, 81.4) | 9 (6, 13) | 81.3 (80.6, 82) | 14 (10, 18) |
| **Loving** | 72.3 (71.9, 72.6) | 22 (20, 23) | 72.2 (71.8, 72.5) | 29 (28, 31) | 78.3 (77.9, 78.6) | 22 (21, 23) | 78 (77.6, 78.4) | 30 (29, 32) |
| **Lubbock** | 73.3 (73, 73.5) | 17 (16, 18) | 74 (73.7, 74.2) | 21 (19, 22) | 79.1 (78.8, 79.3) | 19 (18, 20) | 79.2 (78.9, 79.5) | 25 (24, 27) |
| **Lynn** | 72.6 (72.1, 73.1) | 20 (18, 23) | 72.9 (72.2, 73.4) | 26 (23, 29) | 78.9 (78.4, 79.5) | 19 (17, 22) | 78.7 (78.1, 79.3) | 27 (25, 30) |
| **Madison** | 72 (71.4, 72.3) | 23 (22, 25) | 72.8 (72.1, 73.2) | 26 (24, 29) | 77.6 (77.1, 78) | 24 (23, 27) | 78.4 (77.7, 78.8) | 29 (27, 31) |
| **Marion** | 71.2 (70.6, 71.5) | 26 (25, 29) | 71.7 (71.1, 72.1) | 31 (30, 34) | 78 (77.6, 78.4) | 23 (22, 25) | 78.4 (77.9, 78.9) | 28 (27, 30) |
| **Martin** | 72.4 (71.8, 72.8) | 21 (19, 24) | 72.4 (71.8, 72.8) | 28 (26, 31) | 78.1 (77.4, 78.5) | 23 (21, 25) | 77.8 (77.1, 78.3) | 31 (29, 33) |
| **Mason** | 75.1 (74.5, 75.6) | 8 (6, 11) | 76.4 (75.7, 76.9) | 10 (8, 13) | 81 (80.3, 81.5) | 8 (6, 12) | 81.6 (80.9, 82.2) | 12 (9, 16) |
| **Matagorda** | 71.6 (71, 72) | 24 (23, 27) | 73.4 (72.7, 73.8) | 23 (21, 27) | 78.7 (78.1, 79.2) | 20 (18, 23) | 77.9 (77.3, 78.3) | 30 (29, 32) |
| **Maverick** | 71.6 (71, 72.2) | 24 (22, 27) | 74.2 (73.5, 74.8) | 20 (17, 23) | 78.5 (77.9, 79.1) | 21 (19, 23) | 80.1 (79.3, 80.8) | 20 (17, 25) |
| **McCulloch** | 72.5 (71.7, 73) | 21 (18, 24) | 73.6 (72.8, 74.2) | 22 (20, 26) | 79.2 (78.5, 79.9) | 18 (15, 21) | 78.3 (77.5, 78.9) | 29 (26, 32) |
| **McLennan** | 72.7 (72.4, 72.9) | 20 (19, 21) | 73.4 (73.1, 73.7) | 23 (22, 25) | 78.9 (78.6, 79.2) | 20 (18, 21) | 79.2 (78.9, 79.5) | 25 (24, 27) |
| **McMullen** | 71.4 (70.7, 72) | 25 (23, 29) | 72.9 (72.1, 73.5) | 26 (23, 29) | 78.5 (77.8, 79.1) | 21 (19, 24) | 78.7 (77.8, 79.4) | 27 (24, 31) |
| **Medina** | 73.4 (72.7, 73.9) | 16 (14, 20) | 75.3 (74.6, 75.8) | 14 (12, 18) | 80.1 (79.4, 80.6) | 14 (10, 17) | 79.8 (79.1, 80.4) | 22 (19, 26) |
| **Menard** | 75.1 (74.5, 75.6) | 8 (6, 11) | 76.4 (75.7, 76.9) | 10 (8, 13) | 81 (80.3, 81.5) | 8 (6, 12) | 81.6 (80.9, 82.2) | 12 (9, 16) |
| **Midland** | 74.8 (74.4, 75.2) | 10 (8, 12) | 75.9 (75.4, 76.3) | 12 (10, 14) | 79.8 (79.4, 80.2) | 15 (13, 17) | 81.1 (80.6, 81.5) | 15 (13, 17) |
| **Milam** | 73.1 (72.5, 73.6) | 18 (15, 21) | 73.3 (72.6, 73.8) | 24 (21, 27) | 79 (78.5, 79.5) | 19 (17, 21) | 78.2 (77.6, 78.7) | 29 (27, 32) |
| **Mills** | 73.1 (72.4, 73.6) | 18 (16, 21) | 73.8 (73.1, 74.4) | 21 (19, 25) | 79.2 (78.6, 79.7) | 18 (16, 21) | 79.5 (78.7, 80) | 24 (21, 27) |
| **Mitchell** | 72.2 (71.6, 72.6) | 22 (20, 25) | 72.1 (71.4, 72.5) | 30 (28, 32) | 78.4 (77.8, 78.9) | 21 (20, 24) | 78 (77.3, 78.6) | 30 (28, 32) |
| **Montague** | 72.3 (71.6, 72.7) | 22 (20, 25) | 73.2 (72.5, 73.7) | 24 (22, 28) | 78.7 (78.1, 79.1) | 20 (19, 23) | 79.6 (78.9, 80.2) | 23 (20, 27) |
| **Montgomery** | 73.7 (73.4, 73.9) | 15 (14, 16) | 75.6 (75.3, 75.8) | 13 (12, 14) | 79.1 (78.8, 79.4) | 19 (17, 20) | 80.2 (79.9, 80.4) | 20 (19, 22) |
| **Moore** | 73.4 (72.7, 73.9) | 16 (14, 20) | 73.7 (73, 74.3) | 22 (19, 25) | 79.3 (78.6, 79.8) | 18 (15, 21) | 78.9 (78.1, 79.5) | 27 (24, 30) |
| **Morris** | 71.1 (70.5, 71.5) | 27 (25, 30) | 71.3 (70.7, 71.7) | 33 (31, 36) | 77.9 (77.3, 78.3) | 23 (22, 25) | 78.3 (77.6, 78.7) | 29 (27, 31) |
| **Motley** | 72.6 (71.9, 73.1) | 20 (18, 23) | 73.2 (72.4, 73.8) | 24 (21, 28) | 79 (78.4, 79.6) | 19 (16, 22) | 78.7 (78, 79.4) | 27 (24, 30) |
| **Nacogdoches** | 72.2 (71.7, 72.6) | 22 (20, 24) | 72.6 (72, 73) | 27 (26, 30) | 78.2 (77.7, 78.6) | 22 (21, 24) | 78.8 (78.2, 79.2) | 27 (25, 29) |
[truncated: 66,745 more chars]
